# A Randomised Controlled Trial of SFX-01 After Subarachnoid Haemorrhage — The SAS Study

**DOI:** 10.1007/s12975-024-01278-1

**Published:** 2024-07-19

**Authors:** Ardalan Zolnourian, Patrick Garland, Patrick Holton, Mukul Arora, Jonathan Rhodes, Christopher Uff, Tony Birch, David Howat, Stephen Franklin, Ian Galea, Diederik Bulters

**Affiliations:** 1 Neurosurgery, University Hospital Southampton, Southampton, UK; 2 Clinical Neurosciences, Clinical and Experimental Sciences, Faculty of Medicine, University of Southampton, Southampton, UK; 3 Neuro Intensive Care, Royal Infirmary of Edinburgh, Edinburgh, UK; 4 Neurosurgery, Royal London Hospital, London, UK; 5 Medical Physics, University Hospital Southampton, Southampton, UK; 6 Evgen Pharma, Nether Alderley, UK; 7 Neurology, University Hospital Southampton, Southampton, UK

**Keywords:** Subarachnoid haemorrhage, Randomised clinical trial, Sulforaphane, Nrf2, Haptoglobin, Pharmacokinetics

## Abstract

SFX-01 is a novel drug for clinical delivery of sulforaphane (SFN). SFN is a potent nuclear factor erythroid 2-related factor 2 activator that reduces inflammation and oxidation, improving outcomes after subarachnoid haemorrhage (SAH) in animal models. This was a multi-centre, double-blind, placebo-controlled, parallel-group randomised clinical trial to evaluate the safety, pharmacokinetics and efficacy of 28 days of SFX-01 300 mg BD in patients aged 18–80 with spontaneous SAH and high blood load on CT. Primary outcomes were (1) safety, (2) plasma and CSF SFN and metabolite levels and (3) vasospasm on transcranial doppler ultrasound. Secondary outcomes included CSF haptoglobin and malondialdehyde and clinical outcome on the modified Rankin Scale (mRS) and SAH outcome tool (SAHOT). A total of 105 patients were randomised (54 SFX-01, 51 placebo). There were no differences in adverse events other than nausea (9 SFX-01 (16.7%), 1 placebo (2.0%)). SFN, SFN-glutathione and SFN-N-acetyl-cysteine AUC_last_ were 16.2, 277 and 415 h × ng/ml. Plasma SFN was higher in GSTT1 null individuals (*t* = 2.40, *p* = 0.023). CSF levels were low with many samples below the lower limit of quantification and predicted by the CSF/serum albumin ratio (*R*^2^ = 0.182, *p* = 0.039). There was no difference in CSF haptoglobin (1.981 95%CI 0.992–3.786, *p* = 0.052) or malondialdehyde (1.12 95%CI 0.7477–1.687, *p* = 0.572) or middle cerebral artery flow velocity (1.04 95%CI 0.903–1.211, *p* = 0.545) or functional outcome (mRS 1.647 95%CI 0.721–3.821, *p* = 0.237, SAHOT 1.082 95%CI 0.464–2.525, *p* = 0.855). SFX-01 is safe and effective for the delivery of SFN in acutely unwell patients. SFN penetrated CSF less than expected and did not reduce large vessel vasospasm or improve outcome. Trial registration: NCT02614742 clinicaltrials.gov.

**Supplementary Information:**

The online version contains supplementary material available at 10.1007/s12975-024-01278-1.

## Introduction

Subarachnoid haemorrhage (SAH) is a subtype of stroke that affects younger patients and has worse outcomes than other forms of stroke [1]. There is only one approved medical treatment (nimodipine) and despite this, poor outcome remains common. Even amongst those deemed to have made a good recovery, fatigue, memory impairment and cognitive deficits are frequent, affecting the resumption of activities and return to work [2, 3].

Nuclear factor erythroid 2-related factor 2 (Nrf2) is a redox-sensitive transcription factor. It is a global regulator of detoxifying enzymes [4] and regulates the degradation of red blood cells, haemoglobin, haem and iron through transcriptional upregulation of CD36 [5], haptoglobin [6], hemopexin [7], haem-oxygenase-1 [8] and ferritin [9]. Nrf2 is expressed in the CNS, upregulated after cerebral insults [10], and plays a key role in conditions where inflammation is the hallmark like SAH [11]. In animal SAH models, Nrf2 deletion leads to increased inflammation, oxidative stress, cerebral oedema, neuronal death and poor neurological outcome [12–14]. In humans, the minor T allele in the single nucleotide polymorphism rs10183914 in the *NRF2* gene is associated with poor outcome after SAH [15].

Nrf2 is regulated by Kelch-like ECH-associated protein 1 (KEAP1) in the cytoplasm which normally binds Nrf2. Oxidative stress leads to KEAP1 releasing Nrf2, which translocates into the nucleus leading to transcription [11]. Sulforaphane (SFN) stabilises Nrf2 through the inhibition of ubiquitination activating the Nrf2 pathway. In rodent models of SAH, SFN reduces early brain injury [12], and cerebral vasospasm reduces behavioural deficits, improving functional outcome [12, 16].

SFN is also protective in ischaemic stroke, reducing infarct volume by 30% [17] and improving neurological outcome in preconditioned animals [18]. Delayed cerebral ischaemia (DCI) occurs 3 days to 3 weeks after SAH and also provides a mechanism through which SFN is of benefit, but with a much larger therapeutic window.

SFN has a short half-life rendering it impractical for clinical use [19]. SFX-01 (Evgen Pharma) is a novel agent comprising SFN complexed with α-cyclodextrin. The α-cyclodextrin ring creates a ‘scaffold’ around the SFN stabilising it. On ingestion, SFN is released, providing an effective method to deliver SFN clinically. Two phase I trials (NCT01948362, NCT02055716) have shown that SFX-01 generates good plasma SFN levels with no serious adverse events and very limited side effects.

We therefore designed a randomised controlled trial to test the safety, pharmacokinetics and efficacy of up to 28 days of SFX-01 (300 mg) two times per day in patients within 48 h of aneurysmal SAH.

## Methods

This was a phase II double-blind, placebo-controlled, parallel-group trial in three tertiary neurosciences centres across the UK. A summary of the protocol has been published [20], and a full version is in Appendix A. The trial was conducted in accordance with the Declaration of Helsinki, met the international criteria for Good Clinical Practice and was approved by the National Research Ethics Service (Southern Central Hampshire A) and Medicinal Health Care Authority (MHRA), and was registered on clinicaltrials.gov (NCT02614742). Informed consents were obtained from patients or legal representatives. The Consolidated Standards of Reporting Trials (CONSORT) was followed.

### Patients and Eligibility Criteria

The inclusion criteria were as follows: (1) radiological evidence of spontaneous aneurysmal SAH, (2) Fisher grade 3 or 4, (3) 18 to 80 years, (4) within 48 h, (5) aneurysm treatment not ruled out, (6) previously independent, (7) informed consent from the patient, or legal representative within 24 h of the first dose.

Key exclusion criteria included the following: (1) plasma creatinine ≥ 2.5 mg/dL, (2) bilirubin ≥ twofold upper limit of normal, (3) pregnancy, (4) follow-up not feasible.

### Trial Procedures

After admission, identification by the research team and consent, patients were randomised in a 1:1 ratio to active or placebo. The active group received SFX-01 300 mg capsules. The placebo group received capsules containing α-cyclodextrin only. This was administered orally or via nasogastric tube twice daily for up to 28 days from ictus. Randomisation was stratified by the World Federation of Neurosurgical Societies (WFNS) grade, using sequential pre-numbered treatment packs. Treatment packs were prepared according to a block-balanced randomisation code by a blinded third party. Patients, nurses, clinicians, lab staff and investigators were blinded to allocation; capsules were identical in appearance.

### Outcomes

Full details of trial assessments are in the protocol. These include safety, pharmacokinetics, pharmacodynamics, clinical outcome and imaging data.

### Safety

Safety was assessed using treatment-emergent adverse event (TEAE) reporting. TEAEs were recorded throughout the 180 days of participation, coded following the Medical Dictionary for Regulatory Activities, graded for severity and followed until resolved. All data were captured and reviewed by the independent data safety monitoring board. Safety blood and urine (full blood count, urea and electrolytes, coagulation screen, liver function tests and urine microscopy) were obtained at baseline, post-dose and on days 7 and 28. Additionally, clinically obtained bloods were monitored until discharge.

### Pharmacokinetics

Although excellent plasma SFN levels have been demonstrated with the administration of SFN and SFX-01 in healthy volunteers, this was the first study targeting acutely unwell patients. Moreover, despite the extensive literature supporting SFN in neurological disorders, there is only one animal study (none in humans) quantifying brain penetration [21]. Therefore, paired CSF and plasma samples were obtained 7 days post-ictus. CSF was obtained via lumbar puncture (LP), or an external ventricular drain (EVD) when clinically available. In addition, 12 patients with an EVD consented to a pharmacokinetic sub-study to obtain CSF and blood samples 0, 1, 2, 3, 4, 5 and 6 h after dosing on days 3 and 7. Sample collection and analysis of SFN and its metabolites (glutathione (SFN-GSH) and N-acetyl cysteine (SFN-NAC)) are detailed in supplemental methods, and the validation report is in Appendix B.

### Vasospasm

To determine if SFX-01 reduced middle cerebral artery (MCA) flow velocity following SAH, transcranial doppler (TCD) ultrasound was performed on alternate days during the inpatient stay. MCA flow velocities (time average maximum) were measured bilaterally.

### Secondary Endpoints

Pharmacodynamic analysis of plasma and CSF malondialdehyde for oxidative stress and serum and CSF haptoglobin for transcriptional activity and haemoglobin-binding capacity upregulation was performed on day 7. In addition, a serum sample was obtained on day 28. CSF and serum samples were obtained from patients with EVDs on alternate days while they were in situ*.* Details of haptoglobin and malondialdehyde quantification are in supplemental methods.

DCI (new focal deficit or reduction in Glasgow Coma Scale ≥ 2 not explained by other causes) and initiation of hypertensive therapy were assessed daily. Outcome on the modified Rankin Scale (mRS) was recorded on day 7, discharge and days 28, 90 and 180 after SAH by a trained study nurse. At these visits, the extended Glasgow Outcome Scale (GOSE) [22], 36-Item Short Form Health Survey (SF-36) [23], Brain Injury Community Rehabilitation Outcome Scale (BICRO-39) [24], Checklist for Cognitive and Emotional consequences following stroke (CLCE-24) [25] and Subarachnoid Haemorrhage Outcome Tool (SAHOT) [26] were also obtained.

Glutathione S-Transferase (GST) was genotyped by Kompetitive Allele‐Specific PCR (KASP) at LGC Genomics for an exploratory analysis to assess the relationship of GSTM1 and GSTT1 status to levels of SFN in plasma and CSF.

### Statistical Analysis

The statistical analysis plan (SAP) is in appendix C. The safety analysis included any randomised patient who received at least one dose of study medication. All other analyses were performed on the per protocol population (dosed to day 7 post-ictus or more).

The primary endpoint was maximum (highest of all study visits) MCA flow velocity from an analysis of variance (ANOVA) model. The maximum MCA flow velocity per timepoint (highest side) was also analysed using a mixed model repeated measures (MMRM) approach.

Serum haptoglobin and plasma malondialdehyde concentrations were analysed using MMRM. CSF concentrations on day 7 were analysed using an analysis of covariance (ANCOVA) model with two levels for CSF source (EVD or LP) and an interaction between treatment and CSF source.

The proportion of patients with DCI and those receiving hypertensive therapy was analysed using logistic regression. mRS, GOSE and SAHOT were analysed using proportional odds logistic regression.

In further exploratory analyses, variables were tested for normality with the Shapiro–Wilk test, log transformed as necessary and correlation tested with a Pearson or *t*-test, and Spearman rank or Wilcoxon signed rank where variables remained non-normally distributed. Logistic regression was used for associations with binary outcomes and Tobit regression with the lower limit of quantification (LLOQ) set as left censored for association with SFN concentrations.

Planned analyses were performed in SAS (SAS Institute, Cary NC) and exploratory analyses in R 4.1.2.

## Results

**Fig. 1:**
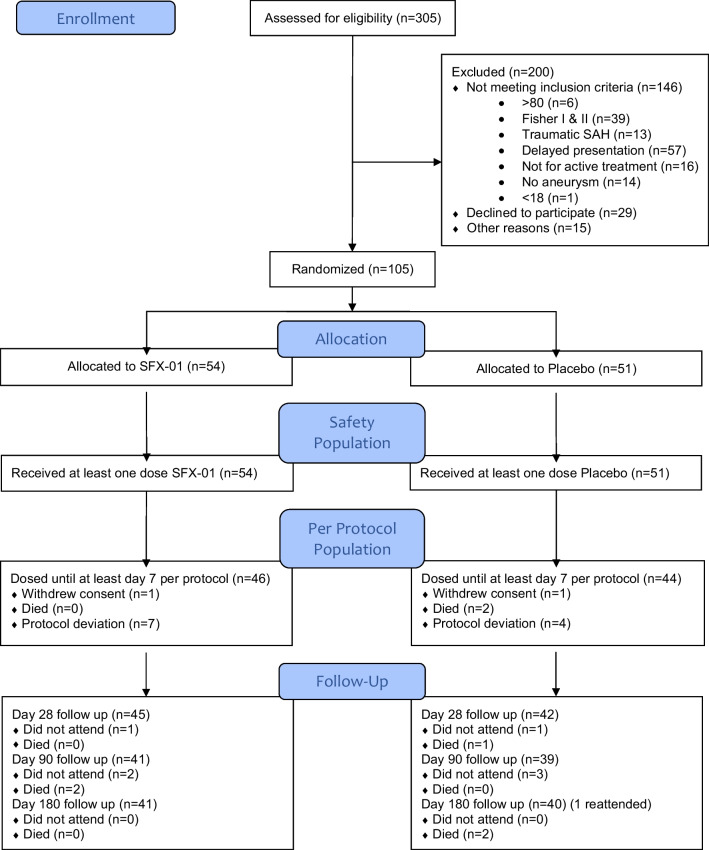
CONSORT flow diagram of patients screened and recruited to the SAS study

Between April 2016 and February 2019, 305 patients with SAH were screened. A total of 105 patients met the inclusion criteria and consented. Fifty-four patients were allocated SFX-01 and 51 placebo. All received at least one dose and are in the intent-to-treat safety analysis. Forty-six allocated SFX-01 and 44 allocated placebo are in the per protocol analyses (Fig. 1). Baseline characteristics are in Table 1.

**Table 1:** Baseline characteristics of the per-protocol population

Treatment	SFX-01	Placebo
Age (years), mean (SD)	55.1 (11.9)	55.3 (10.7)
Sex		
Male	13 (28.3%)	11 (25%)
Female	33 (72.7)	33 (75%)
Hypertension	14/46 (30.4%)	13/44 (29.5%)
Fisher grade		
3	16 (34.7%)	17 (38.6%)
4	30 (65.3%)	27 (61.4%)
WFNS		
1	19 (41.3%)	21 (47.8%)
2	12 (26%)	6 (13.6%)
3	0 (0%)	6 (13.6%)
4	13 (28.3%)	8 (18.2%)
5	2 (4.4%)	3 (6.8%)
Aneurysm location		
Anterior circulation	36 (78.2%)	40 (93%)
Posterior circulation	10 (21.8%)	3 (7%)
Aneurysm treatment		
Clip	12 (26%)	10 (22.7%)
Coil	34 (74%)	33 (75%)
Conservative	0 (0%)	1 (2.3%)
Time from ictus to first dose (hrs), mean (SD)	29.1 (12.5)	32.3 (14.4)
Duration of treatment (days), mean (SD)		
	23.3 (6.0)	23.1 (6.5)

Fifty-three patients (96.1%) allocated SFX-01 and 48 (94.1%) placebo experienced a TEAE (Table S1). The only difference was nausea in nine (16.7%) receiving SFX-01 and one (2.0%) receiving placebo. Vomiting occurred in five (9.3%) allocated SFX-01 and two allocated placebo (3.9%). Two patients taking SFX-01 and none taking placebo discontinued medication due to nausea or vomiting. There were no differences in haematological or biochemical parameters at any time (days 1–5, 6–8, 9–14, 15–21, 22–30, > 30). There were four deaths in both groups.

### Sulforaphane and Metabolite Plasma and CSF Levels

**Fig. 2:**
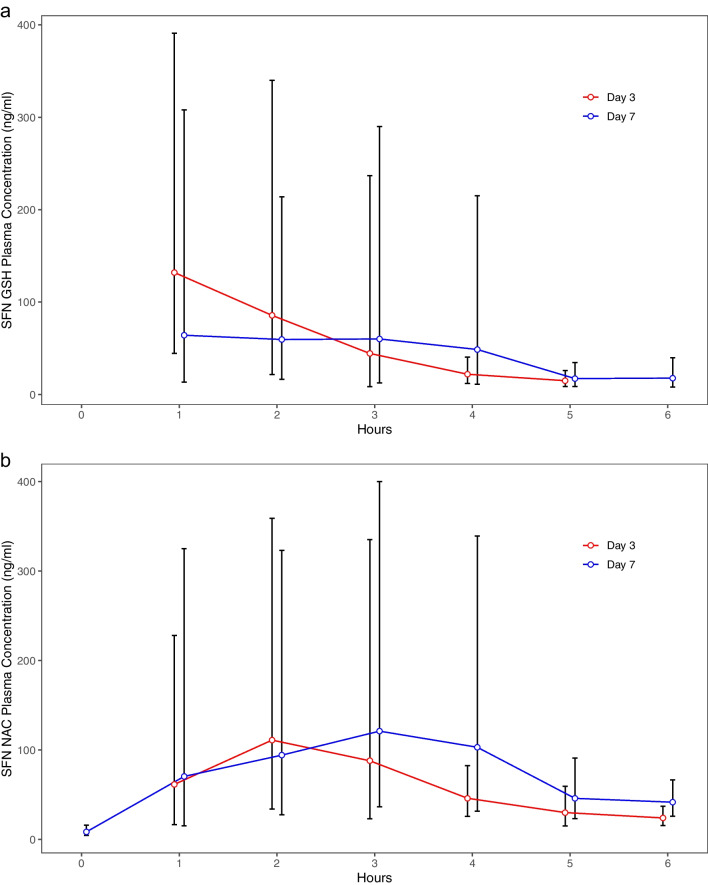
Plasma concentration of **a** SFN-GSH and **b** SFN-NAC in eight patients in the pharmacokinetic sub-study. Geometric mean ± sd

Eight patients in the pharmacokinetic sub-study undergoing hourly plasma and CSF sampling were allocated SFX-01. Plasma SFN-GSH and SFN-NAC are displayed in Fig. 2 and Table S2. At every timepoint, there was at least one patient with plasma SFN below LLOQ, precluding calculation of a geometric mean as in the SAP.

There was at least one patient with CSF SFN, SFN-GSH and SFN-NAC below LLOQ at every timepoint, precluding analysis as per SAP. Five of 95 samples taken 1, 2, 3, 4, 5 and 6 h after dosing for SFN on days 3 and 7 were above LLOQ (geometric mean 105 geometric sd 4.41). Five of 96 samples for SFN-GSH (geometric mean 11.6 geometric sd 1.13) and none of 96 for SFN-NAC were above LLOQ.

**Fig. 3:**
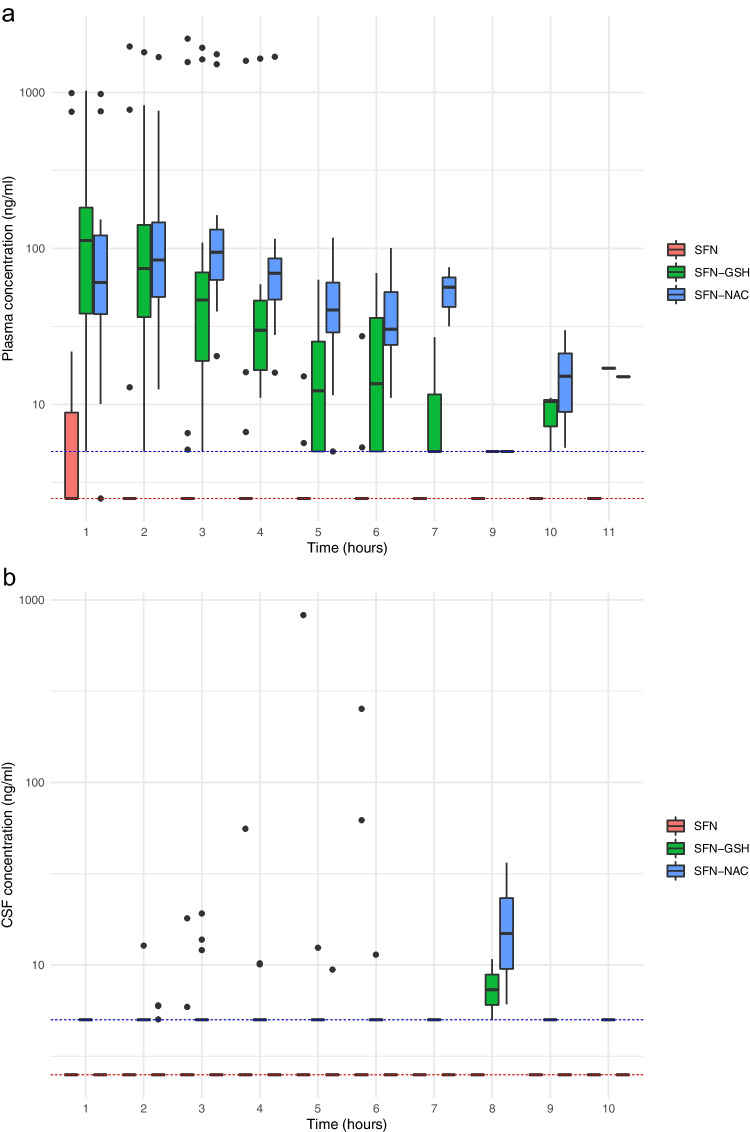
All plasma (**a**) and CSF (**b**) SFN, SFN-GSH, SFN-NAC concentrations for all patients in the per protocol population randomised to SFX-01. Values below LLOQ (5 ng/ml SFN, 10 ng/ml SFN-GSH, 5 ng/ml SFN-NAC) are represented as the midpoint between 0 and the LLOQ (dotted red line for SFN and SFN-NAC and blue for SFN-GSH). Median, 25th and 75th percentile and minimum and maximum (minimum or maximum value in the data within 1.5*IQR of 25th or 75.^th^ percentile)

Including the eight sub-study patients, 45 of 46 in the per protocol population had a plasma sample taken, and 40 of 46 had a CSF sample taken on day 7. All available plasma and CSF SFN, SFN-GSH and SFN-NAC measurements for patients receiving SFX-01 are shown in Fig. 3.

### Determinants of Plasma SFN

**Fig. 4:**
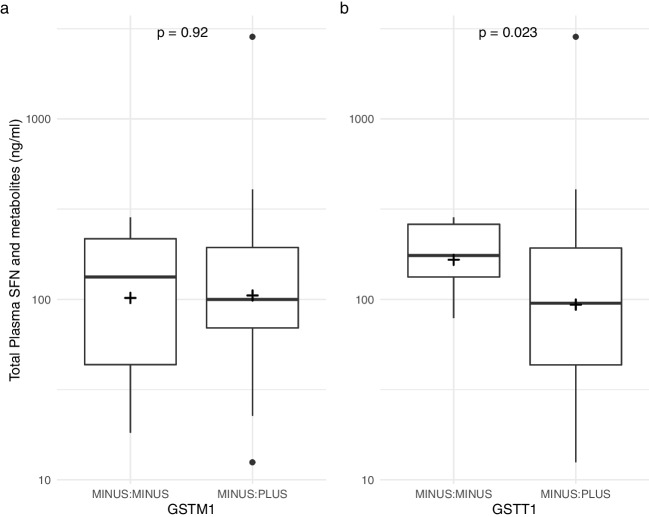
Total plasma SFN and metabolites and **a** GSTM1 and **b** GSTT1 status. *P*-value on *t*-test. Median, 25th and 75th percentile and minimum and maximum (minimum or maximum value in the data within 1.5*IQR of 25th or 75th percentile). Means depicted with +

Given lower than anticipated CSF SFN and metabolite levels, an exploratory analysis was undertaken to ascertain determinants of plasma and CSF concentrations. First, the total of the plasma SFN and each metabolite (SFN + SFN-GSH + SFN-NAC) was calculated for each patient (using the mean on day 7 for patients with multiple samples in the pharmacokinetic sub-study). The mean total plasma SFN correlated with age (*r*(41) = 0.38, *p* = 0.012, Figure S1), but not weight (*r*(41) = 0.06, *p* = 0.731), height (*r*(41) = 0.09, *p* = 0.580), Body Mass Index (*r*(41) = 0.03, *p* = 0.875, Figure S2) or sex (*t*(41) = 0.52, *p* = 0.606, Figure S3). There was no difference with GSTM1 status (*t*(40 =  − 0.10, *p* = 0.922), but there was with GSTT1 (*t*(41) = 2.40, *p* = 0.023, Fig. 4 a and b).

Tobit regression showed age and GSTT1 explained 9.1% of variance (*R*^*2*^ = 0.091, Wald = 12.3, *p* = 0.002) in mean total plasma SFN and metabolites. Age (*β* = 0.035, *p* = 0.003) and GSTT1 status (*β* =  − 0.666, *p* = 0.039) were independent predictors.

### Determinants of CSF SFN

Due to infrequent detection of SFN in CSF, patients were dichotomised as having SFN or metabolites detected in any of their day 7 samples or not. Backward logistic regression to predict the presence of CSF SFN or metabolites above the LLOQ with total plasma SFN and metabolites, CSF sampling method and QAlb as predictors showed that only QAlb was associated (pseudo *R*^2^ = 0.182, Wald = 2.060, *p* = 0.039).

Otherwise, there were no differences between patients with or without detectable CSF SFN or metabolites in age, BMI, WFNS, blood volume on CT, endovascular/microsurgical treatment (Figure S4), CSF sampling method (LP or EVD) (Figure S5), or drop in red blood cells between first and fourth CSF samples (where obtained by LP) (Figure S6).

### Haptoglobin and Malondialdehyde

There was no difference in CSF haptoglobin (SFX-01 2.27 vs placebo 1.17 mg/L, ratio 1.981 95%CI 0.992–3.786, *p* = 0.052) or malondialdehyde (SFX-01 0.116 vs placebo 0.103 g/L, ratio 1.123 95%CI 0.747–1.687, *p* = 0.572). Serum, plasma and CSF levels are in Table S3.

### Transcranial Doppler Ultrasound

**Fig. 5:**
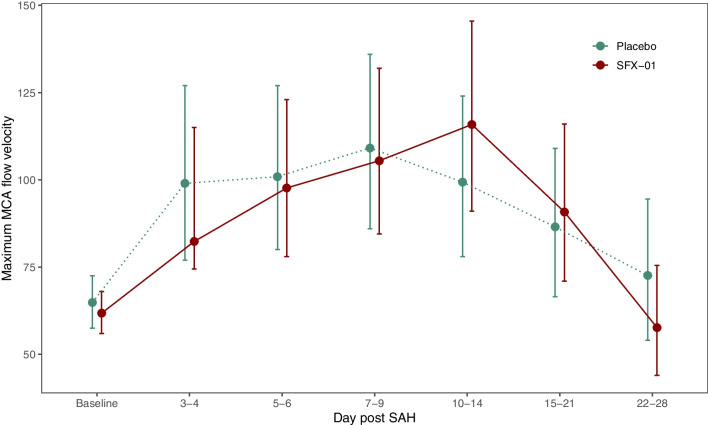
Plot of geometric least squares means over time by treatment for the middle cerebral artery (MCA) mean flow velocity in the per protocol population. Bars indicate the 95% confidence interval. Geometric mean and CI based on standard error of baseline mean are presented at baseline

Maximum MCA flow velocity did not differ between groups (ratio of means 1.046 95% CI 0.903–1.211 *p* = 0.545, Table S4). There were no differences on MRMM analysis at each time point (Table S5 and Fig. 5).

### Clinical Outcomes

**Fig. 6:**
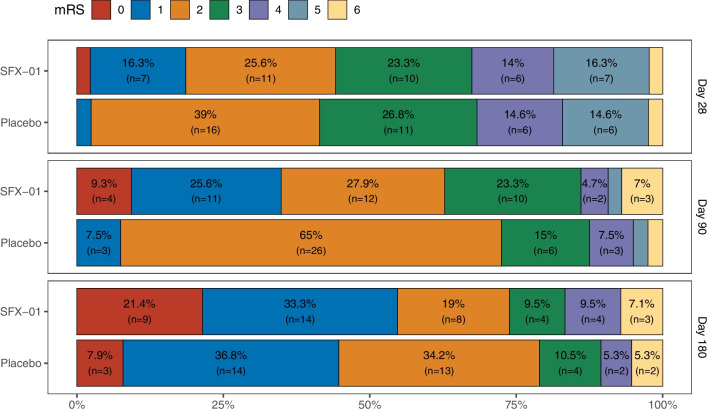
Stacked bar chart for modified Rankin scores at days 28, 90 and 180 in the per protocol population

There was no difference in the incidence of DCI, use of hypertensive therapy, mRS (Fig. 6), GOSE, SF-36, SAHOT, BICRO-39 or CLCE-24 between groups at any timepoint (Tables S6-S10).

## Discussion

In this multicentre double-blinded RCT, we show that oral SFX-01 can be safely administered to an acutely unwell SAH population, with few side effects and excellent plasma concentrations of sulforaphane and its metabolites. CSF penetration was lower than anticipated and we were unable to demonstrate engagement of target mechanisms in the brain (haemoglobin scavenging, oxidative stress, vasospasm) or detect signals of improved clinical outcome.

The safety data is very reassuring, and while SFN and SFX-01 are known to have excellent safety profiles, this was the first time either had been used in large numbers in an acutely unwell population, with many patients intubated in intensive care. This population has high rates of adverse events including haemodynamic, respiratory, haematological, biochemical and infective. None was worsened by SFX-01.

GI dysmotility and gastric intolerance are common in critically ill patients [27], in SAH and with high dose SFN. It was therefore reassuring that with SFX-01 (often given via nasogastric tube), only a small increase in nausea was seen and that plasma SFN levels were similar to smaller studies in healthy participants [28].

CSF levels were lower than anticipated. Many samples were below LLOQ. For this reason, it was difficult to estimate what typical CSF levels were, and we can only comment that they were at least an order of magnitude lower than plasma levels. This was surprising given the extensive literature on SFN for CNS conditions in animals. Few of these have quantified SFN, probably because it is a small lipophilic molecule assumed to readily cross the BBB. There is only one animal study of CSF SFN (none in humans). This found SFN in brain homogenate was fivefold lower than in plasma [21]. There are multiple possible technical explanations for this discrepancy. Animals in that study were not perfused; hence, a contribution from intravascular blood would have led to artefactually high estimates of brain SFN concentration. The SFN doses used in the animal study were two and ten times higher than in our study (based on body surface area conversion) and the relationship between plasma and brain may not be linear across concentrations. Mouse and human BBB permeability, and therefore distribution, may also differ. It is also important to note that CSF is produced by the choroid plexus, which is a highly metabolically active tissue. It is possible that SFN and its metabolites were consumed by the choroid plexus leading to different brain and CSF concentrations. A microdialysis study to determine SFN penetration in the tissue through the BBB proper would therefore be valuable.

Given there was considerable interindividual variability of plasma SFN and low CSF SFN levels, we undertook exploratory analyses of predictors of plasma and CSF SFN levels. Plasma SFN was correlated with age as is observed with many drugs [29]. There was no correlation with sex, weight, height or BMI. Although unusual, some drugs do not show any relationship with size [30]. It means weight-based dosing is unlikely to improve CSF concentrations. SFN is metabolised by GST. Two common gene polymorphisms are GSTM1 and GSTT1 deletions. Their reported relationship to urinary SFN metabolite excretion varies [31, 32], and one study of 16 volunteers reported plasma SFN was higher in GSTM1 null individuals [33]. We saw no similar relationship but did observe a more marked increase in GSTT1 null individuals.

The only other explanation of plasma variability that we did not specifically study was the influence of mode of drug delivery (oral or nasogastric tube). While drugs like nimodipine are widely given nasogastrically after SAH, this may influence bioavailability. However, there are considerable confounders in any analysis of this given patients fed by NG tube are much more likely to have gastric dysmotility. Moreover, the majority of patients would have had periods fed orally and periods via nasogastric tube without washout period making comparisons difficult.

We next examined if variability in CSF SFN was related to plasma SFN or QAlb (surrogate for BBB permeability). QAlb did predict the presence of SFN in CSF. This would be in keeping with it having limited BBB permeability. Furthermore, plasma SFN did not predict its presence in the CSF which suggests it is driven more by BBB permeability than plasma concentration (or alternative mechanisms like active transport out of the central nervous system by BBB transporters as seen with some drugs but not reported for sulforaphane).

BBB permeability can be modelled in silico [34], and models support that permeability to SFN and its metabolites may not be as free as had previously been thought. They predict SFN has borderline permeability (personal communication with Andriy Kovalenko) and that SFN-GSH is permeable and SFN-NAC impermeable. The only other explanations of why CSF levels in this study were low remain either technical which we believe we have ruled out or due to consumption in the choroid plexus which we are unable to prove or disprove with the available data.

Options for increasing CSF SFN by increasing the oral SFX-01/SFN dose are limited. The dose used was selected based on allometric scaling and is slightly higher than the most common dose in rodent studies in SAH and other CNS conditions. Human phase 1 studies showed increasing gastrointestinal adverse events at higher doses. Increasing frequency of dosing is also unlikely to increase efficacy of SFN, since it acts through transcriptional upregulation. The only remaining option would be to deliver SFX-01 intrathecally, but this route is not available to the majority of patients and would require extensive further preclinical testing that is not currently being pursued.

Although CSF SFN levels were low, this does not necessarily mean they were not high enough to engage target pathways. Target engagement has been demonstrated with 5 mg/kg SFN in rodents [35], equating to 60% of the SAS dose. This rodent dose is widespread in the literature supporting SFN in neurological conditions, despite no quantification of brain or CSF levels in any of these studies [11, 12, 16–18]. It has specifically been shown to increase haptoglobin (a haemoglobin-binding protein essential for its neutralisation and degradation) by 1.6-fold in the brain [6]. It is therefore intriguing that we observed a similar numerical difference of twofold in CSF haptoglobin levels (*p* = 0.052). However, we did not see any similar increase in serum levels or reduction in oxidative stress (malondialdehyde), or vasospasm or any downstream clinical outcomes.

Given these possible signals, it would be interesting to have further assessment of the engagement of the Nrf2 pathway and blood samples were obtained for transcriptomic analysis. However, the compartment of interest is the CNS. Transcriptomic analysis here is complicated by the presence of a large number of cells in the blood clot making interpretation of CSF transcriptomics potentially difficult and requiring brain biopsies which would not be practical. Transcriptional activity in the CNS is therefore best assessed by protein expression. However, most proteins are intracellular and their concentrations likely to be confounded by their release with cellular damage after SAH. Haptoglobin is secreted which made it particularly suited as a readout in addition to the direct relevance of its biological effects to SAH.

Ultimately, it is not possible to confidently conclude whether the lack of clinical benefit in this trial was due to inadequate CSF penetration, SFN’s failure to engage target mechanisms or if SAH provides such a strong stimulus for Nrf2 activation that the pathway is fully saturated and SFN provides no additional benefit (although this would be contrary to the animal literature [12, 16]), or if effect sizes were just too small to detect.

Going forwards, options to better target Nrf-2 with SFN in patients with SAH are limited. We chose what appears to have been the optimal/maximal dose for the existing formulation given side effects were just beginning to develop in a small subset of patients. Dosing based on weight or BMI is unlikely to significantly alter this given the lack of association with plasma levels. Given that we saw relatively little signal in any of the efficacy endpoints, it is unlikely that a formulation change will be enough to overcome this. Haptoglobin was the only endpoint showing promise, but any change would need to be orders of magnitude larger to overcome the haemoglobin released by the clot and impact clinical outcome. The only real option would be to consider intrathecal sulforaphane, but this would come with considerable additional complexity.

### Limitations

The study was limited to Fisher grade 3 and 4 patients to select those with high blood load and greatest benefit from the intervention and may not be generalisable to all SAH. The Fisher scale also has many limitations. However, we have additionally quantified blood volume on CT, confirming it was a homogenously severe SAH population.

Patients were enrolled up to 48 h after ictus. While that may seem long, it is shorter than most SAH studies, and actual time to treatment was much shorter (29.1 h). Given that many effects of SAH occur after 72 h and SFN upregulates the Nrf2 transcriptome within hours, it is unlikely that earlier administration would have altered our findings.

Vasospasm as adjudged with TCD was used as a primary outcome. It is increasingly recognised that the contribution of large vessel vasospasm to poor outcome is limited and not all vasospasm leads to poor outcome. An alternative measure such as DCI may have been more clinically relevant. However, given that this was a Phase 2 study, it was optimised to detect engagement of biological mechanisms (and not clinical outcome). SFX-01 mechanism of action is to improve haemoglobin clearance and reduce oxidative stress. This should be reflected by a reduction contraction in cerebral vessels and reduction in MCA flow velocity irrespective of whether this would prove the mechanism by which it improved outcome. This process is continuous and not binary. Preserving the continuous nature of MCA flow velocity gives it considerably greater statistical power to detect a difference in groups compared to a binary outcome like delayed cerebral ischaemia (or a binarized MCA flow velocity to create a vasospasm vs no vasospasm group) and was therefore much more likely to be able to detect if SFX-01 was having a biological effect or not and was therefore selected for the purposes of this phase 2 study to reduce sample size.

Despite groups being randomised, there was some imbalance in number of patients with posterior circulation aneurysms between treatment arms. This has occurred by random chance. It could be hypothesised that posterior circulation aneurysms result in a less pronounced increase in MCA flow velocity than those in the anterior circulation, and this could have altered our findings. However, given there were more patients in the treatment group with posterior circulation aneurysms, this would have only increased the chance of seeing a difference between groups, which was not observed. The imbalance could also have led to more patients expected to have poor outcome being randomised to the SFX-01 group and thereby have masked observation of a clinical effect of the treatment, but the differences in outcome between anterior and posterior circulation aneurysms are small, and we think this unlikely to have been the case.

Fifteen patients with the intention to treat safety analysis did not meet the criteria for the per-protocol analysis. The majority of these were due to patient death before completion of the course of the study drug. The remainder were due to patient withdrawal from the study or missing drug doses. These are inherent in clinical trials necessitating separate analyses. Intention to treat analysis was deemed most appropriate for safety, but per-protocol analysis was more appropriate for mechanistic understanding.

Sampling from either EVD or LP, rather than one CSF source, is a limitation given the known differences in lumbar and ventricular CSF composition. However, a study limited to patients with EVDs would take longer to complete and be less generalizable. Moreover, analyses were corrected for CSF source. LP could also result in higher lumbar SFN concentrations due to contamination by blood, although we found no relationship with the degree of trauma.

While sample timing in the pharmacokinetic sub-study was carefully controlled, CSF samples in the whole study population were not standardised relative to dosing. This was because their primary purpose was to study downstream mechanisms sensitive to day after SAH (but not time of day) and the anticipated practical difficulties performing LPs in a tight time window. Paired CSF and plasma samples were therefore obtained at random times after SFX-01 dosing and while this represents a random spread, there was a paucity of samples within one or two hours when levels are likely to have peaked.

As described, some analyses were undertaken post-hoc to try to better understand unanticipated findings in the prospectively defined outcomes. These analyses are inherently exploratory in nature.

Finally, while a GLP certified laboratory performed SFN quantification, the assays are limited by their LLOQ. Therefore, although many samples were below LLOQ, this does not mean that SFN or its metabolites were absent, and an assay with a lower LLOQ may have detected the analytes.

## Conclusion

SFX-01 is a safe drug for delivery of SFN in acutely unwell patients achieving excellent plasma levels. Plasma levels are influenced by age and GSTT1 status but not weight or BMI. SFN CSF penetration is lower than expected and was not related to plasma levels but was related to BBB permeability suggesting it cannot be simply overcome by higher dosing or dosing to weight. Other than increasing CSF haptoglobin, there was little evidence SFN engaged target pathways in the CNS with no influence on middle cerebral flow velocity or oxidative stress and no influence on clinical outcome. More research is needed into the CNS penetration of SFN and its metabolites.

## Supplementary Information

Below is the link to the electronic supplementary material.

Supplementary file1 - Consort checklist (DOC 219 KB)

Supplementary file2 - Protocol (PDF 672 KB)

Supplementary file3 - Validation report (PDF 6174 KB)

Supplementary file4 - Statistical analysis plan (PDF 894 KB)

Supplementary file5 - Figures (DOCX 232 KB)

Supplementary file6 - Methods (DOCX 14 KB)

Supplementary file7 - Tables (DOCX 28 KB)

## Data Availability

Requests for derived data supporting the study findings will be considered by the corresponding author.
