# Supplementary material for: A Randomised Controlled Trial of SFX-01 After Subarachnoid Haemorrhage — The SAS Study
Source: Transl Stroke Res. 2024 Jul 19;16(4):1031–43. doi: 10.1007/s12975-024-01278-1 (PMC12202693; doi:10.1007/s12975-024-01278-1)
Supplement: Supplementary file 2 — Supplementary file2 - Protocol (PDF 672 KB) [file 12975_2024_1278_MOESM2_ESM.pdf]

CONFIDENTIAL

SFX-01 AFTER SUBARACHNOID HAEMORRHAGE

(SAS STUDY)

PROTOCOL

**Version:**	Version 7
**Version date:**	16th May 2018
**Superseded version:**	Version 6 (22nd December 2017)
**Sponsor:**	Evgen Pharma plc
**Product:**	SFX-01
**Study Number(s):**	EVG001SAH
	EudraCT number: 2014-003284-38

Amendment History

The original protocol was issued 26 November 2015. Amendments are listed beginning with the most recent.

Amendment		Implemented
6	Contact Names and Addresses<br><br>Medical Monitor Contact details updated<br>Diamond Pharma Services Contact details updated	V7<br><br>16th May 2018
6	2.4. Secondary Outcome Endpoints<br>Correction of inconsistency: Removal of SFN analysis for alternate day EVD sampling; addition of Proteomic & Genomic analysis alongside HP & MDA sampling at D7	V7<br><br>16th May 2018
6	7.7. Patients with an External Ventricular Drain fitted: Ongoing EVD single sampling (on alternate days +/- 1day from day of EVD fitting until D14 or until the EVD is removed)<br><br>Correction: deletion of SFN levels from alternate day blood and CSF sampling in EVD group	V7<br><br>16th May 2018
6	7.10. Day 28 post ictus (-6/+2 days):<br>Correction: deletion of blood sampling for SFN level	V7<br><br>16th May 2018
6	7.15. Schedule of Assessments<br>Clarification that alternate day paired CSF/Blood samples are analysed for HP & MDA/ HP & MDA & Proteomic & Genomic respectively	V7<br><br>16th May 2018
6	8.1 Safety measurements<br><br>Correction: Lipid Profile LDH to LDL	V7<br><br>16th May 2018
6	8.3 PK measurements<br><br>Clarification: Sample schedule	V7<br><br>16th May 2018
6	8.6.2 Pregnancy Testing<br><br>Clarification: menstrual status	V7<br><br>16th May 2018
6	8.6.4 EVD Sampling<br>Clarification that the CSF sample taken at day 7 is approximately 20 ml.	V7<br><br>16th May 2018
5	Section 4.1 Subject Recruitment, 7.2. Pre-Dose Assessment (within 48 hours of ictus) & 14 INFORMED CONSENT	V6<br><br>22nd December 2017

	Clarification that emergency dosing (first two doses) is only permissible where local regulations allow; if local regulations do not allow emergency dosing without consent the patient shall not be enrolled into the study.	
5	Section 3.2 Duration of Treatment, 7.9 Within 2d of discharge, Schedule of Assessment and Synopsis<br><br>“discharge” defined:<br>Discharge is where the consultant responsible for the intervention of treating the SAH decides that their specialist care is no longer needed and they can safely transfer that care to another health care professional or send the patient home.	V6<br><br>22nd December 2017
5	Section 3.2. Duration of Treatment, Section 6.3. Packaging & Storage<br>& 6.6. Drug Storage<br>Clarification that storage conditions are 2-8°C (and that patients will be provided with a cool bag for transportation of the IMP to home)	V6<br><br>22nd December 2017
5	Section 3.3.3. Replacements<br>Clarification that patients that have potentially received insufficient or incorrect study medication may be replaced.	V6<br><br>22nd December 2017
5	Synopsis & Section 4.2 Inclusion Criteria:<br><br>Clarification:<br>1. Patients with radiological evidence of spontaneous **aneurysmal** SAH	V6<br><br>22nd December 2017
5	Section 9. ADVERSE EVENT REPORTING<br>Clarification that All AEs will be reported until 30 days after the last dose AEs occurring after 30 days must also be reported if considered related to study drug.	V6<br><br>22nd December 2017
5	11.1. Sample Size<br>Addition that up to 120 patients may be recruited and enrolled into the trial in order to provide 90 who will meet the per protocol criteria	V6<br><br>22nd December 2017
4	Section 1.7 Population<br>Clarification: The population to be studied are patients with spontaneous aneurysmal SAH	V5 31st October 2017
4	Section 2.4. & 11.2.2 Secondary Outcome Endpoints<br>Addition of Proteomic & Genomic blood sampling alongside HP & MDA at (pre dose 0-48 hours), D7 and D28 (All patients)<br><br>Addition of paired SFN / SFN metabolite determination in alternate day paired CSF/Blood samples in EVD patients	V5 31st October 2017

	EVD subset:<br>Baseline Serial paired $CSF_{(EVD)}$/blood SFN/SFN metabolite concentration may be taken at one of the first 3 doses	
4	Section 3.1. Overall Study Design and Plan Description<br>Clarification that there were no dispensing errors in the Per Protocol population.	V5 31st October 2017
4	Section 5. CONCOMITANT MEDICATIONS<br>Removal of statement that no reproductive toxicology have been performed with SFX-01	V5 31st October 2017
4	Section 6.3. Packaging & Storage<br>Clarification on delivery of IMP to sites.<br><br>Update to procedure for randomisation: the next lowest numbered bottle within the specified strata must be selected.	V5 31st October 2017
4	Section 6.4. Labelling<br>Update to labelling such that the strata according to the WFNS grading scale score is included.	V5 31st October 2017
4	Section 6.5.1. Randomization<br>Stratification added to study design:<br><br>"Patients will be allocated to double-blind medication through a stratified randomisation schedule with the strata defined by site and by baseline severity defined by WFNS score of 1-3 or 4 & 5."	V5 31st October 2017
4	7.2. Pre-Dose Assessment (within 48 hours of ictus), 7.4.Post Dose (12-24 hours after first dose), 7.8. Day 7 post ictus (± 1 day), 7.9. Within 2d of discharge, 7.10. Day 28 post ictus (-6/+2 days):<br>Clarification that Safety Bloods are protocol specific	V5 31st October 2017
4	7.3. Dose (within 48 hours of ictus)<br>Clarification that twice daily dosing should occur approximately 12 hours apart	V5 31st October 2017
4	Section 7.6. Ongoing Assessments & 8.5.3. TCD Recordings:<br><br>Clarification that standard of care safety bloods are acceptable for ongoing assessments.<br><br>Clarification that TCD readings (to be performed on alternate days (± 1) post ictus (starting day 3 (± 1) until at least Day 7 (± 1), or discharge whichever is sooner. Any additional TCD readings obtained after this point on clinical grounds will also be recorded).	V5 31st October 2017
4	Section 7.7. Patients with an External Ventricular Drain fitted: Ongoing EVD single sampling (on alternate days +/- 1day from day of EVD fitting until D14 or until the EVD is removed):<br>Addition of blood Proteomic & Genomic analysis<br>Addition Limit of up to Day 14 post ictus for EVD single sampling	V5 31st October 2017

	Addition of SFN analysis of CSF samples	
4	Section 7.8. Day 7 post ictus (± 1 day), 7.10. Day 28 post ictus (-6/+2 days):<br>Blood sample analysis to include HP, MDA *and* Proteomic /Genomic.	V5 31st October 2017
4	Section 8.1. Safety Measurements<br><br>Clarification of safety blood measurements: Biochemistry (Sodium, Potassium, Urea, Creatinine, Glucose, Calcium, Total bilirubin, Alkaline Phosphatase, Alanine Transaminase, Albumin & C-reactive protein), Haematology (Haemoglobin, White Blood Cell count, Neutrophils (absolute), Lymphocytes (absolute) & Platelets), Lipid Profile (LDH, HDL, Triglycerides & Total Cholesterol), Coagulation Status (PT (or INR) & APTT (or APTR) &Fibrinogen)<br><br>Clarification that urine microscopy will be performed in accordance with local procedures.	V5 31st October 2017
4	Section 8.3. PK Measurements<br>Addition of Proteomic /Genomic analysis of blood samples.	V5 31st October 2017
4	25. DATA SAFETY MONITORING BOARD (DSMB)<br>Clarification that The DSMB can meet at any point deemed necessary	V5 31st October 2017
4	Synopsis updated to reflect amendments to protocol	V5 31st October 2017
3	Synopsis<br><br>Typographical correction: Primary Objective, Safety: “To evaluate the safety of up to 28 days of SFX-01 dosed at up to 96 mg Sulforaphane (SFN) per day” amended to “To evaluate the safety of up to 28 days of SFX-01 dosed at up to 92 mg Sulforaphane (SFN) per day”	V4 16th January 2017
3	Synopsis, Schedule of Assessments(footnotes), Section 7.6.Ongoing Assessments<br><br>Ongoing Assessments: Requirement for timing of assessments amended from “alternate days (±1) post ictus (starting day 3 (±1)) until no longer clinically indicated” for all ongoing assessments to “to be performed on alternate days (± 1) post ictus (starting day 3 (± 1) until at least Day 7 (± 1)) and then until no longer clinically indicated. Any additional TCD readings obtained after this point will also be recorded” for ongoing TCD only	V4 16th January 2017
3	Synopsis & Protocol Section 4.4.1. Replacement of Withdrawn Patients<br><br>Addition: Replacement of randomised subjects withdrawn prior to completion of day 7 (post ictus) to be discussed (by the Investigator and Sponsor) and approved	V4 16th January 2017

	by the Investigator and Sponsor's Medical Monitor on a case by case basis	
3	Synopsis, Protocol Section 7.2 Pre-Dose Assessment (within 48 hours of ictus), 8.6.1. Blood Sampling, Schedule of Assessments<br><br>Addition of INR or PT, APTR or APTT, & Fibrinogen (Clauss or Derived) at Pre-Dose Assessment	V4 16th January 2017
3	Synopsis, Protocol Section 2.3 Primary Outcome Endpoints, Section 7 Study Plan, 8.6.1. Blood Sampling & 11.2.1 Statistical parameters and tests /Primary outcome measurements, Schedule of Assessments<br><br>Coagulation tests amended – PT & APTT updated to INR or PT, APTR or APTT, & Fibrinogen (Clauss or Derived)	V4 16th January 2017
3	Synopsis, Protocol Section 2.3 Primary Outcome Endpoints, Section 7 Study Plan, 11.2.1 Statistical parameters and tests /Primary outcome measurements, Schedule of Assessments<br><br>Removal of Urine Dipstick test	V4 16th January 2017
3	Synopsis, Protocol Section 2.3 Primary Outcome Endpoints, Section 7 Study Plan, Section 8.1 Safety Measurements, 8.6.1. Blood Sampling & 11.2.1 Statistical parameters and tests /Primary outcome measurements, Schedule of Assessments<br><br>Removal of lipid tests	V4 16th January 2017
3	Synopsis, Protocol Sections 7.10 Day 28 post ictus & 7.15 Schedule of Assessments<br><br>Day 28 visit window updated to -6/+2 days	V4 16th January 2017
3	Section 9.8 Adverse Reaction to SFX-01 & 9.9 Serious Adverse Events<br><br>Addition of email contact details	V4 16th January 2017
2	Clarification that sub-study patients must have EVD fitted prior to randomisation:<br><br>Synopsis: A sub-study will be conducted in up to 12 patients where an External Ventricular Drain (EVD) fitted.<br>Changed to<br>A sub-study will be conducted in up to 12 patients that already have an External Ventricular Drain (EVD) fitted prior to randomisation;<br><br>A group of up to 12 patients, all of whom have been fitted with an EVD as part of their normal treatment will be selected for a pharmacokinetic sub-study<br>Changed to	V3 18 Mar 2016

	A group of up to 12 patients, all of whom have been fitted with an EVD as part of their normal treatment and prior to randomisation, will be selected for a pharmacokinetic sub-study<br><br>Main protocol 7.13: A group of up to 12 patients, all of whom have been fitted with an EVD as part of normal treatment, will be selected for a pharmacokinetic sub-study<br>Changed to<br>A group of up to 12 patients, all of whom have been fitted with an EVD as part of normal treatment prior to randomisation, will be selected for a pharmacokinetic sub-study<br><br>Main protocol 8.6.4: Twelve patients will participate in a Pharmacokinetic sub-study, all of whom have been fitted with an EVD as part of normal treatment.<br>Changed to<br>Twelve patients will participate in a Pharmacokinetic sub-study, all of whom have been fitted with an EVD as part of normal treatment and prior to randomisation.<br><br>Synopsis and main protocol 7.13: Deleted: If the EVD is fitted after first dose, serial sampling will only take place at day 7.<br>If the EVD is fitted after the first three doses, serial sampling will only take place at day 7.	
	Synopsis and main protocol 4.3: Addition of exclusion criterion 12 - Known hypersensitivity to any component of a sulforaphane containing product including broccoli	V3 18 Mar 2016
	Synopsis and main protocol 25: Clarification to DSMB requirements for meeting.<br><br>Deleted: The DSMB must meet as soon as there have been 2 SAEs that are, at least, possibly linked to the administration of SFX-01<br><br>The DSMB will consider recommending that the study is placed on hold or stopped if the adverse events associated with participation in the study outweigh the potential benefits of the treatment<br>Changed to<br>The DSMB will consider recommending that the study is placed on hold or stopped if the adverse events associated with participation in the study are considered unacceptable.<br><br>The DSMB will review blinded study information which will include<br>Changed to	V3 18 Mar 2016

	The DSMB will review unblinded study information which will include<br><br>Main protocol: Deleted: The DSMB will also be able to request unblinding of patients. The operating procedure will document the planned flow of information in order to describe how the integrity of the study with respect to preventing dissemination of unblinded study information is assured.	
	Remove reference to CTC AE grading throughout and replace with applicable non-CTC grading scheme:<br><br>Synopsis and main protocol 2.3: Change in Common Toxicity Criteria (CTC)<br>Changed to<br>Change in grading of AE severity<br><br>Main protocol 8.1 & 11.2: Escalation in Common Toxicity Criteria (CTC)<br>Change to<br>Escalation in grading of AE severity<br><br>Synopsis and main protocol 25: The DSMB must meet if 2 patients escalate to grade 4 on the Common Toxicity Criteria scale<br>Changed to<br>The DSMB must meet if 2 patients have a grading change in AE severity (from mild/ moderate to severe or life threatening).	V3 18 Mar 2016
	Procedural clarifications	V3 18 Mar 2016
	Administrative changes	V3 18 Mar 2016
1	Protocol Point 11 of exclusion criteria (synopsis and main protocol) (MHRA request) & Main Protocol section 7.9 Within 2d of discharge<br><br>Timeframes for use of contraception – 90 days for men and 30 days for women	V2 27th Jan 2016
	Main Protocol section 1.5 Dose Rationale (MHRA request)<br>"In the pre-clinical studies conducted to date"<br>Changed to<br>"In the clinical studies conducted to date"	V2 27th Jan 2016
	Main Protocol section 7.9 Within 2d of discharge (MHRA request)<br><br>Specifics of what forms of contraception are acceptable	V2 27th Jan 2016
	Synopsis : Pre-Dose Assessment (within 48 hours of ictus) & Main protocol section 7.2 - Clarify procedure:<br>"CT/MRI & Fisher grading"	V2 27th Jan 2016

	Changed to<br>"recording results of CT/MRI & Fisher grading"	
	Synopsis: Between Pre-dose Assessment & Post Dose & Main Protocol section 7.5 - Clarify procedure:<br>"Angiographic assessment (CTA/DSA/MRA) "<br>Changed to<br>"Recording of results and form of angiographic assessment (CTA/DSA/MRA) "	V2 27th Jan 2016
	Main protocol 7.15 Schedule of Assessments - Typographical Error<br>X added against Safety Urine testing at Pre-dose assessment. (Previously in text but missing from Schedule of Assessments)	V2 27th Jan 2016

Protocol Approval Page

This protocol has been read and approved by:

**Sponsor representative:**	
**Signature:**	[illegible]
**Date:**	18/5/18

**Chief Investigator:**	DIEDERIK BULTERS
**Signature:**	[illegible]
**Date:**	17/5/18

**The Clinical Trial Company:**	J. BOOTH.
**Signature:**	[illegible]
**Date:**	17/5/18

**Statistician:**	
**Signature:**	[illegible]
**Date:**	18 May 2018

INVESTIGATOR PROTOCOL APPROVAL PAGE

SFX-01 AFTER SUBARACHNOID HAEMORRHAGE<br><br>(SAS STUDY)

I, the undersigned, have read and understood the protocol and am aware of my responsibilities as an investigator. I agree to conduct the study in accordance with this protocol and any subsequent amendments, the Declaration of Helsinki, ICH GCP guidelines, and the laws and regulations of the country in which the study is being conducted.

**Investigator Name:**<br><br>**Investigator Title:**<br><br>**Investigator Address:**	

**Investigator Signature:**<br><br>**Date:**	

Protocol Synopsis

TITLE:

SFX-01 After Subarachnoid Haemorrhage (SAS)

PROTOCOL NO:

EVG001SAH

STUDY PHASE:

Phase II

INVESTIGATOR STUDY SITES:

This study will be conducted at multiple study sites located in the United Kingdom

OBJECTIVES:

Primary Objectives:

Safety
To evaluate the safety of up to 28 days of SFX-01 dosed at up to 92 mg Sulforaphane (SFN) per day.

Pharmacokinetic
To detect the presence of SFN in Cerebrospinal Fluid (CSF)

Efficacy
To determine if a minimum of 7 days treatment with SFX-01 reduces Middle Cerebral Artery (MCA) peak flow velocity following Subarachnoid Haemorrhage (SAH).

Secondary Objectives:

- To determine if a minimum of 7 days treatment with SFX-01 improves clinical outcome following SAH as measured using the modified Rankin Scale assessed at 7 days, discharge, 28, 90 and 180 days post ictus.
- To determine blood SFN levels (and its metabolites) with treatment with SFX-01 (300mg bid).
- To determine CSF SFN levels and kinetics with treatment with SFX-01 (300mg bid).
- To determine if up to 28 days treatment with SFX-01 increases blood haptoglobin (HP) levels and decreases malondialdehyde (MDA) levels following SAH.
- To determine if up to 28 days treatment with SFX-01 can reduce the incidence of Delayed Cerebral Ischaemia (DCI) following SAH.
- To determine if up to 28 days treatment with SFX-01 improves long-term outcome in subjects following SAH.
- To determine if up to 28 days of treatment with SFX-01 can reduce iron deposition and cortical atrophy following SAH.

METHODOLOGY:

This is a Safety, Tolerability, Pharmacokinetic and Pharmacodynamic Study of SFX-01 in Subarachnoid Haemorrhage, with exploratory evaluations of efficacy.

The study is a randomised, double-blind, parallel-group design comparing SFX-01 (300 mg) taken orally as capsules or as a suspension via a nasogastric tube (NG) twice-daily for up to 28 days versus placebo in up to 120 patients to achieve 90 patients in the per-protocol analysis set, who have had SAH and present within 48 hours of ictus.

The treatment group will receive SFX-01 in order to improve outcome and reduce the long-term complications of SAH such as Delayed Cerebral Ischaemia, as reflected by Trans-Cranial Doppler (TCD) readings. The objective is to demonstrate safety and search for signals of efficacy in patients that have had SAH.

A sub-study will be conducted in up to 12 patients that already have an External Ventricular Drain (EVD) fitted prior to randomisation; serial CSF samples will be taken pre- & post-dose on two occasions to determine pharmacokinetics of Sulforaphane in CSF in comparison with plasma pharmacokinetics. Sub-study patients will undergo all other procedures (with the exception of lumbar puncture).

Treatment duration is up to 28 days; follow up duration is 28 days, three and six months. The planned trial period is 24 months.

The Per Protocol Population (for Primary analysis) will be considered to be those patients that have been dosed for a minimum of 7 days.

A data safety monitoring board (DSMB) will be set-up to monitor safety throughout the trial period and provide recommendations for any necessary actions. A steering committee (comprising the Chief Investigator and the sponsor’s Chief Medical Officer) will receive and review the reports from the DSMB, and take action as appropriate.

NUMBER OF PATIENTS:

Up to 120 patients may be recruited and enrolled into the trial in order to provide 90 who will meet the per protocol criteria and be analysed for the efficacy analyses.

In the instance where patients have been entered into the trial prior to informed consent being obtained (i.e. through the emergency consent procedure) and consent is subsequently refused or not obtained within 24 hours by the patient and/or legal representative the participants shall be withdrawn and replaced.

Replacement of patients who withdraw/are withdrawn prior to completion of day 7 (post ictus) is to be discussed (by the Investigator and Sponsor) and approved by the Investigator and Sponsor’s Medical Monitor on a case by case basis.

Patients who withdraw for any other reason after randomisation will not be replaced.

INCLUSION/EXCLUSION CRITERIA:

Inclusion criteria

1. Patients with radiological evidence of spontaneous aneurysmal SAH
2. Fisher grade 3 or 4 on CT
3. Definitive treatment of aneurysm has not been ruled out
4. Previously living independently

5. In the opinion of the investigator, the delay from ictus to randomisation and initiation of trial medication will not exceed 48 hours
6. Aged 18 to 80 years
7. In the opinion of the investigator it will be possible to obtain Informed Consent from the Patient, Personal Legal Representative or Professional Legal representative within 24 hours of first dose

Exclusion criteria

1 Traumatic SAH
2 Fisher grade 1 or 2
3 SAH diagnosed on lumbar puncture with no evidence of blood on CT
4 Decision not to treat aneurysm has been made
5 Plan to withdraw treatment
6 Significant kidney disease as defined as plasma creatinine ≥2.5mg/dL (221 µmol/l)
7 Liver disease as defined as total bilirubin ≥2-fold the upper limit of normal; as measured by the local laboratory
8 Females who are pregnant or lactating.
9 Participants enrolled in another interventional research trial in the last 30 days
10 Patients for whom it is known, at the time of screening, that clinical follow-up will not be feasible
11 Patients unwilling to use two forms of contraception (one of which being a barrier method see section 7.9) 90 days for men and 30 days for women after last IMP dose
12 Known hypersensitivity to any component of a sulforaphane containing product including broccoli

DOSE/ROUTE/REGIMEN:

Active: SFX-01 (active 300 mg capsule taken orally twice-daily for up to 28 days)

Placebo: SFX-01 placebo (placebo 300 mg capsule taken orally twice-daily for up to 28 days)

For patients unable to take tablets orally but have a nasogastric tube in situ, the study drug will be administered per tube.

Patients will be randomised to double blind active or placebo Investigational Medicinal Product and will be stratified using the most recent WFNS grading score, post ictus and prior to randomization. The two strata will comprise WFNS scores 1-3 and WFNS scores 4-5.

REFERENCE TREATMENT:

Placebo capsules identical in appearance to SFX-01 active capsules.

CRITERIA FOR EVALUATION:

Primary Outcome Variables

Safety

- Concomitant medication
- Adverse events
- Change in grading of AE severity
- FBC, U&Es, LFT, CRP & Urine Microscopy

- INR or PT, APTR or APTT, & Fibrinogen (Clauss or Derived) at 7 & 28 days

Pharmacokinetic

- Presence of SFN in CSF

Efficacy

- The maximum MCA flow velocity determined using TCD.
  Treatment groups will be compared using a t-test.

Secondary Outcome Variables

- Incidence of Delayed Cerebral Ischaemia (DCI) defined as a new focal deficit or reduction in Glasgow Coma Scale (GCS) ≥2 if not explained by other causes (i.e re-bleed, hydrocephalus, seizure, meningitis, sepsis or hyponatraemia)
  Treatment groups will be compared using a chi-square test.
- Incidence of new cerebral infarct on Computed Tomography (CT) or Magnetic Resonance Imaging (MRI)
  Treatment groups will be compared using a chi-square test.
- Institution of hypertensive (triple H) therapy for presumed DCI
  Treatment groups will be compared using a chi-square test.
- Modified Rankin Scale (mRS), at 7 days, discharge, 28, 90 and 180 days.
  Treatment groups will be compared using a van Elteren test.
- SF-36 quality of life survey at 28, 90 & 180 days.
  Treatment groups will be compared using a t-test.
- Checklist for Cognitive and Emotional Consequences (CLCE-24), Brain Injury Community Rehabilitation Outcomes Scale (BICRO-39), 90 & 180 days.
  Treatment groups will be compared using a van Elteren test.
- Subarachnoid Haemorrhage Outcome Tool (SAHOT) and Glasgow Outcome Scale – Extended (GOSE) at 28, 90 & 180 days.
  Treatment groups will be compared using a van Elteren test.
- Length of acute hospital stay
  Treatment groups will be compared using a Wilcoxon-Mann-Whitney-test.
- Discharge location (e.g. home, rehabilitation centre etc.)
  Treatment groups will be compared using a chi-square test.
- Amount of iron identified on MRI Susceptibility Weighted Imaging (SWI) 180 days after start of treatment.
  Treatment groups will be compared using a t-test.
- Cortical atrophy on T1 MRI at 180 days after start of treatment
  Voxel-based morphometry will be used to identify and quantify regional areas of atrophy

Non-EVD Patients: i.e. patients will have a Lumbar Puncture for collection of CSF

- Blood HP and MDA, Proteomic & Genomic concentration at baseline (pre dose 0-48 hours), D7 and D28.
- Paired $CSF_{(Lumbar\ Puncture)}$/blood HP, MDA, Proteomic & Genomic & SFN/SFN metabolite concentrations at Day 7

EVD Patients: (i.e. will not have a lumbar puncture)

- Blood HP and MDA, Proteomic & Genomic concentration at baseline (pre dose 0-48 hours), D7 and D28.

- Paired $CSF_{(EVD)}$/blood HP & MDA, Proteomic & Genomic concentration on alternate days (+/- 1 day) starting on day of EVD fitting until D14 or the EVD removal.
- Paired $CSF_{(EVD)}$/blood HP, MDA, Proteomic & Genomic & SFN/SFN metabolite concentration at day 7

Subset of 12 EVD Patients: In addition to all other sampling the following samples will be taken:

- Serial paired $CSF_{(EVD)}$/blood SFN/SFN metabolite concentrations at one of the first 3 doses and day 7

Measured PK-variables will be log-transformed, if necessary, and descriptively displayed using Box-plots.

STATISTICAL METHODS:

Primary analysis will be carried out using data from the Per-Protocol Population, i.e. those patients that have been dosed to day 7 post ictus.

Exact definition of major protocol deviations will be discussed by the clinical team case by case during the blind review of the data and described in the blind review document.

Protocol violations will be considered for each protocol period separately.

All data will be presented in patient data listings. For continuous variables, descriptive statistics (n, mean, median, standard deviation, minimum, and maximum) will be presented. For categorical variables, frequencies and percentages will be presented. Graphical displays will be presented as appropriate.

Power and Sample Size:

Ninety patients will give 80% power to detect a difference in maximum MCA flow velocity which is approximately half of the standard deviation of the mean value. The standard deviation is assumed to be approximately 30% of the mean value based on historical data.

Efficacy:

The primary efficacy variable will be compared between the treatment groups using a t-test for maximum MCA flow velocity.

Categorical secondary endpoints will be compared using chi-square-tests.

Pharmacokinetics:

Measured PK-variables will be log-transformed, if necessary, and descriptively displayed using Box-plots.

STUDY SCHEDULE:

Continuous Assessment: Adverse Events, Concomitant Medication & Medication Compliance

Pre-Dose Assessment (within 48 hours of ictus): Informed Consent (within 24 hours of first dose), Inclusion/Exclusion (screen failures to be recorded), recording results of CT/MRI & Fisher grading, Demographics/Medical History, Physical Examination, Pregnancy test, Concomitant Medication, protocol specific Safety Bloods (Biochemistry, Haematology, Lipid Profile & Coagulation Status) and Urine, blood HP, MDA,

Proteomic/Genomic sampling and recording of World Federation of Neurological Societies (WFNS) scale after resuscitation (post ictus and prior to randomisation). WFNS score will be used to allocate stratified randomised medication.

Dose (within 48 hours of ictus): Randomisation & administration of the first dosage (within 48 hours of ictus, twice daily for 28 days)

Post Dose (12-24 hours after first dose): protocol specific Safety Bloods

Between Pre-dose Assessment & Post Dose: Recording of results and form of angiographic assessment (CTA/DSA/MRA) and first TCD reading (to measure peak MCA flow)

Ongoing Assessments – All patients: Safety Bloods as part of normal clinical care, TCD readings (to be performed on alternate days (± 1) post ictus (starting day 3 (± 1) until at least Day 7 (± 1), or discharge whichever is sooner. Any additional TCD readings obtained after this point will also be recorded) and Glasgow Coma Score

Patients with an EVD fitted: Ongoing EVD single sampling (on alternate days (+/- 1 day) from day of EVD fitting until the EVD is removed or up to 14 days): A single EVD sample is to be taken, paired with a single blood sample for determination of CSF/blood HP, MDA, Proteomic & Genomic levels

Day 7 post ictus (± 1 day): Protocol specific Safety Bloods and Urine, TCD reading, modified Rankin Score and Concomitant Medication.

- Non-EVD patients only: Paired blood sampling and lumbar puncture CSF sample to determine CSF/blood HP, MDA, Proteomic/Genomic & SFN/SFN metabolite concentrations
- EVD patients only: Paired blood and EVD CSF sampling to determine CSF/blood HP, MDA, Proteomic/Genomic & SFN/SFN metabolite concentrations

Discharge (-2 days): Protocol specific Safety Bloods and Urine, modified Rankin Score, Glasgow Coma Score and Concomitant Medication.
Note: Discharge is defined as when the consultant for the intervention of treating the SAH decides that their specialist care is no longer needed.

Day 28 post ictus (-6/+2 days): Modified Rankin Score, Short Form 36 Health Survey, Subarachnoid Haemorrhage Outcome Tool, Glasgow Outcome Scale (Extended) and Concomitant Medication. Protocol specific Safety Bloods and Urine, HP, MDA, Proteomic/Genomic.

Day 90 post ictus (± 14): Modified Rankin Score, Short Form 36 Health Survey, Checklist for Cognitive and Emotional Consequences, Brain Injury Community Rehabilitation Outcomes Scale, Subarachnoid Haemorrhage Outcome Tool, Glasgow Outcome Scale (Extended) and Concomitant Medication.

Day 180 post ictus (± 28): Modified Rankin Score, Short Form 36 Health Survey, Checklist for Cognitive and Emotional Consequences, Brain Injury Community Rehabilitation Outcomes Scale, Subarachnoid Haemorrhage Outcome Tool, Glasgow Outcome Scale (Extended) and Concomitant Medication. An MRI will be performed within 60 days of the Day 180 visit

Pharmacokinetic sub-study
A group of up to 12 patients, all of whom have been fitted with an EVD as part of their normal treatment and prior to randomisation, will be selected for a pharmacokinetic sub-study.

Additional patient consent will be required for the sub-study; in cases where patients lack capacity the consent of a Personal Legal Representative will be sought before any sub-study procedures are carried out.

The patients will, in addition to all other protocol required procedures of a patient with an EVD fitted, undergo serial paired blood/CSF sampling (1 sample pre dose and hourly ± 5 minutes for 6 hours post dose) after one of the first three doses and on day 7 after the morning dose.

SAFETY

Recruitment:
The DSMB will convene after 20 patients have been dosed to day 7 post ictus (with adequate safety assessment data) as in-patients in tertiary care for a formal safety review.

The safety review shall make a decision on the acceptability of discharging patients from tertiary care with SFX-01 to complete the dosing course to day 28.

Data Safety Monitoring Board

The DSMB will convene under the following circumstances:

- The DSMB must meet once the 20th patient has been dosed to day 7 post ictus
- The DSMB must meet as soon as there has been a SUSAR
- The DSMB must meet if 2 patients have a grading change in AE severity (from mild/ moderate to severe or life threatening).

Study Stopping Rules

The clinical investigation can be placed on hold / stopped early for two reasons and will be based on clinical judgement:

- The DSMB will consider recommending that the study is placed on hold or stopped if the adverse events associated with participation in the study are considered unacceptable.
- The DSMB will consider recommending that the study is placed on hold or stopped if the adverse events associated with SFX-01, in their opinion, significantly outnumber (in frequency or intensity) the adverse events associated with the normal standard of care.
- At any point deemed necessary

Contact Names and Addresses

**Sponsor**:	Evgen Pharma plc
Registered Address:	Liverpool Science Park IC2<br>146 Brownlow Hill<br>Liverpool L3 5RF
Contact details:	Tel: +44(0) 151 705 3532<br>Fax: +44(0) 151 705 3534
Head Office	The Colony<br>Altrincham Road<br>Wilmslow<br>Cheshire SK9 4LY
Contact details:	+44 (0) 1625 466591
Personnel:	Dr Thomas Morris - Medical Monitor
**Chief Investigator**	Mr. Diederik Bulters MBChB and intercalated BSc (pharm), Consultant Neurosurgeon and Honorary Senior Clinical Lecturer
Address:	University Hospital Southampton, Tremona Road, Southampton, Hampshire, SO16 6YD
	Tel: +44(0) 2381 205 311
	Fax: +44(0) 2381 204148
**CRO**:	The Clinical Trial Company Ltd
Address:	Mere View Barn, Park Lane, Pickmere, Knutsford, Cheshire, WA16 OLG
Contact details:	Tel: +44(0) 1565 733 772
	Fax: +44(0) 1565 732 958
Personnel:	Ewan Campbell – Director
	Ingrid Gerber – Project Manager
**Statistician**	Data Magik Limited
Address:	Laburnum House, East Grimstead, Salisbury, Wiltshire, SP5 3RT
Personnel:	David Fleet
Telephone	+44 (0)1722 712 972
**Pharmacovigilance**	Diamond Pharma Services Ltd
Address:	Suite 2, Ground Floor, Field House, Station Approach, Harlow, Essex, CM20 2FB
Telephone	+44 (0) 203 911 9410
Fax	+44 (0)1279 418964

Abbreviations

AE	Adverse Event
ADL	Activities of Daily Living
APTR	Activated Partial Thromboplastin Time Ratio
APTT	Activated Partial Thromboplastin Time
AUC	Area Under the Curve
bid	Two times daily
BICRO	Brain Injury Community Rehabilitation Outcomes Scale
CFR	Code of Federal Regulations
CI	Chief Investigator
CLCE	Checklist for Cognitive and Emotional Consequences (CLCE-24)
eCRF	Electronic Case Report Form
CRP	C-Reactive Protein
CSF	Cerebrospinal Fluid
CT	Computed Tomography
CTA	CT Angiography
DCI	Delayed Cerebral Ischaemia
DSA	Digital Subtraction Angiography
DSMB	Data Safety Monitoring Board
EVD	External Ventricular Drain
FBC	Full Blood Count
FDA	Food and Drug Administration (United States)
GCP	Good Clinical Practice
GCS	Glasgow Coma Scale
GI	Gastro-Intestinal
GOSE	Glasgow Outcome Scale (Extended)
GP	General Practitioner
HO-1	Haeme Oxygenase-1
HP	Haptoglobin
HPMC	Hydroxypropyl Methycellulose
IB	Investigator Brochure
ICH	International Conference on Harmonisation
IMP	Investigational Medicinal Product
INR	International Normalised Ratio
LFT	Liver Function Test(s)
MCA	Middle Cerebral Artery
MDA	Malondialdehyde
MRA	Magnetic Resonance Angiography
MRI	Magnetic Resonance Imaging
mRS	Modified Rankin Scale

Nrf2	Nuclear factor erythroid 2-related factor 2
NG	NasoGastric
NICU	NeuroIntensive Care Unit
NOAEL	No-observed-adverse-effect level
NQO1	NAD(P)H:quinone oxidoreductase-1
PK	Pharmacokinetic
PT	Prothrombin Time
REC	Research Ethics Committee
SAE	Serious Adverse Event
SAH	Subarachnoid Haemorrhage
SAHOT	Subarachnoid Haemorrhage Outcome Tool
SF-36	Short Form (36) Health Survey
SFN	Sulforaphane
SFX-01	The Investigational Medicinal Product/stabilised Sulforaphane
SUSAR	Suspected Unexpected Serious Adverse Reaction
SWI	Susceptibility Weighted Imaging
TCD	Trans-Cranial Doppler
TCTC	The Clinical Trial Company Ltd
TMF	Trial Master File
TSF	Trial Site File
U&Es	Urea & Electrolytes
WFNS	World Federation of Neurosurgical Societies Scale

Table of Contents

1. BACKGROUND INFORMATION AND STUDY RATIONALE 27

1.1. Investigational Agent 27

1.2. Preclinical Data 27

1.2.1. Nrf2 pathway 28

1.2.2. Sulforaphane (SFN) 28

1.3. Risks / Benefits 30

1.4. Study Rationale 31

1.5. Dose Rationale 31

1.6. Trial Conduct 32

1.7. Population 32

2. STUDY OBJECTIVES AND ENDPOINTS 32

2.1. Primary Objective 32

2.2. Secondary Objectives 32

2.3. Primary Outcome Endpoints 33

2.4. Secondary Outcome Endpoints 33

3. STUDY DESIGN 34

3.1. Overall Study Design and Plan Description 34

3.2. Duration of Treatment 35

3.3. Premature Discontinuation of Treatment 35

3.3.1. Cessation of Treatment 35

3.3.2. Withdrawal from Trial 36

3.3.3. Replacements 36

3.4. Discussion of Study Design, Including the Choice of Control Groups 36

4. SUBJECT SELECTION CRITERIA 36

4.1. Subject Recruitment 36

4.2. Inclusion Criteria 37

4.3. Exclusion Criteria 37

4.4. Subject Withdrawals 37

4.4.1. Replacement of Withdrawn Patients 38

5. CONCOMITANT MEDICATIONS 38

5.1. Permitted Concomitant Medications 38

5.2. Non-Permitted Concomitant Medications 38

6. TREATMENTS 38

6.1. Appearance and Content 38

6.2. Dosage and Administration 39

6.2.1. Trial Drug 39

6.2.2. Placebo 39

6.2.3. Administration 39

6.2.4. Dose Frequency De-escalation 39

6.3. Packaging & Storage 39

6.4. Labelling 40

6.5. Blinding and Randomisation 40

6.5.1. Randomization 40

6.5.2. Blinding 40

6.6. Drug Storage 41

6.7. Drug Accountability 42

7. STUDY PLAN 42

7.1. Continuous Assessments: 42

7.2. Pre-Dose Assessment (within 48 hours of ictus): 42

7.3. Dose (within 48 hours of ictus) 43

7.4. Post Dose (12-24 hours after first dose) 43

7.5. Between Pre-dose Assessment & Post Dose: 43

7.6. Ongoing Assessments – All patients: 43

7.7. Patients with an External Ventricular Drain fitted: Ongoing EVD single sampling (on alternate days +/- 1day from day of EVD fitting until D14 or until the EVD is removed): 44

7.8. Day 7 post ictus (± 1 day): 44

7.9. Within 2d of discharge: 44

7.10. Day 28 post ictus (-6/+2 days): 46

7.11. Day 90 post ictus (± 14): 46

7.12. Day 180 post ictus (± 28): 47

7.13. Pharmacokinetic sub-study 47

7.14. Early Termination Visit 47

7.15. Schedule of Assessments 48

8. STUDY PROCEDURES / EVALUATIONS 50

8.1. Safety Measurements 50

8.2. Efficacy Measurements 50

8.3. PK Measurements 51

8.4. Definitions of Assessments 51

8.4.1. Fisher Grade 51

8.4.2. World Federation of Neurological Societies Grading System For Subarachnoid Hemorrhage (WFNS) 52

8.4.3. Modified Rankin Scale (mRS) 52

8.4.4. Short Form Health Survey (SF-36) 52

8.4.5. Checklist for Cognitive and Emotional Consequences (CLCE-24) 52

8.4.6. Brain Injury Community Rehabilitation Outcomes Scale (BICRO-39) 52

8.4.7. Subarachnoid Haemorrhage Outcome Tool (SAHOT) 52

8.4.8. Glasgow Outcome Scale Extended (GOSE) 53

8.4.9. Glasgow Coma Score (GCS) 53

8.5. Clinical Evaluations 53

8.5.1. Medical History 53

8.5.2. Radiology 53

8.5.3. TCD Recordings 53

8.5.4. MRI 53

8.6. Clinical Laboratory Evaluations 54

8.6.1. Blood Sampling 54

8.6.2. Pregnancy Testing 54

8.6.3. Lumbar Puncture 54

8.6.4. EVD Sampling 55

8.6.5. Non-Standard Assays or Procedures 55

9. ADVERSE EVENT REPORTING 56

9.1. Definitions 56

9.2. Expectedness 56

9.3. Intensity of Adverse Event 57

9.4. Causality Assessment 57

9.5. Action Taken Regarding the Study Drug 58

9.6. Outcome 58

9.7. Recording Adverse Events 58

9.8. Adverse Reaction to SFX-01 58

9.9. Serious Adverse Events 58

10. DATA MANAGEMENT 59

10.1. Trial Documentation and Trial Confidentiality 60

10.1.1. Trial Documentation, eCRFs and Document Keeping 60

10.1.2. Confidentiality of Trial Documents and Patient Records 61

11. STATISTICAL ANALYSIS PLAN 61

11.1. Sample Size 61

11.1 Populations for analysis 61

11.2. Statistical parameters and tests 62

11.2.1. Primary outcome 62

11.2.2. Secondary outcomes 62

12. ETHICS COMMITTEE / IRB APPROVAL 63

13. REGULATORY REQUIREMENTS 63

14. INFORMED CONSENT 64

15. DIRECT ACCESS TO SOURCE DOCUMENTATION / DATA 66

16. STUDY MONITORING 66

17. QUALITY ASSURANCE 66

18. TRIAL SCHEDULE 66

19. INSURANCE 66

20. CONFIDENTIALITY 67

21. DATA PROTECTION 67

22. PREMATURE TERMINATION OF THE STUDY 67

23. RECORD RETENTION 67

24. PUBLICATION OF RESULTS 67

25. DATA SAFETY MONITORING BOARD (DSMB) 67

26. STEERING COMMITTEE 69

27. REFERENCES 69

28. APPENDICES 72

28.1. Appendix 1 – Declaration Of Helsinki - 1996 72

1. BACKGROUND INFORMATION AND STUDY RATIONALE

Spontaneous Subarachnoid Haemorrhage (SAH) is a complex cerebrovascular disease with an incidence of 8-10 per 100,000 population affecting more than 7000 patients within the UK annually. Around 85% of cases are due to ruptured intracranial aneurysms. Perimesencephalic non-aneurysmal SAH accounts for 10% of spontaneous SAH [1]. The incidence of SAH is age-related with higher incidence amongst age group 40-60 years and a peak incidence at the age of 55. SAH carries a high overall mortality rate of up to 67% [2] and only half of the survivors are able to live independently. Given the age-related incidence and high morbidity and mortality, SAH has a high burden on society due to the loss of productivity and resources used [3].

Conventionally following SAH, treatment is primarily directed to securing the aneurysm to prevent further re-bleed. This however does nothing to ameliorate the morbidity and mortality due to the haemorrhage. The only approved effective medicine to reduce morbidity is nimodipine [4]. However, its effects are small and despite its use poor outcomes remain a significant problem as evidenced by contemporary outcome data since its introduction [5]. Moreover, even in survivors conventionally considered to have made a good recovery, neurocognitive deficits are common leading to extensive problems with social reintegration and functioning in the workplace [6].

The mechanisms underlying poor outcomes are multifactorial. A significant component is due to secondary injury from inflammation [7], spreading depolarisation [8], macroscopic cerebral vasospasm and microcirculatory disturbance [9]. The common factor in all these mechanisms is that they are initiated by extracellular haemoglobin released as red blood cells in the clot lyse. This results in direct neurotoxicity and increased oxidative stress and further injury [10]. Thus any treatment to ameliorate their effects would be best targeted at reducing the cell free haemoglobin, oxidative stress and inflammation.

Sulforaphane (SFN) is known to up-regulate the nuclear factor erythroid 2-related factor 2 (Nrf2) pathway; Nrf2 is a promoter of haptoglobin (HP) expression and a wide range of anti-oxidant and anti-inflammatory enzymes including Haeme Oxygenase-1 (HO-1) and NAD(P)H:quinone oxidoreductase-1 (NQO1). These effects have been shown to reduce inflammation and neurological deficits seen in rats after intracerebral haemorrhage and SAH [11, 12].

As SFN is an unstable molecule it cannot practically be employed in clinical use. However when complexed with cyclodextrin to form SFX-01 it is stable and can be practically used in the clinical setting. On ingestion, SFN is released from the cyclodextrin and thus SFX-01 is an effective method to deliver SFN.

1.1. Investigational Agent

SFX-01 (300 mg) taken orally or via nasogastric tube twice-daily

1.2. Preclinical Data

There is limited clinical experience with SFX-01 with two Phase I trials completed to date (EVG001/N & EVG002/N). However, there are numerous trials that have used makeshift SFN preparations derived from broccoli extracts.

1.2.1. Nrf2 pathway

Nuclear factor erythroid 2-related factor 2 (Nrf2) is a redox sensitive transcription factor known as a protector for many organs, including the brain. Nrf2 is sequestered in the cytoplasm by kelch-like ECH-associated protein 1 (Keap1) under physiological conditions. It transactivates the expression of a group of anti-oxidant and cytoprotective enzymes, such as HO-1, NQO1 and glutathione S-transferase-a1 [13]. In response to oxidative stress it translocates into the nucleus and binds to the antioxidant-response element. This coupling promotes transcription of protective genes encoding antioxidant and detoxifying enzymes [14]. The full range Nrf2-regulated genes has been documented by induction with sulforaphane [15].

Indeed Nrf2 seems a global neuroprotectant; it has been demonstrated to have a key role in intracerebral haemorrhage and cerebral ischaemia, both of which pathophysiologically overlap with SAH.

A previous study utilising a mouse intracerebral haemorrhage model showed that Nrf2 deficient mice were significantly more prone to haemorrhagic brain injury and neurologic deficits. Nrf2 reduces intracerebral haemorrhage induced early brain injury, possibly by providing protection against leukocyte-mediated free radical oxidative damage [12].

Previous studies have shown that Nrf2 upregulation also provides protection from cerebral ischemia *in vivo* [16, 17]. While clinically it is difficult to capitalise on this as it requires pre conditioning at a time when a stroke may not be expected. In SAH, however, ischaemia has a delayed onset and thus there is an opportunity for augmenting Nrf2 expression prior to DCI.

Critically Nrf2 is a key regulator in reducing oxidative stress, inflammatory damage and accumulation of toxic metabolites involved as part of the underlying process in SAH. This has initially been investigated *in vitro* in [11], and more latterly *in vivo* in mice [18, 19]. In this study the absence of Nrf2 function resulted in exacerbated brain injury with increased brain oedema, blood–brain barrier disruption, neural apoptosis, and severe neurological deficits at 24 hr after SAH. Cerebral vasospasm was severe at 24 hr after SAH, but not significantly different between wild type and Nrf2 knock-out mice after SAH. Meanwhile, Malondialdehyde (MDA), TNF-α and IL-1β were increased and GSH/GSSG ratio was decreased in Nrf2 KO mice after SAH.

1.2.2. Sulforaphane (SFN)

Sulforaphane (SFN) is a well-studied isothiocyanate and potent inducer of Nrf2 signalling. It is formed on ingestion of cruciferous vegetables, particularly broccoli and broccoli sprouts. These contain glucoraphanin which is hydrolysed to sulforaphane by myrosinase, (present in the plant as well as the gut microflora). Sulforaphane is known to cross the blood brain barrier in animal models [20]. Experience in humans with sulforaphane from broccoli and broccoli sprouts is extensive with limited safety data. In a placebo-controlled, double-blind, randomized clinical trial of glucosinolates (principally glucoraphanin) as well as isothiocyanates (principally sulforaphane) no significant or consistent subjective or objective abnormal events [21] were reported. Most of the experience has been at lower doses than in the current proposed study.

Sulforaphane has been tested in animal models of ischaemic stroke, intracerebral haemorrhage and subarachnoid haemorrhage. In a rat ischaemic stroke model, SFN was shown to reduce infarct volume following temporary occlusion of left common carotid artery or middle cerebral artery. Those in the treatment group were injected with intraperitoneal SFN 15 minutes after the onset of ischaemia. SFN was found to increase brain Haeme Oxygenase-1 (HO-1) mRNA levels, an enzyme involved in reducing oxidative stress. Oxidative stress is thought to be a

contributing factor in the pathogenesis of ischaemic stroke and inflammation. The overall infarct volume was significantly reduced in the SFN-treated group by as much as 30% [22]. This is particularly relevant to SAH where in addition to territorial ischaemia from large vessel vasospasm, there is increasing recognition of the effect of microcirculatory disturbances and infarction. This was first demonstrated in a SAH post mortem study which showed small cortical and hypothalamic infarcts in most subjects [23]. These insults are too small to discriminate by computed tomography (CT) and thus under-recognised in clinical practice.

Disruption of the blood-brain barrier and cerebral oedema are the major pathogenic mechanisms leading to neurological dysfunction and death after ischaemic stroke. In a study [24] where rats were preconditioned with SFN prior to ischaemic stroke (similar to the model described above) Nrf2 and HO-1 protein expression was shown to be upregulated in cerebral microvessels of peri-infarct regions as well as cerebral endothelium in the infarct core. In animals pre-treated with SFN there was marked reduction of lesion progression, blood-brain barrier disruption and neurological dysfunction. Delayed Cerebral Ischaemia (DCI) as a complication of SAH presents a few days after the onset of symptoms (between days 3-14). Therefore, preconditioning with SFN is a reasonable and feasible method of preventing this phenomenon in those with SAH even if it is not prior to conventional ischaemic stroke.

Oxidative and cytotoxic damage play an important role in the pathogenesis of intracerebral haemorrhage. In one study rats and Nrf2-deficient or control mice received intrastriatal injection of autologous blood to mimic intracerebral haemorrhage. The treatment group was injected with intraperitoneal SFN; activation of Nrf2 with SFN resulted in upregulation of haptoglobin (central to the haptoglobin-CD163 haemoglobin scavenging system active in human SAH [25]) as well as a range of antioxidative and detoxifying enzymes. There was a resultant reduction in oxidative damage and inflammation in brain areas endangered by the intraparenchymal hematoma. Neutrophil count, oxidative damage, and behavioural deficit were shown to be reduced in intracerebral haemorrhage-affected brain tissue of the SFN treatment group.

Furthermore, Nrf2-deficient mice demonstrated more severe neurologic deficits after intracerebral haemorrhage and did not benefit from the protective effect of SFN. Therefore, activation of Nrf2 with SFN after intracerebral haemorrhage may represent a potential target for combating associated damage [16] or conditions with similar pathogenesis such as SAH.

The protective role of SFN in intracerebral haemorrhage was replicated in a further study investigating the role of haptoglobin after SAH. Haptoglobin is a plasma protein that binds cell free haemoglobin with high affinity. This has been demonstrated to be active in human SAH. In rodents SFN upregulated haptoglobin expression and alleviated intracerebral haemorrhage mediated brain injury [26].

SAH makes a better target for SFN treatment given blood is distributed over the entire surface of cortex resulting in a more severe inflammatory response. This is supported by the observation that there is a higher rate of late secondary deterioration and poor outcome after SAH than in intracerebral haemorrhage. The effect of SFN in SAH has been investigated *in vitro* using vascular smooth muscle cultures from rat aorta exposed to oxyhaemoglobin as a model for SAH. In this model Nrf2 is up-regulated; the effect is increased in the SFN group compared to the control group, whereas the increase in the inflammatory cytokines (IL-1b, IL-6 and TNF-α) observed 48 h after oxyhaemoglobin treatment, is markedly reduced by SFN [11].

The effect of SFN was also investigated in an *in vivo* rat SAH model with intraperitoneal SFN injections 30m, 12 h and 36h after SAH. 0.3 ml fresh arterial, nonheparinized blood into the

prechiasmatic cistern was injected over 20 sec. As a result, Nrf2 and its target gene product, haeme oxygenase-1 (HO-1), were upregulated in the cortex after SAH and peaked at 24 hr post-SAH. After intraperitoneal SFN administration, the elevated expression of Nrf2-antioxidant response element related factors such as Nrf2, HO-1, NAD(P)H:quinone oxidoreductase 1 (NQO1), and glutathione S-transfer- ase-a1 (GST-a1) were detected in the cortex at 48 hr following blood injection. In the SFN treated group, early brain damage such as brain oedema, blood–brain barrier impairment, cortical apoptosis, and motor deficits were significantly ameliorated compared with vehicle- treated SAH rats [19].

1.3. Risks / Benefits

The potential therapeutic benefits of SFX-01 appear to outweigh any potential risks for patients with SAH who may receive it with the intent of reducing DCI. SFN has been demonstrated to be a potent activator of cellular oxidative stress defence mechanisms via activation of Nrf2 and to initiate over-expression of HO-1 and NQO1, both enzymes responsible for maintenance of cellular oxidative balance. This pathway has been demonstrated in the literature to be of critical importance in neuroprotection. These protective effects have been demonstrated with the active component of SFX-01, SFN, in both *in vitro* and *in vivo* animal models of cerebral ischemic disease and SAH. .

In vivo PK studies demonstrate similar PK properties for SFN when delivered from SFX-01 or synthetic SFN itself. As such, we believe that SFX-01 has the potential to offer significant therapeutic benefit for patients with SAH.

In pre-clinical toxicology and safety pharmacology studies of SFX-01, the principle toxicities were GI disturbance, which in dogs was manifested as severe vomiting, precluding their use as a study species. The only other toxicity of note was diffuse urothelial hyperplasia. This was observed in both rat and primate 4-week studies; in the rat, at all doses (i.e. No-Observed-Adverse-Effect level (NOAEL) was undetermined), in the primate, a NOAEL of 65 mg/kg/day was identified. There was also mild epithelial hyperplasia observed in the stomach and small intestine of the rats but not primates, which recovered during the no-treatment period. The significance of this finding in the urothelium is not clear. It has been reported in the rat with a number of other compounds, such as phenacetin, penem antibiotics and sodium saccharin. In no cases did these findings occur in man.

SFN, the active component of SFX-01, has been extensively studied in man when delivered from botanical precursors e.g. myrosinase-activated glucoraphanin. Exposures of 1.3ug/ml (7.4µM) with Area Under the Curve (AUC) of 7.1ug.h/ml (7100ng.h/ml) have been reported in man. These doses produced only mild gastro-intestinal (GI) disturbances and no other significant toxicities [27]. Experience with broccoli sprout extracts, glucoraphanin and SFN in healthy volunteers and patients demonstrated that the associated principle adverse events were self-limiting mild GI disturbance. No significant symptomatic, biochemical or electrophysiological changes were reported.

Note that previous human experience with SFN or precursors to SFN, whilst reported in the literature, may not provide wholly convincing safety information since SFN exposures may have been significantly lower than will be achieved with SFX-01 dosed at 300mg bid.
Given this, the study has been designed with an explicit interim safety assessment by an independent Data Safety Monitoring Board (DSMB) after 20 patients have been dosed (randomised to SFX-01 or placebo) as inpatients. Note also that there is no female human experience with SFX-01; therefore gender difference in kinetics and/or tolerability have not been explored.

In initial clinical trials with SFX-01 (in 12 healthy males) there was no evidence of adverse biochemical or haematological effects in up to a single dose of 700 mg (EVG001N) and repeat (7 days) doses of 600 mg once daily or 300 mg bid (EVG002N).

The compound was well tolerated up to 300 mg but thereafter mild signs of GI effects were observed. Evgen Pharma plc believes that these are due to release of the active pharmaceutical ingredient in the stomach and have now developed an acid resistant capsule formulation to allow the dose to pass directly into the lower GI tract before release. These mild AEs were ameliorated by the acid resistant product and with food even at 600 mg doses.

The exposure in man at the top dose of 700 mg single dose and 600 mg repeat dose remained below those that produced any adverse effects in the primate repeat dose toxicology study. (Primate pharmacokinetics (PK) showed Mean Cmaxs at a dose of 65mg/kg/day of 209ng/ml on day 1 and 135ng/ml on day 28 with mean Areas Under the Curve of 498ng.h/ml on day 1 and 729ng.h/ml on day 28)

Daily doses of 600mg per day in man delivered SFN systemic exposures equivalent to the NOAELs seen in the primate repeat dose toxicology with SFX-01- i.e. there are no margins to the NOAEL. However, for the anticipated therapeutic dose (300 mg SFX-01 bid), the mean Cmax values for sulforaphane plasma concentration ranged between 81.63 ng/mL to 123.24 ng/mL. Median Tmax was found to occur between 1.00-3.00 hours and AUC 0-12 hours ranged from 244.06 ng·hr/ml to 306.09 ng·hr/ml.

The proposed clinical study in man involves daily doses of SFX-01 in patients with SAH at a dose of 300 mg bid SFX-01, (equivalent to a daily dose of 92 mg SFN). The potential benefit to patients is significant with reduced DCI, improved cognition and cerebral function. The risk is modest, with exposures expected to remain below those shown to produce any adverse effects in primates.

In summary, the preclinical data supports the premise that SFX-01 offers significant potential therapeutic benefits, without significant clinical toxicities. The adverse event profile of the compound in primates and Phase I clinical studies is modest; there is a significant margin between the proposed dose in man and that producing any toxicities in primate-repeat dose toxicity studies. As such, studies in patients with SAH are warranted, with a view to defining the clinical benefits and overall efficacy profile.

1.4. Study Rationale

This is a randomised, double-blind, parallel-group study comparing SFX-01 (300 mg) taken orally as capsules or as a suspension via a nasogastric tube (NG) twice-daily for up to 28 days versus placebo in 90 patients who have had SAH and present within 48 hours of ictus. The treatment group will receive SFX-01 in order to improve and reduce the long-term complications of SAH such as DCI, as reflected by Trans-Cranial Doppler (TCD) readings. The objective is to demonstrate safety and tolerability and search for pharmacokinetic and pharmacodynamic signals in patients that have suffered a SAH. The results of the study will be used to design adequately powered efficacy studies with defined clinical endpoints.

1.5. Dose Rationale

Animal studies in ischaemic stroke, intracerebral haemorrhage and subarachnoid haemorrhage have all used doses of 5mg/kg of sulforaphane in rodents [19, 22, 24, 28]. Conversion of this dose to humans following body surface area as has been widely recommended [29, 30] and yields an effective dose of SFN in humans of 50mg. This is equivalent to 300 mg of SFX-01 (300 mg of SFX-01 contains 46.15 mg of SFN).

In the clinical studies conducted to date, SFX-01 has been shown to be well tolerated up to 300 mg twice-daily with no serious adverse effects.

1.6. Trial Conduct

This study will be conducted in compliance with the protocol and according to Good Clinical Practice and applicable regulatory standards. No deviation from the protocol will be implemented without the prior review and approval by the relevant ethics and regulatory authorities, except where it may be necessary to eliminate an immediate hazard to a research subject. In such case, the deviation will be reported to the relevant ethics and regulatory authorities as soon as possible.

1.7. Population

The population to be studied are patients with spontaneous aneurysmal SAH, Fisher grade 3 or 4 on CT, who present within 48 hours of ictus.

2. STUDY OBJECTIVES AND ENDPOINTS

2.1. Primary Objective

Safety

To evaluate the safety of up to 28 days of SFX-01 dosed at up to 92mg Sulforaphane (SFN) per day.

Pharmacokinetic

To detect the presence of SFN in Cerebrospinal Fluid (CSF).

Efficacy

To determine if a minimum of 7 days treatment with SFX-01 reduces Middle Cerebral Artery (MCA) peak flow velocity following subarachnoid haemorrhage (SAH).

2.2. Secondary Objectives

The secondary objectives are:

- To determine if a minimum of 7 days treatment with SFX-01 improves clinical outcome following SAH as measured using the modified Rankin Scale assessed at 7 days, discharge, 28, 90 and 180 days post ictus.
- To determine blood SFN levels (and its metabolites) with treatment with SFX-01 (300mg bid).
- To determine CSF SFN levels and kinetics with treatment with SFX-01 (300mg bid).
- To determine if up to 28 days treatment with SFX-01 increases blood haptoglobin (HP) levels and decreases malondialdehyde (MDA) levels following SAH.
- To determine if up to 28 days treatment with SFX-01 can reduce the incidence of Delayed Cerebral Ischaemia (DCI) following SAH.
- To determine if up to 28 days treatment with SFX-01 improves long-term outcome in subjects following SAH.
- To determine if up to 28 days of treatment with SFX-01 can reduce iron deposition and cortical atrophy following SAH.

2.3. Primary Outcome Endpoints

The primary outcome endpoints are:

Safety

- Concomitant medication
- Adverse events
- Change in grading of AE severity
- FBC, U&Es, LFT, CRP & Urine Microscopy
- INR or PT, APTR or APTT, & Fibrinogen (Clauss or Derived)at 7 & 28 days

Pharmacokinetic

- Presence of SFN (or its metabolites) in CSF

Efficacy

- The maximum Middle Cerebral Artery (MCA) velocity determined using Trans-Cranial Doppler (TCD)

2.4. Secondary Outcome Endpoints

The secondary outcome endpoints are:

- Modified Rankin Scale (mRS), at 7 days, discharge, 28, 90 and 180 days.
- Incidence of Delayed Cerebral Ischaemia (DCI) defined as a new focal deficit or reduction in Glasgow Coma Scale (GCS) ≥2 if not explained by other causes (i.e. re-bleed, hydrocephalus, seizure, meningitis, sepsis or hyponatraemia)
- Incidence of new cerebral infarct on Computed Tomography (CT) or Magnetic Resonance Imaging (MRI).
- Institution of hypertensive (triple H) therapy for presumed DCI
- SF-36 quality of life survey at 28, 90 & 180 days.
- Checklist for Cognitive and Emotional Consequences (CLCE-24), Brain Injury Community Rehabilitation Outcomes Scale (BICRO-39) at 90 & 180 days.
- Subarachnoid Haemorrhage Outcome Tool (SAHOT) and Glasgow Outcome Scale – Extended (GOSE) at 28, 90 & 180 days.
- Length of acute hospital stay
- Discharge location
- Amount of iron identified on MRI Susceptibility Weighted Imaging (SWI) 180 days after start of treatment.
- Cortical atrophy on T1 MRI at 180 days after start of treatment

Non-EVD Patients: i.e. patients will have a Lumbar Puncture for Collection of CSF

- Blood HP and MDA, Proteomic & Genomic concentration at baseline (pre dose 0-48 hours), D7 and D28
- Paired $CSF_{(Lumbar\ Puncture)}$/blood HP, MDA, Proteomic & Genomic & SFN/SFN metabolite concentration at Day 7

EVD Patients: i.e. Will not have a lumbar puncture

- Blood HP, MDA, Genomic & Proteomic concentration at baseline (pre dose 0-48 hours), D7 and D28

- Paired $CSF_{(EVD)}$/blood HP, MDA & Proteomic/Genomic concentration on alternate days (+/- 1 day) starting from day of EVD fitting to D14 or until EVD removal.
- Paired $CSF_{(EVD)}$/blood HP, MDA, Proteomic/Genomic & SFN/SFN metabolite concentration at day 7

Subset of 12 EVD Patients: In addition to all other all other sampling the following samples will be taken:

- Serial paired $CSF_{(EVD)}$/blood SFN/SFN metabolite concentration at one of the first 3 doses and at day 7

3. STUDY DESIGN

3.1. Overall Study Design and Plan Description

This is a Safety, Tolerability, Pharmacokinetic and Pharmacodynamic Study of SFX-01 in Subarachnoid Haemorrhage with exploratory evaluations of efficacy.

The study is a randomised, double-blind, parallel-group design comparing SFX-01 (300 mg) taken orally as capsules or as a suspension via a nasogastric tube (NG) twice-daily for 28 days versus placebo in at least 90 patients who have had SAH and present within 48 hours of ictus.

The treatment group will receive SFX-01 in order to improve and reduce the long-term complications of SAH such as DCI, as reflected by TCD readings. The objective is to demonstrate safety and search for signals of efficacy in patients that have had a SAH.

A sub-study will be conducted in up to 12 patients with an External Ventricular Drain (EVD) fitted; serial CSF samples will be taken pre- & post-dose on two occasions to determine pharmacokinetics of SFN in CSF in comparison with plasma pharmacokinetics. Substudy patients will undergo all other procedures. Initial treatment duration will be for the length of time participants remain an inpatient in tertiary care (up to day 28 post ictus dosing) followed by treatment up to day 28 post ictus (including post-discharge); follow up duration is 28 days, three and six months. The planned trial period is 24 months.

The Per Protocol Population (for Primary analysis) will be considered to be those patients that have been dosed for a minimum of 7 days and for whom it can be shown that there were no dispensing errors.

Flowchart

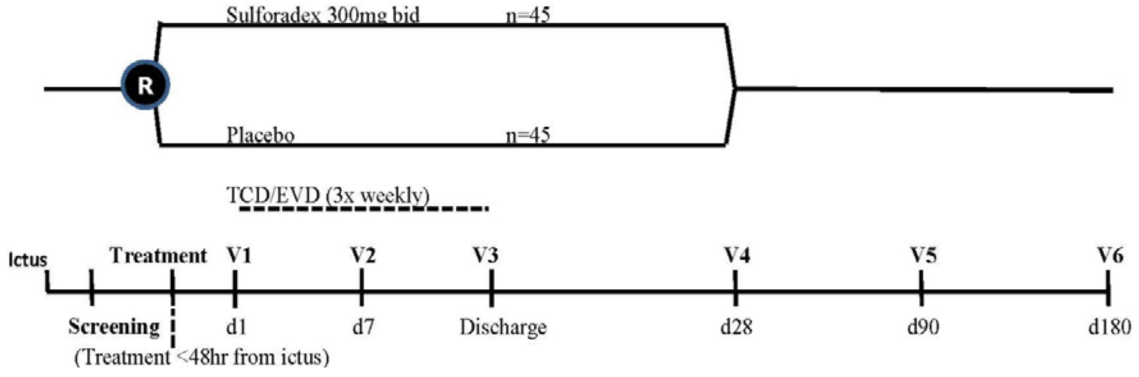

3.2. Duration of Treatment

Initial duration of treatment (for the first 20 patients) will be limited to the length of time they remain an inpatient in tertiary care (up to a maximum of day 28 post ictus). Direct supervision will be guaranteed during the acute inpatient stay in the neurological centre; patients will not be discharged with the investigational medicine.

Once 20 patients have completed dosing up to day 7 post ictus, a DSMB will convene to review the data; a decision will be made as to dosing after discharge from tertiary care.

The final duration of treatment is intended to be up to day 28 post ictus. However, as the sizable group of patients will return to their local hospital or rehabilitation units for further care, on discharge they will be supplied with the medication for the remainder of the treatment together with instruction for the discharge destination. If patients are to be discharged home, they or their carer will be given clear instruction on how to continue with the treatment and the need to store the IMP at the correct refrigerated storage temperature of between 2-8°C. In addition to detailed instructions a medication compliance diary will be provided on discharge and collected at Day 28 together with any remaining study medication for reconciliation.

The definition of “discharge” can change from site to site, therefore, for the purposes of this trial discharge is where the consultant responsible for the intervention of treating the SAH decides that their specialist care is no longer needed and they can safely transfer that care to another health care professional or send the patient home. Even if the patient remains in the same unit they will still be deemed to be discharged. The timing to the next assessment in the schedule should start when this decision has been made.

3.3. Premature Discontinuation of Treatment

Patients have the right to discontinue trial medication at any time and for any reason. The Investigator also has the right to discontinue trial medication if they feel that treatment is no longer appropriate, if in their opinion the patient’s clinical condition is worsening or for safety (adverse events).

Patients removed from treatment will be encouraged to continue in the study and complete the study visits in accordance with the study visit schedule.

Investigators may discontinue a participant from the trial at any time if they encounter any of the exclusion criteria as well as:

- Significant non-compliance with treatment regimen or trial requirements
- An adverse event which requires discontinuation of the trial medication or results in inability to continue to comply with trial procedures
- Disease progression which requires discontinuation of the trial medication or results in inability to continue to comply with trial procedures
- Lost to follow up

If a patient prematurely discontinues treatment the reason for discontinuation will be recorded on the electronic Case Report Form (eCRF). Unless the patient withdraws consent for the use of data, any data from a discontinued patient will still be utilised for study analysis.

3.3.1. Cessation of Treatment

A patient will be classified as having ceased treatment when he or she discontinues medication, prior to the completion of the prescribed course for any of the following reasons:

- Adverse Event

- Death
- Lost to follow up
- Withdrawal of consent
- Protocol Violation
- Pregnancy
- Treatment is no longer appropriate
- Termination of trial

3.3.2. Withdrawal from Trial

Patients have the right to withdraw from the trial at any time and for any reason. If a patient refuses to be seen for further visits the assessments for discharge or Day 28 will be performed at the time they have indicated that they will not attend for further visits, assuming they are available and consent to this.

3.3.3. Replacements

In order to provide reliable data from at least 90 patients who meet the per protocol requirements, additional patients may be recruited to replace individuals when there is evidence either that insufficient study medication has been taken or that there might have been errors in study medication dispensing.

3.4. Discussion of Study Design, Including the Choice of Control Groups

The study has a parallel group design, which is deemed more appropriate than a cross-over design considering the aetiology of the illness. Patients will be randomised, in equal proportions, to placebo or the active dose regimen (SFX-01 300 mg capsule taken orally twice-daily for 28 days).

The only effective treatment to reduce morbidity from SAH is nimodipine [4]. However, its effects are small and despite its use poor outcomes remain a significant problem as evidenced by contemporary outcome data since its introduction [5]. Moreover, even in the survivors conventionally considered to have made a good recovery, neurocognitive deficits are common leading to extensive problems with social reintegration and functioning in the workplace [6]. For this reason SFX-01 is compared to placebo only. However, SFX-01 will be used in conjunction with nimodipine as per routine clinical care. The study is randomised in order to prevent bias in the allocation of treatment and to ensure the comparability of baseline characteristics between the treatment groups. In order to prevent bias in the conduct of the clinical assessments, the study is double blind, so that neither the investigators nor the patient know whether the patient is receiving active treatment or placebo.

4. SUBJECT SELECTION CRITERIA

4.1. Subject Recruitment

All patients, admitted with a diagnosis of spontaneous SAH will be assessed by the study team against the inclusion and exclusion criteria. Identified subjects who fulfil the criteria will then be approached by a member of the research team on the delegation log, who will in turn obtain consent from the subject, the Personal Legal Representative or Professional Legal representative in the case of adults lacking capacity if possible (see section 14).

The Patient Information will be given to the subject, Personal Legal Representative or Professional Legal representative; they will be given sufficient time in order to make a decision.

In the case of adults lacking capacity where no Personal Legal Representative or Professional Legal representative is available patients may be randomised and receive the first two doses whilst consent is being sought. (***Note that this is only permissible where local regulations allow; if local regulations do not allow emergency dosing without consent the patient shall not be enrolled into the study.*** see section 14).

4.2. Inclusion Criteria

1. Patients with radiological evidence of spontaneous aneurysmal SAH
2. Fisher grade 3 or 4 on CT
3. Definitive treatment of aneurysm has not been ruled out
4. Previously living independently
5. In the opinion of the investigator, the delay from ictus to randomisation and initiation of trial medication will not exceed 48 hours
6. Aged 18 to 80 years
7. In the opinion of the investigator it will be possible to obtain Informed Consent from the Patient, Personal Legal Representative or Professional Legal representative within 24 hours of first dose

4.3. Exclusion Criteria

1 Traumatic SAH
2 Fisher grade 1 or 2
3 SAH diagnosed on lumbar puncture with no evidence of blood on CT
4 Decision not to treat aneurysm has been made
5 Plan to withdraw treatment
6 Significant kidney disease as defined as plasma creatinine ≥2.5mg/dL (221 µmol/l)
7 Liver disease as defined as total bilirubin ≥2-fold the upper limit of normal as measured by the local laboratory
8 Females who are pregnant or lactating.
9 Participants enrolled in another interventional research trial in the last 30 days
10 Patients for whom it is known, at the time of screening, that clinical follow-up will not be feasible
11 Patients unwilling to use two forms of contraception (one of which being a barrier method see section 7.9) 90 days for men and 30 days for women after last IMP dose
12 Known hypersensitivity to any component of a sulforaphane containing product including broccoli

4.4. Subject Withdrawals

In accordance with Informed Consent and the Declaration of Helsinki, the patient may discontinue the study at any time without giving any reason.

In all circumstances, Patients or Legal Representatives will be made aware of the rights to refuse participation in a clinical trial and will be entitled to freely withdraw their informed consent, without giving reasons. Patients or Legal Representatives should be assured that their withdrawal from the trial will not cause prejudice, will not result in any detriment and will not affect their treatment. In addition, refusal to give consent or withdrawal of consent to participate in research will not lead to any liability or discrimination (e.g., with regard to insurance or employment) against the person concerned.

In addition, the Investigator also has the right to withdraw subjects from the study for any reason.

Should a Patient and/or Legal Representative decide to withdraw for other reasons, all efforts will be made to complete and report the observations as thoroughly as possible. A complete final evaluation at the time of withdrawal will be performed with an explanation of the reason why the subject is withdrawing from the study.

The Investigator is responsible for the optimal individual treatment of the patient.

The Investigator must fill in the “Study termination” section in the eCRF describing all reasons for withdrawal.

After a patient withdraws from the trial, the Investigator remains responsible for reporting SAEs considered causally related to the study drug. In addition, the Investigator needs to ensure appropriate treatment and follow-up of each adverse event still ongoing at the time of the patient’s discontinuation.

4.4.1. Replacement of Withdrawn Patients

In the instance where a patient has been entered into the trial prior to informed consent being obtained (i.e. through the emergency consent procedure) and consent is subsequently refused or not obtained within 24 hours by the patient and/or legal representative, the participant shall be withdrawn and replaced.

Replacement of patients who withdraw/are withdrawn prior to completion of day 7 (post ictus) is to be discussed (by the Investigator and Sponsor) and approved by the Investigator and Sponsor’s Medical Monitor on a case by case basis.

Patients who withdraw for any other reason after randomisation will not be replaced.

Unless the patient withdraws consent for the use of data, any data from a discontinued patient will still be utilised for study analysis.

5. CONCOMITANT MEDICATIONS

Concomitant treatment will be permitted unless its use is contraindicated or there are significant interactions with SFX-01.

SFX-01 is contraindicated in those who are hypersensitive to any component of a SFN-containing product, including broccoli.

5.1. Permitted Concomitant Medications

In the context of aneurysmal SAH, all patients will also receive nimodipine 60 mg every four hours or 30 mg every two hours.

5.2. Non-Permitted Concomitant Medications

There are no known contraindicated medicines that have a significant interactions with SFX-01.

6. TREATMENTS

6.1. Appearance and Content

SFX-01 (active 300 mg capsule) and placebo (placebo 300 mg capsule) will be taken orally or via a naso-gastric tube twice-daily for 28 days.

6.2. Dosage and Administration

6.2.1. Trial Drug

Name: SFX-01

Presentation: Size 00 acid resistant Hydroxypropyl Methycellulose (HPMC) capsules (White OP) providing 300 mg SFX-01

6.2.2. Placebo

Identical size 00 acid resistant HPMC capsules containing 300 mg alpha cyclodextrin.

6.2.3. Administration

Capsules will be swallowed whole with water.

In patients who are unable to take tablets orally but have a NG tube in situ the study drug will be administered via the tube. The capsule will be opened in the neurointensive care or neurosurgical ward and emptied into 20ml water.

The trial medication will be administered to the patient twice-daily while an inpatient at the neurosurgical centre. The trial medication will be dispensed to the patient at discharge (once the 20 patient DSMB has agreed post-discharge dosing).

6.2.4. Dose Frequency De-escalation

In the event of tolerability problems whilst the patient is in the neurosurgical centre, the Investigator will assess whether simple measures to ease the effects of the adverse event(s) may be implemented (for example ant-acid in the case of GI irritation or anti emetic in the event of nausea).

The investigator will also assess whether or not the adverse event(s) could be related to the trial medication and severe enough to warrant a dose frequency reduction.

In the first instance the investigator may consider missing one dose.

If a dose frequency reduction is warranted, from that point onwards the second daily dose will be omitted; a dose frequency increase back to twice daily will not be permitted.

If tolerability problems continue then investigational medication will be stopped; patients will continue in the study and complete the study visits in accordance with the schedule of assessments.

The staged dose frequency de-escalation (dropping to once daily) will not be carried out after discharge from the neurosurgical centre; if tolerability problems occur after discharge dosing with the investigational medication will be stopped; patients will continue in the study and complete the study visits in accordance with the schedule of assessments.

6.3. Packaging & Storage

IMP is assembled for Qualified Person Certification and release for use in the clinical trial at the contract manufacturing company (PharMaterials) and delivered to sites at refrigerated conditions of between 2-8°C as required. The bulk manufactured IMP is placed into 60 ml Duma high-density polyethylene bottles in compliance with EU Directive 2002/72/EC and FDA title 21 CFR §177.1520 and closed with Duma 45 mm round plastic tamper-evident screw

cap with three breakpoints on the tamper-evident ring and integrated silica gel desiccant in compliance with the specification. Each bottle contains 56 size 00 capsules.

IMP is to be stored in refrigerated conditions of between 2-8°C at all times in the pharmacy with a limited number of bottles to be stored securely and in refrigerated conditions of between 2-8°C on the Neurointensive Care Unit, with access restricted to authorised personnel. When a patient is randomised, the next lowest numbered bottle within the specified strata will be utilised from the stock. A member of the research team will place the patient's name, date of birth and trial number on the bottle; it will be stored on the ward and be discharged with the patient.

Upon discharge from hospital the patient will be provided with a cool bag containing a refrigerated gel pouch for transportation of the IMP to home, or transfer to another hospital where upon the IMP will then be stored at the protocol required temperature of between 2-8°C at all times.

6.4. Labelling

All study medications will be labelled in accordance with Annex 13: Manufacture of investigational products.

Study medication packs will be labelled identifying the site and strata according to the WFNS grading scale score.

6.5. Blinding and Randomisation

6.5.1. Randomization

Patients will be allocated to double-blind medication through a stratified randomisation schedule with the strata defined by site and by baseline severity defined by WFNS score of 1-3 or 4 & 5.

All treatment packs will be otherwise identical in appearance, in order to maintain patient and investigator blinding throughout the trial. The contents will also be indistinguishable should they be opened either inadvertently or for the purposes of NG administration. Patients will be randomised to one of treatment groups by allocation of the appropriate, numbered, treatment pack. The treatment packs will be pre-numbered according to a block balanced randomisation code generated by PharMaterials.

Emergency code envelopes will be produced to provide details of the medication regimes each patient has been allocated to. Sealed code break envelopes will be held by the Pharmacy and by Diamond Pharma Services.

6.5.2. Blinding

<u>Code Breaks at the Trial Centre/ Diamond Pharma Services.</u>

The Pharmacy will receive a sealed envelope containing the identity of each trial medication bottle dispensed during the trial. An envelope may be opened only in the case of a serious adverse event and only when it is essential to the subsequent management of the patient. Diamond Pharma Services will be responsible for breaking codes for regulatory submissions of Suspected Unexpected Serious Adverse Reactions (SUSARs), thereby maintaining the overall confidentiality of the code breaks.

Where a code break has occurred, the Investigator must provide a written record of the circumstances surrounding the event but should take care not to record, on any trial

documentation, details of the unblinded treatment. Such details should be disclosed only to those persons who have responsibility for the immediate management of the patient. The Clinical Trial Company (TCTC) must be notified as soon as possible. If the code is broken the data for that patient will be excluded from the Per Protocol Population analysis but included in the Intention to Treat Analysis. They will continue in the study and complete the study visits in accordance with the study visit schedule.

At the end of the treatment phase of the trial, the envelopes must be returned, along with the drug dispensing records to the trial monitor. The envelopes will be checked to ensure that the seals have not been broken unless (unless a code break has been carried out). Emergency code envelopes may only be opened in an emergency, when the patient's condition requires knowledge of the test medication. Every attempt should be made to ensure that all persons directly involved in the trial remain ignorant of the randomisation codes. The Safety Committee will be able to request unblinding of patients (see section 25).

The randomisation code will not be fully revealed, other than in instances where code break is justified on grounds of safety, until all data have been gathered, entered into the database, clarified, resolved, verified, validated and the database has been closed. The code will then be broken by the statistician. The Investigator will be advised of allocation of trial treatment following communication of the results of the analysis.

Prevention of unblinding by laboratory measurements
Laboratory data for blood/CSF SFN levels will be entered into a separate database by a member of the research team. The data from the laboratory database will be transferred electronically to the main database.

Treatment compliance
Compliance with treatment will be recorded during the inpatient hospital stay by health care professionals and/or a member of the research team. On discharge to the usual residence this responsibility is to be carried out by the patient or their Personal Legal Representative, aided by detailed instructions.

In the event of discharge to a rehabilitation unit or patient local hospital, written instructions will be given to the patients on discharge and verbal communication with the clinical team will be made to ensure compliance.

All patients will be discharged with a patient diary which will be filled in and collected at Day 28.

Compliance will be further monitored by drug reconciliation. Patients will be asked to return the medication bottle and any residual contents at the Day 28 visit. At this time any residual tablets will be counted and recorded.

6.6. Drug Storage

All medication supplied in connection with the trial will be used only for this and no other purpose.

IMP will be stored under refrigerated conditions of between 2-8° C at all times in the Pharmacy, hospital wards and also upon discharge to another hospital and the patient's home. A cool bag containing a refrigerated gel pouch will be provided for transportation purposes.

IMP is to be stored in the pharmacy with a limited number of bottles to be stored securely on the Neurointensive Care Unit.

6.7. Drug Accountability

All members of the research team, including investigators are accountable for the supply of the medication during patients' hospital stay. This requires the keeping of records of dispensing medication, as well as inventory checks. All members of the study team will adhere to local guidelines in addition to GCP. The study drug will only be used in those who are enrolled on to the study and for the named patients only.

Record keeping including delivery of medication to pharmacy and wards, dispensing to the subject, unused study medication and return of unused medications will be continuously monitored and updated. Study medication will be prescribed on the drug chart and the nursing staff and/or study team involved will keep daily records of its administration.

7. STUDY PLAN

7.1. Continuous Assessments:

The following assessments will be carried out on a continuous basis and as events arise:

- Adverse Events
- Concomitant Medication
- Medication Compliance

7.2. Pre-Dose Assessment (within 48 hours of ictus):

The following assessments will be performed and documented in all patients admitted with a diagnosis of spontaneous aneurysmal SAH:

- Informed Consent (within 24 hours of first dose)
- Inclusion/Exclusion (screen failures to be recorded)
- Recording results of CT/MRI & Fisher grading (Required for SAH diagnosis)
- Demographics/Medical History
- Physical Examination
- Pregnancy test - urine or blood is acceptable
- Concomitant Medication,
- Protocol specific Safety Bloods and Urine (Biochemistry, Haematology, Lipid Profile & Coagulation Status - see Section 8.1). If no predose safety urine is available, then baseline urine taken prior to day 2 dosing will be acceptable.
- Blood HP, MDA, Proteomic/Genomic sampling
- Best World Federation of Neurological Societies (WFNS) scale at first presentation, on admission to the neurosurgical unit and after resuscitation (when this should occur)

For patients lacking capacity where a Personal Representative is not immediately available in person, a Professional Legal Representative will be sought. If they are in attendance in person they will discuss the trial with the research team and complete a consent form if they feel it is appropriate for the subject to participate in the trial.

If they are not in attendance in person they will be contacted by telephone and their opinion sought. If in agreement, the study team will document this in the patient notes (details of the representative, date and time of the telephone call, summary of the discussion and Informed Consent process and version of the Informed Consent Form), the patient will be enrolled and the Professional Legal Representative will complete a consent form the next time they attend the patient.

For patients lacking capacity where a Personal Legal Representative was not immediately available, written informed consent will be obtained and documented from the Personal Legal Representative at the earliest opportunity.

For patients lacking capacity at screening, with informed consent obtained from their Personal Legal Representative or Professional Legal Representative, written informed consent will be obtained and documented from the patient as soon as they regain consciousness sufficiently to do so.

In the case of adults lacking capacity where no Personal Legal Representative or Professional Legal representative is immediately available (including Professional Legal Representative unavailable by telephone) patients may be randomised and receive the first two doses whilst consent is being sought. (***Note that this is only permissible where local regulations allow; if local regulations do not allow emergency dosing without consent the patient shall not be enrolled into the study.)***

In the instance where a patient has been entered into the trial prior to informed consent being obtained (i.e. through the emergency consent procedure) and consent is subsequently refused or not obtained within 24 hours by the patient and/or legal representative, the participant shall be withdrawn and replaced (see section 14).

If the patient has not regained capacity by the time of the 180 Day follow up no further attempts will be made to obtain consent directly from the patient.

7.3. Dose (within 48 hours of ictus)

Eligible patients will be randomized to either trial drug (SFX-01 300 mg) or placebo. The first administration of trial medication must not exceed 48 hours from ictus.

Patients are to be dosed with study drug (SFX-01 or placebo) twice daily, approximately 12 hours apart, until Day 28 post ictus.

7.4. Post Dose (12-24 hours after first dose)

The following procedure will be carried out after the first dose:

- Protocol specific safety blood tests (see Section 8.1)

7.5. Between Pre-dose Assessment & Post Dose:

The following assessments are to be carried out pre or post dose.

- Recording of results and form of angiographic assessment by Computed Tomography Angiography/Digital Subtraction Angiography/Magnetic Resonance Angiography
- First Trans-Cranial Doppler reading (to measure peak MCA flow)

The Angiographic Assessment (CTA/DSA/MRA) and procedure planned and carried out (clipping/coiling) is to be recorded

7.6. Ongoing Assessments – All patients:

The following procedures and assessments are to be carried out on alternate days until no longer clinically indicated:

- Safety Bloods as part of normal standard clinical care

- TCD readings (to be performed on alternate days (± 1) post ictus (starting day 3 (± 1) until at least Day 7 (± 1) or discharge whichever is sooner. Any additional TCD readings obtained after this point will also be recorded)
- Glasgow Coma Score

7.7. Patients with an External Ventricular Drain fitted: Ongoing EVD single sampling (on alternate days +/- 1day from day of EVD fitting until D14 or until the EVD is removed):

Patients with an External Ventricular Drain (as part of standard of care) will have the following procedures and assessments carried out on alternate days (±1) from the day of EVD fitting until removal:

- An alternate day single EVD CSF sample for determination of HP & MDA levels
- An alternate day single blood sample for HP, MDA & Proteomic/Genomic analysis

The samples are to be taken at the same time (paired sample).

7.8. Day 7 post ictus (± 1 day):

The following procedures and assessments are to be carried out at Day 7 post ictus:

- Protocol specific Safety Bloods and Urine (see Section 8.1)
- TCD reading
- modified Rankin Scale
- Concomitant Medication Review

Non-EVD patients only:

- Paired blood Sampling and lumbar puncture to determine CSF/blood HP, MDA, Proteomic /Genomic & SFN/SFN metabolite concentrations

EVD patients only:

- Paired blood and EVD sampling to determine CSF/blood HP, MDA, Proteomic /Genomic & SFN/SFN metabolite concentrations

7.9. Within 2d of discharge:

The definition of “discharge” can change from site to site, therefore, for the purposes of this trial discharge is where the consultant responsible for the intervention of treating the SAH decides that their specialist care is no longer needed and they can safely transfer that care to another health care professional or send the patient home. Even if the patient remains in the same unit they will still be deemed to be discharged. The timing to the next assessment should start when this decision has been made.

The following procedures and assessments are to be carried within two days of discharge from the neurosurgical unit:

- Protocol specific Safety Bloods and Urine (see Section 8.1)
- modified Rankin Score
- Glasgow Coma Score

If the outcome of the 20 patient DSMB has determined that post-discharge dosing is acceptable, the patient will be discharged with sufficient medication to ensure full-compliance until Day 28

post-ictus. If patients are to be discharged home they or their Personal Legal Representative will be given clear instruction on how to continue with the treatment including the requirements of refrigerated storage conditions of between 2-8°C at all times. In the event of discharge to a rehabilitation unit or hospital, written instructions will be given to the patients on discharge and verbal communication with the clinical team will be made to ensure compliance. All patients will be discharged with a treatment compliance sheet (diary) which will be filled in and collected at Day 28 post ictus.

The patient will be reminded to use two forms of contraception (one of which being a barrier method) for 90 days for men and 30 days for women (see Contraceptive Requirements)

The patient's GP will also be informed of the patient's discharge and entry in to the clinical trial. If discharged to home, the patient, or their carer, will receive a follow-up phone call within 2 days following discharge.

Contraceptive Requirements:

Male subjects

Male subjects whose female partner(s) is (are) pregnant must use a condom from the time of the first administration of treatment or study medication until three months (90 days) following administration of the last treatment or dose of study medication.

If the subject has undergone surgical sterilisation (vasectomy with documentation of azoospermia) a condom must be used.

Male subjects must use acceptable methods of contraception if the male subject's partner could become pregnant from the time of the first administration of treatment or study medication until three months following administration of the last treatment or dose of study medication. The acceptable methods of contraception are as follows:

- Condom and occlusive cap (diaphragm or cervical/vault caps) with spermicidal foam/gel/film/cream/suppository
- Surgical sterilisation (vasectomy with documentation of azoospermia) and a barrier method (condom or occlusive cap (diaphragm or cervical/vault caps) used with spermicidal foam/gel/film/cream/suppository)
- The female partner uses oral contraceptives (combination oestrogen/ progesterone pills), injectable progesterone or subdermal implants and a barrier method (condom or occlusive cap (diaphragm or cervical/vault caps) used with spermicidal foam/gel/film/cream/suppository)
- The female partner has undergone documented tubal ligation (female sterilisation). In addition, a barrier method (condom or occlusive cap (diaphragm or cervical/vault caps) used with spermicidal foam/gel/film/cream/suppository) must be used
- The female partner has undergone documented placement of an intrauterine device (IUD) or intrauterine system (IUS) and the use of a barrier method (condom or occlusive cap (diaphragm or cervical/vault caps) used with spermicidal foam/gel/film/cream/suppository)
- True abstinence when this is in line with the preferred and usual lifestyle of the subject. *Periodic abstinence (e.g., calendar, ovulation, symptothermal, post-ovulation methods) and withdrawal are not acceptable methods of contraception*

Female subjects

Female subjects of childbearing potential must use medically acceptable methods of contraception from the time of the first administration of treatment or study medication until one month (30 days) following administration of the last treatment or dose of study medication. Acceptable methods include:

- A documented placement of an IUD or IUS and the use of a barrier method (condom or occlusive cap (diaphragm or cervical/vault caps) used with spermicidal foam/gel/film/cream/suppository)
- Documented tubal ligation (female sterilisation). In addition, a barrier method (condom or occlusive cap (diaphragm or cervical/vault caps) used with spermicidal foam/gel/film/cream/suppository) should also be used;
- Double barrier method: condom and occlusive cap (diaphragm or cervical/vault caps) with spermicidal foam/gel/film/cream/suppository
- Oral contraceptives (combination oestrogen/progesterone pills), injectable progesterone or subdermal implants and the use of a barrier method (condom or occlusive cap (diaphragm or cervical/vault caps) used with spermicidal foam/gel/film/cream/suppository)
- True abstinence when this is in line with the preferred and usual lifestyle of the subject. *Periodic abstinence (e.g., calendar, ovulation, symptothermal, post-ovulation methods) and withdrawal are not acceptable methods of contraception*

7.10. Day 28 post ictus (-6/+2 days):

The following procedures and assessments are to be carried out at the Day 28 post ictus visit:

- modified Rankin Score
- Short Form 36 Health Survey
- Subarachnoid Haemorrhage Outcome Tool
- Glasgow Outcome Scale (Extended)
- Concomitant Medication Review
- Protocol specific Safety Bloods and Urine (see Section 8.1)
- Blood sampling for HP & MDA, Proteomic /Genomic determination

Patients stop taking study medication at this visit – any remaining IMP will be collected.
It is anticipated that some patients will not be able to attend this visit in person. In this event, a member of the research team will visit the patient for sample collection (other assessments may be made by telephone).

7.11. Day 90 post ictus (± 14):

The following procedures and assessments are to be carried out at Day 90 post ictus:

- modified Rankin Score
- Short Form 36 Health Survey
- Checklist for Cognitive and Emotional Consequences
- Brain Injury Community Rehabilitation Outcomes Scale
- Subarachnoid Haemorrhage Outcome Tool
- Glasgow Outcome Scale (Extended)
- Concomitant Medication Review

A telephone interview with the patient or Personal Legal Representative will be arranged if attendance at the neurosurgical centre is not feasible. Where preferred by patients or Personal Legal Representatives questionnaires may be sent by mail or email.

7.12. Day 180 post ictus (± 28):

The following procedures and assessments are to be carried out at Day 180 post ictus:

- modified Rankin Score
- Short Form 36 Health Survey
- Checklist for Cognitive and Emotional Consequences
- Brain Injury Community Rehabilitation Outcomes Scale
- Subarachnoid Haemorrhage Outcome Tool
- Glasgow Outcome Scale (Extended)
- Concomitant Medication Review

- An MRI will be performed within 60 days of the Day 180 visit

A telephone interview with the patient or Personal Legal Representative will be arranged if attendance at the neurosurgical centre is not feasible. Where preferred by patients or Personal Legal Representatives questionnaires may be sent by mail or email.

7.13. Pharmacokinetic sub-study

A group of up to 12 patients, all of whom have been fitted with an EVD as part of normal treatment and prior to randomisation, may be selected for a pharmacokinetic sub-study. Additional patient consent will be required for the sub-study; in cases where patients lack capacity the consent of a Personal Legal Representative will be sought before any sub-study procedures are carried out.

The patients will, in addition to all other procedures (with the exception of lumbar puncture), undergo serial paired blood/CSF sampling (1 sample pre dose and hourly ± 5 minutes for 6 hours post dose) at one of the first three doses and day 7±1 post ictus.

7.14. Early Termination Visit

Patients have the right to withdraw from the trial at any time and for any reason. If a patient refuses to be seen for further visits the assessments at discharge & Day 28 should be performed at the time they have indicated that they will not attend for further visits, assuming they are available and consent to this.

7.15. Schedule of Assessments

	Pre-Dose Assessment	Dose	Post Dose	Ongoing assessments	Day 7 (±1)	At Discharge[1] (-2)[2]	Day 28 (-6/+2))	Day 90 (±14)	Day 180 (±28)
Time after Ictus →	0-48 hrs		12-24 hrs post dose						
Consent	X								
Inclusion/Exclusion	X								
Record results of CT/MRI & Fisher[3]	X								
Angiographic[4] assessment		X							
Medical history	X								
Physical exam	X								
Pregnancy Test[5]	X								
Con med review	X				X	X	X	X	X
Adverse events	X	X	X	X	X	X	X	X	X
Safety bloods[6]	X		X	X[7]	X	X	X[8]		
Lipid Profile & Coagulation Status[9]	X				X		X		
Safety urine[10]	X				X	X	X[8]		
Randomisation		X							
IMP Treatment[11]		X	X		X	X	X[8]		
TCD reading[12]		X[13]		X[14]	X				
Blood samples taken for HP, MDA & Proteomic / Genomic analysis (patients with an EVD fitted)[15]	X				X[16]		X[8]		
EVD CSF & paired blood sample taken for SFN & metabolites analysis (patients with an EVD fitted)[15]					X[16]				
Blood samples taken for HP, MDA & Proteomic / Genomic analysis (Patients without an EVD fitted)	X				X[16]		X[8]		
Lumbar Puncture CSF & paired blood samples for SFN & metabolites analysis (Patients without an EVD fitted)					X[16]				
Medication compliance			X		X	X	X		
WFNS (score required for randomisation to correct strata)	X								
mRS					X	X	X	X	X
SF36							X	X	X
CLCE-24								X	X
BICRO-39								X	X
SAHOT							X	X	X
GOSE							X	X	X
GCS				X		X			
MRI									X[17]

Main study: EVD Patients
Patients with an EVD fitted will also undergo paired single CSF/blood sampling on alternate days following the SAH (starting from day of EVD fitting) until day 14 or the EVD is removed – i.e. a single EVD sample is to be taken and paired with a single blood sample for determination of CSF/blood HP & MDA levels (and SFN at day 7).

---

[1]Discharge is when the consultant for the intervention of treating the SAH decides that their specialist care is no longer needed
[2]Procedures can be carried out up to 2 days prior to discharge
[3] Required for SAH diagnosis
[4] The Angiographic Assessment (CTA/DSA/MRA) and procedure planned and carried out (clipping/coiling) to be recorded – this can be carried out pre or post dose
[5] Urine or blood pregnancy test can be taken
[6] Safety bloods as per protocol requirements taken at predose, post dose, day 7, discharge and day 28 the tests required comprise:
Biochemistry: Sodium, Potassium, Urea, Creatinine, Glucose, Calcium, Total Bilirubin, Alkaline Phosphatase, Alanine Transaminase, Albumin, C-Reactive Protein /
Haematology: Haemoglobin, White Blood Cell Count, Neutrophils (Absolute), Lymphocytes (Absolute), Platelets
[7] Safety bloods carried out as part of normal SAH clinical care until no longer clinically indicated
[8] A member of the research team will visit the patient (other assessments may be made by telephone)
[9] Lipid Profile: LDL, HDL, Triglycerides, Total Cholesterol; coagulation status: INR or PT, APTR or APTT, & Fibrinogen (Clauss or Derived)
[10] Urine Microscopy. If no predose safety urine is available, then baseline urine taken prior to day 2 dosing will be acceptable
[11] Twice Daily approx. 12 hours apart until day 28 post ictus, time of dosing to be recorded. Dosing after discharge to be allowed dependent on 20 patient DSMB review.
[12] Reading Peak Velocity MCA flow
[13] Where possible - record timing of first TCD & whether pre- or post- dose
[14] TCD to be performed on alternate days (± 1) post ictus (starting day 3 (± 1) until at least Day 7 (± 1), or discharge whichever is sooner. Any additional TCD readings obtained after this point on clinical grounds will also be recorded.
[15]EVD Patients **do not** undergo Lumbar Puncture however if in the sub study set of patients, have serial paired blood and CSF samples as described in the protocol at one of the first 3 doses and on day 7.
[16]Paired blood sample taken at same time as Lumbar Puncture
[17]Within 60 days of 180 day target

Additional Interventions for Pharmacokinetic Sub-Study (Serial EVD sampling)

Substudy patients will undergo all procedures (except Lumbar Puncture) and *additionally* serial paired CSF_{EVD}/ blood sampling at one of the first three doses and on day 7 for determination of SFN metabolites:

	Pre-Dose Assessment	Dose	Post Dose	7 days (±1)	At Discharge (-2)	28 days (-6/+2)	90 days (±14)	180 days (±28)
Time after Ictus →	0-48 hrs							
Consent[18]	X							
Serial CSF Sampling (EVD)		X[19]		X[19]				
Serial blood SFN metabolites		X[20]		X[20]				

Note that alternate-day paired single CSF/blood sampling is also carried out with Sub-Study patients (as with all EVD patients): CSF sample for determination of HP & MDA levels & blood sample for HP, MDA & Proteomic/Genomic analysis.

[18] Substudy specific consent
[19] Trough EVD CSF sample taken prior to dose and every hour ± 5 minutes after dosing for six hours for determination of SFN metabolites
[20] Paired blood sample taken at same time as EVD sampling for determination of SFN metabolites

8. STUDY PROCEDURES / EVALUATIONS

8.1. Safety Measurements

The following safety parameters will be recorded according to the trial protocol:

- Concomitant medication
- Adverse events
- Escalation in grading of AE severity
- Safety blood tests
  - Safety Blood Tests should include the following parameters:
  - Biochemistry
    - Sodium
    - Potassium
    - Urea
    - Creatinine
    - Glucose
    - Calcium
    - Total bilirubin
    - Alkaline Phosphatase
    - Alanine Transaminase
    - Albumin
    - C-reactive protein
  - Haematology
    - Haemoglobin
    - White Blood Cell count
    - Neutrophils (absolute)
    - Lymphocytes (absolute)
    - Platelets
  - Lipid Profile
    - LDL
    - HDL
    - Triglycerides
    - Total Cholesterol
  - Coagulation Status
    - PT (or INR)
    - APTT (or APTR)
  - Fibrinogen

- Safety Urine tests (Microscopy)
  - Urine microscopy will be performed in accordance with local procedures. If no predose safety urine is available, then baseline urine taken prior to day 2 dosing will be acceptable.

8.2. Efficacy Measurements

The following efficacy parameters will be recorded according to the trial protocol:

- TCD
- WFNS
- mRS
- SF-36
- CLCE-24
- BICRO-39
- SAHOT
- GOSE
- GCS
- MRI

8.3. PK Measurements

The following PK parameters will be recorded according to the trial protocol:

Non-EVD Patients: i.e. patients will have a Lumbar Puncture for Collection of CSF

- Blood HP and MDA, Proteomic & Genomic concentration at baseline (pre dose 0-48 hours), D7 and D28
- Paired $CSF_{(Lumbar\ Puncture)}$/blood HP, MDA, Proteomic & Genomic & SFN/SFN metabolite concentration at Day 7

EVD Patients: i.e. Will not have a lumbar puncture

- Blood HP, MDA, Genomic & Proteomic concentration at baseline (pre dose 0-48 hours), D7 and D28
- Paired $CSF_{(EVD)}$/blood HP, MDA & Proteomic/Genomic concentration on alternate days (+/- 1 day) starting from day of EVD fitting to D14 or until EVD removal.
- Paired $CSF_{(EVD)}$/blood HP, MDA, Proteomic/Genomic & SFN/SFN metabolite concentration at day 7

Subset of 12 EVD Patients: In addition to all other all other sampling the following samples will be taken:

- Serial paired $CSF_{(EVD)}$/blood SFN/SFN metabolite concentration at one of the first 3 doses and at day 7

8.4. Definitions of Assessments

8.4.1. Fisher Grade

The Fisher grade is commonly used to predict the risk of cerebral vasospasm after SAH based on the amount of blood shown on initial CT scans within 5 days of SAH.

The Fisher grading system is split into four levels:

Grade 1: No blood

Grade 2: Diffuse or thin layer of blood less than 1 mm thick (interhemispheric, insular, or ambient cisterns)

Grade 3: Localized clots and/or layers of blood greater than 1 mm thick in the vertical plane

Grade 4: Intracerebral or intraventricular clots with diffuse or absent blood in basal cisterns

8.4.2. World Federation of Neurological Societies Grading System For Subarachnoid Hemorrhage (WFNS)

The WFNS is a 5 point scale that is a simple, reliable and clinically valid way to grade a patient with SAH. This system offers less inter-observer variability than some of the earlier classification systems. Randomisation of patients in this study is to either WFNS score strata 1-3 or WFNS score strata 4-5.

8.4.3. Modified Rankin Scale (mRS)

The mRS is widely used as a functional outcome measure in stroke. The purpose of the Rankin Focused Assessment (RFA) is to assign patients to mRS grades in a systematic way. The assessment consists of sections corresponding to levels of disability among stroke survivors on the mRS.

8.4.4. Short Form Health Survey (SF-36)

The SF-36 quality of life scale is a multi-purpose, short-form health survey with only 36 questions. It yields an 8-scale profile of functional health and well-being scores as well as psychometrically-based physical and mental health summary measures and a preference-based health utility index. It is a generic measure and the SF-36 has proven useful in surveys of general and specific populations, comparing the relative burden of diseases, and in differentiating the health benefits produced by a wide range of different treatments.

8.4.5. Checklist for Cognitive and Emotional Consequences (CLCE-24)

The CLCE-24 is a checklist used for identification of cognitive and emotional problems after stroke. The CLCE-24 is a usable and valid instrument for cognitive screening by health care professionals in the stroke service in the chronic phase after stroke.

8.4.6. Brain Injury Community Rehabilitation Outcomes Scale (BICRO-39)

The BICRO-39 questionnaire is a multidimensional, quantitative assessment designed to measure community functioning in areas of activity, social participation, and psychological components. This assessment requires patients and/or caregivers to evaluate the level of functioning on each item pre- and post-injury. It can also be used to track changes in performance across time. Functional areas assessed include personal care, psychological, socializing, self-organization, mobility, productive employment, and family contact.

The questionnaire consists of three forms: patient pre-injury (P-PRE), patient post-injury (P-POST) and carer post-injury (C-POST) with each item on the questionnaires assigned a score of zero to five.

8.4.7. Subarachnoid Haemorrhage Outcome Tool (SAHOT)

The SAHOT is a new SAH-specific outcome assessment form that is filled by patient and Personal Legal Representative. SAHOT was designed and developed in Southampton University to assess recovery following SAH. It consists of a series of questions which aim to assess the degree of change in the above fields following SAH. The degree of change is graded into five main categories. This questionnaire demonstrates the impact of SAH in four main aspects of daily life including: general aspects, physical, cognitive and behavioural/psychological.

8.4.8. Glasgow Outcome Scale Extended (GOSE)

The Glasgow Outcome Scale is a global scale for functional outcome that rates patient status into one of five categories: Dead, Vegetative State, Severe Disability, Moderate Disability or Good Recovery. The Extended Glasgow Outcome Scale (GOSE) provides more detailed categorization into eight categories by subdividing the categories of severe disability, moderate disability and good recovery into a lower and upper category.

A structured interviews guide will be used for this trial.

8.4.9. Glasgow Coma Score (GCS)

The GCS is a standard measure to assess the level of consciousness of patients who have sustained head injuries. The GCS is part of standard treatment protocols and used for general decision-making for critically ill patients. It is an objective and reliable scale employed for initial and subsequent assessments. It consists of three items, Eyes (E), Verbal (V), and Motor (M) with each domain scoring a minimum of 1 giving an overall score ranging from 3 to 15.

8.5. Clinical Evaluations

8.5.1. Medical History

All patients will typically have full medical history taken followed by thorough physical examination as part of their admission routine.

8.5.2. Radiology

CT (or MRI) will be used to confirm the presence of SAH.

CTA, DSA or MRA will be used to confirm the presence of an aneurysm in this cohort of patients as per normal clinical care.

8.5.3. TCD Recordings

Trans Cranial Doppler readings are obtained on alternate days (± 1) post ictus (starting day 3 (± 1) until at least Day 7 (± 1)) or discharge whichever is sooner. Any additional TCD readings obtained after this point will also be recorded as this may continue as per usual until the subject is discharged from the neurosurgical Centre. TCDs will be performed by an experienced member of the medical physics or neuro-intensive care team or other appropriately trained personnel who have otherwise carried out the same procedure on the same group of patients. The readings will be kept in the patient's medical notes and values will be entered on to the assessment eCRF by the research team.

8.5.4. MRI

All patients will have an MRI with Susceptibility Weighted Imaging (SWI) sequences performed at six months (+/- 60 days).

In those patients whose aneurysm have been coiled (expected to be ~70% of patients) it would be expected that if they had recovered sufficiently that they would undergo an MRI on clinical grounds at this time. For these patients the SWI sequences will be added to the same MRI session as their scheduled clinical scan. For patients whose aneurysms have been surgically clipped or patients who have been coiled but are elderly in whom retreatment of any aneurysmal recurrence would not normally be contemplated, this is likely to represent an extra study intervention.

To quantify brain atrophy, 3D T1-weighted images (with high resolution isotropic voxels) will be acquired using neurosurgical centre's standard protocol. Whole brain atrophy will be assessed by using the modified cella media index and by more detailed analysis deriving CSF to intracranial volume ratio after image segmentation (using Statistical Parametric Mapping Software). Voxel-based morphometry will be used to identify and quantify regional areas of atrophy. Clip/coil artefact will be masked to ensure correct normalization of images and segmentation into CSF, grey matter and white matter.

To quantify iron, fully flow-compensated, 3D, high-resolution, gradient-echo Susceptibility-Weighted Imaging sequences will be used to collect magnitude and phase data. The phase images will be processed and analysed (using Signal Processing in NMR software) to quantify iron in regions of interest, such as orbitofrontal cortex, inferior temporal gyrus, hippocampus, parahippocampal gyrus and thalamus – i.e. the basal regions likely to be most exposed to haemoglobin following SAH.

8.6. Clinical Laboratory Evaluations

The following investigations will be performed during the acute admission of the subject to the neurosurgical centre. The details will be recorded on the eCRF form and upon completion they will be filed in the Trial Master File (TMF). Samples will be analysed at the local laboratory at the neurosurgical centre.

8.6.1. Blood Sampling

During the inpatient stay at the neurosurgical centre, routine blood tests including FBC, U&Es, LFTs and CRP are taken regularly, initially daily and later on an alternate daily basis as part of their care in NICU or neurosurgical wards. The results from these tests will be required for study purposes and at several specific time points. These will be measured at baseline on admission (pre-dose assessment), within 24 hours of first dose, at day 7 post ictus, discharge and at day 28.

As well as routine blood tests Coagulation status (INR or PT, APTR or APTT, & Fibrinogen (Clauss or Derived)) will be measured at Pre-Dose Assessment, day 7 and day 28.

8.6.2. Pregnancy Testing

Women of childbearing potential (all premenopausal women, or in cases where menstrual status cannot be ascertained including women under the age of 55) should have a urine pregnancy test performed at screening before study drug initiation.

8.6.3. Lumbar Puncture

Patients (without an External Ventricular Drain fitted) will have a lumbar puncture performed on day 7 following the SAH for study purposes. In many patients they will have this performed for clinical reasons during this window and thus the test will not be repeated. Around 30% of eligible patients will have an External Ventricular Drain (EVD) sited for clinical reasons and CSF will be obtained through the EVD i.e. if patients have an EVD fitted lumbar puncture is not carried out.

For the remaining patients that are required to have a lumbar puncture for study purposes alone this can be justified by:

1 the known high incidence of low grade hydrocephalus which may be diagnosed and relieved by lumbar puncture

2 the known advantage of CSF drainage to reduce blood load and reduce inflammation

The benefits of these effects have been demonstrated previously in a randomised controlled trial [9] showing a significantly better short term outcome with CSF drainage although the significance of this effect was lost on longer term follow up; hence it has not necessarily been seen as cost effective and universally adopted. However, in the context of this study there is good evidence that CSF drainage is at least beneficial in the short term and not harmful in the long term and can thus be justified as a study specific intervention.

Opening pressure will be recorded and approximately 17 ml of CSF will be taken at the time of lumbar puncture for research purposes or as much as is required to halve the pressure (as per routine clinical practice). Of the volume collected, 1 ml will be sent for routine microbiological assessment (microscopy culture and sensitivity). The remainder will be taken for study purposes to measure HP, MDA and SFN levels. Paired blood samples will be taken at the same time (for HP, MDA & SFN determination).

In the event that patients undergo further lumbar puncture outside the day 7 window for clinical reasons, CSF not utilised for clinical purposes and that would otherwise be discarded may be retained for future research purposes. The results from these samples will not be included in the study report.

8.6.4. EVD Sampling

Around 30% of eligible patients will have an External Ventricular Drain (EVD) sited for clinical reasons. This cohort would normally, on clinical grounds, have daily blood samples and regular CSF sampling ranging from daily to twice weekly. In this study, for this cohort of patients approximately 10ml CSF will be taken on alternate days for study purposes (starting on day of EVD fitting) until day 14 or the EVD is removed. Paired blood samples will be taken with every EVD CSF sample. The samples will be used to determine CSF/blood HP & MDA levels (and CSF/blood SFN levels at day 7 requiring a further 10 ml CSF).

Twelve patients may participate in a Pharmacokinetic sub-study, all of whom have been fitted with an EVD as part of normal treatment and prior to randomisation.
The patients will, in addition to all other procedures (with the exception of lumbar puncture), undergo serial CSF sampling (1 sample pre dose and hourly ± 5 minutes for 6 hours post dose) at one of the first three doses and day 7±1 post ictus..

In the case of Serial CSF_{EVD} sampling, approximately 5ml CSF will be removed per time point. The samples will be used to determine CSF SFN levels.

8.6.5. Non-Standard Assays or Procedures

Blood samples (up to a maximum of 25ml in one sample) will be taken for the following purposes:

- Determination of SFN/SFN metabolite, HP and MDA levels

- Proteomic/genomic Evaluation
  - DNA and RNA will be extracted and used for subgroup analysis and evaluation of the effects of SFX-01. These evaluations are for research purposes and may be stored for future genetic testing; the results will not be included in the study report.

9. ADVERSE EVENT REPORTING

All AEs will be reported from the time a signed informed consent form is obtained until 30 days after the last dose of study medication, Adverse events occurring after 30 days following the last dose of study medication must also be reported if considered related to study drug.

9.1. Definitions

Adverse Event:
Any untoward medical occurrence in a patient or clinical investigation subject administered a pharmaceutical product and which does not necessarily have a causal relationship with this treatment.

An adverse event can therefore be any unfavourable and unintended sign (including an abnormal laboratory finding), symptom or disease temporally associated with the use a medicinal (investigational) product, whether or not related to the medicinal (investigational) product.

Adverse Drug Reaction:
Untoward and unintended responses to an investigational medicinal product related to any dose administered.

All adverse events judged by either the reporting Investigator or the Sponsor as having a reasonable causal relationship to a medicinal product qualify are adverse reactions. The expression “reasonable causal relationship” means to convey in general that there are facts or evidence meant to suggest a causal relationship.

Serious Adverse Event (SAE):
Any untoward medical occurrence or effect that at any dose falls in one or more of the following categories:

- Results in death
- Is life-threatening
- Requires hospitalisation or prolongation of existing inpatients’ hospitalisation. Hospitalisation refers to a situation whereby an AE is associated with unplanned overnight admission into hospital.
- Results in persistent or significant disability or incapacity
- Is a congenital anomaly or birth defect
- Is a medically significant adverse event
- adverse events of special interest

9.2. Expectedness

An expected adverse reaction is an adverse reaction, the nature or severity of which is consistent with the applicable product information in the Investigator’s Brochure, otherwise it is considered unexpected.

9.3. Intensity of Adverse Event

Each adverse event must be rated according to the following:

Mild: A type of adverse event that is usually transient and may require only minimal treatment or therapeutic intervention. The event does not generally interfere with usual activities of daily living.

Moderate: A type of adverse event that is usually alleviated with additional specific therapeutic intervention. The event interferes with usual activities of daily living, causing discomfort, but poses no significant or permanent risk of harm to the subject.

Severe: Marked limitation in activity; medical intervention/therapy required, hospitalisation possible

Life-threatening: Extreme limitation in activity, significant medical intervention/therapy required; hospitalisation care possible.

9.4. Causality Assessment

Certain	A clinical event, including laboratory test abnormality, occurring in a plausible time relationship to drug administration, and which cannot be explained by concurrent disease or other drugs or chemicals. The response to withdrawal of the drug (dechallenge) should be clinically plausible. The event must be definitive pharmacologically or phenomenologically, using a satisfactory rechallenge procedure if necessary.
Probable / likely	A clinical event, including laboratory test abnormality, with a reasonable time sequence to administration of the drug, unlikely to be attributed to concurrent disease or other drugs or chemicals, and which follows a clinically reasonable response on withdrawal (dechallenge). Rechallenge information is not required to fulfil this definition.
Possible	A clinical event, including laboratory test abnormality, with a reasonable time sequence to administration of the drug, but which could also be explained by concurrent disease or other drugs or chemicals. Information on drug withdrawal may be lacking or unclear.
Unlikely	A clinical event, including laboratory test abnormality with a temporal relationship to drug administration which makes a causal relationship improbable, and in which other drugs, chemicals or underlying disease provide plausible explanations.
Conditional / unclassified	A clinical event, including laboratory test abnormality, reported as an adverse reaction, about which more data is essential for a proper assessment or the additional data are under examination.

Unassessible / unclassifiable	A report suggesting an adverse reaction which cannot be judged because information is insufficient or contradictory, and which cannot be supplemented.

9.5. Action Taken Regarding the Study Drug

The action taken regarding study drug must be described by selecting one of the following:

- Permanently discontinued
- Stopped temporarily
- Dose reduced
- Dose increased
- No action taken
- Unknown / not applicable

9.6. Outcome

Each AE must be rated by selecting one of the following:

- Recovered / resolved
- Recovering / resolving
- Not recovered / not resolved
- Recovered with sequelae / resolved with sequelae
- Fatal
- Unknown

9.7. Recording Adverse Events

It is the responsibility of the investigator to collect all AEs (both serious and non-serious).

All AEs occurring during the study must be documented on the appropriate section of the case report form (CRF).

If AE is considered serious, it must also be recorded on the Serious Adverse Event Form provided separately.

9.8. Adverse Reaction to SFX-01

Any non-serious adverse events that are deemed to be directly related to the IMP should be reported within 24 hours of awareness.

All Adverse Reactions must be emailed or faxed to Diamond Pharma Services:
Email: PVServices@diamondpharmaservices.com
Fax number: +44 (0)1279 418 964

9.9. Serious Adverse Events

All SAEs must be reported to the CI, CRO and sponsor within 24 hours of awareness, regardless of causal relationship. An SAE form must be filled in by a member of the research team and kept in the TMF.

All SAEs occurring until the end of the trial must be reported by fax immediately by the Investigator or designated assistant is made aware of the event and by full report as soon as possible thereafter.

All SAEs must be emailed or faxed to Diamond Pharma Services Ltd:
Email: PVServices@diamondpharmaservices.com
Fax number: +44 (0)1279 418 964

Where the investigator requires advice regarding the handling of Serious Adverse Events, the contact in case of emergency is:

Diamond Pharma Services Ltd Emergency 24 hour phone number: +44 (0) 1249 406 759

Pregnancies occurring during the study must be reported immediately by fax using the SAE Form.

Diamond Pharma Services will report any SUSARs occurring in the trial to the relevant CA. Evgen Pharma plc will report SUSARs to relevant Research Ethics Committees (REC(s)) and the DSMB as outlined in section 25.

Evgen Pharma plc and Diamond Pharma Services Ltd will keep the Investigator and DSMB (section 25) informed of all SAEs reported to them for the product under investigation, from anywhere in the world, for the duration of the trial at a frequency appropriate to the trial.

In addition, any new safety information that would adversely affect the safety of patients or the conduct of the trial will be reported by Evgen Pharma plc to the CAs, RECs, DSMB and Investigators. If the trial is to be suspended as a result of a SUSAR, or due to any urgent safety measure taken, the CA and REC will be notified as soon as possible and within three days of the decision.

Evgen Pharma plc will submit Safety Reports to the CAs and RECs annually or more frequently if so requested.

10. DATA MANAGEMENT

Data will be recorded on an eCRF by the Investigator (or designee). The database, data entry and electronic checks will be developed using a Clinical Database Management System. Computerised data cleaning checks will be used in addition to manual review to check for discrepancies and to ensure consistency and completeness of the data. An electronic audit trail system will be used to track all data changes in the database.

Data Clarification Forms (DCFs) will be generated in order to clarify any issues which arise during data cleaning. These will be distributed to site for resolving and sign off by the Investigator.

A 100 % quality control check of the data entry will be performed on a randomly selected sample of the eCRFs.

Medical history findings and adverse events will be coded using the MedDRA dictionary; medications will be coded using the World Health Organisation Drug dictionary.

10.1. Trial Documentation and Trial Confidentiality

10.1.1. Trial Documentation, eCRFs and Document Keeping

The Investigator must generate and maintain adequate records (patient medical records, Case Report Forms, source documents) to enable the conduct of this trial to be fully documented. Each patient enrolled into the trial must have an eCRF completed and the eCRF must be reviewed and electronically signed off by the investigator. This applies to those patients who failed to complete the trial (even during the pre-randomisation period). eCRFs are to be completed either at the time of the patient's visit or as soon as possible after the visit so that they always reflect the latest observations on the patients participating in the study. The investigator must electronically verify that all data entries in the eCRFs are accurate and correct. The nature and location of all source documents will be identified to ensure that all sources of original data required to complete the eCRF are known to the company and investigational staff are accessible for verification by the clinical monitor. If electronic records are maintained, the method of verification must be discussed with the investigational staff. A source data verification log will be prepared by TCTC. This will describe the proportion of eCRF data that will be verified by the monitor against the patients' medical records and source data.

The sponsor recommends that the author of an entry in the source documents be identifiable. Direct access to source documentation (medical records) must be allowed for the purpose of verifying that the data recorded on the eCRF are consistent with the original source data.

If data are recorded directly into the eCRF, there should be, at a minimum, an entry in the medical record that each of the assessments was performed; who performed it and the date it was done.

The eCRF will be compared with the source documents to ensure that there are no discrepancies between critical data. All entries, corrections, and alterations are to be made by the responsible investigator or an authorised member of the investigational staff.

Data clarification and query resolution will be conducted on an ongoing basis by the monitor and the contract data management company. Sponsor will have overall responsibility for the data.

The Principal Investigator must be aware of their responsibility to retain patient identification codes in line with regulatory requirements after completion or discontinuation of the trial. If a patient ceases treatment prematurely, then the reason must be noted in the eCRF. If a patient ceases treatment because of an adverse event, reasonable efforts must be made to clearly document the outcome.

The Principal Investigator will allow authorised Sponsor personnel, auditors and regulatory authorities direct access to the patients' medical records.

Copies of protocols, eCRF page/printouts, originals of test results, reports, drug dispensing logs, correspondence, records of informed consent or other documents pertaining to the conduct of the trial must be kept on file by the Investigator in line with regulatory requirements or for the period of time specified by local law for the preservation of hospital patient documents, whichever is the longer. No trial documents should be destroyed without prior written agreement between Sponsor and the Investigator. Where storage at the centre is limited,

the Sponsor may make arrangement for documents to be stored at an independent data archiving facility on behalf of the Principal Investigator. Should the Investigator wish to assign the trial records to another party, or move them to another location, the clinical trial monitor must be consulted.

10.1.2. Confidentiality of Trial Documents and Patient Records

The Investigator must ensure the patients' anonymity is maintained. On CRFs or other documents submitted to TCTC/the Sponsor/third party contractor, patients must NOT be identified by their names, but by an identification code (usually their trial number). The Investigator will be responsible for maintaining a separate log of patients' codes, names and unique identifiers. This log will be maintained as required by applicable regulatory requirements. Documents not for submission to TCTC /the Sponsor/third party contractor, e.g. patients' written consent forms, must be maintained by the Investigator in strict confidence.

11. STATISTICAL ANALYSIS PLAN

A detailed statistical analysis plan (SAP) will be produced after having finalised the protocol and prior to database lock.

11.1. Sample Size

Up to 120 patients may be recruited and enrolled into the trial in order to provide 90 who will meet the per protocol criteria and be analysed for the efficacy analyses.

No formal sample size calculation has been carried out; the Power associated with a sample size of 90 is based on the following assumptions:

- The error probability for the Type-I error should not exceed 5% for a 1-sided test;
- The primary endpoint will be compared between treatment groups by means of a t-test
- The mean maximum MCA flow velocity for patients treated with SFX-01 is estimated as 175 cm/s [31] and
- The standard deviation is set to 50 cm/s

Under these assumptions 90 patients will give 80% power to detect a difference in maximum MCA velocity which is approximately half of the standard deviation of the mean value. The standard deviation was assumed to be approximately 30% of the mean value.

11.1 Populations for analysis

The following populations will be considered for the analysis:

- **Intention-to-Treat population (ITT):** all randomised patients who receive at least one dose of study medication and with any post-dose efficacy evaluations.
  Patients where the time from ictus to admission is unknown are to be considered as part of the ITT population
- **Per-protocol population (PPP):** The Per Protocol Population (for Primary analysis) will be considered to be those patients in the ITT population that have been dosed for a minimum to day 7 post ictus without any major protocol violations (e.g., wrong inclusions, forbidden concomitant medications, etc.). Exact definition of major protocol deviations will be discussed by the clinical team case by case during the Blind Review of the data and described in the Blind Review document. Protocol violations will be considered for each treatment period separately.

- **Safety population:** all randomised patients who have taken at least one dose of study medication.

11.2. Statistical parameters and tests

11.2.1. Primary outcome

Safety

- Concomitant medication
- Adverse events
- Escalation in grading of AE severity
- FBC, U&Es, LFT, CRP & Urine Microscopy
- INR or PT, APTR or APTT, & Fibrinogen (Clauss or Derived) at 7 & 28 days

Pharmacokinetic

- Presence of SFN in CSF

Efficacy

- The maximum MCA flow velocity determined using TCD.
  Treatment groups will be compared using a t-test.

11.2.2. Secondary outcomes

- modified Rankin Scale (mRS), at 7 days, discharge, 28, 90 and 180 days.
  Treatment groups will be compared using a van Elteren test.
- Incidence of Delayed Cerebral Ischaemia (DCI) defined as a new focal deficit or reduction in (Glasgow Coma Scale) GCS ≥2 if not explained by other causes (i.e re-bleed, hydrocephalus, seizure, meningitis, sepsis or hyponatraemia)
  Treatment groups will be compared using a chi-square test.
- Incidence of new cerebral infarct on Computed Tomography (CT) or Magnetic Resonance Imaging (MRI)
  Treatment groups will be compared using a chi-square test.
- Institution of hypertensive (triple H) therapy for presumed DCI
  Treatment groups will be compared using a chi-square test.
- SF-36 quality of life survey at 28, 90 & 180 days.
  Treatment groups will be compared using a t-test.
- Checklist for Cognitive and Emotional Consequences (CLCE-24), Brain Injury Community Rehabilitation Outcomes Scale (BICRO-39), 90 & 180 days.
  Treatment groups will be compared using a van Elteren test.
- Subarachnoid Haemorrhage Outcome Tool (SAHOT) and Glasgow Outcome Scale – Extended (GOSE) at 28, 90 & 180 days.
  Treatment groups will be compared using a van Elteren test.
- Length of acute hospital stay
  Treatment groups will be compared using a Wilcoxon-Mann-Whitney-test.
- Discharge location
  Treatment groups will be compared using a chi-square test.

- Amount of iron identified on MRI Susceptibility Weighted Imaging (SWI) 180 days after start of treatment.
  Treatment groups will be compared using a t-test.
- Cortical atrophy on T1 MRI at 180 days after start of treatment
  Voxel-based morphometry will be used to identify and quantify regional areas of atrophy

Non-EVD Patients: i.e. patients will have a Lumbar Puncture for collection of CSF

- Blood HP and MDA, Proteomic & Genomic concentration at baseline (pre dose 0-48 hours), D7 and D28
- Paired $CSF_{(Lumbar\ Puncture)}$/blood HP, MDA, Proteomic & Genomic & SFN/SFN metabolite concentration at Day 7

EVD Patients: (i.e. will not have a lumbar puncture)

- Blood HP and MDA, Proteomic & Genomic concentration at baseline (pre dose 0-48 hours), D7 and D28
- Paired $CSF_{(EVD)}$/blood HP & MDA, Proteomic & Genomic concentration on alternate days (+/- 1 day) starting on day of EVD fitting until D14 or the EVD removal
- Paired $CSF_{(EVD)}$/blood HP, MDA, Proteomic /Genomic & SFN/SFN metabolite concentration at day 7

Subset of 12 EVD Patients: In addition to all other sampling the following samples will be taken:

- Serial paired $CSF_{(EVD)}$/blood SFN/SFN metabolite concentration at one of the first 3 doses and day 7

Measured PK-variables will be log-transformed, if necessary, and descriptively displayed using Box-plots.

12. ETHICS COMMITTEE / IRB APPROVAL

The study proposal will be submitted to the Ethics Committee in accordance with the national requirements.

The EC shall give its opinion in writing before the clinical trial commences. The investigator should provide written reports to the EC annually or more frequently if requested on any changes significantly affecting the conduct of the trial and / or increasing risk to the subjects.

13. REGULATORY REQUIREMENTS

The study will be authorised by the Medicines and Healthcare products Regulatory Agency (MHRA) in the UK.

Enrolment of subjects will not start until approval has been received from both the Ethics Committee(s) and Competent authorities.

The study will be conducted in accordance with the Declaration of Helsinki, Good Clinical Practice (ICH-GCP) and all other national requirements.

14. INFORMED CONSENT

Due to the nature of SAH it is expected that the majority of participants will be considered to be 'incapacitated adults' at the time of entry into the study where an incapacitated adult is defined as "an adult unable by virtue of physical or mental incapacity to give informed consent".

The hierarchy for consent is considered to be:

1. Patients with Capacity<br>Those patients able to give written informed consent
2. Personal Legal Representative<br>A person not connected with the conduct of the trial who is:<br>(a) Suitable to act as the legal representative by virtue of their relationship with the adult,<br><br>and<br><br>(b) Available and willing to do so
3. Professional legal representative<br>A person not connected with the conduct of the trial who is:<br>(a) The doctor primarily responsible for the adult's medical treatment, or<br><br>(b) A person nominated by the relevant health care provider<br><br>A professional legal representative may be approached if no suitable personal legal representative is available
4. No representative<br>In emergency situations where the treatment to be given to an incapacitated adult as part of the trial needs to be given urgently, time may not allow for the written consent of a legal representative to be obtained first.<br><br>NOTE: Option 4. No representative - Dosing is *only* permissible where local regulations allow

Patients with capacity

Written and verbal versions of the patient information and informed consent form will be presented to the participants detailing the exact nature of the trial, what it will involve for the subject, the implications and constraints of the protocol and the known side effects and risks involved in taking part. It will clearly state that the participant is free to withdraw from the trial at any time for any reason without prejudice to future care and with no obligation to give the reason for withdrawal.

The patients will be allowed as much time as they need in which to decide whether to participate in the trial. The 48 hour timeline for trial medication initiation will not be used to put pressure on the patient to make a decision.

The Investigator should explain to the patient that they are at liberty to refuse entry to the trial or, should they decide to participate, to withdraw from the trial at any time. Such a decision will not, in any way, impinge on their future management.

Written Informed Consent will be obtained by means of participant dated signature with dated signature of the person who presented and obtained the Informed Consent. The person who obtains the consent must be suitably qualified and experienced and have been authorised to do so by the Chief/Principal Investigator. A copy of the signed Informed Consent will be given to the participant and copy will be kept in the patient's notes. The original signed form will be retained in the trial site file (TSF).

Patients lacking capacity with Personal Legal Representative immediately available
Where patients lack capacity and the Personal Legal Representative is immediately available in person an identical approach will be taken substituting the Personal Legal Representative for the patient.

Recording consent of the Personal Legal Representative by telephone is not permitted.

For patients that were unconscious at screening, and informed consent was obtained from their Personal Legal Representative, written informed consent will be obtained and documented from the patient as soon as they regain consciousness sufficiently to do so, respecting their right to withdraw from the study should they wish to.

If the patient has not regained capacity by the time of their 6 month follow up no further attempts will be made to obtain consent.

Patients lacking capacity where Personal Legal Representative is not immediately available
For patients lacking capacity where a Personal Representative is not immediately available in person, a Professional Legal Representative will be sought (providing they are not part of the study team). If they are in attendance in person they will discuss the trial with the research team and complete a consent form if they feel it is appropriate for the subject to participate in the trial.

If they are not in attendance in person they will be contacted by telephone and their opinion sought. If in agreement, the study team will document the verbal consent in the patient notes (details of the representative, date and time of the telephone call, summary of the discussion and Informed Consent process and version of Informed Consent Form), the patient will be enrolled and the Professional Legal Representative will complete a consent form the next time they attend the patient.

For those patients that were unconscious at screening, with informed consent obtained from their Professional Legal Representative, written informed consent will be obtained and documented from the patient as soon as they regain consciousness sufficiently to do so.

For patients lacking capacity and where a Personal Legal Representative was not immediately available, written informed consent will be obtained and documented from the Personal Legal Representative at the earliest opportunity, respecting their right to withdraw the patient from the study should they wish to.

If the patient has not regained capacity by the time of their 6 month follow up no further attempts will be made to obtain consent.

Patients lacking capacity where Professional Legal Representative is not immediately available
For patients lacking capacity where a Personal Representative or Professional Legal Representative is not immediately available (including Professional Legal Representative unavailable by telephone), the study team will discuss potential recruitment of the patient into the study and complete a consent form if they feel it is appropriate for the subject to participate in the trial. The patient will be randomised and receive the first two doses whilst consent is being sought.

Note that this is only permissible where local regulations allow; if local regulations do not allow emergency dosing without consent the patient shall not be enrolled into the study.

In the instance where a patient has been entered into the trial prior to informed consent being obtained (i.e. through the emergency consent procedure) and consent is subsequently refused or not obtained within 24 hours by the patient and/or legal representative, the participant shall be withdrawn and replaced.

The consent of the patient, Personal Legal Representative and Professional Legal Representative will continue to be sought; if consent has not been obtained when the third dose is due the patient shall be withdrawn from the study.

EVD Sub-study
Only Patient consent or that of a Personal Legal Representative (in the case of patients lacking capacity) will be sought prior to any sub-study procedures being carried out.

15. DIRECT ACCESS TO SOURCE DOCUMENTATION / DATA

The investigator must permit trial-related monitoring, audits, Ethics Committee review or regulatory inspection, providing direct access to source data / documents.

16. STUDY MONITORING

It is understood that the study monitor(s) will contact and visit the investigator/clinical site before the study, regularly throughout the study and after the study has been completed. At these visits the monitor(s) will inspect various study records; case report forms, investigator site file and source data, provided that subject confidentiality is respected. The investigator and / or site staff will be expected to be available if requested by the monitor.

17. QUALITY ASSURANCE

The sponsor Evgen Pharma plc may perform an audit at any time according to the sponsor's Standard Operating Procedure, in order to verify whether the study is being conducted according to GCP.

18. TRIAL SCHEDULE

This trial is expected to start in Quarter 2 2016, with all patients recruited and the treatment phase completed Quarter 2 2018. The integrated clinical and statistical report will be completed Quarter 4 2018.

19. INSURANCE

Appropriate insurance cover has been undertaken in favour of patients participating in clinical trials. The cover is provided to the patient on terms and conditions of the clinical trial insurance. Insurance cover exists for health damages as a result of measures carried out in connection with the clinical trial.

20. CONFIDENTIALITY

All study documents are provided by the sponsor in confidence to the investigator and appointed staff. No study material may be disclosed to any party not directly involved in the study without written permission from the sponsor.

The investigator must assure that subject's anonymity will be provided. The investigator will keep a separate list with at least the initials, the subject's study number, names, addresses and telephone numbers. The investigator will maintain this for as long as requested by the sponsor.

21. DATA PROTECTION

Details of access to the patients' data, conforming to the requirements of EU Directive 95/46/EC, will be fully described within the patient information sheet. The consequence of the patients' withdrawal of consent with regards to the use of data will also be described.

22. PREMATURE TERMINATION OF THE STUDY

Both the sponsor and the investigator reserve the right to terminate the study at any time. Should this be necessary, the procedures for an early termination or temporary halt will be arranged after consultation with all parties.

23. RECORD RETENTION

After completion of the study, all documents and data relating to the study will be kept in an orderly manner by the Investigator in a secure study file.

Essential documents must be retained for at least two years after the final marketing approval in an ICH region or until two years have elapsed since the formal interruption of the clinical development of the product under study.

It is the responsibility of the sponsor to inform the investigator of when these documents can be destroyed. The Investigator must contact the sponsor before destroying any trial-related documentation. In addition, all subjects' medical records and other source documentation will be kept for the maximum time permitted by the institution.

24. PUBLICATION OF RESULTS

The sponsor is entitled to publish and/or present any results at scientific meetings, and to submit clinical trial data to national and international Regulatory Authorities. The sponsor reserves the right to use such data for industrial purposes.

Investigators must inform the sponsor before using the results of the study for publication or presentation and agree to provide the sponsor with a copy of the proposed presentation.

25. DATA SAFETY MONITORING BOARD (DSMB)

A committee will be set-up to monitor safety throughout the trial period. The committee will comprise a group of independent experts and will include a chairperson, a specialist neurosurgeon (experienced in SAH) and a statistician all of whom will be independent of the sponsor and will not be involved in the conduct of the trial.

A charter describing how the DSMB works and how it communicates with other study participants (e.g. steering committee) will be prepared.

The DSMB will review unblinded study information which will include:

- List of any protocol violations
- Numbers of patient withdrawals/reason for withdrawal
- Adverse/serious adverse events
- Laboratory data

Following review of the safety data, the committee will prepare written reports (quarterly at a minimum) which will be forwarded to the steering committee advising of any recommendations regarding modifications, continuation or termination of the study.

Where changes in the study conduct are recommended to the steering committee, sufficient (blinded) information will be provided to allow the sponsor to decide whether and how to implement these recommendations.

DSMB Meetings:

The DSMB will convene after 20 patients have been dosed to day 7 post ictus (with adequate safety assessment data) as in-patients in tertiary care for a formal safety review.
The safety review shall make a decision on the acceptability of discharging patients from tertiary care with SFX-01 to complete the dosing course to day 28.
The assessment will provide four possible outcomes:

1. Proceed as planned including allowing continuation of dosing as outpatients from tertiary care
2. Continue with tertiary care inpatient dosing only
3. Proceed after substantial modification of the protocol
4. Discontinue the study

Recruitment will continue throughout the 20 patient DSMB – note that all patients (including those discharged home) complete all the safety assessments.

Data Safety Monitoring Board

The DSMB will convene under the following circumstances:

- The DSMB must meet once the 20th patient has been dosed to day 7 post ictus
- The DSMB must meet as soon as there has been a SUSAR
- The DSMB must meet if 2 patients have a grading change in AE severity (from mild/ moderate to severe or life threatening)
- The DSMB can meet at any point deemed necessary

Study Stopping Rules

The clinical investigation can be placed on hold / stopped early for two reasons and will be based on clinical judgement:

- The DSMB will consider recommending that the study is placed on hold or stopped if the adverse events associated with participation in the study are considered unacceptable.

- The DSMB will consider recommending that the study is placed on hold or stopped if the adverse events associated with SFX-01, in their opinion, significantly outnumber (in frequency or intensity) the adverse events associated with the normal standard of care.

26. STEERING COMMITTEE

The steering committee for the SFX-01 clinical programme will be called the SFX-01 Executive Committee. The executive committee (who will be blinded) will comprise at a minimum, representative(s) of the sponsor (Chief Medical Officer) and the Chief Investigator; the committee will receive and review the reports from the DSMB, and take action as appropriate. This may be a decision to either modify, continue or terminate the study.

27. REFERENCES

1. Van Gijn J, Rinkel GJ. Subarachnoid haemorrhage: diagnosis, causes and management. Brain. 2001; 124: 249-78.
2. Nieuwkamp DJ, Setz LE, Algra A, Linn FH, de Rooij NK, GJ R. Changes in case fatality of aneurysmal subarachnoid haemorrhage over time, according to age, sex, and region: a meta-analysis. Lancet Neurol. 2009;8: 635-42.
3. Kreiter KT, D. C. Predictors of cognitive dysfunction after subarachnoid hemorrhage. Stroke. 2002; 33: 200–8.
4. Pickard JD MG, Illingworth R, Shaw MD, Teasdale GM, Foy PM, Humphrey PR, Lang DA, Nelson R, Richards P, et al. Effect of oral nimodipine on cerebral infarction and outcome after subarachnoid haemorrhage: British aneurysm nimodipine trial. BMJ 1989 11; 298:636-42. 1989.
5. Dorhout Mees SM, Rinkel GJ, Feigin VL, Algra A, van den Bergh WM, Vermeulen M, et al. Calcium antagonists for aneurysmal subarachnoid haemorrhage. Cochrane Database Syst Rev. 2007: CD000277.
6. Hackett ML, CS. A. Health outcomes 1 year after subarachnoid hemorrhage: An international population-based study. The Australian Cooperative Research on Subarachnoid Hemorrhage Study Group. Neurology. 2000; 55: 658-62.
7. David H. Edwards, M.Phil., James V. Byrne, M.D., and Tudor M. Griffith, M.D., Ph.D.. (1992) The effect of chronic subarachnoid hemorrhage on basal endothelium-derived relaxing factor activity in intrathecal cerebral arteries. Journal of Neurosurgery 76:5, 830-837.
8. Leng LZ, Fink ME, C I. Spreading depolarization: a possible new culprit in the delayed cerebral ischemia of subarachnoid hemorrhage. Arch Neurol. 2011;68: 31-6.
9. Al-Tamimi YZ, Orsi NM, Quinn AC, Homer-Vanniasinkam S, Ross SA. A review of delayed ischemic neurologic deficit following aneurysmal subarachnoid hemorrhage: historical overview, current treatment, and pathophysiology. World Neurosurg. 2010; 73: 654-67.
10. Pluta RM, Hansen-Schwartz J, Dreier J, et al. Cerebral vasospasm following subarachnoid hemorrhage: time for a new world of thought. Neurological Research. 2009; 31(2):151–158.
11. Zhao XD, Zhou YT, Lu XJ. Sulforaphane enhances the activity of the Nrf2-ARE pathway and attenuates inflammation in OxyHb-induced rat vascular smooth muscle cells. Inflamm Res. 2013; 62: 857-63.

12. Wang JA, Fields J, Zhao CY, Langer J, Thimmulappa RK, Kensler TW, et al. Role of Nrf2 in protection against intracerebral hemorrhage injury in mice. Free Radic Biol Med. 2007; 43: 408–14.
13. Kong AN, Shen G. Nrf2 Plays an Important Role in Coordinated Regulation of Phase II Drug Metabolize Enzymes and Phase III Drug Transporters. Biopharm Drug Dispos. 2009 Oct; 30(7): 345–355.
14. Itoh K, Chiba T, Takahashi S, Ishii T, Igarashi K, Katoh Y, et al. An Nrf2/small Maf heterodimer mediates the induction of phase II detoxifying enzyme genes through antioxidant response elements. Biochem Biophys Res Commun. 1997 Jul 18; 236(2):313-22.
15. Thimmulappa, R. K., Mai, K. H., Srisuma, S., Kensler, T. W., Yamamoto, M., & Biswal, S. (2002). Identification of Nrf2-regulated genes induced by the chemopreventive agent sulforaphane by oligonucleotide microarray. Cancer Research, 62(18), 5196–5203.
16. Zhao X, Sun G, Zhang J, Strong R, Dash PK, Kan YW, et al. Transcription factor Nrf2 protects the brain from damage produced by intracerebral hemorrhage. Stroke. 2007; 38: 3280-6.
17. Satoh T, Okamoto SI, Cui J, Watanabe Y, Furuta K, Suzuki M, et al. Activation of the Keap1/Nrf2 pathway for neuroprotection by electrophilic phase II inducers. Proc Natl Acad Sci U S A. 2006; 103: 768-73.
18. Li, T., Wang, H., Ding, Y., Zhou, M., Zhou, X., Zhang, X., et al. (2014). Genetic elimination of Nrf2 aggravates secondary complications except for vasospasm after experimental subarachnoid hemorrhage in mice. Brain Research, 1558, 90–99.
19. Chen, G., Fang, Q., Zhang, J., Zhou, D., & Wang, Z. (2011). Role of the Nrf2-ARE pathway in early brain injury after experimental subarachnoid hemorrhage. Journal of Neuroscience Research, 89(4), 515–523.
20. Clarke, J. D., Hsu, A., Williams, D. E., Dashwood, R. H., Stevens, J. F., Yamamoto, M., & Ho, E. (2011). Metabolism and Tissue Distribution of Sulforaphane in Nrf2 Knockout and Wild-Type Mice. Pharmaceutical Research, 28(12), 3171–3179
21. Shapiro, T. A., Fahey, J. W., Dinkova-Kostova, A. T., Holtzclaw, W. D., Stephenson, K. K., Wade, K. L., et al. (2006). Safety, Tolerance, and Metabolism of Broccoli Sprout Glucosinolates and Isothiocyanates: A Clinical Phase I Study. Nutrition and Cancer, 55(1), 53–62.
22. Zhao J, Kobori N, Aronowski J, Dash PK. Sulforaphane reduces infarct volume following focal cerebral ischemia in rodents. Neurosci Lett. 2006; 393:108–112.
23. Neil-Dwyer G, Lang DA, Doshi B, Gerber CJ, Smith PWF. Delayed Cerebral Ischaemia: The Pathological Substrate. Acta Neurochir. 1994; 131: 137-45.
24. Alfieri A., Srivastava S., Siow R.C., Cash D., Modo M., Duchen M.R., Fraser P.A., Williams S.C., Mann G.E. Sulforaphane preconditioning of the Nrf2/HO-1 defense pathway protects the cerebral vasculature against blood-brain barrier disruption and neurological deficits in stroke. Free Radic. Biol. Med. 2013; 65:1012–1022.
25. Galea, J., Cruickshank, G., Teeling, J. L., Boche, D., Garland, P., Perry, V. H., & Galea, I. (2012). The intrathecal CD163-haptoglobin-hemoglobin scavenging system in subarachnoid hemorrhage. Journal of Neurochemistry, 121(5), 785–792.
26. Zhao, X., Song, S., Sun, G., Strong, R., Zhang, J., Grotta, J. C., & Aronowski, J. (2009). Neuroprotective Role of Haptoglobin after Intracerebral Hemorrhage. Journal of Neuroscience, 29(50), 15819–15827.

27. Gasper AV, Al-Janobi A, Smith JA, Bacon JR, Fortun P, Atherton C, et al. Glutathione S-transferase M1 polymorphism and metabolism of sulforaphane from standard and high-glucosinolate broccoli. Am J Clin Nutr. 2005; 82: 1283-91.
28. Zhao, X., Sun, G., Zhang, J., Strong, R., Dash, P. K., Kan, Y. W., et al. (2007). Transcription factor Nrf2 protects the brain from damage produced by intracerebral hemorrhage. Stroke, 38(12), 3280–3286.
29. Reagan-Shaw, S., Nihal, M., & Ahmad, N. (2008). Dose translation from animal to human studies revisited. FASEB Journal: Official Publication of the Federation of American Societies for Experimental Biology, 22(3), 659–661.
30. Sharma, V., & McNeill, J. H. (2009). To scale or not to scale: the principles of dose extrapolation. British Journal of Pharmacology, 157(6), 907–921.
31. Niesen WD, Rosenkranz M, Schummer W, Weiller C, Sliwka U. Cerebral venous flow velocity predicts poor outcome in subarachnoid hemorrhage. Stroke. 2004; 35: 1873-8.

28. APPENDICES

28.1. Appendix 1 – Declaration Of Helsinki - 1996

Recommendations Guiding Medical Physicians in Biomedical Research Involving Human Volunteers

Adopted by the 18th World Medical Assembly, Helsinki, Finland, June 1964, amended by the 29th World Medical Assembly, Tokyo, Japan, October 1975 and the 35th World Medical Assembly, Venice, Italy, October 1983 and revised 41st World Medical Assembly Hong Kong, 1989 and by the 48th World Medical Assembly, South Africa, October 1996

Introduction

It is the mission of the physician to safeguard the health of the people. His or her knowledge and conscience are dedicated to the fulfilment of this mission.

The Declaration of Geneva of the World Medical Association binds the physician with the words, "The health of my patient will be my first consideration", and the International Code of Medical Ethics declares that, "A physician shall act only in the patient's interest when providing medical care which might have the effect of weakening the physical and mental condition of the patient".

The purpose of biomedical research involving human volunteers must be to improve diagnostic, therapeutic and prophylactic procedures and the understanding of the aetiology and pathogenesis of disease.

In current medical practice most diagnostic, therapeutic or prophylactic procedures involve hazards. This applies especially to biomedical research.

Medical progress is based on research which ultimately must rest in part on experimentation involving human volunteers.

In the field of biomedical research a fundamental distinction must be recognised between medical research in which the aim is essentially diagnostic or therapeutic for a patient, and medical research, the essential object of which is purely scientific and without implying direct diagnostic or therapeutic value to the person volunteered to the research.

Special caution must be exercised in the conduct of research which may affect the environment, and the welfare of animals used for research must be respected.

Because it is essential that the results of laboratory experiments be applied to human beings to further scientific knowledge and to help suffering humanity, the World Medical Association has prepared the following recommendations as a guide to every physician in biomedical research involving human volunteers. They should be kept under review in the future.

It must be stressed that the standards as drafted are only a guide to physicians all over the world. Physicians are not relieved from criminal, civil and ethical responsibilities under the laws of their own countries.

I Basic Principles

Biomedical research involving human volunteers must conform to generally accepted scientific principles and should be based on adequately performed laboratory and animal experimentation and on a thorough knowledge of the scientific literature.

The design and performance of each experimental procedure involving human volunteers should be clearly formulated in an experimental protocol which should be transmitted for consideration, comment and guidance to a specially appointed committee independent of the investigator and the sponsor provided that this independent committee is in conformity with the laws and regulations of the country in which the research experiment is performed.

Biomedical research on human volunteers should be conducted only by scientifically qualified persons and under the supervision of a clinically competent medical person. The responsibility for the human volunteer must always rest with a medically qualified person and never rest on the volunteer of the research, even though the volunteer has given his or her consent.

Biomedical research involving human volunteers cannot legitimately be carried out unless the importance of the objective is in proportion to the inherent risk to the volunteer.

Every biomedical research project involving human volunteers should be preceded by careful assessment of predictable risks in comparison with foreseeable benefits to the volunteer or to others. Concern for the interest of the volunteer must always prevail over the interests of science and society.

The right of the research volunteer to safeguard his or her integrity must always be respected. Every precaution should be taken to respect the privacy of the volunteer and to minimise the impact of the study on the volunteer's physical and mental integrity and on the personality of the volunteer.

Physicians should abstain from engaging in research projects involving human volunteers unless they are satisfied that the hazards involved are believed to be predictable. Physicians should cease any investigation if the hazards are found to outweigh the potential benefits.

In publication of the results of his or her research, the physician is obliged to preserve the accuracy of the results. Reports on experimentation not in accordance with the principles laid down in this Declaration should not be accepted for publication.

In any research on human beings, each potential volunteer must be adequately informed of the aims, methods, anticipated benefits and potential hazards of the study and the discomfort it may entail. He or she should be informed that he or she is at liberty to abstain from participation in the study and that he or she is free to withdraw his or her consent to participation at any time. The physician should then obtain the volunteer's freely-given informed consent, preferably in writing.

When obtaining informed consent for the research project the physician should be particularly cautious if the volunteer is in a dependent relationship to him or her or may consent under duress. In that case the informed consent should be obtained by a physician who is not engaged in the investigation and who is completely independent of this official relationship.

In the case of legal incompetence, informed consent should be obtained from the legal guardian in accordance with national legislation. Where physical or mental incapacity makes it impossible to obtain informed consent, or when the volunteer is a minor, permission from the responsible relative replaces that of the volunteer in accordance with national legislation.

Whenever the minor child is in fact able to give consent, the minor's consent must be obtained in addition to the consent of the minor's legal guardian.

The research protocol should always contain a statement of the ethical considerations involved and should indicate that the principles enunciated in the present Declaration are complied with.

II Medical Research Combined With Professional Care (CLINICAL RESEARCH)

In the treatment of the sick person, the physician must be free to use a new diagnostic and therapeutic measure, if in his or her judgement it offers hope of saving life, re-establishing health or alleviating suffering.

The potential benefits, hazards and discomfort of a new method should be weighed against the advantages of the best current diagnostic and therapeutic methods.

In any medical study, every patient - including those of a control group, if any - should be assured of the best proven diagnostic and therapeutic method.

The refusal of the patient to participate in a study must never interfere with the physician-patient relationship.

If the physician considers it essential not to obtain informed consent, the specific reasons for this proposal should be stated in the experimental protocol for transmission to the independent committee.

The physician can combine medical research with professional care, the objective being the acquisition of new medical knowledge, only to the extent that medical research is justified by its potential diagnostic or therapeutic value for the patient

III Non-Therapeutic Biomedical Research Involving Human Volunteers (NON-CLINICAL BIOMEDICAL RESEARCH)

In the purely scientific application of medical research carried out on a human being, it is the duty of the physician to remain the protector of the life and health of that person on whom biomedical research is being carried out.

The volunteer should be volunteers - either healthy persons or patients for whom the experimental design is not related to the patient's illness.

The investigator or the investigating team should discontinue the research if in his/her or their judgement it may, if continued, be harmful to the individual.

In research on man, the interest of science and society should never take precedence over considerations related to the well-being of the volunteer.
