# Supplementary material for: A Randomised Controlled Trial of SFX-01 After Subarachnoid Haemorrhage — The SAS Study
Source: Transl Stroke Res. 2024 Jul 19;16(4):1031–43. doi: 10.1007/s12975-024-01278-1 (PMC12202693; doi:10.1007/s12975-024-01278-1)
Supplement: Supplementary file 3 — Supplementary file3 - Validation report (PDF 6174 KB) [file 12975_2024_1278_MOESM3_ESM.pdf]

The Validation of an Analytical Procedure for the Determination of Sulforaphane (SFN), Sulforaphane N-acetyl Cysteine (SFN-NAC) and Sulforaphane Glutathione (SFN-GSH) in Human Cerebrospinal Fluid (CSF) by LC-MS/MS, using Artificial CSF as a Surrogate Matrix

Study Sponsor	Evgen Ltd.<br>146 Brownlow Hill<br>Liverpool<br>L3 5RF
Bioanalytical Test Site	Alderley Analytical<br>BioHub at Alderley Park<br>Alderley Edge<br>Cheshire<br>SK10 4TG
Bioanalytical Project Leader	Alan Gibbs<br>alan.gibbs@alderleyanalytical.com<br>Telephone : +44 (0)1625 238610
Bioanalytical Method Title	Method for the Determination of SFN, SFN N-acetyl Cysteine and SFN Glutathione in CSF by LC-MS/MS
Alderley Analytical Study Number	0014/003
Alderley Analytical Method Number	0001/023
Species/Matrix/Stabiliser	Human / CSF / 0.5M Citric Acid
Stabiliser Concentration	300 µL of 0.5M citric acid per 17.7 mL of CSF
Surrogate Matrix	Artificial stabilised CSF
Validation Study Plan Client Issue Date	08 March 2016
Experimental Start Date	11 April 2016
Experimental Completion Date	10 May 2016
Report Issue Date	08 February 2017
Version	Final

MANAGEMENT STATEMENT

The bioanalytical validation report entitled "The Validation of an Analytical Procedure for the Determination of Sulforaphane (SFN), Sulforaphane N-acetyl Cysteine (SFN-NAC) and Sulforaphane Glutathione (SFN-GSH) in Human Cerebrospinal Fluid (CSF) by LC-MS/MS, using Artificial CSF as a Surrogate Matrix" has been reviewed and authorised by a member of the Alderley Analytical management team.

Elizabeth Thomas 08 FEB 2017

Elizabeth Thomas
CEO

Date

COMPLIANCE STATEMENT

Study Number: 0014/003

Study Title: The Validation of an Analytical Procedure for the Determination of Sulforaphane (SFN), Sulforaphane N-acetyl Cysteine (SFN-NAC) and Sulforaphane Glutathione (SFN-GSH) in Human Cerebrospinal Fluid (CSF) by LC-MS/MS, using Artificial CSF as a Surrogate Matrix

No formal claim of GLP compliance is required for work of this type, and no claim of compliance will be made for this validation.

However, all work carried out in this study was conducted in a Good Laboratory Practice (GLP) accredited laboratory in accordance with the OECD guidelines for GLP as incorporated into the United Kingdom statutory instrument for GLP 1999 No. 3106, as amended by statutory instrument 2004 No. 994.

All work was carried out in accordance with the Standard Operating Procedures of Alderley Analytical.

Alan Gibbs
Project Leader

08 Feb 2017
Date

QUALITY ASSURANCE STATEMENT

Study Number: 0014/003

Study Title: The Validation of an Analytical Procedure for the Determination of Sulforaphane (SFN), Sulforaphane N-acetyl Cysteine (SFN-NAC) and Sulforaphane Glutathione (SFN-GSH) in Human Cerebrospinal Fluid (CSF) by LC-MS/MS, using Artificial CSF as a Surrogate Matrix

Alderley Analytical QA has reviewed this report. The report is considered to accurately describe the methods and procedures used in the study and to accurately reflect the raw data of the study.

Inspections of this study were carried out on the following dates. Findings were reported to the Project Leader and to Management.

Object of Inspection	Inspection No.	Date of Inspection	Date reported to Project Leader and Management
Final Report Audit	QAAT014	06-19 Oct 2016	19 Oct 2016

Facilities relevant to this type of study are audited on an annual basis. Findings are reported to Management.

J Pearse.

Jean Pearson
QA Representative

08 February 2017.

Date

TABLE OF CONTENTS

Page Number

MANAGEMENT STATEMENT **2**
COMPLIANCE STATEMENT **3**
QUALITY ASSURANCE STATEMENT **4**
TABLE OF CONTENTS **4**
LIST OF TABLES **5**
LIST OF FIGURES **7**
1 SUMMARY **8**
2 VALIDATION RESULTS **13**
Selectivity 13
Linear Range and Response Function 13
Precision and Accuracy 13
Sensitivity 15
Evaluation of Large Run Size 15
Dilution Integrity 15
Recovery (Extraction Efficiency) 15
Matrix Effects 16
Evaluation of Artificial CSF + 0.1% Blood 17
Evaluation of Human CSF 18
Carryover Evaluation 18
Stability 18
3 DEVIATIONS **22**
4 CONCLUSIONS **23**
5 ARCHIVE PROCEDURE **23**
6 REFERENCES **23**
7 TABLES **24**
8 FIGURES **68**
9 AMENDMENT HISTORY **81**
10 APPENDICES **82**
Appendix 1 Bioanalytical Method 82
Appendix 2 Certificates of Analysis 93
Appendix 3 Validation Plan 98
Appendix 4 Stock Stability 114

LIST OF TABLES

Table 1a Selectivity for SFN 24
Table 1b Selectivity for SFN Internal Standard 24
Table 1c Selectivity for SFN-NAC 25
Table 1d Selectivity for SFN-NAC Internal Standard 25
Table 1e Selectivity for SFN-GSH 26
Table 1f Selectivity for SFN-GSH Internal Standard 26
Table 2a Standard Curve Parameters for SFN 27
Table 2b Standard Curve Parameters for SFN-NAC 28
Table 2c Standard Curve Parameters for SFN-GSH 29
Table 3a Standard Curve Back Calculated Concentrations of SFN 30
Table 3b Standard Curve Back Calculated Concentrations of SFN-NAC 31
Table 3c Standard Curve Back Calculated Concentrations of SFN-GSH 32
Table 4a Intra-Run Quality Control Sample Concentrations of SFN 33
Table 4b Intra-Run Quality Control Sample Concentrations of SFN-NAC 34

Table 4c Intra-Run Quality Control Sample Concentrations of SFN-GSH 35
Table 4d Intra-Run Quality Control Sample Concentrations of SFN-GSH (outliers removed) 36
Table 4e Inter-Run Quality Control Sample Concentrations of SFN 37
Table 4f Inter-Run Quality Control Sample Concentrations of SFN-NAC 37
Table 4g Inter-Run Quality Control Sample Concentrations of SFN-GSH 38
Table 4h Inter-Run Quality Control Sample Concentrations of SFN-GSH (outliers removed) 38
Table 5a Upper Limit of Quantitation for SFN 39
Table 5b Upper Limit of Quantitation for SFN-NAC 39
Table 5c Upper Limit of Quantitation for SFN-GSH 40
Table 6a Dilution Integrity (10-fold) for SFN 41
Table 6b Dilution Integrity (10-fold) for SFN-NAC 41
Table 6c Dilution Integrity (10-fold) for SFN-GSH 42
Table 7a Recovery (Extraction Efficiency) for SFN 43
Table 7b Recovery (Extraction Efficiency) for SFN-NAC 43
Table 7c Recovery (Extraction Efficiency) for SFN-GSH 43
Table 8a Recovery (Extraction Efficiency) for SFN Internal Standard 44
Table 8b Recovery (Extraction Efficiency) for SFN-NAC Internal Standard 44
Table 8c Recovery (Extraction Efficiency) for SFN-GSH Internal Standard 45
Table 9a Matrix Effects at 15 ng/mL of SFN (Low QC) 46
Table 9b Matrix Effects at 1600 ng/mL of SFN (High QC) 46
Table 9c Matrix Effects at 15 ng/mL of SFN-NAC (Low QC) 47
Table 9d Matrix Effects at 1600 ng/mL of SFN-NAC (High QC) 47
Table 9e Matrix Effects at 30 ng/mL of SFN-GSH (Low QC) 48
Table 9f Matrix Effects at 1600 ng/mL of SFN-GSH (High QC) 48
Table 10a Normalised Matrix Effects at 15 ng/mL of SFN (Low QC) 49
Table 10b Normalised Matrix Effects at 1600 ng/mL of SFN (High QC) 49
Table 10c Normalised Matrix Effects at 15 ng/mL of SFN-NAC (Low QC) 50
Table 10d Normalised Matrix Effects at 1600 ng/mL of SFN-NAC (High QC) 50
Table 10e Normalised Matrix Effects at 30 ng/mL of SFN-GSH (Low QC) 51
Table 10f Normalised Matrix Effects at 1600 ng/mL of SFN-GSH (High QC) 51
Table 11a SFN in aCSF + 0.1% Whole Blood 52
Table 11b SFN-NAC in aCSF + 0.1% Whole Blood 52
Table 11c SFN-GSH in aCSF + 0.1% Whole Blood 53
Table 12a SFN in hCSF 54
Table 12b SFN-NAC in hCSF 54
Table 12c SFN-GSH in hCSF 55
Table 13a Carryover Assessment for SFN 56
Table 13b Carryover Assessment for SFN-NAC 56
Table 13c Carryover Assessment for SFN-GSH 56
Table 14a Carryover Assessment for SFN IS 57
Table 14b Carryover Assessment for SFN-NAC IS 57
Table 14c Carryover Assessment for SFN-GSH IS 57
Table 15a 24 Hour Re-Injection Reproducibility and Autosampler Stability for SFN 58
Table 15b 24 Hour Re-Injection Reproducibility and Autosampler Stability for SFN-NAC 59
Table 15c 24 Hour Re-Injection Reproducibility and Autosampler Stability for SFN-GSH 60
Table 16a 2.5 Hour Benchtop Stability for SFN in aCSF 61
Table 16b 2.5 Hour Benchtop Stability for SFN-NAC in aCSF 61
Table 16c 2.5 Hour Benchtop Stability for SFN-GSH in aCSF 61
Table 17a 2.5 Hour Benchtop Stability for SFN in hCSF 62
Table 17b 2.5 Hour Benchtop Stability for SFN-NAC in hCSF 62
Table 17c 2.5 Hour Benchtop Stability for SFN-GSH in hCSF 62
Table 18a 2-Cycle Freeze (-80°C) /Thaw Stability for SFN in aCSF 63
Table 18b 2-Cycle Freeze (-80°C) /Thaw Stability for SFN-NAC in aCSF 63

Table 18c 2-Cycle Freeze (-80°C) /Thaw Stability for SFN-GSH in aCSF ... 63
Table 19a 2-Cycle Freeze (-80°C) /Thaw Stability for SFN in hCSF ... 64
Table 19b 2-Cycle Freeze (-80°C) /Thaw Stability for SFN-NAC in hCSF ... 64
Table 19c 2-Cycle Freeze (-80°C) /Thaw Stability for SFN-GSH in hCSF ... 64
Table 20a Interference Screens Relative to SFN ... 65
Table 20b Interference Screens Relative to SFN-NAC ... 66
Table 20c Interference Screens Relative to SFN-GSH ... 67

LIST OF FIGURES

Figure 1a Standard Curve of SFN in Artificial Cerebrospinal Fluid, P&A Run 1 ... 68
Figure 1b Standard Curve of SFN in Artificial Cerebrospinal Fluid, P&A Run 2 ... 69
Figure 1c Standard Curve of SFN in Artificial Cerebrospinal Fluid, P&A Run 3 ... 70
Figure 2a Standard Curve of SFN-NAC in Artificial Cerebrospinal Fluid, P&A Run 1 ... 71
Figure 2b Standard Curve of SFN-NAC in Artificial Cerebrospinal Fluid, P&A Run 2 ... 72
Figure 2c Standard Curve of SFN-NAC in Artificial Cerebrospinal Fluid, P&A Run 3 ... 73
Figure 3a Standard Curve of SFN-GSH in Artificial Cerebrospinal Fluid, P&A Run 1 ... 74
Figure 3b Standard Curve of SFN-GSH in Artificial Cerebrospinal Fluid, P&A Run 2 ... 75
Figure 3c Standard Curve of SFN-GSH in Artificial Cerebrospinal Fluid, P&A Run 3 ... 76
Figure 4 Representative Chromatogram of a Human Cerebrospinal Fluid Blank ... 77
Figure 5 Representative Chromatogram of an LLOQ Calibration Standard for SFN and SFN-NAC in Artificial Cerebrospinal Fluid ... 78
Figure 6 Representative Chromatogram of an LLOQ Calibration Standard for SFN-GSH in Artificial Cerebrospinal Fluid ... 79
Figure 7 Representative Chromatogram of a ULOQ Calibration Standard for SFN, SFN-NAC and SFN-GSH in Artificial Cerebrospinal Fluid ... 80

1 SUMMARY

The objective of this study was to implement and validate a bioanalytical method for the quantitative analysis of SFN, SFN-NAC and SFN-GSH in Human Cerebrospinal Fluid.

Alderley Analytical has developed an LC-MS/MS assay for the measurement of SFN, SFN-NAC and SFN-GSH in human CSF (hCSF) samples, using artificial CSF (aCSF) as a surrogate matrix. This report details the accuracy and reproducibility of data obtained during the validation of the method.

The CSF standards and samples were extracted by a solid phase extraction method and analysed using a Waters I-Class UPLC, coupled to a Waters TQ-S Mass Spectrometer. UNIFI software (version number 1.7.1.0.0) was used to quantify peaks. Quantification was achieved using analyte peak area to internal standard (SFN-d_8 used as IS for SFN, SFN-NAC-d_8 used as IS for SFN-NAC and SFN-GSH) peak area ratios. Concentrations of the calibration curve standards, quality control samples and study samples were determined by the method of ($1/x^2$) weighted least squares linear regression.

The bioanalytical method (Alderley Analytical Method 0001/023) for the determination of SFN, SFN-NAC and SFN-GSH concentrations in CSF over ranges of 5 to 2000 ng/mL for SFN and SFN-NAC, and 10 to 2000 ng/mL for SFN-GSH, using a sample volume of 100 µL, can be found in Appendix 1.

The Certificates of Analysis for each compound used in the analysis are provided in Appendix 2.

The LC-MS/MS method for the determination of SFN, SFN-NAC and SFN-GSH concentrations in CSF was validated according to Alderley Analytical SOP L001 (Ref. 1), and Validation Plan Number 0014/003 (Appendix 3) for the given concentration ranges. The method validation results are summarised below.

**Method Description**	
Analyte	SFN, SFN-NAC, SFN-GSH
Matrix	Human Cerebrospinal Fluid, using Artificial Cerebrospinal Fluid as a surrogate matrix
Stabiliser	300 µL of 0.5M citric acid per 17.7 mL of CSF
Extraction Method	Solid Phase Extraction
Detection Method	LC-MS/MS
Sample Aliquot Volume	100 µL
Regression, Weighting	Linear, $1/x^2$
Quantification	Peak Area Ratios
Calibration Range	5 to 2000 ng/mL – SFN and SFN-NAC<br>10 to 2000 ng/mL – SFN-GSH

QC Levels	5, 15, 800, 1600 and 8000 ng/mL – SFN and SFN-NAC<br>10, 30, 800, 1600 and 8000 ng/mL – SFN-GSH		
**Assay Performance**			
Selectivity Blanks	SFN: ≤12.87%<br>SFN IS: all 0.00%<br>SFN-NAC: all 0.00%<br>SFN-NAC IS: ≤3.58%<br>SFN-GSH: ≤3.46%<br>SFN-GSH IS: ≤3.49%		
Overall Precision and Accuracy		Precision (%CV)	Accuracy (%RE)
SFN	LLOQ:	10.24	94
	Low QC:	6.42	91
	Mid QC:	3.23	93
	High QC:	5.48	93
	ULOQ:	1.70	100
SFN-NAC	LLOQ:	13.53	103
	Low QC:	6.80	99
	Mid QC:	4.43	99
	High QC:	7.51	97
	ULOQ:	1.17	100
SFN-GSH	LLOQ:	10.03	107
	Low QC:	13.16	102
	Mid QC:	9.67	99
	High QC:	5.39	102
	ULOQ:	3.25	115
Dilution Integrity	SFN 10-fold: Precision 3.91%, Accuracy 95%<br>SFN-NAC 10-fold: Precision 4.48%, Accuracy 93%<br>SFN-GSH 10-fold: Precision 5.30%, Accuracy 91%		
Recovery	SFN:	95-102%	
	SFN-NAC:	56-77%	
	SFN-GSH:	27-41%	

	SFN IS:	91%
	SFN-NAC IS:	65%
	SFN-GSH IS:	65%
Matrix Effect Factor %CV	20.04% & 11.18% (SFN), 10.91% & 4.64% (SFN-NAC), 9.15% & 4.52% (SFN-GSH)	
Normalised Matrix Effect Factor %CV	5.50% & 6.67% (SFN), 7.54% & 4.24% (SFN-NAC), 5.75% & 2.69% (SFN-GSH)	
Effect of aCSF + 0.1% Blood	SFN:	Precision: 8.32% & 2.60%
		Accuracy: 106% & 102%
	SFN-NAC:	Precision: 4.73% & 1.60%
		Accuracy: 104% & 97%
	SFN-GSH:	Precision: 4.50% & 2.18%
		Accuracy: 88% & 106%
Effect of hCSF	SFN:	Precision: 4.86% & 2.68%
		Accuracy: 123% & 111%
	SFN-NAC:	Precision: 2.92% & 3.17%
		Accuracy: 90% & 91%
	SFN-GSH:	Precision: 5.16% & 4.84%
		Accuracy: 92% & 108%
Carryover	SFN:	0.00% to 17.47%
	SFN IS:	0.00%
	SFN-NAC:	0.00%
	SFN-NAC IS:	0.00%
	SFN-GSH:	0.00% to 7.17%
	SFN-GSH IS:	0.00%
Solution Stability (nominal 4^oC)	SFN : 67 days	102%
	SFN-NAC : 69 days	96%
	SFN-GSH : 67 days	112%

Re-injection Reproducibility against original curve for partial batch re-injection	SFN Precision:	2.08% to 2.79%
	SFN Accuracy:	98% to 101%
	SFN-NAC Precision:	1.45% to 4.04%
	SFN-NAC Accuracy:	101% to 105%
	SFN-GSH Precision:	3.97% to 6.92%
	SFN-GSH Accuracy:	97% to 105%
Re-injection Reproducibility against re-injected curve for full batch re-injection	SFN Precision:	2.08% to 2.90%
	SFN Accuracy:	95% to 96%
	SFN-NAC Precision:	1.45% to 3.95%
	SFN-NAC Accuracy:	107% to 109%
	SFN-GSH Precision:	3.97% to 6.95%
	SFN-GSH Accuracy:	100% to 109%
Bench Top Stability on wet ice (aCSF)	SFN over 2.5 hours: Precision 1.39% to 6.76%, Accuracy 92% to 103%	
	SFN-NAC over 2.5 hours: Precision 1.36% to 5.15%, Accuracy 95% to 103%	
	SFN-GSH over 2.5 hours: Precision 0.94% to 6.70%, Accuracy 96% to 109%	
Bench Top Stability on wet ice (hCSF)	SFN over 2.5 hours: Precision 1.74% to 2.83%, Accuracy 112% to 116%	
	SFN-NAC over 2.5 hours: Precision 3.53% to 3.96%, Accuracy 84% to 100%	
	SFN-GSH over 2.5 hours: Precision 3.65% to 6.19%, Accuracy 84% to 94%	
Freeze/Thaw Stability at -80°C/RT (aCSF)	SFN over 2 cycles: Precision 0.26% to 3.96%, Accuracy 91% to 98%	
	SFN-NAC over 2 cycles: Precision 1.21% to 3.02%, Accuracy 95% to 102%	
	SFN-GSH over 2 cycles: Precision 1.33% to 2.59%, Accuracy 97% to 119%	

Freeze/Thaw Stability at -80ºC/RT (hCSF)	SFN over 2 cycles: Precision 3.34% to 4.07%, Accuracy 108% to 109%
	SFN-NAC over 2 cycles: Precision 1.11% to 8.59%, Accuracy 85% to 96%
	SFN-GSH over 2 cycles: Precision 1.13% to 4.90%, Accuracy 94% to 118%
Interference Screens	Maximum contribution: 12.69% (SFN not included in data as metabolites known to contain SFN)
Relative Retention Acceptance Range	acceptable

2 VALIDATION RESULTS

Selectivity

During Run 8, blank extracts generated from six individual lots of blank matrix were analysed. They were labelled as follows;

Matrix 1 = pooled human CSF (hCSF)
Matrix 2-4 = three lots of individual human CSF
Matrix 5 = individual human CSF + approximately 0.1% whole blood
Matrix 6 = stabilised artificial CSF (aCSF)

No significant interfering peaks (>20% of the lower limit of quantitation response) were observed at the retention time of SFN, SFN-NAC and SFN-GSH. In addition, no significant interfering peaks (>5% of mean internal standard response) were observed at the retention time of the internal standards. These results indicate that selectivity has been demonstrated for this assay in CSF (Tables 1a to 1f).

Linear Range and Response Function

A line was fitted through the data points of calibration standards prepared in aCSF by weighted linear regression (weight = $1/x^2$) for SFN, SFN-NAC and SFN-GSH of the concentration (x-axis) vs. peak area ratio (y-axis). SFN and SFN-NAC have a range of 5 to 2000 ng/mL, and SFN-GSH has a range of 10 to 2000 ng/mL (Figures 1a to 3c). Correlation coefficients for all calibration curves were at least 0.982 for SFN, SFN-NAC and SFN-GSH (Tables 2a to 2c).

Concentrations of the calibration standards were back-calculated and results are provided in Tables 3a to 3c. A calibration curve is acceptable provided that 75% of the back-calculated values for the standards analysed in the run do not deviate by more than ±15% at all concentrations, except at the LLOQ level where values can deviate up to ±20% of the nominal value. At least six concentration levels (including at least one replicate each of the LLOQ and ULOQ) must remain in the curve. All acceptance criteria were met.

Precision and Accuracy

Inter-run and intra-run precision and accuracy were determined by analysing four concentrations of QC samples prepared in aCSF in replicates of six over three separate batch runs on three different days.

- 5, 15, 800, and 1600 ng/mL for SFN and SFN-NAC
- 10, 30, 800, and 1600 ng/mL for SFN-GSH

For acceptance of the low, mid, and high QC levels, the precision around the mean value must not exceed 15% and the accuracy must be within ±15% of the nominal value. For acceptance at the LLOQ QC level, the precision around the mean value must not exceed 20% and the accuracy must be within ±20% of the nominal value.

Precision of the method, defined by the percent coefficient of variation (%CV) [standard deviation / mean × 100], was determined from the interpolated QC sample concentrations.

The intra-run precision for the QC samples at the LLOQ, low, mid, and high QC levels over the three precision and accuracy batch runs were all within batch acceptance criteria (Tables 4a to 4d).

The overall inter-run precision for the QC samples at the LLOQ, low, mid, and high QC levels over the three precision and accuracy batch runs was 10.24%, 6.42%, 3.23% and 5.48% respectively for SFN (Table 4e), 13.53%, 6.80%, 4.43% and 7.51% respectively for SFN-NAC (Table 4f), and 10.03%, 13.16%, 9.67% and 5.39% respectively for SFN-GSH (Table 4g including outliers, Table 4h with outliers removed).

The acceptance criteria for precision were met.

Accuracy of the method was defined by the percent relative error (%RE) [(mean observed concentration - nominal concentration) / nominal concentration × 100].

The intra-run accuracy for the QC samples at the LLOQ, low, mid, and high QC levels over the three precision and accuracy batch runs were all within batch acceptance criteria (Tables 4a to 4d) other than one level of one batch for SFN-GSH only (see section 3, point 1). Two results for SFN-GSH were removed from the calculations; these sample results were >1 SD from the appropriate intra-day mean value and >3 SD from the nominal concentrations, so are therefore considered to be statistical outliers.

The overall inter-run accuracy for the QC samples at the LLOQ, low, mid, and high QC levels over the three precision and accuracy batch runs was 94%, 91%, 93% and 93% respectively for SFN (Table 4e), 103%, 99%, 99% and 97% respectively for SFN-NAC (Table 4f), and 107%, 102%, 99% and 102% respectively for SFN-GSH (Table 4g including outliers, Table 4h with outliers removed).

The acceptance criteria for accuracy were met.

The upper limit of quantitation (ULOQ) of the assay was defined as the highest calibration standard concentration. Samples prepared at a concentration of 2000 ng/mL were analysed in replicates of six in Run 11 to yield precision and accuracy results of 1.70% and 100% respectively for SFN (Table 5a), 1.17% and 100% respectively for SFN-NAC (Table 5b) and 3.25% and 115% respectively for SFN-GSH (Table 5c).

The precision and accuracy are calculated from all replicates of the ULOQ sample. For acceptance, the precision around the mean value must not exceed 15% and the accuracy must be within ±15% of the nominal value.

The acceptance criteria for precision and accuracy were met.

Sensitivity

The lower limit of quantitation (LLOQ) of the assay was defined as the lowest calibration standard concentration. Samples prepared at a concentration of 5 ng/mL (for SFN and SFN-NAC) or 10 ng/mL (for SFN-GSH) were analysed in duplicate on each of three batch runs. The signal-to-noise ratio for each LLOQ standard was ≥5:1, and therefore met the acceptance criteria.

Evaluation of Large Run Size

Evaluation of Large Run Size was not performed during the validation (see section 3, point 2).

Dilution Integrity

QC samples prepared in aCSF containing 8000 ng/mL of SFN, SFN-NAC AND SFN-GSH were diluted 10-fold with blank matrix, extracted, and analysed in Run 9. A correction factor of 10 was applied to the concentration results of samples diluted 10-fold to yield precision and accuracy results of 3.91% and 95% respectively for SFN (Table 6a), 4.48% and 93% respectively for SFN-NAC (Table 6b) and 5.30% and 91% respectively for SFN-GSH (Table 6c).

The precision and accuracy are calculated from all replicates of the diluted QC sample. For acceptance, the precision around the mean value must not exceed 15% and the accuracy must be within ±15% of the nominal value.

The acceptance criteria for precision and accuracy were met. These results indicate that a sample with an analyte concentration higher than the upper limit of the calibration curve can be diluted 10-fold successfully.

Recovery (Extraction Efficiency)

Human Cerebrospinal Fluid samples at three concentrations in replicates of three were extracted and injected onto the LC-MS/MS system (*extracted* samples) in Run 13.

- 15, 800 and 1600 ng/mL for SFN and SFN-NAC
- 30, 800 and 1600 ng/mL for SFN-GSH

Solutions of SFN, SFN-NAC AND SFN-GSH spiked into extracted matrix blanks at concentrations representing 100% extraction recovery were also analysed by LC-MS/MS in replicates of three (*post-*

spiked samples). The comparison of the individual peak areas of extracted samples to the mean peak area of the post-spiked samples provided the extraction recovery results. The mean extraction recovery values ranged from 95% to 102% for SFN, from 56% to 77% for SFN-NAC and from 27% to 41% for SFN-GSH (Tables 7a to 7c).

The same experiment was used to determine the extraction recovery of the internal standard at the working concentration (50 ng/mL for internal standard in matrix) used for the assay. The overall mean extraction recovery was 91% for SFN IS, 65% for SFN-NAC IS and 65% for SFN-GSH IS (Tables 8a to 8c). Note that whilst SFN-NAC and SFN-GSH use the same internal standard, different values are used to calculate the results due to different Low QC2 samples being used.

Acceptance limits are not placed on recovery. The recovery of analytes and internal standards need not be 100%, but should be consistent across the concentration range. The recovery values obtained were sufficiently consistent across the concentration range to be considered acceptable (see section 3, point 3).

Matrix Effects

During Run 13, possible matrix effects were assessed in six individual lots of blank matrix. Each of the six lots of blank matrix (coding relating to blank matrices was as per the Selectivity section) were extracted in triplicate and then spiked with analyte and internal standard at concentrations equivalent to extracted low and high QC level samples (*spiked* samples with matrix present). Additionally, analytical solutions containing analyte and internal standard were prepared in triplicate in reconstitution solution, also at concentrations equivalent to extracted low and high QC samples (analytical solutions, no matrix present).

The matrix effect was calculated for each blank matrix lot using the Matrix Factor equation below:

Matrix Factor = (mean peak area in presence of matrix ions) / (mean peak area in absence of matrix ions)

A Matrix Factor (MF) of one indicates no matrix effect. An MF of less than one may indicate matrix suppression and an MF greater than one may indicate enhancement. An MF of one is not necessary, however the overall precision (%CV) of the Matrix Factors across all six lots should be ≤15%. If overall precision of the Matrix Factors is not within this limit, then the assessment is still acceptable, provided that the overall precision of the Normalized (internal standard adjusted) Matrix Factors across all six lots is ≤15%. The normalized matrix factor is calculated for each lot using the equation below:

Normalized Matrix Factor = (mean peak area ratio in presence of matrix ions) / (mean peak area ratio in absence of matrix ions)

The precision of the Matrix Factors for SFN at the low QC level (15 ng/mL) over the six lots of matrix was 20.04%, and the precision of the normalized matrix factors over the same six lots of matrix was 5.50% (Tables 9a and 10a), thus meeting the acceptance criteria of ≤15%. The precision of the Matrix Factors for SFN at the high QC level (1600 ng/mL) over the six lots of matrix was 11.18%, and the precision of the normalized matrix factors over the same six lots of matrix was 6.67% (Tables 9b and 10b), thus meeting the acceptance criteria of ≤15%.

The precision of the Matrix Factors for SFN-NAC at the low QC level (15 ng/mL) over the six lots of matrix was 10.91%, and the precision of the normalized matrix factors over the same six lots of matrix was 7.54% (Tables 9c and 10c), thus meeting the acceptance criteria of ≤15%. The precision of the Matrix Factors for SFN-NAC at the high QC level (1600 ng/mL) over the six lots of matrix was 4.64%, and the precision of the normalized matrix factors over the same six lots of matrix was 4.24% (Tables 9d and 10d), thus meeting the acceptance criteria of ≤15%.

The precision of the Matrix Factors for SFN-GSH at the low QC level (30 ng/mL) over the six lots of matrix was 9.15%, and the precision of the normalized matrix factors over the same six lots of matrix was 5.75% (Tables 9e and 10e), thus meeting the acceptance criteria of ≤15%. The precision of the Matrix Factors for SFN-GSH at the high QC level (1600 ng/mL) over the six lots of matrix was 4.52%, and the precision of the normalized matrix factors over the same six lots of matrix was 2.69% (Tables 9f and 10f), thus meeting the acceptance criteria of ≤15%.

Evaluation of Artificial CSF + 0.1% Blood

The effect resulting from the presence of blood in a CSF sample was assessed by preparing QC samples at low and high concentrations into artificial stabilised CSF containing 0.1% whole blood.

Six replicates of each aCSF + 0.1% blood sample, stored at the requisite temperature for at least 24 hours prior to use, were analysed along with a standard curve and run acceptance QCs in at least duplicate, prepared in artificial stabilised CSF.

For SFN, the precision and accuracy over the six lots of aCSF + 0.1% blood were 8.32% and 106% respectively at the low QC level, and 2.60% and 102% respectively at the high QC level (Table 11a), thus meeting the acceptance criteria of ≤15%.

For SFN-NAC, the precision and accuracy over the six lots of aCSF + 0.1% blood were 4.73% and 104% respectively at the low QC level, and 1.60% and 97% respectively at the high QC level (Table 11b), thus meeting the acceptance criteria of ≤15%.

For SFN-GSH, the precision and accuracy over the six lots of aCSF + 0.1% blood were 4.50% and 88% respectively at the low QC level, and 2.18% and 106% respectively at the high QC level (Table 11c), thus meeting the acceptance criteria of ≤15%.

Evaluation of Human CSF

The effect resulting from the use of human CSF was assessed by preparing QC samples at low and high concentrations into human CSF.

Six replicates of each hCSF sample, stored at the requisite temperature for at least 24 hours prior to use, were analysed along with a standard curve and run acceptance QCs in at least duplicate, prepared in artificial stabilised CSF.

For SFN, the precision and accuracy over the six lots of hCSF were 4.86% and 123% respectively at the low QC level, and 2.68% and 111% respectively at the high QC level (Table 12a), thus the acceptance criteria of ≤15% was not met for accuracy at one level only (see section 3, point 4).

For SFN-NAC, the precision and accuracy over the six lots of hCSF were 2.92% and 90% respectively at the low QC level, and 3.17% and 91% respectively at the high QC level (Table 12b), thus meeting the acceptance criteria of ≤15%.

For SFN-GSH, the precision and accuracy over the six lots of hCSF were 5.16% and 92% respectively at the low QC level, and 4.84% and 108% respectively at the high QC level (Table 12c), thus meeting the acceptance criteria of ≤15%.

Carryover Evaluation

Blank samples were analysed after each ULOQ standard (2000 ng/mL for SFN, SFN-NAC and SFN-GSH) to assess carryover. The response (peak area) for the blank samples must be ≤20% of the mean LLOQ response to be acceptable. No significant carryover was observed for SFN, SFN-NAC and SFN-GSH (Tables 13a to 13c).

In addition, the carryover response for the internal standard must be ≤5% of the mean internal standard response to be acceptable. No significant carryover was observed for the internal standard (Tables 14a to 14c).

Stability

Stability in matrix is proven (for all assessments, except solution stability) provided that at least 2/3 of the stability quality control samples at each level (as indicated in the stability sections below) are within ±15% of nominal concentrations and the mean concentrations do not deviate from the nominal concentrations by more than ±15% relative error.

Stability in analytical solutions is proven provided that the percent difference of the stored solution compared to the fresh solution is within ±10%.

Stability was determined for the following conditions:

2.1.1 Solution Stability (nominal 4ºC/-20ºC and Room Temperature)

2.1.1.1 Nominal 4ºC/-20ºC

Stock stability testing at a nominal 4ºC/-20ºC was performed as part of method validation study number 0014/001 (Ref. 3). The relevant results are included in Appendix 4.

2.1.2 Extract Stability

Extract Stability is determined over the anticipated time that an entire run may be stored prior to analysis. It is established by storing an entire run (at a minimum, blanks, calibration standards, and triplicate low, medium and high QC samples) for the desired time at the requisite temperature prior to injection. Extract stability is calculated from the completion of sample preparation until injection of the first QC sample or calibration standard.

This testing was not performed as part of this validation (see section 3, point 5).

2.1.3 Re-Injection Reproducibility / Autosampler Stability

Re-injection reproducibility / autosampler stability is determined over the anticipated time that a batch run may be stored prior to its re-injection. It is established by analysing a previously injected batch run (at a minimum, blanks, standards, and triplicate low, medium and high QC samples) for the desired time after storage at the requisite autosampler tray temperature. Re-injection reproducibility storage time is calculated from the original injection of the first QC sample or calibration standard until re-injection of the first QC sample or calibration standard.

The re-injected QC samples were compared to both the original calibration curve and to the re-injected calibration curve (Tables 15a to 15c). This is to confirm partial batch re-injection and full batch re-injection, respectively. Acceptance criteria is as per standard batch acceptance.

All acceptance criteria were met, therefore both partial batches and full batches of extracted samples of SFN, SFN-NAC and SFN-GSH can be re-injected, following storage on the autosampler of up to 24 hours.

2.1.4 Benchtop Stability

QC samples at three concentrations in both artificial and human cerebrospinal fluid were stored for 2.5 hours on wet ice prior to aliquoting and extracting in replicates of three.

- 15, 1600 and 8000 ng/mL for SFN and SFN-NAC
- 30, 1600 and 8000 ng/mL for SFN-GSH

The results indicate that SFN, SFN-NAC and SFN-GSH are stable in both artificial and human cerebrospinal fluid for at least 2.5 hours on wet ice (see section 3, point 6). The accuracy after 2.5

hours on wet ice in aCSF ranged from 92% to 103% for SFN, 95% to 103% for SFN-NAC and 96% to 109% for SFN-GSH (Tables 16a to 16c). The accuracy after 2.5 hours on wet ice in hCSF ranged from 112% to 116% for SFN, 84% to 100% for SFN-NAC and 84% to 94% for SFN-GSH (Tables 17a to 17c).

2.1.5 Freeze/Thaw Stability

QC samples at three concentrations in both artificial and human cerebrospinal fluid were prepared and subjected to two freeze (-80°C)/thaw cycles (see section 3, point 7). At the end of the second freeze/thaw cycle, the samples were aliquoted in triplicate, extracted, and analysed.

- 15, 1600 and 8000 ng/mL for SFN and SFN-NAC
- 30, 1600 and 8000 ng/mL for SFN-GSH

The results indicate that SFN, SFN-NAC and SFN-GSH are stable in both artificial and human cerebrospinal fluid for at least two freeze (-80°C)/thaw cycles prior to analysis (see section 3, point 8). The accuracy after two freeze (-80°C)/thaw cycles in aCSF ranged from 91% to 98% for SFN, 95% to 102% for SFN-NAC and 97% to 119% for SFN-GSH (Tables 18a to 18c). The accuracy after two freeze (-80°C)/thaw cycles in hCSF ranged from 108% to 109% for SFN, 85% to 96% for SFN-NAC and 94% to 118% for SFN-GSH (Tables 19a to 19c).

2.1.6 Interference Screens

SFN, SFN-NAC and SFN-GSH were analysed independently to monitor the contribution of each analyte on the other. This was performed at the Low QC and High QC level for each analyte.

Independent samples containing each separate analyte were prepared at the Low QC and High QC levels (without addition of internal standard) in artificial CSF and analysed in triplicate. These were analysed for the other analytes (i.e. samples spiked with SFN will be analysed for SFN-NAC and SFN-GSH, and so on).

The maximum amount of interference noted in any one sample was 12.69%, other than for SFN (Tables 20a to 20c). It was previously known that the pure standards of the metabolites contained SFN, therefore the standard interference screen acceptance criteria of <20% of the mean Low QC level did not apply to that analyte. All interference screen results were therefore acceptable.

2.1.7 Long Term Storage Stability

A separate study, to investigate frozen stability over three time points, has been agreed. This will therefore be covered in a separate report (study number 0014/004).

2.1.8 Retention Time

Absolute retention was monitored during each validation run by comparing the retention times of the analyte and internal standard between the beginning and end of each run. These varied by no more than 10% for any run.

2.1.9 Typical Chromatograms

Typical chromatograms of a blank sample, and at the lower and upper limits of quantification, are shown in Figures 4 to 7.

2.1.10 Analytical Notes

A summary of all runs performed in the validation of the bioanalytical method for SFN, SFN-NAC and SFN-GSH in CSF is provided below:

**Run No. (Date)**	**Description / Comments**	**Results**
Run 1<br>11 Apr 2016	• n/a	Not used as a result of updated method
Run 2<br>14 Apr 2016	• n/a	Not used as a result of updated method
Run 3<br>15 Apr 2016	• n/a	Not used as a result of updated method
Run 4<br>18 Apr 2016	• n/a	Not used as a result of updated method
Run 5<br>19 Apr 2016	• n/a	Not used as a result of updated method
Run 6<br>25 Apr 2016	• Precision & Accuracy Run 1<br>• Carryover	Pass
Run 7<br>26 Apr 2016	• Precision & Accuracy Run 2<br>• Carryover	Pass
Run 8<br>27 Apr 2016	• Precision & Accuracy Run 3<br>• Carryover<br>• Selectivity	Pass
Run 9<br>28 Apr 2016	• Dilution QCs	Pass
Run 10<br>29 Apr 2016	• Reinjection Reproducibility (24 hours)	Pass

Run 11<br>03 May 2016	• Precision & Accuracy at ULOQ<br>• aCSF + 0.1% Blood Testing<br>• hCSF Testing	Pass
Run 12<br>03 May 2016	• n/a	Fail – Additional on-system testing, insufficient stability
Run 13<br>04 May 2016	• Recovery<br>• Matrix Effects	Pass
Run 14<br>06 May 2016	• Interference Screens	Pass
Run 15<br>09 May 2016	• Bench-Top Stability	Pass
Run 16<br>10 May 2016	• Freeze-Thaw Cycling	Pass

3 DEVIATIONS

1) One intra-day result for SFN-GSH on one batch run at one concentration only gave a 116% accuracy, outside standard acceptance of 85-115%. This is considered to be acceptable.

2) It is expected that this method will only be used to analyse small numbers of clinical samples, which will be received and analysed on an ongoing basis. Therefore, evaluation of a large batch size was not considered to be required as part of this validation.

3) Post-spiked samples' precision values for SFN-NAC and SFN-GSH recovery testing were approximately 1-2% greater than standard acceptance criteria. This is not considered to be significant.

4) Human CSF accuracy results were outside standard acceptance criteria (123%), at the low QC level only, for SFN only. As the high QC level results for SFN were acceptable, and results in human CSF for the other two analytes were also acceptable, it is thought that this relates to a preparative error rather than a significant bioanalytical issue. This is therefore considered to be acceptable.

5) It is expected that all batch runs during sample analysis will be analysed immediately upon their preparation, therefore separate pre-analysis storage testing was not performed as part of this validation.

6) Bench-top stability testing was performed on wet ice over a period of 2.5 hours, rather than the 4 hours stated in the study plan. It will be ensured that this will be the maximum time for which samples will be stored on bench-top prior to extraction. For bench-top stability in hCSF, all three analytes had one accuracy result of ±16% at one concentration only, outside

standard acceptance of 85-115%. As all analytes' overall mean accuracy was within this range, this is considered to be acceptable.

7) Freeze-thaw cycling was performed over two, rather than three cycles. It will be ensured all clinical samples will undergo no more than two freeze-thaw cycles prior to analysis.

8) Freeze-thaw cycling results for SFN-GSH at the high QC level only were outside standard acceptance criteria for both aCSF and hCSF (119% and 118%). As this was seen in both matrices at one level only, and overall mean accuracy was within the standard acceptance range on both occasions, it is thought that this relates to a preparative error rather than a significant bioanalytical issue. This is therefore considered to be acceptable.

4 CONCLUSIONS

The LC-MS/MS analytical method for the determination of SFN, SFN-NAC and SFN-GSH concentrations in human CSF, using artificial CSF as a surrogate matrix, over ranges of 5 to 2000 ng/mL for SFN and SFN-NAC, and 10 to 2000 ng/mL for SFN-GSH, using a sample volume of 100 µL, has been demonstrated to be precise and accurate, and is suitable for the analysis of clinical study samples.

5 ARCHIVE PROCEDURE

All records of the study including the validation plan, raw data and approved final report are archived at Alderley Analytical, approved and documented according to Alderley Analytical SOP QA009 (Ref. 2). Records will be retained for a period of two years from report finalisation, after which time the Sponsor will be contacted to determine requirements for further storage, return or destruction of materials. No materials will be destroyed without written instruction from the Sponsor.

6 REFERENCES

1. Alderley Analytical SOP L001: Validation of Bioanalytical Methods.
2. Alderley Analytical SOP QA009: Archiving Procedures / Records Management.
3. Alderley Analytical Study No. 0014/001: The Validation of an Analytical Procedure for the Determination of Sulforaphane (SFN), Sulforaphane N-acetyl Cysteine (SFN-NAC) and Sulforaphane Glutathione (SFN-GSH) in Human K_2 EDTA Stabilised Plasma by LC-MS/MS.

7 TABLES

Table 1a Selectivity for SFN

Sample ID	SFN Peak Area	% Response compared to Mean
LLOQ QC1	18923	
LLOQ QC2	18990	
LLOQ QC3	21434	
LLOQ QC4	20038	
LLOQ QC5	19223	
LLOQ QC6	21869	
Mean	20080	
Matrix 1	0	0.00%
Matrix 2	0	0.00%
Matrix 3	2012	10.02%
Matrix 4	2585	12.87%
Matrix 5	0	0.00%
Matrix 6	2180	10.86%

Table 1b Selectivity for SFN Internal Standard

Sample ID	SFN IS Peak Area	% Response compared to IS Mean
LLOQ QC1	522167	
LLOQ QC2	548787	
LLOQ QC3	549905	
LLOQ QC4	559015	
LLOQ QC5	534934	
LLOQ QC6	585325	
Mean	550022	
Matrix 1	0	0.00%
Matrix 2	0	0.00%
Matrix 3	0	0.00%
Matrix 4	0	0.00%
Matrix 5	0	0.00%
Matrix 6	0	0.00%

Table 1c Selectivity for SFN-NAC

Sample ID	SFN-NAC Peak Area	% Response compared to Mean
LLOQ QC1	33717	
LLOQ QC2	39590	
LLOQ QC3	39895	
LLOQ QC4	48812	
LLOQ QC5	42928	
LLOQ QC6	39196	
Mean	40690	
Matrix 1	0	0.00%
Matrix 2	0	0.00%
Matrix 3	0	0.00%
Matrix 4	0	0.00%
Matrix 5	0	0.00%
Matrix 6	0	0.00%

Table 1d Selectivity for SFN-NAC Internal Standard

Sample ID	SFN-NAC IS Peak Area	% Response compared to IS Mean
LLOQ QC1	297131	
LLOQ QC2	299396	
LLOQ QC3	317563	
LLOQ QC4	306223	
LLOQ QC5	278658	
LLOQ QC6	280457	
Mean	296571	
Matrix 1	10629	3.58%
Matrix 2	0	0.00%
Matrix 3	0	0.00%
Matrix 4	0	0.00%
Matrix 5	0	0.00%
Matrix 6	0	0.00%

Table 1e Selectivity for SFN-GSH

Sample ID	SFN-GSH Peak Area	% Response compared to Mean
LLOQ QC1	251939	
LLOQ QC2	257623	
LLOQ QC3	251691	
LLOQ QC4	268555	
LLOQ QC5	246069	
LLOQ QC6	228207	
Mean	250681	
Matrix 1	0	0.00%
Matrix 2	0	0.00%
Matrix 3	3820	1.52%
Matrix 4	0	0.00%
Matrix 5	8681	3.46%
Matrix 6	0	0.00%

Table 1f Selectivity for SFN-GSH Internal Standard

Sample ID	SFN-GSH IS Peak Area	% Response compared to IS Mean
LLOQ QC1	309300	
LLOQ QC2	303793	
LLOQ QC3	316975	
LLOQ QC4	293182	
LLOQ QC5	303600	
LLOQ QC6	302447	
Mean	304883	
Matrix 1	10629	3.49%
Matrix 2	0	0.00%
Matrix 3	0	0.00%
Matrix 4	0	0.00%
Matrix 5	0	0.00%
Matrix 6	0	0.00%

Table 2a Standard Curve Parameters for SFN

Run Number	Gradient	Intercept	Correlation (R^2)
Run 6	5.37×10^{-3}	6.86×10^{-3}	0.995270
Run 7	5.86×10^{-3}	5.65×10^{-3}	0.992853
Run 8	6.13×10^{-3}	6.10×10^{-3}	0.989477
Run 9	8.20×10^{-3}	3.29×10^{-3}	0.998728
Run 10	7.91×10^{-3}	7.54×10^{-3}	0.990996
Run 11	5.46×10^{-3}	7.33×10^{-3}	0.996198
Run 13	N/A	N/A	N/A
Run 14	N/A	N/A	N/A
Run 15	5.51×10^{-3}	5.90×10^{-3}	0.994734
Run 16	5.81×10^{-3}	5.76×10^{-3}	0.994188

Runs 1-5 and 12 standard curve summary data not tabulated as the runs were not used as part of the validation, as described in Section 2.1.10.

No calibration curve was run with Runs 13 and 14 as results used peak areas only.

Table 2b Standard Curve Parameters for SFN-NAC

Run Number	Gradient	Intercept	Correlation (R^2)
Run 6	2.42×10^{-2}	-1.03×10^{-2}	0.993750
Run 7	2.64×10^{-2}	-9.52×10^{-3}	0.990379
Run 8	2.38×10^{-2}	9.04×10^{-3}	0.993938
Run 9	2.57×10^{-2}	1.18×10^{-2}	0.992599
Run 10	2.47×10^{-2}	2.79×10^{-3}	0.989107
Run 11	2.62×10^{-2}	1.68×10^{-2}	0.997285
Run 13	N/A	N/A	N/A
Run 14	N/A	N/A	N/A
Run 15	2.60×10^{-2}	3.11×10^{-3}	0.994905
Run 16	2.68×10^{-2}	9.06×10^{-3}	0.996980

Runs 1-5 and 12 standard curve summary data not tabulated as the runs were not used as part of the validation, as described in Section 2.1.10.

No calibration curve was run with Runs 13 and 14 as results used peak areas only.

Table 2c Standard Curve Parameters for SFN-GSH

Run Number	Gradient	Intercept	Correlation (R^2)
Run 6	5.97×10^{-2}	7.03×10^{-2}	0.988169
Run 7	8.53×10^{-2}	-2.77×10^{-2}	0.989047
Run 8	8.98×10^{-2}	-5.32×10^{-2}	0.996910
Run 9	9.97×10^{-2}	2.22×10^{-1}	0.988412
Run 10	9.65×10^{-2}	2.32×10^{-1}	0.982919
Run 11	6.40×10^{-2}	5.07×10^{-3}	0.993318
Run 13	N/A	N/A	N/A
Run 14	N/A	N/A	N/A
Run 15	6.05×10^{-2}	-9.34×10^{-2}	0.988898
Run 16	5.86×10^{-2}	-6.77×10^{-2}	0.993799

Runs 1-5 and 12 standard curve summary data not tabulated as the runs were not used as part of the validation, as described in Section 2.1.10.

No calibration curve was run with Runs 13 and 14 as results used peak areas only.

Table 3a Standard Curve Back Calculated Concentrations of SFN

Run Number#	Nominal Concentration (ng/mL)								
	5.00	10.00	25.00	50.00	100.00	250.00	500.00	1000.00	2000.00
Run 6	4.68	10.14	26.89	49.63	106.60	256.46	517.42	970.99	1906.04
	5.38	9.26							1858.39
Run 7	5.81	9.76	25.37	49.05	103.49	251.46	509.76	1026.72	1951.15
	4.26	9.88							1925.08
Run 8	5.79	10.62	26.39	52.92	103.95	250.49	509.24	962.52	1921.49
	4.14	9.29							1852.85
Run 9	4.97	9.38	25.82	50.85	103.04	251.97	499.97	981.90	2031.91
	5.14	9.97							1912.45
Run 10	5.58	10.67	26.81	53.82	104.25	257.41	474.03	926.03	1996.14
	4.40	8.93							1901.61
Run 11	5.34	9.69	27.49	50.41	103.96	254.57	495.27	991.89	1928.73
	4.84	9.13							1938.85
Run 15	4.78	9.54	27.83	49.60	103.66	256.26	493.60	998.01	1891.19
	5.46	9.03							1995.97
Run 16	5.14	11.31	25.57	54.94	95.46	248.91	490.91	955.47	1995.98
	4.68	9.18							1959.64
Number	16	16	8	8	8	8	8	8	16
Mean	5.02	9.74	26.52	51.40	103.05	253.44	498.78	976.69	1935.47
SD	0.52	0.67	0.90	2.20	3.25	3.15	13.59	30.39	50.84
CV	10.34%	6.91%	3.38%	4.28%	3.15%	1.24%	2.72%	3.11%	2.63%
Accuracy	100.49%	97.36%	106.09%	102.81%	103.05%	101.38%	99.76%	97.67%	96.77%

Key

Runs 1-5 and 12 standard curve concentration data not tabulated as the runs were not used as part of the validation, as described in Section 2.1.10.

Table 3b **Standard Curve Back Calculated Concentrations of SFN-NAC**

Run Number[#]	Nominal Concentration (ng/mL)								
	5.00	10.00	25.00	50.00	100.00	250.00	500.00	1000.00	2000.00
Run 6	4.83	9.67	27.31	51.21	101.06	252.13	509.63	947.76	1762.95
	4.82	11.31							1976.71
Run 7	5.61	8.55	26.93	54.44	98.48	248.81	500.92	994.26	1939.80
	4.44	10.78							1889.82
Run 8	5.13	10.54	24.73	50.80	102.01	255.12	507.11	996.87	1819.23
	4.42	11.26							1888.06
Run 9	4.34	9.84	25.01	54.34	104.21	251.57	491.29	1005.61	1862.17
	5.74	9.63							1964.07
Run 10	4.08	9.52	24.81	47.77	96.66	257.61	516.55	997.67	1980.43
	5.98	10.38							2064.84
Run 11	4.83	9.66	24.44	49.45	100.91	261.64	517.16	1004.91	1898.72
	5.42	9.41							2063.75
Run 15	4.53	10.10	27.92	51.09	98.54	257.59	497.97	988.79	1900.65
	5.43	9.55							1905.57
Run 16	4.85	9.91	25.37	54.71	98.58	259.46	500.17	991.74	1880.62
	4.99	10.47							1855.84
Number	16	16	8	8	8	8	8	8	16
Mean	4.97	10.04	25.82	51.73	100.06	255.49	505.10	990.95	1915.83
SD	0.55	0.72	1.35	2.55	2.43	4.37	9.14	18.41	80.57
CV	11.04%	7.20%	5.25%	4.93%	2.43%	1.71%	1.81%	1.86%	4.21%
Accuracy	99.30%	100.36%	103.26%	103.45%	100.06%	102.20%	101.02%	99.10%	95.79%

Key

Runs 1-5 and 12 standard curve concentration data not tabulated as the runs were not used as part of the validation, as described in Section 2.1.10.

Table 3c Standard Curve Back Calculated Concentrations of SFN-GSH

Run Number#	Nominal Concentration (ng/mL)							
	10.00	25.00	50.00	100.00	250.00	500.00	1000.00	2000.00
Run 6	8.35	28.20	49.97	99.33	233.22	519.43	948.80	2013.79
	11.17							*
Run 7	10.99	26.05	54.80	95.86	220.75	491.11	979.13	2262.41
	8.74							1909.79
Run 8	10.58	23.61	52.38	92.60	257.66	499.87	1050.04	2022.55
	9.60							1943.74
Run 9	8.24	*	55.75	98.65	248.98	473.62	960.88	2036.15
	11.56							1993.10
Run 10	8.18	28.75	46.10	94.89	243.74	458.12	956.10	2082.37
	11.45							2255.44
Run 11	8.89	23.98	51.05	100.62	239.46	492.37	986.23	2143.25
	11.24							*
Run 15	10.64	25.77	43.55	89.99	233.20	474.93	1044.44	2282.34
	9.62							2209.17
Run 16	9.89	25.15	48.39	90.42	233.40	498.09	1048.23	2257.69
	10.27							*
Number	16	7	8	8	8	8	8	13
Mean	9.96	25.93	50.25	95.30	238.80	488.44	996.73	2108.60
SD	1.20	1.96	4.17	4.06	11.35	19.00	43.79	132.70
CV	12.01%	7.54%	8.30%	4.26%	4.75%	3.89%	4.39%	6.29%
Accuracy	99.63%	103.72%	100.50%	95.30%	95.52%	97.69%	99.67%	105.43%

Key

# Runs 1-5 and 12 standard curve concentration data not tabulated as the runs were not used as part of the validation, as described in Section 2.1.10.

* Calibration standard removed from the regression equation as the % deviation exceeded the acceptance criteria of ±15% (±20% at LLOQ).

Table 4a Intra-Run Quality Control Sample Concentrations of SFN

Nominal Conc.	LLOQ QC1 5.00 ng/mL	Low QC2 15.00 ng/mL	Mid QC3 800.00 ng/mL	High QC4 1600.00 ng/mL
	Calc. Conc.	Calc. Conc.	Calc. Conc.	Calc. Conc.
Run 6 (P&A Run 1)	4.20	14.56	706.52	1485.87
	4.36	13.91	714.04	1364.63
	3.71	12.88	741.39	1357.30
	4.47	11.79	732.46	1372.96
	4.49	14.85	722.70	1352.21
	4.85	12.67	713.23	1391.77
Mean	4.34	13.44	721.72	1387.46
SD	0.38	1.19	13.15	50.17
CV	8.72%	8.87%	1.82%	3.62%
Accuracy	87%	90%	90%	87%
Run 7 (P&A Run 2)	4.22	14.22	751.37	1539.59
	4.53	14.68	773.27	1545.49
	4.54	12.42	779.14	1553.97
	5.89	13.54	791.47	1550.06
	4.93	14.68	755.68	1550.57
	4.65	13.52	764.91	1559.21
Mean	4.79	13.84	769.30	1549.82
SD	0.58	0.87	15.03	6.77
CV	12.20%	6.26%	1.95%	0.44%
Accuracy	96%	92%	96%	97%
Run 8 (P&A Run 3)	4.92	13.86	741.96	1524.49
	4.65	12.80	764.00	1477.80
	5.36	13.83	730.54	1595.40
	4.85	13.21	731.47	1487.62
	4.87	14.38	759.16	1543.04
	5.10	14.09	756.88	1506.93
Mean	4.96	13.70	747.34	1522.55
SD	0.25	0.58	14.64	42.89
CV	4.94%	4.26%	1.96%	2.82%
Accuracy	99%	91%	93%	95%

Table 4b Intra-Run Quality Control Sample Concentrations of SFN-NAC

Nominal Conc.	LLOQ QC1 5.00 ng/mL	Low QC2 15.00 ng/mL	Mid QC3 800.00 ng/mL	High QC4 1600.00 ng/mL
	Calc. Conc.	Calc. Conc.	Calc. Conc.	Calc. Conc.
Run 6 (P&A Run 1)	3.96	15.23	698.44	1507.93
	4.54	16.18	757.54	1370.30
	5.88	14.46	761.67	1309.78
	5.49	15.49	785.69	1405.26
	3.87	12.30	758.32	1461.84
	5.28	15.93	770.24	1435.02
Mean	4.84	14.93	755.32	1415.02
SD	0.84	1.42	29.78	69.85
CV	17.31%	9.51%	3.94%	4.94%
Accuracy	97%	100%	94%	88%
Run 7 (P&A Run 2)	5.87	14.29	784.04	1558.09
	5.68	15.72	782.31	1552.10
	5.18	15.58	785.94	1592.57
	5.10	14.29	763.68	1525.98
	4.52	14.03	793.31	1619.60
	5.29	14.95	803.39	1670.94
Mean	5.27	14.81	785.45	1586.55
SD	0.47	0.72	13.19	52.76
CV	9.00%	4.87%	1.68%	3.33%
Accuracy	105%	99%	98%	99%
Run 8 (P&A Run 3)	4.39	14.21	808.88	1673.45
	5.17	14.13	827.50	1535.07
	4.90	14.52	835.60	1637.67
	6.31	13.85	830.34	1716.63
	6.09	14.53	810.52	1692.19
	5.49	16.40	837.26	1605.95
Mean	5.39	14.61	825.02	1643.49
SD	0.73	0.91	12.38	66.06
CV	13.51%	6.26%	1.50%	4.02%
Accuracy	108%	97%	103%	103%

Table 4c Intra-Run Quality Control Sample Concentrations of SFN-GSH

Nominal Conc.	LLOQ QC1 10.00 ng/mL	Low QC2 30.00 ng/mL	Mid QC3 800.00 ng/mL	High QC4 1600.00 ng/mL
	Calc. Conc.	Calc. Conc.	Calc. Conc.	Calc. Conc.
Run 6 (P&A Run 1)	11.63	36.09	794.65	1679.98
	11.36	30.89	840.21	1478.83
	10.82	35.81	908.69	1462.41
	14.62	33.77	956.66	1707.15
	11.98	37.98	909.02	1744.08
	12.32	44.36	860.06	1685.40
Mean	12.12	36.48	878.21	1626.31
SD	1.33	4.55	57.95	122.79
CV	10.95%	12.48%	6.60%	7.55%
Accuracy	121%	122%	110%	102%
Run 7 (P&A Run 2)	9.79	30.91	761.02	1614.15
	10.62	29.90	772.40	1638.27
	10.10	32.80	695.37	1678.89
	11.29	34.64	773.24	1675.70
	12.59	30.25	719.57	1774.82
	10.11	28.16	707.38	1602.97
Mean	10.75	31.11	738.16	1664.13
SD	1.04	2.29	34.78	62.47
CV	9.70%	7.37%	4.71%	3.75%
Accuracy	107%	104%	92%	104%
Run 8 (P&A Run 3)	9.66	26.22	803.06	1709.66
	10.03	27.55	763.92	1588.78
	9.43	25.54	720.25	1652.02
	10.79	25.33	714.68	1695.98
	9.61	28.12	760.16	1590.27
	8.99	25.97	754.88	1499.51
Mean	9.75	26.46	752.82	1622.71
SD	0.61	1.13	32.31	78.96
CV	6.26%	4.26%	4.29%	4.87%
Accuracy	98%	88%	94%	101%

Table 4d **Intra-Run Quality Control Sample Concentrations of SFN-GSH (outliers removed)**

Nominal Conc.	LLOQ QC1 10.00 ng/mL	Low QC2 30.00 ng/mL	Mid QC3 800.00 ng/mL	High QC4 1600.00 ng/mL
	Calc. Conc.	Calc. Conc.	Calc. Conc.	Calc. Conc.
Run 6 (P&A Run 1)	11.63	36.09	794.65	1679.98
	11.36	30.89	840.21	1478.83
	10.82	35.81	908.69	1462.41
	14.62*	33.77	956.66	1707.15
	11.98	37.98	909.02	1744.08
	12.32	44.36*	860.06	1685.40
Mean	11.62	34.91	878.21	1626.31
SD	0.58	2.70	57.95	122.79
CV	4.96%	7.73%	6.60%	7.55%
Accuracy	116%	116%	110%	102%
Run 7 (P&A Run 2)	9.79	30.91	761.02	1614.15
	10.62	29.90	772.40	1638.27
	10.10	32.80	695.37	1678.89
	11.29	34.64	773.24	1675.70
	12.59	30.25	719.57	1774.82
	10.11	28.16	707.38	1602.97
Mean	10.75	31.11	738.16	1664.13
SD	1.04	2.29	34.78	62.47
CV	9.70%	7.37%	4.71%	3.75%
Accuracy	107%	104%	92%	104%
Run 8 (P&A Run 3)	9.66	26.22	803.06	1709.66
	10.03	27.55	763.92	1588.78
	9.43	25.54	720.25	1652.02
	10.79	25.33	714.68	1695.98
	9.61	28.12	760.16	1590.27
	8.99	25.97	754.88	1499.51
Mean	9.75	26.46	752.82	1622.71
SD	0.61	1.13	32.31	78.96
CV	6.26%	4.26%	4.29%	4.87%
Accuracy	98%	88%	94%	101%

* QC sample result removed from calculations due to being a statistical outlier

Table 4e **Inter-Run Quality Control Sample Concentrations of SFN**

Nominal Conc. (ng/mL)	Number	Mean	SD	CV	Accuracy
5.00	18	4.70	0.48	10.24%	94%
15.00	18	13.66	0.88	6.42%	91%
800.00	18	746.12	24.10	3.23%	93%
1600.00	18	1486.61	81.43	5.48%	93%

Table 4f **Inter-Run Quality Control Sample Concentrations of SFN-NAC**

Nominal Conc. (ng/mL)	Number	Mean	SD	CV	Accuracy
5.00	18	5.17	0.70	13.53%	103%
15.00	18	14.78	1.01	6.80%	99%
800.00	18	788.59	34.92	4.43%	99%
1600.00	18	1548.35	116.28	7.51%	97%

Table 4g **Inter-Run Quality Control Sample Concentrations of SFN-GSH**

Nominal Conc. (ng/mL)	Number	Mean	SD	CV	Accuracy
10.00	18	10.87	1.40	12.84%	109%
30.00	18	31.35	5.08	16.20%	104%
800.00	18	789.73	76.37	9.67%	99%
1600.00	18	1637.72	88.25	5.39%	102%

Table 4h **Inter-Run Quality Control Sample Concentrations of SFN-GSH (outliers removed)**

Nominal Conc. (ng/mL)	Number	Mean	SD	CV	Accuracy
10.00	17	10.65	1.07	10.03%	107%
30.00	17	30.58	4.02	13.16%	102%
800.00	18	789.73	76.37	9.67%	99%
1600.00	18	1637.72	88.25	5.39%	102%

Table 5a Upper Limit of Quantitation for SFN

Nominal Conc.	ULOQ QC5<br>2000.00 ng/mL
	Calc. Conc.
Run 11	2052.06<br>1992.45<br>2002.80<br>1969.68<br>1985.77<br>1953.84
Mean<br>SD<br>CV<br>Accuracy	1992.77<br>33.80<br>1.70%<br>100%

Table 5b Upper Limit of Quantitation for SFN-NAC

Nominal Conc.	ULOQ QC5<br>2000.00 ng/mL
	Calc. Conc.
Run 11	1962.62<br>2016.43<br>2002.83<br>2017.88<br>1969.84<br>1997.51
Mean<br>SD<br>CV<br>Accuracy	1994.52<br>23.37<br>1.17%<br>100%

Table 5c Upper Limit of Quantitation for SFN-GSH

Nominal Conc.	ULOQ QC5 2000.00 ng/mL
	Calc. Conc.
Run 11	2336.84
	2392.26
	2340.16
	2230.05
	2219.89
	2226.69
Mean	2290.98
SD	74.40
CV	3.25%
Accuracy	115%

Table 6a Dilution Integrity (10-fold) for SFN

Nominal Conc.	Dil QC 8000.00 ng/mL
	Calc. Conc.
Run 9	7182.31
	7965.28
	7734.52
	7414.46
	7499.47
	7871.16
Mean	7611.20
SD	297.76
CV	3.91%
Accuracy	95%

Table 6b Dilution Integrity (10-fold) for SFN-NAC

Nominal Conc.	Dil QC 8000.00 ng/mL
	Calc. Conc.
Run 9	7161.85
	7975.31
	7166.51
	7714.79
	7244.04
	7553.99
Mean	7469.42
SD	334.79
CV	4.48%
Accuracy	93%

Table 6c Dilution Integrity (10-fold) for SFN-GSH

Nominal Conc.	Dil QC 8000.00 ng/mL
	Calc. Conc.
Run 9	7103.00
	7928.71
	6931.98
	7495.51
	7251.80
	6930.76
Mean	7273.62
SD	385.29
CV	5.30%
Accuracy	91%

Table 7a Recovery (Extraction Efficiency) for SFN

Nominal Conc.	Low QC2<br>15.00 ng/mL		Mid QC3<br>800.00 ng/mL		High QC4<br>1600.00 ng/mL	
	Peak Areas		Peak Areas		Peak Areas	
	Post Spiked	Extracted	Post Spiked	Extracted	Post Spiked	Extracted
Run 13	38072	38087	1897239	1955262	3929017	3770366
	40842	41599	1968212	1921232	3542619	3542645
	39783	41219	2086821	2001292	3820315	3423307
Mean	39565	40302	1984090	1959262	3763984	3578773
SD	1398	1928	95783	40179	199263	176328
CV	3.53%	4.78%	4.83%	2.05%	5.29%	4.93%
Recovery		102%		99%		95%

Table 7b Recovery (Extraction Efficiency) for SFN-NAC

Nominal Conc.	Low QC2<br>15.00 ng/mL		Mid QC3<br>800.00 ng/mL		High QC4<br>1600.00 ng/mL	
	Peak Areas		Peak Areas		Peak Areas	
	Post Spiked	Extracted	Post Spiked	Extracted	Post Spiked	Extracted
Run 13	232363	128457	10414756	7992349	18687094	14071366
	249479	139641	10836101	8007427	17335861	13871977
	246413	142198	11096040	8466046	18055172	13633432
Mean	242752	136765	10782299	8155274	18026043	13858925
SD	9127	7308	343814	269242	676087	219259
CV	3.76%	5.34%	3.19%	3.30%	3.75%	1.58%
Recovery		56%		76%		77%

Table 7c Recovery (Extraction Efficiency) for SFN-GSH

Nominal Conc.	Low QC2<br>30.00 ng/mL		Mid QC3<br>800.00 ng/mL		High QC4<br>1600.00 ng/mL	
	Peak Areas		Peak Areas		Peak Areas	
	Post Spiked	Extracted	Post Spiked	Extracted	Post Spiked	Extracted
Run 13	253555	78336	46644050	18818982	88897351	36991263
	263955	65052	49634606	18313830	83152238	35649496
	248022	66511	50722141	19172679	88811907	33233128
Mean	255177	69966	49000266	18768497	86953832	35291296
SD	8090	7285	2111752	431644	3292554	1904501
CV	3.17%	10.41%	4.31%	2.30%	3.79%	5.40%
Recovery		27%		38%		41%

Table 8a Recovery (Extraction Efficiency) for SFN Internal Standard

	IS In-Sample Conc. 50 ng/mL	
	IS Peak Areas	
	Post Spiked	Extracted
Low QC2	477902<br>477665<br>462726	429370<br>428232<br>419113
Mid QC3	444837<br>410632<br>439987	396500<br>403286<br>444194
High QC4	460106<br>434556<br>442236	404009<br>382383<br>392964
Mean<br>SD<br>CV<br>Recovery	450072<br>21747<br>4.83%	411117<br>20202<br>4.91%<br>91%

Table 8b Recovery (Extraction Efficiency) for SFN-NAC Internal Standard

	IS In-Sample Conc. 50 ng/mL	
	IS Peak Areas	
	Post Spiked	Extracted
Low QC2	702845<br>704348<br>740837	379509<br>380996<br>396357
Mid QC3	559700<br>572847<br>588205	427709<br>404418<br>424312
High QC4	540620<br>493878<br>489794	365126<br>373470<br>366381
Mean<br>SD<br>CV<br>Recovery	599230<br>94027<br>15.69%	390920<br>23667<br>6.05%<br>65%

Table 8c **Recovery (Extraction Efficiency) for SFN-GSH Internal Standard**

	IS In-Sample Conc. 50 ng/mL	
	IS Peak Areas	
	Post Spiked	Extracted
Low QC2	760774<br>736142<br>708964	376019<br>391571<br>399040
Mid QC3	559700<br>572847<br>588205	427709<br>404418<br>424312
High QC4	540620<br>493878<br>489794	365126<br>373470<br>366381
Mean<br>SD<br>CV<br>Recovery	605658<br>103321<br>17.06%	392005<br>23718<br>6.05%<br>65%

Table 9a **Matrix Effects at 15 ng/mL of SFN (Low QC)**

	Peak Area						
	Solution	Matrix 1	Matrix 2	Matrix 3	Matrix 4	Matrix 5	Matrix 6
Low QC1	65214	21820	49369	54349	38970	39444	47034
Low QC2	48462	22591	33588	33796	35333	32533	40210
Low QC3	43507	22118	24459	33787	30102	28618	37611
Mean	52394	22176	35805	40644	34802	33532	41619
CV	21.71%	1.75%	35.20%	29.20%	12.81%	16.35%	11.69%
Matrix Factor		0.4233	0.6834	0.7757	0.6642	0.6400	0.7943
Overall Precision							20.04%

Table 9b **Matrix Effects at 1600 ng/mL of SFN (High QC)**

	Peak Area						
	Solution	Matrix 1	Matrix 2	Matrix 3	Matrix 4	Matrix 5	Matrix 6
High QC1	3808418	1927255	3084863	2723257	2473716	2517807	2820952
High QC2	3187527	2163583	2674439	2565547	2193984	2539397	2964613
High QC3	3243873	2182702	2575724	2649405	2386150	2653433	2785350
Mean	3413273	2091180	2778342	2646070	2351284	2570212	2856972
CV	10.06%	6.80%	9.72%	2.98%	6.09%	2.84%	3.32%
Matrix Factor		0.6127	0.8140	0.7752	0.6889	0.7530	0.8370
Overall Precision							11.18%

Table 9c **Matrix Effects at 15 ng/mL of SFN-NAC (Low QC)**

	Peak Area						
	Solution	Matrix 1	Matrix 2	Matrix 3	Matrix 4	Matrix 5	Matrix 6
Low QC1	134504	157314	158668	185823	154239	175804	183973
Low QC2	171849	170037	151022	183962	189421	160487	207195
Low QC3	153579	151146	125404	196368	190652	167664	195626
Mean	153311	159499	145032	188718	178104	167985	195598
CV	12.18%	6.04%	12.01%	3.55%	11.61%	4.56%	5.94%
Matrix Factor		1.0404	0.9460	1.2309	1.1617	1.0957	1.2758
Overall Precision							10.91%

Table 9d **Matrix Effects at 1600 ng/mL of SFN-NAC (High QC)**

	Peak Area						
	Solution	Matrix 1	Matrix 2	Matrix 3	Matrix 4	Matrix 5	Matrix 6
High QC1	13904961	12758528	12801548	13094616	12853907	12627203	13369210
High QC2	14260472	14304996	12078609	12999648	12230080	13428834	14569333
High QC3	14323102	15510058	12499325	14739536	14263841	14849424	13966086
Mean	14162845	14191194	12459827	13611267	13115943	13635154	13968210
CV	1.59%	9.72%	2.91%	7.19%	7.94%	8.25%	4.30%
Matrix Factor		1.0020	0.8798	0.9611	0.9261	0.9627	0.9863
Overall Precision							4.64%

Table 9e **Matrix Effects at 30 ng/mL of SFN-GSH (Low QC)**

	Peak Area						
	Solution	Matrix 1	Matrix 2	Matrix 3	Matrix 4	Matrix 5	Matrix 6
Low QC1	108066	178253	179025	160314	161815	178361	214757
Low QC2	112859	204779	161857	157498	189814	181544	196168
Low QC3	116554	212665	200371	182731	189298	196481	237723
Mean	112493	198566	180418	166848	180309	185462	216216
CV	3.78%	9.08%	10.69%	8.29%	8.88%	5.22%	9.63%
Matrix Factor		1.7651	1.6038	1.4832	1.6028	1.6487	1.9220
Overall Precision							9.15%

Table 9f **Matrix Effects at 1600 ng/mL of SFN-GSH (High QC)**

	Peak Area						
	Solution	Matrix 1	Matrix 2	Matrix 3	Matrix 4	Matrix 5	Matrix 6
High QC1	53267140	70252499	74513374	70005281	68081585	67823241	67061570
High QC2	53842979	79814648	71719863	68877233	63614190	72021762	70887144
High QC3	54510516	78244062	67784233	70038635	71402704	76365019	64906176
Mean	53873545	76103736	71339157	69640383	67699493	72070007	67618296
CV	1.16%	6.74%	4.74%	0.95%	5.77%	5.93%	4.48%
Matrix Factor		1.4126	1.3242	1.2927	1.2566	1.3378	1.2551
Overall Precision							4.52%

Table 10a Normalised Matrix Effects at 15 ng/mL of SFN (Low QC)

	Peak Area Ratio						
	Solution	Matrix 1	Matrix 2	Matrix 3	Matrix 4	Matrix 5	Matrix 6
Low QC1	0.118	0.105	0.115	0.105	0.108	0.099	0.100
Low QC2	0.095	0.105	0.119	0.097	0.105	0.106	0.090
Low QC3	0.101	0.118	0.098	0.096	0.105	0.102	0.097
Mean	0.104	0.109	0.110	0.100	0.106	0.102	0.096
CV	11.39%	6.64%	10.28%	4.86%	1.77%	3.46%	5.36%
Normalised Matrix Factor		1.0451	1.0574	0.9544	1.0128	0.9765	0.9160
Overall Precision							5.50%

Table 10b Normalised Matrix Effects at 1600 ng/mL of SFN (High QC)

	Peak Area Ratio						
	Solution	Matrix 1	Matrix 2	Matrix 3	Matrix 4	Matrix 5	Matrix 6
High QC1	9.424	9.315	10.675	11.147	9.640	10.084	8.964
High QC2	9.182	10.248	11.254	10.085	9.054	10.694	9.148
High QC3	9.270	9.652	9.926	10.094	11.127	10.092	8.232
Mean	9.292	9.738	10.618	10.442	9.940	10.290	8.782
CV	1.32%	4.86%	6.27%	5.85%	10.75%	3.40%	5.52%
Normalised Matrix Factor		1.0480	1.1427	1.1237	1.0698	1.1074	0.9451
Overall Precision							6.67%

Table 10c Normalised Matrix Effects at 15 ng/mL of SFN-NAC (Low QC)

	Peak Area Ratio						
	Solution	Matrix 1	Matrix 2	Matrix 3	Matrix 4	Matrix 5	Matrix 6
Low QC1	0.325	0.288	0.262	0.267	0.266	0.286	0.342
Low QC2	0.355	0.290	0.347	0.325	0.318	0.276	0.360
Low QC3	0.365	0.297	0.305	0.329	0.336	0.313	0.362
Mean	0.349	0.292	0.304	0.307	0.307	0.292	0.355
CV	6.03%	1.46%	14.02%	11.34%	11.86%	6.41%	3.18%
Normalised Matrix Factor		0.8371	0.8735	0.8813	0.8794	0.8370	1.0180
Overall Precision							7.54%

Table 10d Normalised Matrix Effects at 1600 ng/mL of SFN-NAC (High QC)

	Peak Area Ratio						
	Solution	Matrix 1	Matrix 2	Matrix 3	Matrix 4	Matrix 5	Matrix 6
High QC1	38.257	30.849	30.509	33.624	31.742	29.026	34.320
High QC2	35.780	31.295	30.692	30.504	29.086	32.344	37.200
High QC3	36.208	30.501	31.371	33.515	34.093	34.469	32.002
Mean	36.748	30.882	30.857	32.548	31.640	31.946	34.508
CV	3.60%	1.29%	1.47%	5.44%	7.92%	8.59%	7.55%
Normalised Matrix Factor		0.8404	0.8397	0.8857	0.8610	0.8693	0.9390
Overall Precision							4.24%

Table 10e Normalised Matrix Effects at 30 ng/mL of SFN-GSH (Low QC)

	Peak Area Ratio						
	Solution	Matrix 1	Matrix 2	Matrix 3	Matrix 4	Matrix 5	Matrix 6
Low QC1	0.246	0.352	0.425	0.328	0.319	0.344	0.379
Low QC2	0.250	0.306	0.314	0.343	0.332	0.307	0.336
Low QC3	0.286	0.338	0.388	0.384	0.329	0.332	0.358
Mean	0.261	0.332	0.376	0.352	0.327	0.328	0.358
CV	8.46%	7.12%	14.98%	8.25%	2.07%	5.67%	5.90%
Normalised Matrix Factor		1.2725	1.4410	1.3501	1.2523	1.2564	1.3712
Overall Precision							5.75%

Table 10f Normalised Matrix Effects at 1600 ng/mL of SFN-GSH (High QC)

	Peak Area Ratio						
	Solution	Matrix 1	Matrix 2	Matrix 3	Matrix 4	Matrix 5	Matrix 6
High QC1	146.557	169.864	177.582	179.756	168.125	155.906	172.156
High QC2	135.093	174.612	182.241	161.622	151.289	173.466	180.999
High QC3	137.798	153.868	170.124	159.255	170.663	177.263	148.725
Mean	139.816	166.115	176.649	166.878	163.359	168.878	167.293
CV	4.29%	6.54%	3.46%	6.72%	6.45%	6.75%	9.97%
Normalised Matrix Factor		1.1881	1.2634	1.1936	1.1684	1.2079	1.1965
Overall Precision							2.69%

Table 11a SFN in aCSF + 0.1% Whole Blood

Nominal Conc.	Low QC2 15.00 ng/mL	High QC4 1600.00 ng/mL
	Calc. Conc.	Calc. Conc.
	16.64	1667.39
	15.07	1647.91
	17.62	1582.12
	13.86	1632.68
	16.39	1661.39
	15.61	1566.82
Mean	15.87	1626.39
SD	1.32	42.23
CV	8.32%	2.60%
Accuracy	106%	102%

Table 11b SFN-NAC in aCSF + 0.1% Whole Blood

Nominal Conc.	Low QC2 15.00 ng/mL	High QC4 1600.00 ng/mL
	Calc. Conc.	Calc. Conc.
	16.32	1568.93
	15.45	1557.32
	14.99	1588.61
	14.92	1570.45
	15.30	1542.95
	16.73	1517.06
Mean	15.62	1557.55
SD	0.74	24.95
CV	4.73%	1.60%
Accuracy	104%	97%

Table 11c SFN-GSH in aCSF + 0.1% Whole Blood

Nominal Conc.	Low QC2 30.00 ng/mL	High QC4 1600.00 ng/mL
	Calc. Conc.	Calc. Conc.
	27.91	1687.10
	27.46	1759.93
	24.65	1717.63
	26.38	1676.83
	26.41	1708.27
	25.63	1653.20
Mean	26.40	1700.49
SD	1.19	37.05
CV	4.50%	2.18%
Accuracy	88%	106%

Table 12a SFN in hCSF

Nominal Conc.	Low QC2<br>15.00 ng/mL	High QC4<br>1600.00 ng/mL
	Calc. Conc.	Calc. Conc.
	18.15	1779.06
	18.75	1781.21
	18.01	1704.71
	19.62	1774.27
	18.92	1851.68
	17.02	1754.47
Mean	18.41	1774.23
SD	0.89	47.53
CV	4.86%	2.68%
Accuracy	123%	111%

Table 12b SFN-NAC in hCSF

Nominal Conc.	Low QC2<br>15.00 ng/mL	High QC4<br>1600.00 ng/mL
	Calc. Conc.	Calc. Conc.
	13.62	1429.45
	13.76	1472.69
	13.31	1461.28
	13.02	1533.69
	13.28	1425.41
	14.12	1404.32
Mean	13.52	1454.47
SD	0.39	46.13
CV	2.92%	3.17%
Accuracy	90%	91%

Table 12c SFN-GSH in hCSF

Nominal Conc.	Low QC2<br>15.00 ng/mL	High QC4<br>1600.00 ng/mL
	Calc. Conc.	Calc. Conc.
	28.66	1673.27
	26.10	1723.51
	28.78	1780.28
	29.05	1829.37
	25.94	1732.19
	26.89	1590.39
Mean	27.57	1721.50
SD	1.42	83.34
CV	5.16%	4.84%
Accuracy	92%	108%

Table 13a Carryover Assessment for SFN

Sample ID	Peak Area	% Response of Mean LLOQ	Peak Area	% Response of Mean LLOQ	Peak Area	% Response of Mean LLOQ
	Run 6		Run 7		Run 8	
STD1A	20843	n/a	20670	n/a	24594	n/a
STD1B	17738	n/a	11967	n/a	18886	n/a
Mean LLOQ Response	19290		16318		21740	
Blank 1	3126	16.21%	2757	16.89%	2112	9.72%
Blank 2	0	0.00%	854	5.23%	3798	17.47%

Table 13b Carryover Assessment for SFN-NAC

Sample ID	Peak Area	% Response of Mean LLOQ	Peak Area	% Response of Mean LLOQ	Peak Area	% Response of Mean LLOQ
	Run 6		Run 7		Run 8	
STD1A	27609	n/a	28833	n/a	44185	n/a
STD1B	26172	n/a	31445	n/a	30494	n/a
Mean LLOQ Response	26890		30139		37340	
Blank 1	0	0.00%	0	0.00%	0	0.00%
Blank 2	0	0.00%	0	0.00%	0	0.00%

Table 13c Carryover Assessment for SFN-GSH

Sample ID	Peak Area	% Response of Mean LLOQ	Peak Area	% Response of Mean LLOQ	Peak Area	% Response of Mean LLOQ
	Run 6		Run 7		Run 8	
STD2A	202441	n/a	214517	n/a	326423	n/a
STD2B	223637	n/a	203482	n/a	231155	n/a
Mean LLOQ Response	213039		209000		278789	
Blank 1	13478	6.33%	13224	6.33%	16832	6.04%
Blank 2	14753	6.92%	0	0.00%	19981	7.17%

Table 14a Carryover Assessment for SFN IS

Sample ID	IS Peak Area	% Response of Mean IS in LLOQ	IS Peak Area	% Response of Mean IS in LLOQ	IS Peak Area	% Response of Mean IS in LLOQ
	Run 6		Run 7		Run 8	
STD1A	651420	n/a	520075	n/a	591608	n/a
STD1B	496029	n/a	390609	n/a	599493	n/a
Mean IS in LLOQ Response	573725		455342		595551	
Blank 1	0	0.00%	0	0.00%	0	0.00%
Blank 2	0	0.00%	0	0.00%	0	0.00%

Table 14b Carryover Assessment for SFN-NAC IS

Sample ID	IS Peak Area	% Response of Mean IS in LLOQ	IS Peak Area	% Response of Mean IS in LLOQ	IS Peak Area	% Response of Mean IS in LLOQ
	Run 6		Run 7		Run 8	
STD1A	259045	n/a	207963	n/a	337026	n/a
STD1B	246121	n/a	292279	n/a	266869	n/a
Mean IS in LLOQ Response	252583		250121		301948	
Blank 1	0	0.00%	0	0.00%	0	0.00%
Blank 2	0	0.00%	0	0.00%	0	0.00%

Table 14c Carryover Assessment for SFN-GSH IS

Sample ID	IS Peak Area	% Response of Mean IS in LLOQ	IS Peak Area	% Response of Mean IS in LLOQ	IS Peak Area	% Response of Mean IS in LLOQ
	Run 6		Run 7		Run 8	
STD2A	356124	n/a	235953	n/a	363698	n/a
STD2B	303476	n/a	283640	n/a	285511	n/a
Mean IS in LLOQ Response	329800		259797		324605	
Blank 1	0	0.00%	0	0.00%	0	0.00%
Blank 2	0	0.00%	0	0.00%	0	0.00%

Table 15a   24 Hour Re-Injection Reproducibility and Autosampler Stability for SFN

	Re-Run QCs (Run 10) Calculated Against	
	Original Cal Curve<br>Run 9	Re-Injected Cal Curve<br>Run 10
Low QC<br>15 ng/mL	14.97<br>15.06<br>14.94<br>15.24<br>16.06<br>15.04	13.97<br>14.06<br>13.93<br>14.23<br>15.02<br>14.03
Mean<br>SD<br>CV<br>Accuracy	15.22<br>0.42<br>2.79%<br>101%	14.21<br>0.41<br>2.90%<br>95%
Mid QC<br>800 ng/mL	785.14<br>771.53<br>800.23<br>797.14<br>785.35<br>819.59	760.98<br>747.77<br>775.61<br>772.61<br>761.18<br>794.39
Mean<br>SD<br>CV<br>Accuracy	793.16<br>16.48<br>2.08%<br>99%	768.76<br>15.98<br>2.08%<br>96%
High QC<br>1600 ng/mL	1525.37<br>1530.42<br>1550.80<br>1590.78<br>1615.92<br>1602.07	1478.94<br>1483.84<br>1503.61<br>1542.39<br>1566.77<br>1553.34
Mean<br>SD<br>CV<br>Accuracy	1569.23<br>38.71<br>2.47%<br>98%	1521.48<br>37.55<br>2.47%<br>95%

Table 15b 24 Hour Re-Injection Reproducibility and Autosampler Stability for SFN-NAC

	Re-Run QCs (Run 10) Calculated Against	
	Original Cal Curve Run 9	Re-Injected Cal Curve Run 10
Low QC 15 ng/mL	14.60	15.55
	14.31	15.24
	15.68	16.67
	15.40	16.39
	15.26	16.24
	15.87	16.87
Mean	15.19	16.16
SD	0.61	0.64
CV	4.04%	3.95%
Accuracy	101%	108%
Mid QC 800 ng/mL	840.50	874.64
	804.67	837.37
	830.26	863.98
	830.26	863.99
	878.04	913.68
	838.95	873.02
Mean	837.11	871.11
SD	23.82	24.77
CV	2.85%	2.84%
Accuracy	105%	109%
High QC 1600 ng/mL	1651.60	1718.32
	1660.30	1727.38
	1611.82	1676.94
	1684.72	1752.78
	1660.78	1727.87
	1646.21	1712.72
Mean	1652.57	1719.34
SD	23.93	24.89
CV	1.45%	1.45%
Accuracy	103%	107%

Table 15c 24 Hour Re-Injection Reproducibility and Autosampler Stability for SFN-GSH

	Re-Run QCs (Run 10) Calculated Against	
	Original Cal Curve Run 9	Re-Injected Cal Curve Run 10
Low QC 30 ng/mL	31.34	32.29
	27.96	28.79
	25.96	26.73
	28.38	29.24
	29.73	30.63
	30.87	31.81
Mean	29.04	29.91
SD	2.01	2.08
CV	6.92%	6.95%
Accuracy	97%	100%
Mid QC 800 ng/mL	781.47	807.69
	729.84	754.32
	795.68	822.38
	806.76	833.83
	843.77	872.09
	873.00	902.30
Mean	805.09	832.10
SD	49.84	51.52
CV	6.19%	6.19%
Accuracy	101%	104%
High QC 1600 ng/mL	1640.91	1696.08
	1636.30	1691.32
	1606.79	1660.81
	1739.72	1798.22
	1775.89	1835.61
	1711.34	1768.88
Mean	1685.16	1741.82
SD	66.91	69.16
CV	3.97%	3.97%
Accuracy	105%	109%

Table 16a 2.5 Hour Benchtop Stability for SFN in aCSF

	Low QC 15 ng/mL	High QC 1600 ng/mL	Dil QC 8000 ng/mL
	Calc. Conc.	Calc. Conc.	Calc. Conc.
	14.20	1539.58	8320.62
	13.99	1551.86	8098.60
	13.10	1733.70	8257.20
Mean	13.76	1608.38	8225.47
SD	0.58	108.71	114.36
CV	4.25%	6.76%	1.39%
Accuracy	92%	101%	103%

Table 16b 2.5 Hour Benchtop Stability for SFN-NAC in aCSF

	Low QC 15 ng/mL	High QC 1600 ng/mL	Dil QC 8000 ng/mL
	Calc. Conc.	Calc. Conc.	Calc. Conc.
	15.14	1521.89	8000.07
	14.23	1534.86	8430.00
	15.77	1494.40	8224.40
Mean	15.04	1517.05	8218.16
SD	0.78	20.66	215.03
CV	5.15%	1.36%	2.62%
Accuracy	100%	95%	103%

Table 16c 2.5 Hour Benchtop Stability for SFN-GSH in aCSF

	Low QC 30 ng/mL	High QC 1600 ng/mL	Dil QC 8000 ng/mL
	Calc. Conc.	Calc. Conc.	Calc. Conc.
	30.84	1726.74	7886.39
	27.20	1755.14	7811.55
	27.96	1726.90	7456.60
Mean	28.67	1736.26	7718.18
SD	1.92	16.35	229.60
CV	6.70%	0.94%	2.97%
Accuracy	96%	109%	96%

Table 17a   2.5 Hour Benchtop Stability for SFN in hCSF

	Low QC 15 ng/mL	High QC 1600 ng/mL	Dil QC 8000 ng/mL
	Calc. Conc.	Calc. Conc.	Calc. Conc.
	16.69	1775.18	9064.00
	17.33	1834.50	9326.66
	17.65	1787.95	9463.89
Mean	17.22	1799.21	9284.85
SD	0.49	31.22	203.20
CV	2.83%	1.74%	2.19%
Accuracy	115%	112%	116%

Table 17b   2.5 Hour Benchtop Stability for SFN-NAC in hCSF

	Low QC 15 ng/mL	High QC 1600 ng/mL	Dil QC 8000 ng/mL
	Calc. Conc.	Calc. Conc.	Calc. Conc.
	14.92	1388.03	8264.88
	13.79	1326.02	8042.20
	14.47	1295.35	7648.96
Mean	14.39	1336.46	7985.34
SD	0.57	47.21	311.87
CV	3.96%	3.53%	3.91%
Accuracy	96%	84%	100%

Table 17c   2.5 Hour Benchtop Stability for SFN-GSH in hCSF

	Low QC 30 ng/mL	High QC 1600 ng/mL	Dil QC 8000 ng/mL
	Calc. Conc.	Calc. Conc.	Calc. Conc.
	27.32	1570.97	7159.70
	25.46	1500.16	6385.47
	25.99	1434.51	6517.74
Mean	26.26	1501.88	6687.64
SD	0.96	68.25	414.13
CV	3.65%	4.54%	6.19%
Accuracy	88%	94%	84%

Table 18a 2-Cycle Freeze (-80°C) /Thaw Stability for SFN in aCSF

	Low QC 15 ng/mL	High QC 1600 ng/mL	Dil QC 8000 ng/mL
	Calc. Conc.	Calc. Conc.	Calc. Conc.
	13.92	1522.03	7862.26
	13.00	1506.33	7894.11
	13.96	1477.24	7855.56
Mean	13.62	1501.87	7870.64
SD	0.54	22.73	20.60
CV	3.96%	1.51%	0.26%
Accuracy	91%	94%	98%

Table 18b 2-Cycle Freeze (-80°C) /Thaw Stability for SFN-NAC in aCSF

	Low QC 15 ng/mL	High QC 1600 ng/mL	Dil QC 8000 ng/mL
	Calc. Conc.	Calc. Conc.	Calc. Conc.
	15.30	1474.13	8115.01
	14.63	1565.50	8258.23
	14.84	1512.80	8069.42
Mean	14.93	1517.48	8147.56
SD	0.34	45.86	98.52
CV	2.30%	3.02%	1.21%
Accuracy	100%	95%	102%

Table 18c 2-Cycle Freeze (-80°C) /Thaw Stability for SFN-GSH in aCSF

	Low QC 30 ng/mL	High QC 1600 ng/mL	Dil QC 8000 ng/mL
	Calc. Conc.	Calc. Conc.	Calc. Conc.
	29.15	1850.77	8675.05
	28.33	1927.60	8904.02
	29.65	1942.89	8832.09
Mean	29.04	1907.09	8803.72
SD	0.67	49.37	117.09
CV	2.30%	2.59%	1.33%
Accuracy	97%	119%	110%

Table 19a 2-Cycle Freeze (-80°C) /Thaw Stability for SFN in hCSF

	Low QC 15 ng/mL	High QC 1600 ng/mL	Dil QC 8000 ng/mL
	Calc. Conc.	Calc. Conc.	Calc. Conc.
	16.14	1818.38	8849.51
	15.59	1698.53	8400.92
	16.91	1722.48	8949.32
Mean	16.21	1746.46	8733.25
SD	0.66	63.42	292.10
CV	4.07%	3.63%	3.34%
Accuracy	108%	109%	109%

Table 19b 2-Cycle Freeze (-80°C) /Thaw Stability for SFN-NAC in hCSF

	Low QC 15 ng/mL	High QC 1600 ng/mL	Dil QC 8000 ng/mL
	Calc. Conc.	Calc. Conc.	Calc. Conc.
	15.77	1347.00	8003.71
	13.51	1376.53	7465.90
	13.80	1367.36	7433.52
Mean	14.36	1363.63	7634.38
SD	1.23	15.11	320.26
CV	8.59%	1.11%	4.19%
Accuracy	96%	85%	95%

Table 19c 2-Cycle Freeze (-80°C) /Thaw Stability for SFN-GSH in hCSF

	Low QC 30 ng/mL	High QC 1600 ng/mL	Dil QC 8000 ng/mL
	Calc. Conc.	Calc. Conc.	Calc. Conc.
	33.07	1883.67	7805.27
	30.80	1913.95	7199.21
	30.14	1872.70	7478.03
Mean	31.33	1890.11	7494.17
SD	1.54	21.36	303.35
CV	4.90%	1.13%	4.05%
Accuracy	104%	118%	94%

Table 20a **Interference Screens Relative to SFN**

Sample ID	SFN Peak Area	% Response compared to Mean
Low QC Q1A	100129	
Low QC Q1B	101336	
Low QC Q1C	106556	
Mean	102674	
SFN-NAC Low QC		
Q1A	624	0.61%
Q1B	597	0.58%
Q1C	0	0.00%
SFN-GSH Low QC		
Q1A	4568	4.45%
Q1B	0	0.00%
Q1C	5902	5.75%
SFN-NAC High QC		
Q1A	194151	189.09%
Q1B	210463	204.98%
Q1C	219728	214.01%
SFN-GSH High QC		
Q1A	396121	385.81%
Q1B	412685	401.94%
Q1C	405888	395.32%

Table 20b Interference Screens Relative to SFN-NAC

Sample ID	SFN-NAC Peak Area	% Response compared to Mean
Low QC Q1A	61815	
Low QC Q1B	61538	
Low QC Q1C	62656	
Mean	62003	
SFN Low QC		
Q1A	0	0.00%
Q1B	0	0.00%
Q1C	0	0.00%
SFN-GSH Low QC		
Q1A	0	0.00%
Q1B	0	0.00%
Q1C	0	0.00%
SFN High QC		
Q1A	0	0.00%
Q1B	0	0.00%
Q1C	5044	8.14%
SFN-GSH High QC		
Q1A	5448	8.79%
Q1B	0	0.00%
Q1C	0	0.00%

Table 20c Interference Screens Relative to SFN-GSH

Sample ID	SFN-GSH Peak Area	% Response compared to Mean
Low QC Q1A	69478	
Low QC Q1B	83480	
Low QC Q1C	76819	
Mean	76592	
SFN Low QC		
Q1A	0	0.00%
Q1B	5819	7.60%
Q1C	0	0.00%
SFN-NAC Low QC		
Q1A	0	0.00%
Q1B	5067	6.62%
Q1C	0	0.00%
SFN High QC		
Q1A	6952	9.08%
Q1B	8354	10.91%
Q1C	0	0.00%
SFN-NAC High QC		
Q1A	9719	12.69%
Q1B	7210	9.41%
Q1C	0	0.00%

8 FIGURES

Figure 1a Standard Curve of SFN in Artificial Cerebrospinal Fluid, P&A Run 1

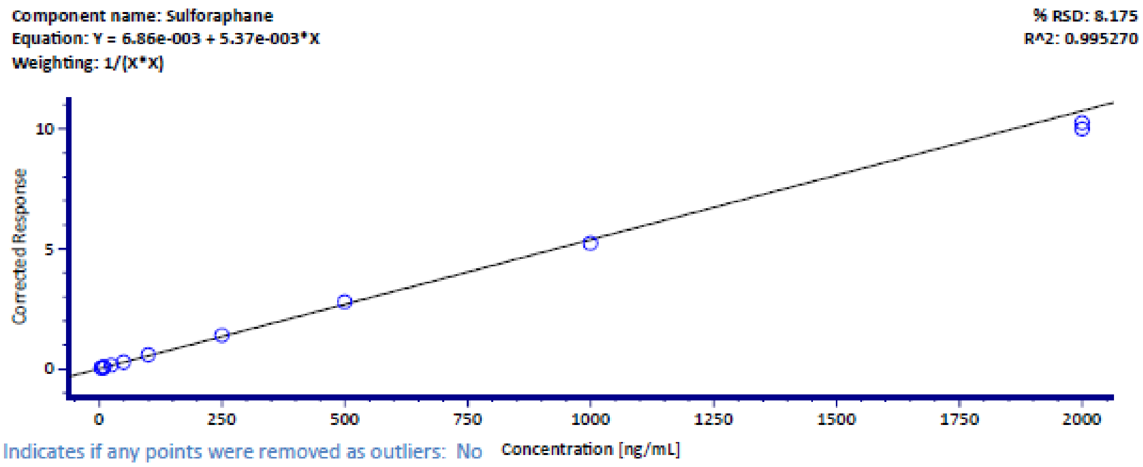

Component name: Sulforaphane

.	Sample name	Description	Rep	Sample Position	Response	IS Response	Response Ratio	Known Conc	Calc Conc	% Dev	Manual
1	6STD1A	STD	1	2:A,12	20843	651420	0.032	5.00	4.68	-6.41	None
2	6STD1B	STD	1	2:B,1	17738	496029	0.036	5.00	5.38	7.60	None
3	6STD2A	STD	1	2:B,2	38958	635346	0.061	10.00	10.14	1.38	None
4	6STD2B	STD	1	2:B,3	29583	522616	0.057	10.00	9.26	-7.39	None
5	6STD3	STD	1	2:B,4	82163	542957	0.151	25.00	26.89	7.57	None
6	6STD4	STD	1	2:B,5	152506	557739	0.273	50.00	49.63	-0.75	None
7	6STD5	STD	1	2:B,6	348947	602137	0.580	100.00	106.60	6.60	None
8	6STD6	STD	1	2:B,7	818377	591103	1.384	250.00	256.46	2.58	None
9	6STD7	STD	1	2:B,8	1432582	514145	2.786	500.00	517.42	3.48	None
10	6STD8	STD	1	2:B,9	2933264	561629	5.223	1000.00	970.99	-2.90	None
11	6STD9A	STD	1	2:B,10	5235129	510959	10.246	2000.00	1906.04	-4.70	None
12	6STD9B	STD	1	2:B,11	4343153	434762	9.990	2000.00	1858.39	-7.08	None

Figure 1b Standard Curve of SFN in Artificial Cerebrospinal Fluid, P&A Run 2

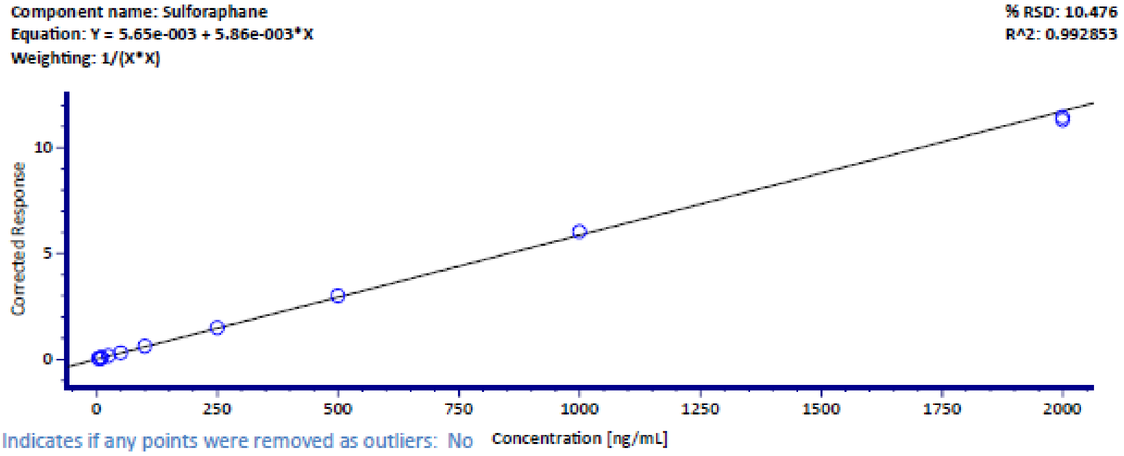

Indicates if any points were removed as outliers: No

Component name: Sulforaphane

.	Sample name	Description	Rep	Sample Position	Response	IS Response	Response Ratio	Known Conc	Calc Conc	% Dev	Manual
1	7STD1A	STD	1	2:A,12	20670	520075	0.040	5.00	5.81	16.26	None
2	7STD1B	STD	1	2:B,1	11967	390609	0.031	5.00	4.26	-14.79	None
3	7STD2A	STD	1	2:B,2	30399	483252	0.063	10.00	9.76	-2.38	None
4	7STD2B	STD	1	2:B,3	22772	358043	0.064	10.00	9.88	-1.19	None
5	7STD3	STD	1	2:B,4	69986	453099	0.154	25.00	25.37	1.49	None
6	7STD4	STD	1	2:B,5	134961	460153	0.293	50.00	49.05	-1.91	None
7	7STD5	STD	1	2:B,6	264116	431140	0.613	100.00	103.49	3.49	None
8	7STD6	STD	1	2:B,7	652110	440491	1.480	250.00	251.46	0.58	None
9	7STD7	STD	1	2:B,8	1161772	387861	2.995	500.00	509.76	1.95	None
10	7STD8	STD	1	2:B,9	2364141	392244	6.027	1000.00	1026.72	2.67	None
11	7STD9A	STD	1	2:B,10	3684435	321817	11.449	2000.00	1951.15	-2.44	None
12	7STD9B	STD	1	2:B,11	3986166	352884	11.296	2000.00	1925.08	-3.75	None

Figure 1c Standard Curve of SFN in Artificial Cerebrospinal Fluid, P&A Run 3

Component name: Sulforaphane
Equation: Y = 6.10e-003 + 6.13e-003*X
Weighting: 1/(X*X)

% RSD: 12.679
R^2: 0.989477

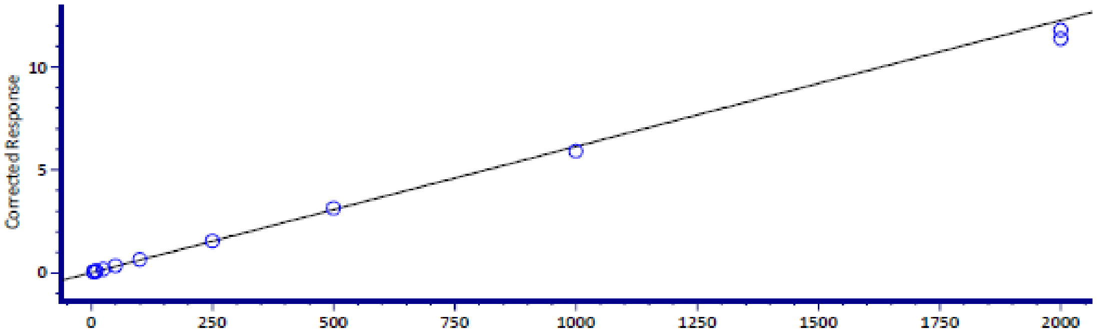

Indicates if any points were removed as outliers: No

Component name: Sulforaphane

.	Sample name	Description	Rep	Sample Position	Response	IS Response	Response Ratio	Known Conc	Calc Conc	% Dev	Manual
1	8STD1A	STD	1	2:A,12	24594	591608	0.042	5.00	5.79	15.73	None
2	8STD1B	STD	1	2:B,1	18886	599493	0.032	5.00	4.14	-17.12	None
3	8STD2A	STD	1	2:B,2	44212	621056	0.071	10.00	10.62	6.19	None
4	8STD2B	STD	1	2:B,3	37455	594365	0.063	10.00	9.29	-7.15	None
5	8STD3	STD	1	2:B,4	98436	586450	0.168	25.00	26.39	5.55	None
6	8STD4	STD	1	2:B,5	183719	555973	0.330	50.00	52.92	5.83	None
7	8STD5	STD	1	2:B,6	375320	583475	0.643	100.00	103.95	3.95	None
8	8STD6	STD	1	2:B,7	787194	510677	1.541	250.00	250.49	0.20	None
9	8STD7	STD	1	2:B,8	1495760	478263	3.127	500.00	509.24	1.85	None
10	8STD8	STD	1	2:B,9	2772807	469504	5.906	1000.00	962.52	-3.75	None
11	8STD9A	STD	1	2:B,10	5058987	429318	11.784	2000.00	1921.49	-3.93	None
12	8STD9B	STD	1	2:B,11	5637221	496099	11.363	2000.00	1852.85	-7.36	None

Figure 2a Standard Curve of SFN-NAC in Artificial Cerebrospinal Fluid, P&A Run 1

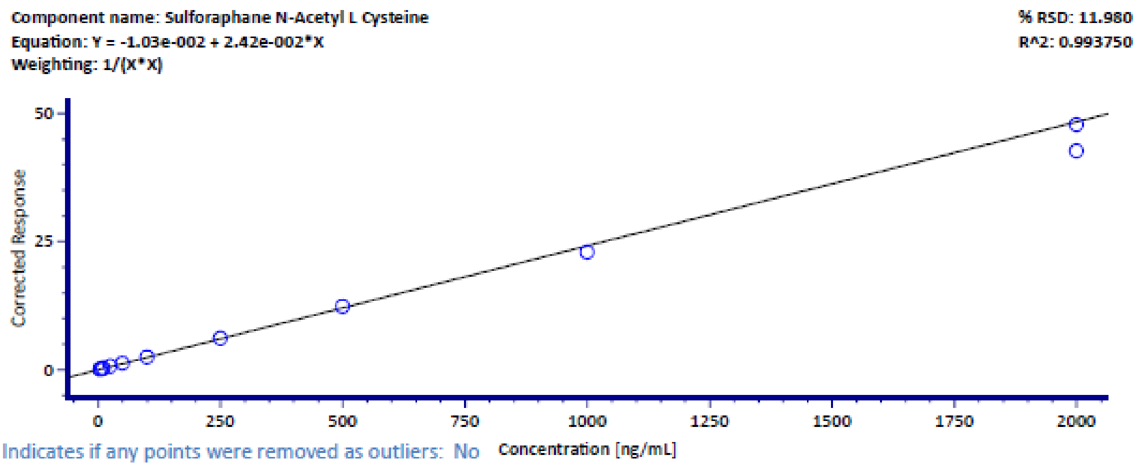

Indicates if any points were removed as outliers: No

Component name: Sulforaphane N-Acetyl L Cysteine

.	Sample name	Description	Rep	Sample Position	Response	IS Response	Response Ratio	Known Conc	Calc Conc	% Dev	Manual
1	6STD1A	STD	1	2:A,12	27609	259045	0.107	5.00	4.83	-3.39	None
2	6STD1B	STD	1	2:B,1	26172	246121	0.106	5.00	4.82	-3.60	None
3	6STD2A	STD	1	2:B,2	79642	356124	0.224	10.00	9.67	-3.32	None
4	6STD2B	STD	1	2:B,3	79892	303476	0.263	10.00	11.31	13.06	None
5	6STD3	STD	1	2:B,4	193450	297421	0.650	25.00	27.31	9.23	None
6	6STD4	STD	1	2:B,5	396795	322932	1.229	50.00	51.21	2.42	None
7	6STD5	STD	1	2:B,6	805260	330698	2.435	100.00	101.06	1.06	None
8	6STD6	STD	1	2:B,7	2082294	341905	6.090	250.00	252.13	0.85	None
9	6STD7	STD	1	2:B,8	3901468	316663	12.321	500.00	509.63	1.93	None
10	6STD8	STD	1	2:B,9	8193942	357480	22.921	1000.00	947.76	-5.22	None
11	6STD9A	STD	1	2:B,10	13978974	327794	42.646	2000.00	1762.95	-11.85	None
12	6STD9B	STD	1	2:B,11	14718309	307801	47.818	2000.00	1976.71	-1.16	None

Figure 2b **Standard Curve of SFN-NAC in Artificial Cerebrospinal Fluid, P&A Run 2**

Component name: Sulforaphane N-Acetyl L Cysteine
Equation: Y = -9.52e-003 + 2.64e-002*X
Weighting: 1/(X*X)

% RSD: 14.736
R^2: 0.990379

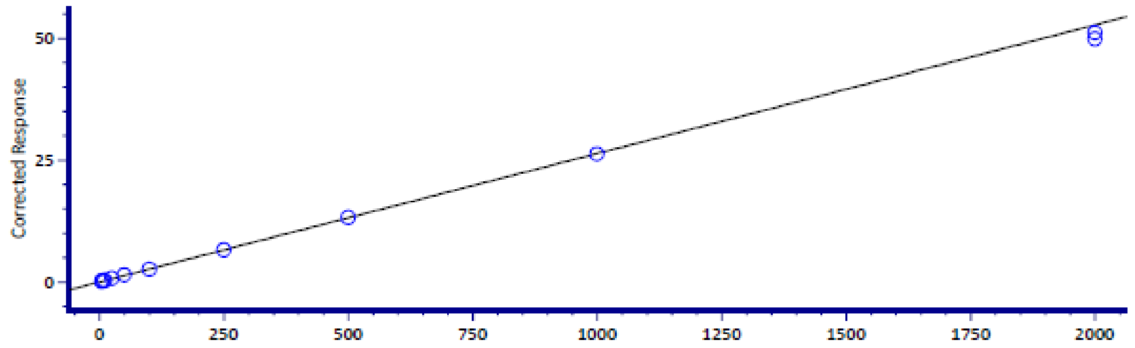

Indicates if any points were removed as outliers: No

Component name: Sulforaphane N-Acetyl L Cysteine

.	Sample name	Description	Rep	Sample Position	Response	IS Response	Response Ratio	Known Conc	Calc Conc	% Dev	Manual
1	7STD1A	STD	1	2:A,12	28833	207963	0.139	5.00	5.61	12.29	None
2	7STD1B	STD	1	2:B,1	31445	292279	0.108	5.00	4.44	-11.25	None
3	7STD2A	STD	1	2:B,2	50999	235953	0.216	10.00	8.55	-14.49	None
4	7STD2B	STD	1	2:B,3	77972	283640	0.275	10.00	10.78	7.77	None
5	7STD3	STD	1	2:B,4	167235	238545	0.701	25.00	26.93	7.70	None
6	7STD4	STD	1	2:B,5	335290	234942	1.427	50.00	54.44	8.88	None
7	7STD5	STD	1	2:B,6	684762	264444	2.589	100.00	98.48	-1.52	None
8	7STD6	STD	1	2:B,7	1932919	294801	6.557	250.00	248.81	-0.47	None
9	7STD7	STD	1	2:B,8	3773403	285654	13.210	500.00	500.92	0.18	None
10	7STD8	STD	1	2:B,9	7436664	283529	26.229	1000.00	994.26	-0.57	None
11	7STD9A	STD	1	2:B,10	12228210	238916	51.182	2000.00	1939.80	-3.01	None
12	7STD9B	STD	1	2:B,11	15286270	306565	49.863	2000.00	1889.82	-5.51	None

Figure 2c Standard Curve of SFN-NAC in Artificial Cerebrospinal Fluid, P&A Run 3

Component name: Sulforaphane N-Acetyl L Cysteine
Equation: Y = 9.04e-003 + 2.38e-002*X
Weighting: 1/(X*X)

% RSD: 10.445
R^2: 0.993938

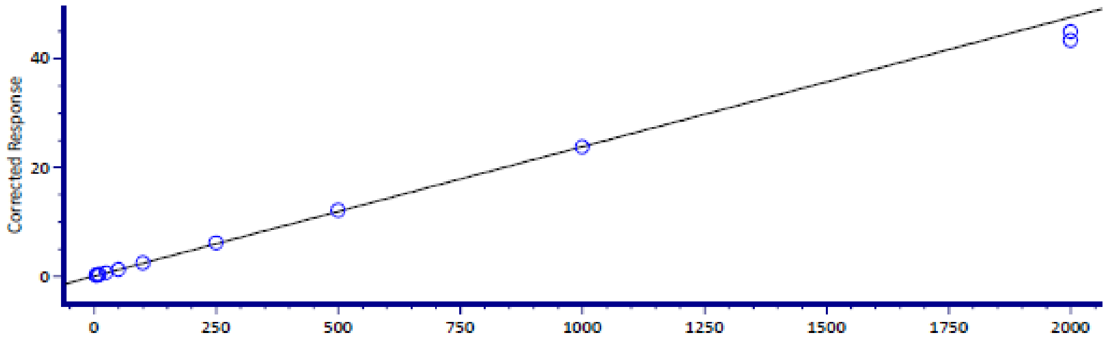

Indicates if any points were removed as outliers: No

Component name: Sulforaphane N-Acetyl L Cysteine

.	Sample name	Description	Rep	Sample Position	Response	IS Response	Response Ratio	Known Conc	Calc Conc	% Dev	Manual
1	8STD1A	STD	1	2:A,12	44185	337026	0.131	5.00	5.13	2.53	None
2	8STD1B	STD	1	2:B,1	30494	266869	0.114	5.00	4.42	-11.62	None
3	8STD2A	STD	1	2:B,2	94606	363698	0.260	10.00	10.54	5.45	None
4	8STD2B	STD	1	2:B,3	79144	285511	0.277	10.00	11.26	12.62	None
5	8STD3	STD	1	2:B,4	216669	362456	0.598	25.00	24.73	-1.10	None
6	8STD4	STD	1	2:B,5	391225	321046	1.219	50.00	50.80	1.59	None
7	8STD5	STD	1	2:B,6	905448	371393	2.438	100.00	102.01	2.01	None
8	8STD6	STD	1	2:B,7	2051103	337135	6.084	250.00	255.12	2.05	None
9	8STD7	STD	1	2:B,8	3859667	319402	12.084	500.00	507.11	1.42	None
10	8STD8	STD	1	2:B,9	7290146	307005	23.746	1000.00	996.87	-0.31	None
11	8STD9A	STD	1	2:B,10	12621699	291309	43.328	2000.00	1819.23	-9.04	None
12	8STD9B	STD	1	2:B,11	12680652	282001	44.967	2000.00	1888.06	-5.60	None

Figure 3a Standard Curve of SFN-GSH in Artificial Cerebrospinal Fluid, P&A Run 1

Component name: Sulforaphane Glutathione
Equation: Y = 7.03e-002 + 5.97e-002*X
Weighting: 1/(X*X)

% RSD: 14.281
R^2: 0.988169

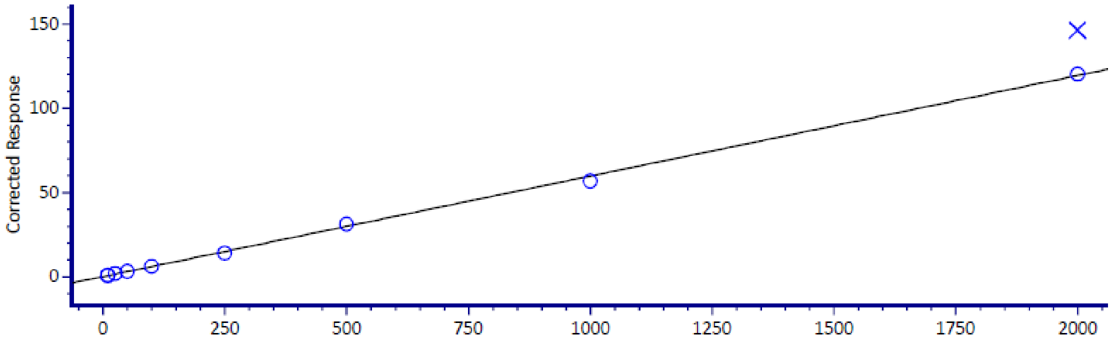

Indicates if any points were removed as outliers: Yes

Component name: Sulforaphane Glutathione

.	Sample name	Description	Rep	Sample Position	Response	IS Response	Response Ratio	Known Conc	Calc Conc	% Dev	Manual
1	6STD1A	STD	1	2:A,12	109347	259045	0.422		5.90		None
2	6STD1B	STD	1	2:B,1	117300	246121	0.477		6.81		None
3	6STD2A	STD	1	2:B,2	202441	356124	0.568	10.00	8.35	-16.51	None
4	6STD2B	STD	1	2:B,3	223637	303476	0.737	10.00	11.17	11.72	None
5	6STD3	STD	1	2:B,4	521303	297421	1.753	25.00	28.20	12.78	None
6	6STD4	STD	1	2:B,5	985571	322932	3.052	50.00	49.97	-0.06	None
7	6STD5	STD	1	2:B,6	1983288	330698	5.997	100.00	99.33	-0.67	None
8	6STD6	STD	1	2:B,7	4782094	341905	13.987	250.00	233.22	-6.71	None
9	6STD7	STD	1	2:B,8	9837114	316663	31.065	500.00	519.43	3.89	None
10	6STD8	STD	1	2:B,9	20263915	357480	56.685	1000.00	948.80	-5.12	None
11	6STD9A	STD	1	2:B,10	39411724	327794	120.233	2000.00	2013.79	0.69	None
12	6STD9B	STD	1	2:B,11	45020466	307801	146.265	2000.00	2450.05	22.50	Manual Calibration

Figure 3b Standard Curve of SFN-GSH in Artificial Cerebrospinal Fluid, P&A Run 2

Component name: Sulforaphane Glutathione
Equation: Y = -2.77e-002 + 8.53e-002*X
Weighting: 1/(X*X)

% RSD: 16.483
R^2: 0.989047

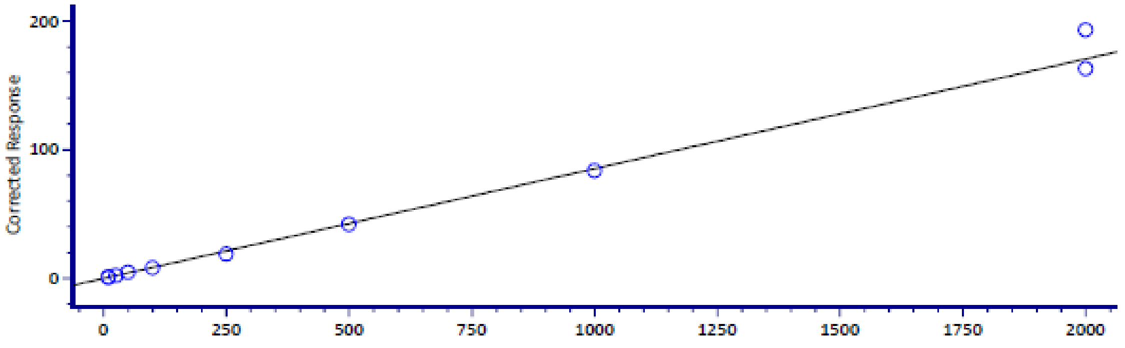

Indicates if any points were removed as outliers: No

Component name: Sulforaphane Glutathione

.	Sample name	Description	Rep	Sample Position	Response	IS Response	Response Ratio	Known Conc	Calc Conc	% Dev	Manual
1	7STD1A	STD	1	2:A,12	119443	207963	0.574		7.06		None
2	7STD1B	STD	1	2:B,1	122288	292279	0.418		5.23		None
3	7STD2A	STD	1	2:B,2	214517	235953	0.909	10.00	10.99	9.89	None
4	7STD2B	STD	1	2:B,3	203482	283640	0.717	10.00	8.74	-12.60	None
5	7STD3	STD	1	2:B,4	523173	238545	2.193	25.00	26.05	4.20	None
6	7STD4	STD	1	2:B,5	1091149	234942	4.644	50.00	54.80	9.60	None
7	7STD5	STD	1	2:B,6	2153782	264444	8.145	100.00	95.86	-4.14	None
8	7STD6	STD	1	2:B,7	5539910	294801	18.792	250.00	220.75	-11.70	None
9	7STD7	STD	1	2:B,8	11952416	285654	41.842	500.00	491.11	-1.78	None
10	7STD8	STD	1	2:B,9	23660083	283529	83.449	1000.00	979.13	-2.09	None
11	7STD9A	STD	1	2:B,10	46076118	238916	192.855	2000.00	2262.41	13.12	None
12	7STD9B	STD	1	2:B,11	49906397	306565	162.792	2000.00	1909.79	-4.51	None

Figure 3c Standard Curve of SFN-GSH in Artificial Cerebrospinal Fluid, P&A Run 3

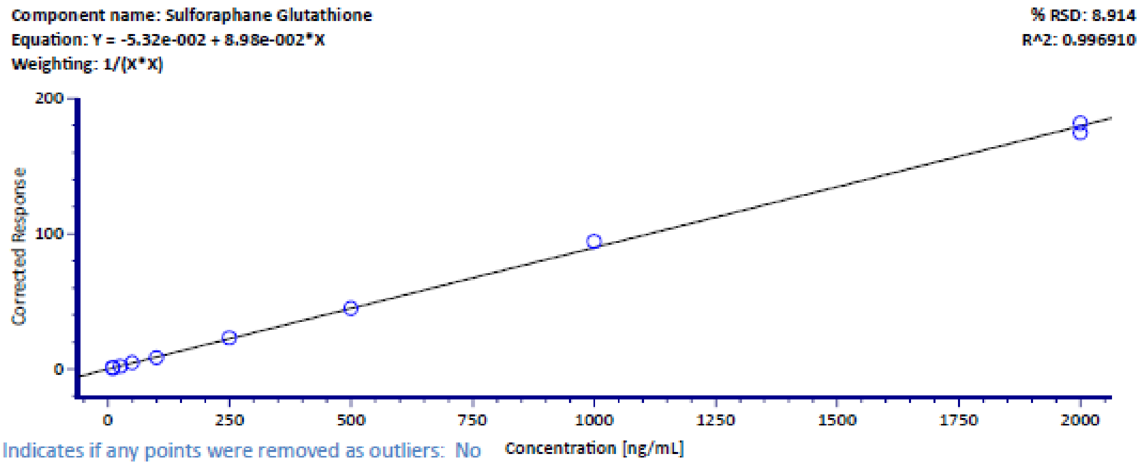

Component name: Sulforaphane Glutathione

.	Sample name	Description	Rep	Sample Position	Response	IS Response	Response Ratio	Known Conc	Calc Conc	% Dev	Manual
1	8STD1A	STD	1	2:A,12	165239	337026	0.490		6.05		None
2	8STD1B	STD	1	2:B,1	125195	266869	0.469		5.81		None
3	8STD2A	STD	1	2:B,2	326423	363698	0.898	10.00	10.58	5.82	None
4	8STD2B	STD	1	2:B,3	231155	285511	0.810	10.00	9.60	-3.97	None
5	8STD3	STD	1	2:B,4	749435	362456	2.068	25.00	23.61	-5.57	None
6	8STD4	STD	1	2:B,5	1493808	321046	4.653	50.00	52.38	4.77	None
7	8STD5	STD	1	2:B,6	3070059	371393	8.266	100.00	92.60	-7.40	None
8	8STD6	STD	1	2:B,7	7786101	337135	23.095	250.00	257.66	3.06	None
9	8STD7	STD	1	2:B,8	14327021	319402	44.856	500.00	499.87	-0.03	None
10	8STD8	STD	1	2:B,9	28945464	307005	94.283	1000.00	1050.04	5.00	None
11	8STD9A	STD	1	2:B,10	52917789	291309	181.655	2000.00	2022.55	1.13	None
12	8STD9B	STD	1	2:B,11	49230194	282001	174.575	2000.00	1943.74	-2.81	None

Figure 4 Representative Chromatogram of a Human Cerebrospinal Fluid Blank

Sample Information

Item name:	8SEL2A	Sample type:	Reagent blank
Description:	Selectivity	Sample position:	2:F,7
Acquisition start time:	Apr 27, 2016 18:45:41 GMT Daylight Time	E Cord ID:	

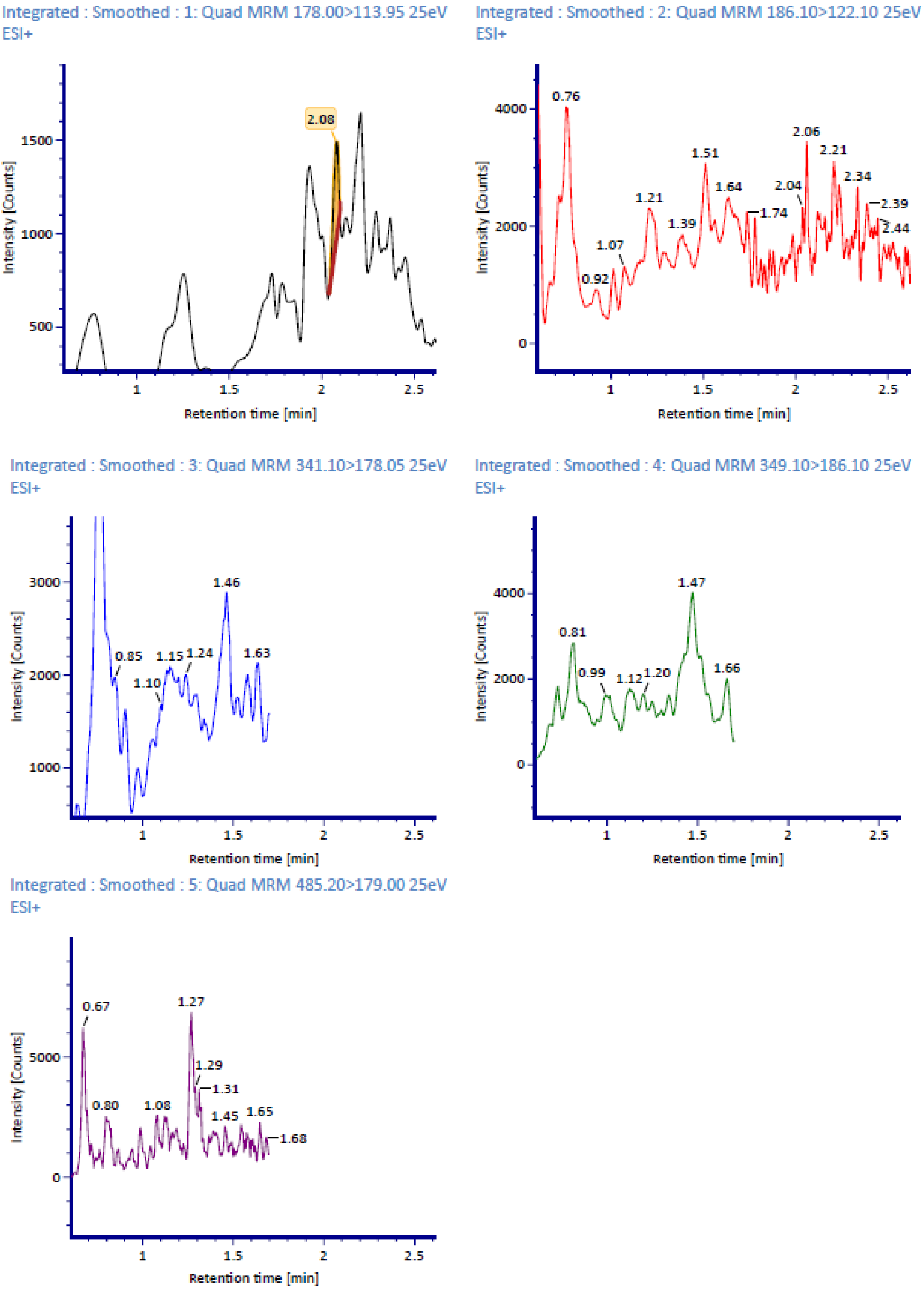

Figure 5 **Representative Chromatogram of an LLOQ Calibration Standard for SFN and SFN-NAC in Artificial Cerebrospinal Fluid**

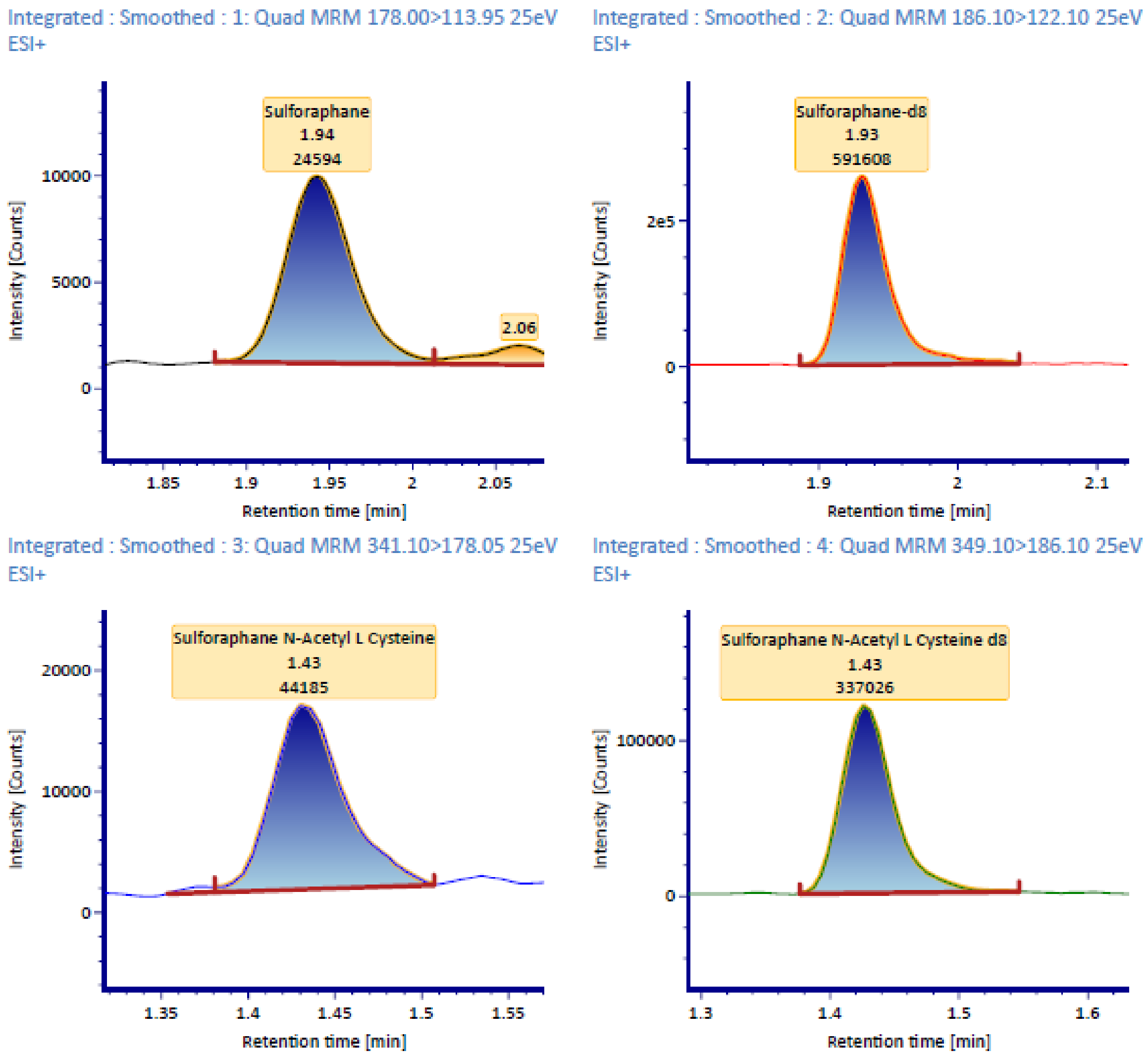

Figure 6 **Representative Chromatogram of an LLOQ Calibration Standard for SFN-GSH in Artificial Cerebrospinal Fluid**

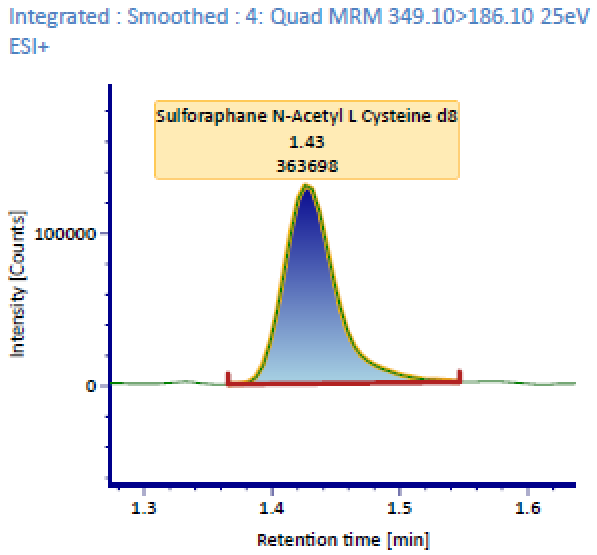

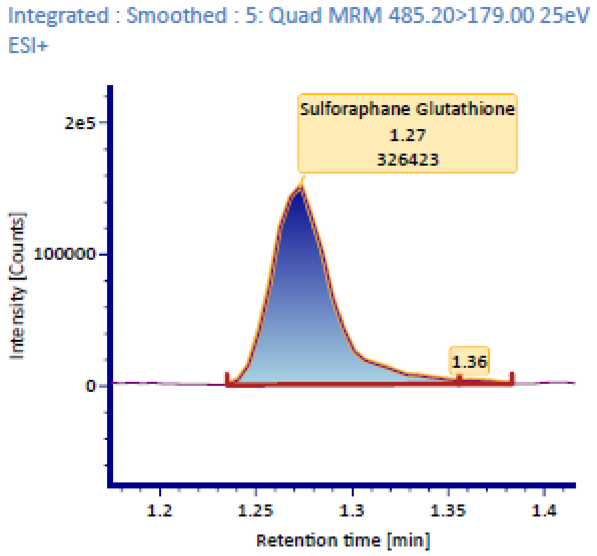

Figure 7 **Representative Chromatogram of a ULOQ Calibration Standard for SFN, SFN-NAC and SFN-GSH in Artificial Cerebrospinal Fluid**

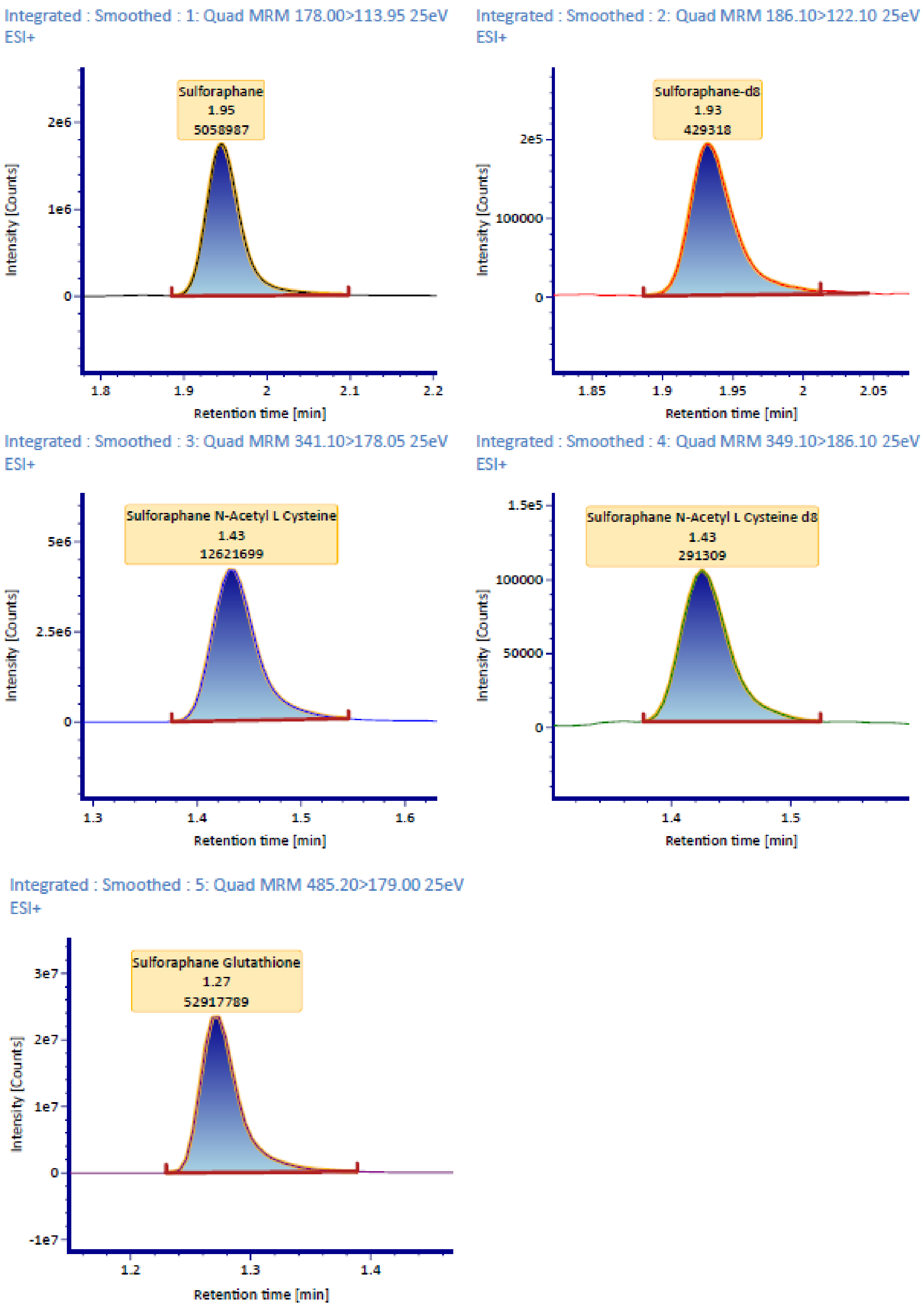

9 AMENDMENT HISTORY

As of the report issue date, no amendments have been issued for this report.

10 APPENDICES

Appendix 1 Bioanalytical Method

METHOD FOR THE DETERMINATION OF D,L SULFORAPHANE, D,L SULFORAPHANE GLUTATHIONE AND D,L SULFORAPHANE N-ACETYL CYSTEINE IN HUMAN CEREBROSPINAL FLUID (CSF) BY LC-MS/MS USING ARTIFICIAL CSF AS A SURROGATE MATRIX

Date	Signed By Reviewer:
25APR2016	[signature]

Date	Signed by Approver:
25APR2016	[signature]
Justification	I approve this document for content

Date	Signed by Project Leader:
Justification	I approve this method for use with Project No.:

Contents

1 INTRODUCTION 3
2 MATERIALS AND EQUIPMENT 3
2.1 Analytical Standards 3
2.2 Reagents and Matrix 4
2.3 Consumables 4
3 REAGENT PREPARATION 5
3.1 Mobile Phase A - 0.01% Aqueous Formic Acid 5
3.2 Mobile Phase B - Acetonitrile 5
3.3 Sample Manager Wash 5
3.4 Seal/Purge Wash 5
3.5 Purge Wash Solution (for infusion) 5
3.6 10 mM Ammonium Acetate Solution 5
3.7 10 mM Ammonium Acetate/MeCN (50:50) 5
3.8 0.5M Citric Acid 5
3.9 Solution A for Artificial CSF 6
3.10 Solution B for Artificial CSF 6
3.11 Artificial CSF 6
3.12 Stabilised CSF 6
4 D,L SULFORAPHANE, D,L SULFORAPHANE GLUTATHIONE AND D,L SULFORAPHANE N-ACETYL CYSTEINE SOLUTION PREPARATION FOR CALIBRATION STANDARDS 6
5 CALIBRATION STANDARD PREPARATION 7
6 D,L SULFORAPHANE, D,L SULFORAPHANE GLUTATHIONE AND D,L SULFORAPHANE N-ACETYL CYSTEINE SOLUTION PREPARATION FOR QUALITY CONTROL SAMPLES 7
7 BULK QUALITY CONTROL SAMPLES 8
8 INTERNAL STANDARDS 8
9 PRE-EXTRACTION PROCEDURE 9
10 EXTRACTION PROCEDURE 10
11 SYSTEM SUITABILITY 10
12 LC-MS/MS METHOD 11
13 AMENDMENT HISTORY 11
Appendix 1 12

1 INTRODUCTION

CSF extracts are analysed for D,L Sulforaphane (SFN), D,L Sulforaphane glutathione (SFN-GSH) & D,L Sulforaphane N-acetyl cysteine (SFN-NAC) by LC-MS/MS. D,L Sulforaphane-d8 and D,L-Sulforaphane N-acetyl cysteine-d8 sodium salt are added as internal standards. The calibration range is between 5 and 2000 ng/mL for D,L Sulforaphane and D,L Sulforaphane N-acetyl cysteine, and between 10 and 2000 ng/mL for D,L Sulforaphane glutathione.

Alternative preparations are allowed and must be fully documented.
Equivalent substitutions may be made.
Adjustable pipettes are used in this method.
Unless otherwise specified, all volumetric glassware is Class A.
Weights and volumes may be scaled as needed.
Alternate volumes of solutions and quality control samples may be prepared as needed.
Purity and freebase conversion will vary depending on drug standard lot (refer to certificate of analysis).
Scan functions may differ slightly upon infusion to the instrument.
The suppliers of reagents and matrix can differ from those indicated in the method, but the grade used must be comparable to that stated.

2 MATERIALS AND EQUIPMENT

2.1 Analytical Standards

D,L-Sulforaphane (SFN)

Empirical Formula:	$C_6H_{11}NOS_2$
Molecular Weight:	177.29

D,L-Sulforaphane glutathione (SFN-GSH)

Empirical Formula:	$C_{16}H_{28}N_4O_7S_3$
Molecular Weight:	484.61

D,L-Sulforaphane N-acetyl cysteine (SFN-NAC)

Empirical Formula:	$C_{11}H_{20}N_2O_4S_3$
Molecular Weight:	340.49

D,L-Sulforaphane-d8 (Internal Standard)

Empirical Formula: $C_6H_3D_8NOS_2$
Molecular Weight: 185.34

D,L-Sulforaphane N-acetyl cysteine-d8 Sodium Salt (Internal Standard)

Empirical Formula: $C_{11}H_{11}D_8N_2NaO_4S_3$
Molecular Weight: 370.51

2.2 Reagents and Matrix

Reagent	Grade	Supplier
DMSO	Reagent	Fisher
Acetonitrile	HPLC	Fisher
Ultra-pure water	Milli-Q	In House
Formic acid	98-100%	Sigma Aldrich
Methanol	LC-MS	Fisher
Ammonium acetate	HPLC	Fisher
Sodium chloride	≤99.5%	Sigma Aldrich
Potassium chloride	≤99.0%	Sigma Aldrich
Magnesium chloride hexahydrate	≤99.0%	Sigma Aldrich
Sodium phosphate dibasic dihydrate	≤99.0%	Sigma Aldrich
Calcium chloride dihydrate	AR	Fisher
Sodium phosphate monobasic monohydrate	≤98%	Sigma Aldrich
Citric acid	99.5-100.5 %	Sigma Aldrich
**Matrix**	**Product Number**	**Supplier**
Human CSF	N/A	Supplied by Sponsor

2.3 Consumables

Description	Supplier	Product Number
ACE 5 C18-AR 50 x 3.0 mm C18 Column	Hichrom Ltd.	ACE-129-0503
Oasis HLB µElution Plate 30 µm	Waters	186001828BA

3 REAGENT PREPARATION

3.1 Mobile Phase A - 0.01% Aqueous Formic Acid

100 µL of formic acid is added to 1000 mL Milli-Q ultra-pure water. The mobile phase is sonicated for ~10 minutes after preparation.

3.2 Mobile Phase B - Acetonitrile

1000 mL of HPLC grade acetonitrile is transferred to a 1 litre bottle. The mobile phase is sonicated for ~10 minutes after preparation.

3.3 Sample Manager Wash

900 mL of HPLC grade acetonitrile and 100 mL of Milli-Q ultra-pure water are measured out separately and mixed together. The sample manager wash is sonicated for ~10 minutes after preparation.

3.4 Seal/Purge Wash

100 mL of HPLC grade acetonitrile and 900 mL of Milli-Q ultra-pure water are measured out separately and mixed together. The seal/purge wash is sonicated for ~10 minutes after preparation.

3.5 Purge Wash Solution (for infusion)

350 mL of HPLC grade acetonitrile and 150 mL of Milli-Q ultra-pure water are measured out separately and mixed together. The purge wash solution is sonicated for ~10 minutes after preparation.

3.6 10 mM Ammonium Acetate Solution

0.76 g of HPLC grade ammonium acetate is weighed out and transferred to a 1000 mL volumetric flask and dissolved in 1 litre of Milli-Q ultra-pure water.

3.7 10 mM Ammonium Acetate/MeCN (50:50)

50 mL of MeCN and 50 mL of 10 mM Ammonium Acetate Solution are measured out separately and mixed together.

3.8 0.5M Citric Acid

9.6 g of citric acid is weighed out and is dissolved in 100 mL of Milli-Q ultra-pure water.

3.9 Solution A for Artificial CSF

8.66 g of sodium chloride, 0.224 g of potassium chloride, 0.206 g of calcium chloride dihydrate and 0.163 g of magnesium chloride hexahydrate are weighed out and dissolved in 500 mL of Milli-Q ultra-pure water.

3.10 Solution B for Artificial CSF

0.027 g of sodium phosphate monobasic monohydrate and 0.142 g of sodium phosphate dibasic dihydrate are weighed out and dissolved in 500 mL of Milli-Q ultra-pure water.

3.11 Artificial CSF

50 mL of Solution A for Artificial CSF is mixed with 50 mL of Solution B for Artificial CSF.

3.12 Stabilised CSF

2 mL of 0.5 M citric acid is added to 100 mL blank human CSF or blank artificial CSF.

4 D,L SULFORAPHANE, D,L SULFORAPHANE GLUTATHIONE AND D,L SULFORAPHANE N-ACETYL CYSTEINE SOLUTION PREPARATION FOR CALIBRATION STANDARDS

Prepare a stock solution of D,L Sulforaphane by accurately weighing approximately 1 mg of D,L Sulforaphane into an amber glass vial and accurately adding the correct volume of DMSO to make a 1 mg/mL solution, taking into account any correction factor for purity. Mix well. Solution is labelled as "D,L Sulforaphane Solution A".

Prepare a stock solution of D,L Sulforaphane glutathione by accurately weighing approximately 1 mg of D,L Sulforaphane glutathione into an amber glass vial and accurately adding the correct volume of methanol to make a 1 mg/mL solution, taking into account any correction factor for purity. Mix well. Solution is labelled as "D,L Sulforaphane glutathione Solution B".

Prepare a stock solution of D,L Sulforaphane N-acetyl cysteine by accurately weighing approximately 1 mg of D,L Sulforaphane N-acetyl cysteine into an amber glass vial and accurately adding the correct volume of methanol to make a 1 mg/mL solution, taking into account any correction factor for purity. Mix well. Solution is labelled as "D,L Sulforaphane N-acetyl cysteine Solution C".

Prepare "Solution D" in a polypropylene bottle by accurately measuring 200 µL of "D,L Sulforaphane Solution A", 200 µl of "D,L Sulforaphane glutathione Solution B" and 200 µL of "D,L Sulforaphane N-acetyl cysteine Solution C", and mixing with 4400 µL of 10 mM Ammonium Acetate/MeCN (50:50) to make a 40 µg/mL solution of all three analytes.

5 CALIBRATION STANDARD PREPARATION

Prepare a set of calibration standards for D,L Sulforaphane, D,L Sulforaphane glutathione & D,L Sulforaphane N-acetyl cysteine in blank stabilised artificial CSF in micro centrifuge tubes at the following concentrations, as shown in Table 1.

Table 1: Calibration Standards

Solution ID	Solution Used	Volume (µL)	Stabilised aCSF (µL)	Overall Volume (mL)	Calibration Standard Concentration (ng/mL)
**STD9**	D (40 µg/mL)	50	950	1	2000
**STD8**	STD9	500	500	1	1000
**STD7**	STD8	500	500	1	500
**STD6**	STD7	500	500	1	250
**STD5**	STD6	500	750	1.25	100
**STD4**	STD5	500	500	1	50
**STD3**	STD4	500	500	1	25
**STD2**	STD3	500	750	1.25	10
**STD1**	STD2	500	500	1	5

NB – STD1 is not used for D,L Sulforaphane glutathione analysis.

Calibration standards should be mixed well and prepared daily. After preparation, calibration standards should be kept on wet ice before extraction.

6 D,L SULFORAPHANE, D,L SULFORAPHANE GLUTATHIONE AND D,L SULFORAPHANE N-ACETYL CYSTEINE SOLUTION PREPARATION FOR QUALITY CONTROL SAMPLES

Prepare a stock solution of D,L Sulforaphane by accurately weighing approximately 1 mg of D,L Sulforaphane into an amber glass vial and accurately adding the correct volume of DMSO to make a 1 mg/mL solution, taking into account any correction factor for purity. Mix well. Solution is labelled as "D,L Sulforaphane Solution E".

Prepare a stock solution of D,L Sulforaphane glutathione by accurately weighing approximately 1 mg of D,L Sulforaphane glutathione into an amber glass vial and accurately adding the correct volume of methanol to make a 1 mg/mL solution, taking into account any correction factor for purity. Mix well. Solution is labelled as "D,L Sulforaphane glutathione Solution F".

Prepare a stock solution of D,L Sulforaphane N-acetyl cysteine by accurately weighing approximately 1 mg of D,L Sulforaphane N-acetyl cysteine into an amber glass vial and accurately adding the correct volume of methanol to make a 1 mg/mL solution, taking into account any correction factor for purity. Mix well. Solution is labelled as "D,L Sulforaphane N-acetyl cysteine Solution G".

Prepare "Solution H" in a polypropylene bottle by accurately measuring 200 µL of "D,L Sulforaphane Solution E", "200 µL of "D,L Sulforaphane glutathione Solution F" and 200 µL of "D,L Sulforaphane N-acetyl cysteine Solution G", and mixing with 4400 µL of 10 mM Ammonium Acetate/MeCN (50:50) to make a 40 µg/mL solution of all three analytes.

7 BULK QUALITY CONTROL SAMPLES

Prepare a set of bulk QC samples for D,L Sulforaphane, D,L Sulforaphane glutathione & D,L Sulforaphane N-acetyl cysteine in blank stabilised artificial CSF in micro centrifuge tubes as shown in Table 2.

Table 2: Quality Control Samples

Solution ID	Solution Used	Volume (µL)	Stabilised aCSF (µL)	Overall Volume (mL)	QC Sample Concentration (ng/mL)
**QCDIL**	H (40 µg/mL)	800	3200	4	8000
**QC5**	H (40 µg/mL)	500	9500	10	2000
**QC4**	QC5	8000	2000	10	1600
**QC3**	QC4	2500	2500	5	800
**QC2**	QC3	225	11775	12	15
**QC1**	QC2	2000	4000	6	5
**GSHQC2**	QC3	450	11550	12	30
**GSHQC1**	GSHQC2	2000	4000	6	10

NB – GSHQC2 and GSHQC1 are used for D,L Sulforaphane glutathione analysis only.

Bulk Quality Control Samples should be mixed well, frozen at -80°C and defrosted on wet ice prior to use. Appropriate volumes for sets of each individual quality control sample will be pipetted out prior to freezing.

The sample volume for each quality control sample will be 700 µL.

8 INTERNAL STANDARDS

Mix all preparations well before use. Store preparations in polypropylene bottles at nominal 4°C.

1 mg/mL D,L Sulforaphane-d8

D,L Sulforaphane-d8 is supplied as an exact weight of approximately 1 mg. Add the correct volume of DMSO to make a 1 mg/mL solution. Mix well. Solution is labelled as "Internal Standard Solution V".

1 mg/mL D,L Sulforaphane N-acetyl cysteine-d8 sodium salt

D,L Sulforaphane N-acetyl cysteine-d8 sodium salt is supplied as an exact weight of approximately 1 mg. Add the correct volume of DMSO to make a 1 mg/mL solution. Mix well. Solution is labelled as "Internal Standard Solution W".

10 µg/mL D,L Sulforaphane-d8 & D,L Sulforaphane N-acetyl cysteine-d8 sodium salt

Prepare "Internal Standard Solution X" by accurately measuring 50 µL of Internal Standard Solution V and 50 µL of Internal Standard Solution W, and mixing with 4900 µL of 10 mM Ammonium Acetate/MeCN (50:50).

100 ng/mL D,L Sulforaphane-d8 & D,L Sulforaphane-d8 N-Acetyl-L-cysteine sodium salt

Prepare "Internal Standard Solution Y" by accurately measuring 200 µL of "Internal Standard Solution X" and mixing with 19800 µL of 10 mM Ammonium Acetate/MeCN (50:50).

Addition of 50 µL of "Internal Standard Solution Y" to the sample volume of 100 µL gives an in-sample IS concentration of 50 ng/mL for D,L Sulforaphane-d8 and 50 ng/mL for D,L Sulforaphane-d8 N-acetyl-L-cysteine sodium salt.

9 PRE-EXTRACTION PROCEDURE

Before beginning the extraction procedure, ensure the following samples have been prepared and/or are available:

1.	100 µL of appropriate STD samples have been aliquoted into a 2mL 96 well collection plate.
2.	Prepare a blank standard (STD0) by adding 100 µL of blank stabilised aCSF to a 2mL 96 well collection plate.
3.	100 µL of appropriate QC samples have been aliquoted into a 2mL 96 well collection plate.
4.	Study samples are available and thawed, and 100 µL of each study sample aliquoted into a 2mL 96 well collection plate.

5.	Enough CSF blanks have been prepared, by adding 100 µL blank stabilised aCSF to a 2mL 96 well collection plate.

10 EXTRACTION PROCEDURE

1.	Add 50 µL of Internal Standard Solution Y to each calibration standard (including STD0), quality control samples and study samples, **but not CSF blanks**.
2.	Add 50 µL of 10 mM Ammonium Acetate/MeCN (50:50) to the plasma blank samples **only**. All samples should now be 150 µL in volume, made up of 100 µL aCSF (or human CSF) and 50 µL of 10 mM Ammonium Acetate/MeCN (50:50).
3.	Add 300 µL of 0.5M citric acid solution to each sample, and mix using a 1200 µL multipipette.
4.	Ensure the waste plate is in place. Condition the HLB µElution plate with 200 µL of methanol. Apply a vacuum of ~ -13 in. Hg until the methanol has pulled through the plate.
5.	Repeat step 4 with 200 µL of MilliQ ultra-pure water.
6.	Remove the waste collection plate and insert the load collection plate. Load the standards, blanks, and study samples onto the HLB µElution plate using a 1200 µL multipipette. Once all samples are loaded, quickly switch the vacuum on then off, and allow the analytes to interact with the SPE bed for approximately 5 minutes. Then apply a vacuum of ~ -13 in. Hg until the run has pulled through the plate.
7.	Remove the load collection plate and reinsert the waste plate. Wash with 200 µL of MilliQ ultra-pure water. Apply a vacuum of ~ -13 in. Hg until the water has pulled through the plate.
8.	Remove waste plate and insert the elution collection plate.
9.	Elute with 2 x 50 µL of acetonitrile.
10.	Remove the elution collection plate from SPE device and transfer 100 µL of the load collection samples into the elution collection plate ensuring that samples are transferred in the same well plate conformation.
11.	Apply heat seal.

11 SYSTEM SUITABILITY

The system suitability will be verified before each sample analysis batch run by performing a system suitability test. The system suitability test will cover the following areas:

- Carryover - An extracted sample at a concentration equivalent to the ULOQ will be injected, followed by an injection of a blank. Any response in the blank at the retention time of the analytes must be less than 20% of the subsequent LLOQ sample response.

Any response in the blank at the retention time of the internal standards must be less than 5% of the internal standard response in the LLOQ sample.

- Absolute Retention - The absolute retention time must be within the range determined during the method qualification.
- Sensitivity - An extracted sample at the LLOQ will be injected directly after a blank sample and must show a signal-to-noise ratio equal to or greater than 5:1 to be acceptable.
- Chromatography - Peak shape will be assessed based on scientific experience of the Laboratory Scientist and/or the Project Leader.

12 LC-MS/MS METHOD

The LC-MS/MS method is attached as a pdf file (see Appendix 1). Peak processing settings may be optimised on a batch by batch basis.

13 AMENDMENT HISTORY

Method	Version	Changes from previous version
0001/023	V01	First version; Effective Date 30 Mar 2016
0001/023	V02	Version 02; change to calibration standard preparation, change of preparation to IS solution Y, minor change to extraction procedure; Effective Date 08 Apr 2016
0001/023	V03	Version 03; change to the preparation of stabilised aCSF, changes to the extraction procedure. Effective Date 25Apr2016

Appendix 2 Certificates of Analysis

Toronto Research Chemicals Inc.

CERTIFICATE OF ANALYSIS

2 Brisbane Road, Toronto, ON. M3J 2J8 Canada Tel: (416) 665-9696 Fax: (416) 665-4439
E-mail: orders@trc-canada.com Website: www.trc-canada.com

1. Identification

CAS Number:
4478-93-7

Catalogue Number:
S699115

Product:
D,L-Sulforaphane

LGC Standards GmbH
Mercatorstr. 51
46485 Wesel
Germany

Tel: +49 (0)281 9887 0
Fax: +49 (0)281 9887 199
Email: de@lgcstandards.com
Web: www.lgcstandards.com

Synonyms:
1-Isothiocyanato-4-(methylsulfinyl)-butane; 4-Methylsulfinylbutyl Isothiocyanate; Sulforaphan;

Structure:

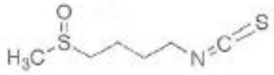

Molecular Formula:
$C_6H_{11}NOS_2$

Molecular Weight:
177.29

Source of Product:
Synthetic

2. Analytical Information

Lot Number:
5-YMK-40-1

Melting Point:
N/A

Boiling Point:
N/A

Atmosphere:
Inert Gas

Appearance of Product:
Pale Yellow Oil

Solubility
Chloroform (Slightly), Ethyl Acetate (Slightly)

Method for Determining Identity:
^{1}H NMR (DMSO-d_6, $CDCl_3$), ^{13}C NMR (DMSO-d_6), FT-IR, and MS

Stability
Light Sensitive

Purity:
95%

Long Term Storage Condition:
Amber Vial, -20°C Freezer, Under Inert Atmosphere

Additional Information:
TLC Conditions: SiO_2; Dichloromethane : Methanol = 7 : 1; Visualized with UV and AMCS; Single Spot, Rf = 0.45.
^{1}H NMR, ^{13}C NMR, FT-IR, and MS conform to structure.
Elemental Analysis: (Found) %C: 39.12, %H: 6.90, %N: 7.52; (Calculated) %C: 40.65, %H: 6.25, %N: 7.90

Philip Chan, Head of Quality Assurance

QC Test Date
October 7, 2015

Retest Date
October 5, 2018

Alderley Analytical

CERTIFICATE OF ANALYSIS

TRC Toronto Research Chemicals Inc.
Bringing you products for innovative research.
2 Brisbane Road, Toronto, ON. M3J 2J8 Canada Tel: (416) 665-9696 Fax: (416) 665-4439
E-mail: orders@trc-canada.com Website: www.trc-canada.com

1. Identification

CAS Number:
836682-32-7

Catalogue Number:
S699117

LGC Standards GmbH
Mercatorstr. 51
46485 Wesel
Germany
Tel: +49 (0)281 9887 0
Fax: +49 (0)281 9887 199
Email: de@lgcstandards.com
Web: www.lgcstandards.com

Product:
D,L-Sulforaphane-d8

Synonyme:
1-Isothiocyanato-4-(methylsulfinyl)-butane-d8; 4-Methylsulfinylbutyl Isothiocyanate-d8; Sulforaphan-d8;

Structure:

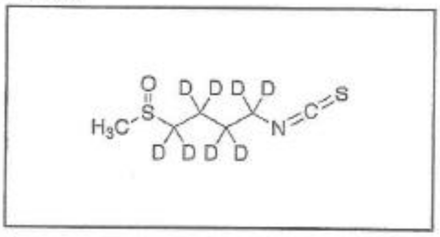

Molecular Formula:
$C_6H_3D_8NOS_2$

Molecular Weight:
185.34

Source of Product:
Synthetic

2. Analytical Information

Lot Number:
10-SBT-79-5

Melting Point:
N/A

Boiling Point:
N/A

Atmosphere:
Inert Gas

Appearance of Product:
Orange - Yellow Oil

Solubility
Chloroform (Slightly), Methanol (Slightly)

Method for Determining Identity:
. NMR ($CDCl_3$) and MS

Stability
Hygroscopic

Purity:
Chemical Purity: 98%
Isotopic Purity: 99.7%

Long Term Storage Condition:
Hygroscopic, Refrigerator, Under Inert Atmosphere

Additional Information:
TLC Conditions: SiO_2: Dichloromethane : Methanol = 9 : 1; Visualized with UV and $KMnO_4$; Rf = 0.75.
^1H NMR and MS conform to structure.
Elemental Analysis: (Found) %C: 38.96, %H: 6.05, %N: 7.13; (Calculated) %C: 38.88, %H: 5.99, %N: 7.56
Normalized Intensities: d_0 = 0.02%, d_1 = 0.00%, d_2 = 0.01%, d_3 = 0.00%, d_4 = 0.00%, d_5 = 0.08%, d_6 = 0.04%, d_7 = 1.56%, d_8 = 98.29%

Philip Chan, Head of Quality Assurance

QC Test Date
July 28, 2015

Retest Date
July 26, 2020

CERTIFICATE OF ANALYSIS

2 Brisbane Road, Toronto, ON. M3J 2J8 Canada Tel: (416) 665-9696 Fax: (416) 665-4439
E-mail: orders@trc-canada.com Website: www.trc-canada.com

1. Identification

CAS Number:
334829-66-2

Catalogue Number:
S699120

LGC Standards GmbH
Mercatorstr. 51
46485 Wesel
Germany

Tel: +49 (0)281 9887 0
Fax: +49 (0)281 9887 199
Email: de@lgcstandards.com
Web: www.lgcstandards.com

Product:
D,L-Sulforaphane N-Acetyl-L-cysteine

Synonyms:
N-Acetyl-S-[[[4-(methylsulfinyl)butyl]amino]thioxomethyl]-L-cysteine; Sulforaphane NAC;

Structure:

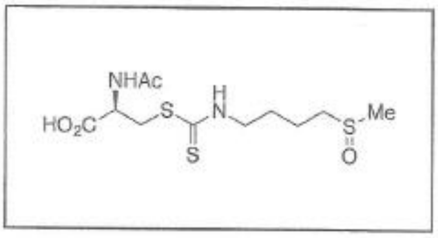

Molecular Formula:
$C_{11}H_{20}N_2O_4S_3$

Molecular Weight:
340.49

Source of Product:
N/A

2. Analytical Information

Lot Number:
6-GHZ-12-1

Melting Point:
58 - 63°C

Boiling Point:
N/A

Atmosphere:
Inert Gas

Appearance of Product:
Off-White Solid

Solubility
Methanol (Slightly)

Method for Determining Identity:
NMR (CD_3OD) and MS

Stability
Hygroscopic

Purity:
98%

Long Term Storage Condition:
Hygroscopic, -20°C Freezer, Under Inert Atmosphere

Additional Information:
TLC Conditions: C_{18}; Acetonitrile : Water = 9 : 1; Visualized with UV and AMCS; Single Spot, Rf = 0.50.
^{1}H NMR and MS conform to structure.
Specific Rotation: +11.1° (c = 0.5, Water)

Philip Chan, Head of Quality Assurance

QC Test Date
September 24, 2015

Retest Date
September 22, 2020

CERTIFICATE OF ANALYSIS

Toronto Research Chemicals Inc.
2 Brisbane Road, Toronto, ON. M3J 2J8 Canada Tel: (416) 665-9696 Fax: (416) 665-4439
E-mail: orders@trc-canada.com Website: www.trc-canada.com

1. Identification

LGC Standards GmbH
Mercatorstr. 51
46485 Wesel
Germany

Tel: +49 (0)281 9887 0
Fax: +49 (0)281 9887 199
Email: de@lgcstandards.com
Web: www.lgcstandards.com

CAS Number:
N/A

Catalogue Number:
S699122

Product:
D,L-Sulforaphane-d8 N-Acetyl-L-cysteine Sodium Salt

Synonyms:
N-Acetyl-S-[[[4-(methylsulfinyl)butyl-d8]amino]thioxomethyl]-L-cysteine Sodium Salt; Sulforaphane NAC Sodium Salt;

Structure:

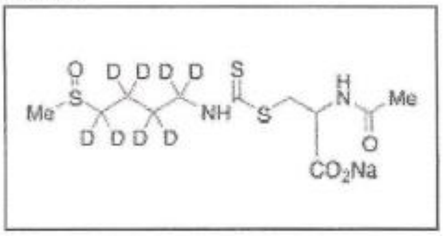

Molecular Formula:
$C_{11}H_{11}D_8N_2NaO_4S_3$

Molecular Weight:
370.51

Source of Product:
Synthetic

2. Analytical Information

Lot Number:
1-YSS-36-3

Melting Point:
>165.0°C (dec.)

Boiling Point:
N/A

Atmosphere:
Inert Gas

Appearance of Product:
Off-White Solid

Solubility
Methanol, Water

Method for Determining Identity:
^{1}H NMR (CD_3OD) and MS

Stability
Hygroscopic

Purity:
Chemical Purity: 95%
Isotopic Purity: 99.6%

Long Term Storage Condition:
Hygroscopic, -20°C Freezer, Under inert atmosphere

Additional Information:
TLC Conditions: C_{18}; Acetonitrile : Water = 9 : 1; Visualized with UV and $KMnO_4$; Single Spot, Rf = 0.45.
^{1}H NMR and MS conform to structure.
Elemental Analysis: (Found) %C: 33.28, %H: 5.72, %N: 7.01; (Calculated) %C: 37.91, %H: 5.79, %N: 8.04
Normalized Intensity: d_0 = 0.00%, d_1 = 0.00%, d_2 = 0.00%, d_3 = 0.00%, d_4 = 0.00%, d_5 = 0.00%, d_6 = 0.34%, d_7 = 2.68%, d_8 = 96.98%.
Water Content: 12.5% by Karl Fischer
Sodium Content: 5.67%

Philip Chan, Head of Quality Assurance

QC Test Date
August 14, 2014

Retest Date
August 12, 2017

CERTIFICATE OF ANALYSIS

Toronto Research Chemicals Inc.
2 Brisbane Road, Toronto, ON. M3J 2J8 Canada Tel: (416) 665-9696 Fax: (416) 665-4439
E-mail: orders@trc-canada.com Website: www.trc-canada.com

1. Identification

CAS Number:
289711-21-3

Catalogue Number:
S699430

LGC Standards GmbH
Mercatorstr. 51
46485 Wesel
Germany

Tel: +49 (0)281 9887 0
Fax: +49 (0)281 9887 199
Email: de@lgcstandards.com
Web: www.lgcstandards.com

Product:
D,L-Sulforaphane Glutathione

Synonyms:
L-γ-Glutamyl-S-[[[4-(methylsulfinyl)butyl]amino]thioxomethyl]-L-cysteinylglycine; SFN-GSH;

Structure:

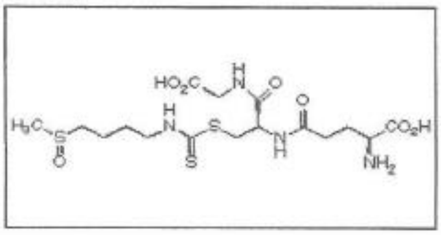

Molecular Formula:
$C_{16}H_{28}N_4O_7S_3$

Molecular Weight:
484.61

Source of Product:
Synthetic

2. Analytical Information

Lot Number:
1-CRD-3-1

Melting Point:
147 - 153°C (dec.)

Boiling Point:
N/A

Atmosphere:
Inert Gas

Appearance of Product:
Off-White Solid

Solubility
Methanol (Sparingly), Water (Sparingly)

Method for Determining Identity:
^1H NMR (D_2O) and MS

Stability
Hygroscopic

Purity:
95%

Long Term Storage Condition:
Hygroscopic, -20°C Freezer, Under inert atmosphere

Additional Information:
TLC Conditions: C_{18}; Methanol : Water = 9 : 1; Visualized with UV, AMCS, and Ninhydrin; Rf = 0.70.
^1H NMR and MS conform to structure.

Philip Chan, Head of Quality Assurance

QC Test Date
December 24, 2013

Retest Date
December 22, 2018

Appendix 3 Validation Plan

Bioanalytical Validation Plan Amendment 1 for the Determination of Sulforaphane (SFN), SFN N-acetyl Cysteine and SFN Glutathione in Human Cerebrospinal Fluid (CSF) by LC-MS/MS, using Artificial CSF as a Surrogate Matrix

Additions are indicted in bold italics text. Deletions are indicated by a strikethrough of the text.

Reasons for amendment:

1. Change to assigned Bioanalytical Project Leader due to extended absence.
2. Analyte previously named SFN N-Glutathione in error; changed in all places to SFN Glutathione.
3. An increase in the LLOQ for SFN Glutathione from 5 ng/mL to 10 ng/mL. The increase to the LLOQ is required for SFN Glutathione due to results obtained during method development for the selectivity of the assay for SFN Glutathione. When using an LLOQ of 5 ng/mL the blank samples analysed for the selectivity assessment were found to have SFN Glutathione peak areas that were greater than 20% of the LLOQ peak area. As a result the LLOQ will be increased.
4. Due to point 3 the calibration range for SFN Glutathione will be amended appropriately.
5. Correction of typographical errors.

Study Sponsor	Evgen Ltd.<br>146 Brownlow Hill<br>Liverpool<br>L3 5RF
Sponsor Contact	Dr David Howat<br>d.howat@evgen.com<br>Office +44 (0)151 705 3532<br>Cell +44 (0)7341 479346
Bioanalytical Test Site	Alderley Analytical<br>BioHub at Alderley Park<br>Alderley Edge<br>Cheshire<br>SK10 4TG
Bioanalytical Project Leader	~~Claire Wildgoose~~<br>~~claire.wildgoose@alderleyanalytical.com~~<br>~~Office +44 (0)1625 238610~~<br>***Alan Gibbs***<br>***alan.gibbs@alderleyanalytical.com***<br>***Office +44 (0)1625 238610***
Bioanalytical Method Title	Method for the Determination of SFN, SFN N-acetyl Cysteine and SFN Glutathione in CSF by LC-MS/MS
Alderley Analytical Method Number	0001/023
Alderley Analytical Study Number	0014/003

Species/Matrix/Stabiliser	Human / CSF / 0.5M citric acid
Stabiliser Concentration	***300 µL of 0.5M citric acid per 17.7 mL of CSF***
Surrogate Matrix	Artificial stabilised CSF
Projected Experimental Start Date*	~~March 2016~~ ***April 2016***
Projected Experimental Completion Date*	~~May 2016~~ ***June 2016***
Reference Standards	SFN<br>SFN N-acetyl Cysteine<br>SFN Glutathione<br>SFN-d8 (Internal Standard for SFN)<br>SFN-d8 N-acetyl Cysteine-d8 sodium salt (Internal Standard for SFN N-acetyl Cysteine and SFN Glutathione)

* A change in date does not necessitate an amendment to this document.

SIGNATURES OF APPROVAL

I hereby approve this validation study plan amendment and agree that this study will be conducted in accordance with the validation study plan and amendment, applicable regulatory requirements and the Alderley Analytical Standard Operating Procedures.

Alan Gibbs
Project Leader

23 May 2016
Date

I hereby assign the above-named Project Leader to the validation study described in this validation study plan amendment. The above named Project Leader is a permanent replacement for the previously assigned Project Leader.

Elizabeth Thomas
Elizabeth Thomas
CEO

23 May 2016
Date

I hereby approve this validation study plan amendment.

Dave Chadwick
David Chadwick
Study Sponsor Representative

23 MAY 2016
Date

TABLE OF CONTENTS

Page Number

1 INTRODUCTION 5

1.1 Objective 5
1.2 Compliance 5
1.3 Quality Assurance Evaluation 5

2 METHOD 5

2.1 Analysis Method Summary 5
2.2 Equipment 6
2.3 Surrogate Matrix 6
2.4 Preparation of Standard Calibration Curves 6
2.5 Preparation of Quality Control (QC) Samples 6

3 FULL VALIDATION 7

3.1 Linear Range and Response Function 7
3.1.1 Acceptance Criteria for Each Calibration Curve 7
3.2 Accuracy, Precision (Reproducibility), Sensitivity (LLOQ), and Upper Limit of Quantification (ULOQ) 8
3.2.1 Acceptance Criteria for the Accuracy and Precision (including LLOQ) of the Method 8
3.2.2 Acceptance Criteria for the Accuracy and Precision of the Upper Limit of Quantitation (ULOQ) of the Method 9
3.2.3 Sensitivity of the Method 9
3.3 Dilution Integrity 9
3.4 Recovery (Extraction Efficiency) 9
3.5 Selectivity 10
3.6 Matrix Effects for LC-MS/MS Methods 10
3.6.1 Evaluation of Human Stabilised CSF 11
3.6.2 Evaluation of CSF containing blood 11
3.7 Evaluation of a Large Run Size 11
3.8 Carryover Evaluation 12
3.9 Stability 12
3.9.1 Solution Stability 12
3.9.2 Extracted Sample Storage 12
3.9.2.1 Extract Stability 13
3.9.2.2 Re-injection Reproducibility / Autosampler Stability 13
3.9.3 Matrix Stability 14
3.9.3.1 Bench Top Stability at the Required Temperature 14
3.9.3.2 Freeze/Thaw Stability 14
3.9.3.3 Long Term Storage Stability (Frozen Stability) 14
3.10 Interference Screens 15
3.11 Retention Time Variability 15

4 REPORT FORMAT 15

4.1 Review of a Validation Prior to Validation Report Release 15
4.2 Issue of the Validation Report 15

5 ARCHIVE PROCEDURE 16

6 REFERENCES 16

1 INTRODUCTION

The validation procedures outlined in this validation plan follow Standard Operating Procedure (SOP) L001 (Ref. 1), which is broadly based on the Guidance for Industry document titled: "Bioanalytical Method Validation" generated by the U.S. Department of Health and Human Services, Food and Drug Administration, Centre for Drug Evaluation and Research (CDER), May 2001 and the European Medicines Agency "Guideline on Bioanalytical Method Validation", effective from 1 February 2012.

1.1 Objective

The full validation of a method for the quantitative analysis of SFN, SFN N-acetyl Cysteine and SFN Glutathione in human CSF with a calibration range of 5 to 2000 ng/mL ***for SFN and SFN N-acetyl Cysteine and 10 to 2000 ng/mL for SFN Glutathione***, using artificial CSF as a surrogate matrix.

1.2 Compliance

This study will be conducted in a laboratory inspected by the United Kingdom Good Laboratory Practice Monitoring Authority (GLPMA), and in accordance with Medicines and Healthcare products Regulatory Agency (MHRA) published guidance on Good Laboratory Practice for Clinical Laboratories. All work performed at Alderley Analytical will be carried out in accordance with the Alderley Analytical Standard Operating Procedures.

No formal claim of compliance will be made for this validation study.

1.3 Quality Assurance Evaluation

The Alderley Analytical Quality Assurance Unit will be responsible for the following quality assurance functions on the study:

- Validation Plan review
- Study specific procedure audits
- Data/report audit

2 METHOD

2.1 Analysis Method Summary

An LC-MS/MS (Liquid Chromatography with Tandem Mass Spectrometry) assay has been developed for the measurement of SFN, SFN N-acetyl Cysteine and SFN Glutathione in human stabilised CSF, using artificial stabilised CSF as a surrogate matrix. The method involves solid phase extraction from 100 µL of stabilised CSF. Separation is achieved using an ACE 5 C18-AR analytical column and a mobile phase consisting of 0.01% aqueous formic acid and acetonitrile.

2.2 Equipment

The equipment to be used for the determination of SFN, SFN N-acetyl Cysteine and SFN Glutathione in human and artificial CSF comprises of a Waters Acquity I-Class UPLC coupled to a Waters Xevo TQ-S Mass Spectrometer.

LC-MS/MS operation and data acquisition will be performed using UNIFI software V1.7.

2.3 Surrogate Matrix

The artificial CSF consists of the following components:

- Sodium chloride (NaCl)
- Potassium chloride (KCl)
- Calcium chloride dihydrate ($CaCl_2 . 2H_2O$)
- Magnesium chloride hexahydrate ($MgCl . 6H_2O$)
- Sodium dihydrogen phosphate (NaH_2PO_4)
- Disodium hydrogen phosphate dihydrate ($Na_2HPO_4 . 2H_2O$)

The preparation of the artificial CSF will be fully documented in the study file.

The artificial CSF will be stabilised with ***300*** µL of 0.5M citric acid per ***17.7*** mL of artificial CSF.

2.4 Preparation of Standard Calibration Curves

Calibration standards are prepared by adding diluted standard solutions of SFN in dimethyl sulfoxide (DMSO) and SFN N-acetyl Cysteine and SFN Glutathione in methanol to blank artificial stabilised CSF to create calibration standards at concentrations of 5.0 (Lower Limit of Quantification (LLOQ)), 10, 25, 50, 100, 250, 500, 1000 and 2000 (Upper Limit of Quantification (ULOQ)) ng/mL **for SFN and SFN N-acetyl Cysteine and 10 (Lower Limit of Quantification (LLOQ)), 25, 50, 100, 250, 500, 1000 and 2000 (Upper Limit of Quantification (ULOQ)) ng/mL for SFN Glutathione**. Single replicates of each calibration standard are produced, other than for those at the LLOQ and ULOQ, which are prepared in duplicate. Calibration curves will be produced by the simplest and most appropriate linear regression mode to describe the concentration-response relationship.

2.5 Preparation of Quality Control (QC) Samples

Bulk QC samples are prepared in artificial stabilised CSF and stored at a nominal temperature of -80°C at the start of method validation. QCs are prepared by adding diluted standard solutions of SFN in DMSO and SFN N-acetyl Cysteine and SFN Glutathione in methanol to artificial stabilised CSF to give a range of concentrations of approximately 5, 15, 800, 1600 and 2000 ng/mL for ***SFN and SFN N-acetyl Cysteine and 10, 30, 800, 1600 and 2000 ng/mL for SFN Glutathione***.

The QC samples should be prepared from different weighings of the analytical reference standards from those used to prepare the calibration curve. Alternatively, the QC samples may be prepared from

the same weighings as the standards, if the weighings in solution compare within ±5% to another independent weighing according to the formula below.

% difference = [(M1 – M2)/average of M1 and M2)] * 100

The concentrations of SFN, SFN N-acetyl Cysteine and SFN Glutathione in the QC samples are calculated by reference to the appropriate calibration curve and then compared to the theoretical (nominal) concentrations.

QC samples stored at a nominal -80°C will be analysed in a minimum of three validation batch runs, other than the QC sample at the ULOQ, which is analysed in one run only.

A dilution QC is prepared in replicates of 6 at 8000 ng/mL, see section 3.3.

3 FULL VALIDATION

3.1 Linear Range and Response Function

The relationship between response and concentration should be demonstrated to be continuous and reproducible. **A minimum of** eight levels of non–zero standards, as described in section 2.4, are used for constructing a calibration curve. In addition, a blank (named "Reagent Blank") and a "zero" standard (blank with internal standard, named "Blank") are analysed. One replicate of each standard is injected in ascending order at the start of the batch run. The remaining ULOQ and LLOQ standards should be run at the end of the batch, with all other samples in the batch run 'bracketed' by these two sets of calibration standards. Duplicate blank samples should also be injected after each ULOQ standard (see section 3.8).

Typically, a weighted linear regression is used, though the simplest relationship providing the best accuracy for the back-calculated concentrations for the standards is employed. For multiple analyte assays, the simplest weighting may differ for one or more analyte. In such cases, the more complex weighting may be used for all analytes in the method.

3.1.1 Acceptance Criteria for Each Calibration Curve

Any calibration standard having an assignable cause for rejection will be removed from the calibration curve first. Any remaining standards not meeting the acceptance criteria i.e. having a back-calculated concentration with a deviation of more than ±15% (±20% at the LLOQ) from its nominal concentration are then excluded from the calibration curve and the curve is sequentially reprocessed, beginning with the standard having the greatest percent difference from nominal. This procedure is repeated until all remaining standards are acceptable.

To accept a calibration curve, for each analyte:

- The standard curve must be constructed from a minimum of 75% of all of the calibration standards analysed in a run (including any that are excluded for assignable cause).
- At least one replicate each at the LLOQ and ULOQ must be included.

- The standard curve must contain at least one calibration standard at each of a minimum of six concentration levels.

3.2 Accuracy, Precision (Reproducibility), Sensitivity (LLOQ), and Upper Limit of Quantification (ULOQ)

Intra-run and inter-run accuracy and precision are determined by analysing artificial stabilised CSF quality control (QC) samples, as described in section 2.5, in replicates of at least six, at a minimum of four concentrations (LLOQ, low, mid and high), over a minimum of three batches on at least two different days.

During at least one batch run, extra samples at the ULOQ concentration are prepared in replicates of six (not including the standards used in the calibration curve) to assess accuracy and precision at the upper curve limit (see section 3.2.2).

3.2.1 Acceptance Criteria for the Accuracy and Precision (including the LLOQ) of the Method

For each validation occasion the intra-run accuracy and precision of the method will be determined at each concentration level, for each analyte as follows:

- For each concentration level, intra-run precision is defined as the coefficient of variation (CV) at each concentration level for each individual run. Precision should not exceed 15%, except at the LLOQ where a 20% limit is acceptable.
- For each concentration level, intra-run accuracy is defined as the percent bias (% bias) at each concentration level for each individual run. Acceptable limits are ±15% of the nominal concentration, except at the LLOQ where a ±20% limit is acceptable.
- For the LLOQ QC samples all results are used to calculate accuracy and precision, regardless of whether the observed concentration is below the nominal LLOQ concentration.

Note: If two consecutive validation occasions fail to meet the above intra-run acceptance criteria, the method will be re-evaluated and re-initiated when any issues have been resolved.

Across all validation occasions the accuracy and precision of the method will be determined at each concentration level as follows:

- Inter-run precision, defined as the CV, is calculated from all QCs analysed on all precision and accuracy validation occasions, at each concentration level. Precision should not exceed 15%, except at the LLOQ where a 20% limit is acceptable.
- Inter-run accuracy, defined as the %bias, is calculated from all QCs analysed on all precision and accuracy validation occasions at each concentration level. Acceptable limits are ±15% of the nominal concentration, except at the LLOQ where a ±20% limit is acceptable.

Quality control samples may be excluded from the tabulated data only if:

- There is assignable cause (e.g. anomalous chromatography or lack of internal standard).
- The sample is lost during the extraction or analysis.

Outliers can also be excluded if proven significant by a statistical method. Within a run, no more than a single replicate at each concentration level may be rejected as an outlier for calculation of precision and accuracy. If outliers are excluded, results calculated both with and without outliers will be presented in the validation report.

3.2.2 Acceptance Criteria for the Accuracy and Precision of the Upper Limit of Quantitation (ULOQ) of the Method

All results for the ULOQ samples should be used to calculate accuracy and precision, regardless of whether the observed concentration is above the nominal ULOQ concentration.

- Precision (coefficient of variation (CV)) for the six replicates should not exceed 15%.
- Accuracy for the six replicates should not deviate from the nominal concentration by more than ±15%.

3.2.3 Sensitivity of the Method

The LLOQ QC samples from all precision and accuracy batch runs are evaluated for adequate sensitivity, and must ideally demonstrate a signal to noise ratio of ≥5:1. Signal to noise of 5:1 is not mandatory and ultimately the definitive evidence of assay performance is that the precision and accuracy calculated at the LLOQ is within the acceptable limits of precision and accuracy.

3.3 Dilution Integrity

A QC sample (QC Dil) will be prepared in human stabilised CSF at a concentration of 8000 ng/mL for SFN, SFN N-acetyl Cysteine and SFN Glutathione, and diluted into the calibration range using a dilution factor of 10. The QC Dil sample is frozen for at least 24 hours, thawed, diluted, extracted, and analysed in replicates of at least six. The dilution of the QC Dil sample will be performed using artificial stabilised CSF. The replicates of the QC Dil sample must have a mean accuracy (%bias) of within ±15% of the nominal concentration, and the precision (%CV) must not exceed 15%, to be acceptable.

3.4 Recovery (Extraction Efficiency)

Recovery is determined by analysing at least three replicates of extracted samples at the low, mid, and high QC sample concentrations, along with at least three replicates of fortified samples at concentrations equivalent to extracted low, mid, and high QC sample concentrations. The recovery of the internal standards are determined in a similar manner at the working concentrations of the internal standards.

No formal acceptance limits are placed on the minimal recovery required; however, recovery should be consistent over the calibration range of the assay. The precision of recovery samples at each concentration level should not exceed 15%.

3.5 Selectivity

Six lots of blank matrix are processed and analysed in single replicates according to the method being validated. The six lots will be made up of the following;

- 1 pooled human CSF sample
- 3 individual human CSF donors
- 1 individual human CSF donor containing a level of blood (estimated to be approximately 0.1%)
- 1 lot of artificial CSF

The blank matrix samples should not produce any significant interference (i.e., a response (peak area, height or appropriate ratio)) that is:

- Greater than 20% of the average lower limit of quantification (LLOQ) response at the retention times of SFN, SFN N-acetyl Cysteine and SFN Glutathione.
- Greater than 5% of the mean internal standard response at the retention times of the internal standards.

However, in the event that run response deteriorates or improves over the course of the batch run, it is acceptable to compare the blank matrix to the LLOQ standard that was injected closest to the matrix blank.

If one of the lots fails to meet the above acceptance criteria, then the number of lots should be increased to 10, and at least 90% of the lots should not produce any significant interference. If two or more lots fail to meet the above acceptance criteria, the method should be investigated prior to proceeding.

3.6 Matrix Effects for LC-MS/MS Methods

Six lots of blank matrix (as described in section 3.5) are extracted in triplicate and then fortified with SFN, SFN N-acetyl Cysteine, SFN Glutathione and internal standards at a concentration equivalent to an extracted low QC sample and at a concentration equivalent to an extracted high QC sample (a total of 36 fortified samples (18 at each concentration level)). Additionally, two analytical solutions containing SFN, SFN N-acetyl Cysteine, SFN Glutathione and internal standards are prepared in triplicate in reconstitution solution, also at concentrations equivalent to an extracted low QC sample and an extracted high QC sample (a total of 6 analytical samples (3 at each level)). These are analysed in the following order:

Fortified lot 1 replicate 1, fortified lot 2 replicate 1, etc., with the three analytical solutions dispersed among the fortified samples (low QC level samples), followed by the same injection order for all high QC level samples.

The matrix effect is calculated for each lot using the Matrix Factor equation below:

$$\text{Matrix Factor} = \frac{\text{Mean Peak Area in Presence of Matrix Ions}}{\text{Mean Peak Area in Absence of Matrix Ions}}$$

At each concentration assessed, the overall precision of the matrix factors across all six lots should be ≤15%. If the overall precision of the matrix factors is not within these limits, the assessment may still

be considered acceptable if the overall precision of the normalised (IS adjusted) matrix factors across all six lots is ≤15%.

The normalised matrix effect is calculated for each lot using the equation below:

$$\text{Normalised Matrix Factor} = \frac{\text{Mean Peak Area Ratio in Presence of Matrix Ions}}{\text{Mean Peak Area Ratio in Absence of Matrix Ions}}$$

If both the Matrix Factor and Normalised Matrix Factor fail to meet the above acceptance criteria, then the method should be re-evaluated. However, precision outside the above acceptance criteria may be accepted, but the rationale for acceptance must be fully documented in the study file.

3.6.1 Evaluation of Human Stabilised CSF

The matrix effect of human stabilised CSF on the precision and accuracy of the method will be assessed. QC samples at low and high concentrations are prepared by spiking SFN, SFN N-acetyl Cysteine and SFN Glutathione into human stabilised CSF.

Six replicates of each QC sample, stored at the requisite temperature (nominal -80ºC) for at least 24 hours prior to use, will be analysed in a minimum of one validation batch run, along with a standard curve and run acceptance QCs in at least duplicate, prepared in artificial stabilised CSF.

Acceptance criteria for the human stabilised CSF QCs are the same as the criteria defined in section 3.2.1 for intra-run precision and accuracy.

3.6.2 Evaluation of CSF containing blood

The matrix effect resulting from the presence of blood in a CSF sample on the precision and accuracy of the method will be assessed. QC samples at low and high concentrations are prepared by spiking SFN, SFN N-acetyl Cysteine and SFN Glutathione into artificial stabilised CSF containing 0.1% K_2EDTA whole blood.

Six replicates of each QC sample, stored at the requisite temperature (nominal -80ºC) for at least 24 hours prior to use, will be analysed in a minimum of one validation batch run, along with a standard curve and run acceptance QCs in at least duplicate, prepared in artificial stabilised CSF.

Acceptance criteria for the QCs containing blood are the same as the criteria defined in section 3.2.1 for intra-run precision and accuracy.

3.7 Evaluation of a Large Run Size

At least one batch run should be injected that is approximately equivalent in size to a prospective sample analysis run. QCs should be extracted in replicates of at least six, but LLOQ QC samples need not be tested as part of this assessment.

The desired batch size may be achieved by extracting and injecting extra zero standards (recommended), or if sample volume permits, by re-injecting standards and/or QCs (or an entire batch) as many times as needed to achieve the desired run size. If multiple re-injections of the same QC

samples are made to achieve the desired run size, it must be specified in advance which QC injections are used to quantify this assessment.

For the assessment to be deemed acceptable the calibration curve acceptance criteria stated in section 3.1.1 and the intra-run accuracy and precision acceptance criteria stated in section 3.2.1 must be met. Furthermore, the run should be assessed for any significant bias from the beginning to the end of the run. If bias is observed it should be addressed in the raw data on a run by run basis.

3.8 Carryover Evaluation

Instrument carryover (cross-contamination) is evaluated by analysing two blank samples following each high standard during each validation run. The blanks should not produce any significant carryover i.e. a response (peak area or height) that is greater than 20% of the average LLOQ response, at the retention time of SFN, SFN N-acetyl Cysteine and SFN Glutathione. In addition, the blanks should not produce any significant carryover i.e. a response (peak area or height) that is greater than 5% of the mean internal standard response, at the retention time of the internal standard.

3.9 Stability

For all stability experiments, the date and time of sample removal and return to storage are documented appropriately.

3.9.1 Solution Stability

The solution stability of SFN, SFN N-acetyl Cysteine and SFN Glutathione will be determined during method validation study 0014/001 (Ref 2).

The results obtained will be included as an Appendix in the final report for this method validation.

The stability assessed during method 0014/001 will be as follows;

- Storage under the relevant conditions (i.e. appropriate solvent, container material and storage condition/temperature) for specific time period for the highest concentration and the lowest concentration solutions that are to be stored.
- Solution stability evaluated at ambient temperature after storage for approximately six hours or longer. An aliquot of the solution to be evaluated will be stored at ambient temperature, while another aliquot of the same solution is maintained at the requisite (cold) storage temperature. After storage, the two aliquots are compared.

3.9.2 Extracted Sample Storage

Three assessments are performed to demonstrate extracted sample storage stability and these are typically run concurrently, but may be run individually if needed.

- Extract Stability

- Re-injection Reproducibility
- Autosampler Stability

Extract storage times are established provided that:

- The calibration curve meets criteria in Section 3.1.1.
- At least two-thirds of the stored QC samples at each level (low, medium, and high) are within ±15% of their nominal concentrations.
- The mean concentration of the stored QC samples at each level (low, medium, and high) does not deviate from the nominal concentration by more than ±15%.

Note that the LLOQ QC samples are not used for these assessments.

3.9.2.1 Extract Stability

Extract Stability is determined over the anticipated time that an entire run may be stored prior to analysis. It will be established by storing an entire run (at a minimum; blanks, calibration standards and low, mid and high QC samples in triplicate) for the desired time at the requisite temperature prior to injection. Extract stability is calculated from the completion of sample preparation until injection of the first QC sample or calibration standard.

3.9.2.2 Re-injection Reproducibility / Autosampler Stability

Re-injection reproducibility / autosampler stability will be established by re-analysing a previously injected run (at a minimum, blanks, standards, and triplicate low, medium and high QC samples) after storage at the requisite autosampler temperature for the desired time. Freshly prepared calibration standards must *not* be added for the second injection of an entire run.

The re-injected QC samples will be compared separately to both the original calibration curve and to the re-injected calibration curve.

If the complete re-injection run meets acceptance criteria, an entire run may be re-injected during sample analysis after an interruption in analysis.

If the re-injected QCs meet acceptance criteria against the original calibration curve a partial run may be re-injected during sample analysis.

If the re-injected QCs and/or re-injected standards fail to meet acceptance criteria no reinjections may be performed and complete re-extraction and re-analysis will be required during sample analysis.

The re-injection reproducibility / autosampler stability storage time will be calculated from the original injection of the first QC sample or calibration standard until re-injection of the first QC sample or calibration standard.

It must be documented as to whether samples are stored with pierced or re-capped vials after the initial injection.

3.9.3 Matrix Stability

Quality control samples used for stability are typically prepared in bulk, aliquoted to smaller volumes, and stored frozen prior to use. For the following matrix stability assessments, the stability QC samples are extracted along with blanks, and a freshly prepared calibration curve (i.e., prepared fresh from stable stock and/or spiking solutions).

Run acceptance QCs (low, mid, and high concentrations prepared in artificial stabilised CSF, in at least duplicate) must also be analysed if the stability assessment is not conducted in a precision and accuracy run during validation. Run acceptance QC samples are separate from the stability QC samples and must be within proven stability.

At least 2/3 of the run acceptance QC samples, with at least 50% at each level, must be within ±15% of their nominal concentrations.

If degradation is indicated for any of the matrix stability assessments, a shorter storage time may be analysed to determine when the instability occurs. Alternatively, a colder temperature (e.g., an ice bath or colder freezer temperature) or other means of stabilising a sample can be used, and the test repeated.

3.9.3.1 Bench Top Stability at the Required Temperature

Three levels of QC samples (low, high and QC Dil) in both human and artificial stabilised CSF are analysed in at least triplicate after maintaining them at the required temperature for a minimum of four hours prior to extraction. The temperature and storage time assessed must be documented in the study file.

Stability is indicated provided that at least 2/3 of the stability QC samples at each level are within ±15% of their nominal concentrations, and the mean concentration at each level does not deviate from the nominal concentration by more than ±15% in both artificial and human CSF.

3.9.3.2 Freeze/Thaw Stability

Three levels of QC samples (low, high and QC Dil) in both human and artificial stabilised CSF are frozen for at least 24 hours at a nominal temperature of -80ºC and thawed on wet ice. When completely thawed, the samples are then returned to the freezer for at least 12 hours under the same storage conditions. This cycle is performed a minimum of three times. After the final cycle, the samples are analysed in at least triplicate. The acceptance criteria is as above in section 3.9.3.1.

3.9.3.3 Long Term Storage Stability (Frozen Stability)

A separate study, to investigate frozen stability over three time points, has been agreed. This will therefore be covered in a separate analysis plan (study number 0014/004).

3.10 Interference Screens

SFN, SFN N-acetyl Cysteine and SFN Glutathione will be analysed independently to monitor the contribution of each analyte on the other. This will be performed at the LLOQ and ULOQ level for each analyte.

Independent samples containing each separate analyte will be prepared at the lower and upper limit of quantification (without addition of internal standard) in artificial CSF and analysed in triplicate. These will be analysed for the other analytes (i.e. samples spiked with SFN will be analysed for SFN N-acetyl Cysteine and SFN Glutathione, and so on).

The pure standards of the metabolites are known to contain an amount of SFN and therefore rather than assign definitive acceptance criteria for the response observed in the interference samples (typically when spiked at the ULOQ any interference response noted should be less than 20% of the average LLOQ response for the analyte in question), the impact of any interference observed over the concentration range will be assessed and the results discussed in the validation report.

3.11 Retention Time Variability

Absolute retention time is monitored during each validation run by comparing the retention times of analytes and internal standards at the beginning of a run to those at the end of a run. These should vary by no more than 10%.

4 REPORT FORMAT

A validation report will be prepared from the Alderley Analytical validation report template.

4.1 Review of a Validation Prior to Validation Report Release

It is acceptable to begin study sample analysis after the validation is completed but prior to the validation report being reviewed by the Quality Assurance Unit. In such instances, sample analysis may proceed provided that the Project Leader has reviewed the validation data, validation acceptance criteria are met, and the Sponsor is in agreement with this action.

4.2 Issue of the Validation Report

A draft report will be prepared following completion of the study and will be finalised following consultation with the Sponsor and Quality Assurance review. The report will include all information necessary to provide a complete and accurate description of the experimental methods and results and any circumstances that may have affected the quality or integrity of the study.

The Sponsor will receive an electronic version of the draft validation report in a Microsoft Word format. Any comments made by the Sponsor must be returned as tracked changes using the Microsoft Word version. The final validation report will be provided in Adobe Acrobat PDF format. The validation report will be created from electronic files to the extent possible, including text and tables generated by Alderley Analytical. Report entries not available as electronic files and/or original signature pages will

be scanned and converted to PDF files for incorporation into the report. An original copy of the report with Alderley Analytical handwritten signatures will be retained in the Alderley Analytical Archive.

5 ARCHIVE PROCEDURE

All records of the study including the Validation Plan, raw data and approved final report are archived in the Alderley Analytical Archive, as documented according to Alderley Analytical SOP QA009 (Ref. 3). Materials will be retained for a period of two years from the date of finalising the report after which time the Sponsor will be contacted to determine requirements for further storage, return or destruction of materials. No materials will be destroyed without written instruction from the Sponsor.

6 REFERENCES

1. Alderley Analytical SOP L001: Validation of Bioanalytical Methods
2. Alderley Analytical Study Number 0014/001: Validation for the Determination of SFN, SFN N-acetyl Cysteine and SFN Glutathione in Human K_2 EDTA Stabilised Plasma by LC-MS/MS.
3. Alderley Analytical SOP QA009: Archiving Procedures / Records Management

Appendix 4 Stock Stability

Table 15a SFN Stock Stability (Low Conc Solution) over 29 Days

Solution Concentration 40 µg/mL	Stored Soln – 29 Days in 50/50 10 mM Ammonium Acetate/ACN at 4°C	Fresh Soln
	Peak Area	Peak Area
	648775	636855
	704539	636372
	683944	632412
Mean	679086	635213
SD	28198	2438
CV	4.15%	0.38%
Stability	107%	

Table 15b SFN Stock Stability (High Conc Solution) over 67 Days

Solution Concentration 1 mg/mL	Stored Soln – 67 Days in DMSO at 4°C	Fresh Soln
	Peak Area	Peak Area
	3360129	3342288
	3325825	3335828
	3305668	3282253
	3257128	3479061
	3292992	3504633
	3278510	3540893
	3531944	3158282
	3559979	3176226
	3602142	3189603
Mean	3390480	3334341
SD	134936	146770
CV	3.98%	4.40%
Stability	102%	

Table 15c SFN-NAC Stock Stability (Low Conc Solution) over 29 Days

Solution Concentration 40 µg/mL	Stored Soln – 29 Days in 50/50 10 mM Ammonium Acetate/ACN at 4ºC	Fresh Soln
	Peak Area	Peak Area
	2277997	2185857
	2449085	2183819
	2434323	2156971
Mean	2387135	2175549
SD	94804	16121
CV	3.97%	0.74%
Stability	110%	

Table 15d SFN-NAC Stock Stability (High Conc Solution) over 69 Days

Solution Concentration 1 mg/mL	Stored Soln – 69 Days in Methanol at -20ºC	Fresh Soln
	Peak Area	Peak Area
	1073071	1408127
	1076520	1345766
	1049102	1316492
	1160270	1194793
	1132882	1102941
	1160013	1136124
	1237470	1094431
	1236991	1102936
	1248821	1113150
Mean	1152793	1201640
SD	76576	122346
CV	6.64%	10.18%
Stability	96%	

Table 15e SFN-GSH Stock Stability (Low Conc Solution) over 29 Days

Solution Concentration 40 µg/mL	Stored Soln – 29 Days in 50/50 10mM Ammonium Acetate/ACN at 4°C	Fresh Soln
	Peak Area	Peak Area
	10345831	11350731
	11005958	11208759
	10926766	11368572
Mean	10759519	11309354
SD	360445	87573
CV	3.35%	0.77%
Stability	95%	

Table 15f SFN-GSH Stock Stability (High Conc Solution) over 67 Days

Solution Concentration 1 mg/mL	Stored Soln – 67 Days in Methanol at -20°C	Fresh Soln
	Peak Area	Peak Area
	1657416	1307866
	1600269	1400582
	1628381	1434932
	1660799	1507275
	1668402	1517363
	1649089	1485711
	1715400	1534809
	1627455	1512937
	1665618	1553216
Mean	1652536	1472743
SD	32448	78355
CV	1.96%	5.32%
Stability	112%	
