# Supplementary material for: A Randomised Controlled Trial of SFX-01 After Subarachnoid Haemorrhage — The SAS Study
Source: Transl Stroke Res. 2024 Jul 19;16(4):1031–43. doi: 10.1007/s12975-024-01278-1 (PMC12202693; doi:10.1007/s12975-024-01278-1)
Supplement: Supplementary file 4 — Supplementary file4 - Statistical analysis plan (PDF 894 KB) [file 12975_2024_1278_MOESM4_ESM.pdf]

DocuSign Envelope ID: 07B92F3A-AB49-4D0C-B36F-26112605A392



# Evgen Pharma Plc

# Protocol: EVG001SAH

## Eudract No.: 2014-003284-38

## *Statistical Analysis Plan:*

A Phase II Safety, Tolerability, Pharmacokinetic and Pharmacodynamic Study of SFX-01 in Subarachnoid Haemorrhage, with exploratory efficacy evaluations. The study is a randomised, double-blind, parallel-group design comparing SFX-01 (300 mg) taken orally as capsules or as a suspension via a nasogastric tube (NG) twice-daily for up to 28 days versus placebo in 90 patients who have had SAH and present within 48 hours of ictus.

| | |
|---|---|
| Sponsor: | Evgen Pharma plc<br><br>Liverpool Science Park IC2<br>146 Brownlow Hill<br>Liverpool<br>L3 5RF |
| Author & Qualifications | Andrew Stone M.Sc. C.Stat. |
| Document Date: | 04 February 2019 |
| Version/Status: | Final |

## AUTHORISATION

| Position | Name | Signature | Date (dd mon yyyy) |
|---|---|---|---|
| Medical Advisor<br>Evgen Pharma plc | Dr. Thomas Morris | DocuSigned by:<br>Thomas Morris<br>62C1C1B7C7114D3... | 2/4/2019 |
| Clinical Development Officer<br>Evgen Pharma plc | Sally Ross | DocuSigned by:<br>Sally Ross<br>6A6F318FC77041D... | 04 Feb 2019 |
| Project Manager<br>TCTC Ltd | Ingrid Gerber | DocuSigned by:<br>Ingrid Gerber<br>3E763580DFD443E... | 2/5/2019 |
| Statistical Consultant<br>Stone Biostatistics Ltd | Andrew Stone | DocuSigned by:<br>A Stone<br>BF0293A0587A44B... | 2/4/2019 |

## DOCUMENT HISTORY

| Version | Date | Author | Section/Page Amendment |
|---|---|---|---|
| Final | 04 Feb 2019 | Andrew Stone | Finalised SAP |

DocuSign Envelope ID: 07B92F3A-AB49-4D0C-B36F-26112605A392



# Table of Contents

1 INTRODUCTION ........ 7

2 OBJECTIVES ........ 8

3 STUDY DESIGN ........ 9

3.1 Sample Size ........ 9

4 ANALYSIS POPULATIONS ........ 10

5 PRIMARY AND SECONDARY VARIABLES ........ 11

5.1 Trans-Cranial Doppler ........ 11

5.2 Modified Rankin Score ........ 12

5.3 Glasgow Outcome Score Extended ........ 12

5.4 Incidence of Delayed Cerebral Ischaemia ........ 13

5.5 Incidence of New Cerebral Infarct ........ 13

5.6 Triple H therapy ........ 13

5.7 SF-36 Quality of Life Survey ........ 13

5.8 CLCE-24 ........ 15

5.9 BICRO-39 ........ 16

5.10 Subarachnoid Haemorrhage Outcomes Tool ........ 17

5.11 Length of acute hospital stay ........ 18

5.12 MRI Susceptibility Weighted Imaging ........ 18

5.13 Safety Data ........ 18

5.14 Pharmacokinetic Data ........ 19

5.15 Time-Windows ........ 19

6 STATISTICAL METHODS ........ 20

6.1 General Considerations ........ 20

6.2 Analysis Methods ........ 20

6.3 Multiplicity ........ 22

6.4 Analysis of the Primary Endpoint ........ 22

6.5 Analysis of Secondary Efficacy Endpoints ........ 23

DocuSign Envelope ID: 07B92F3A-AB49-4D0C-B36F-26112605A392



**6.6 Analysis of Pharmacokinetic Endpoints** ..... 24

**6.7 Missing Data Diagnostics** ..... 24

**6.8 Subgroup analyses** ..... 25

**6.9 Changes from Protocol** ..... 25

**7 PRESENTATION OF DATA** ..... 27

**8 REFERENCES** ..... 32

DocuSign Envelope ID: 07B92F3A-AB49-4D0C-B36F-26112605A392



# List of Abbreviations and Definition Of Terms

| | |
|---|---|
| AE | Adverse Event |
| ANOVA | Analysis Of Variance |
| ATC | Anatomical Therapeutic Chemical |
| AUC | Area Under the Curve |
| bid | Two times daily |
| BICRO-39 | Brain Injury Community Rehabilitation Outcomes Scale |
| BP | Pain |
| CLCE-24 | Checklist for Cognitive and Emotional Consequences CRF |
| CRF | Case Report Form |
| CRP | C-reactive protein |
| CSF | Cerebrospinal Fluid |
| CSR | Clinical Study Report |
| CT | Computed Tomography |
| CV | Coefficient of Variation |
| DCI | Delayed Cerebral Ischaemia |
| DSA | Digital Subtraction Angiography |
| DSMB | Data Safety Monitoring Board |
| EOS | End of Study |
| EVD | External Ventricular Drain |
| EW | Emotional Well-Being |
| GCS | Glasgow Coma Scale |
| GH | General Health |
| GOSE | Glasgow Outcome Scale (Extended) |
| HL | Hodges-Lehmann |
| HP | Haptoglobin |
| ICH | International Conference on Harmonisation |
| ITT | Intention-to-Treat |
| IV | Intravenous |
| LP | Lumbar Puncture |
| MCA | Middle Cerebral Artery |
| MDA | Malondialdehyde |
| MedDRA | Medical Dictionary for Regulatory Activities |
| MFV | Mean Flow Velocity |
| MH | Mental Health |
| MMRM | Mixed Model Repeated Measures |
| MRA | Magnetic Resonance Angiography |
| MRI | Magnetic Resonance Imaging |
| mRS | Modified Rankin Scale |
| NA | Not Applicable |
| NG | NasoGastric |
| PF | Physical Functioning |
| PK | Pharmacokinetic |
| PP | Per-Protocol |
| PTAE | Pre-Treatment Adverse Event |
| RE | Role Limitations Due to Emotional Problems |
| RP | Role Limitations Due to Physical Health |
| SAE | Serious Adverse Event |
| SAH | Subarachnoid Haemorrhage |
| SAP | Statistical Analysis Plan |
| SAHOT | Subarachnoid Haemorrhage Outcome Tool |

DocuSign Envelope ID: 07B92F3A-AB49-4D0C-B36F-26112605A392



| | |
|---|---|
| SF | Social Functioning |
| SF-36 | Short Form (36) Health Survey |
| SFN | Sulforaphane |
| SFX-01 | The Investigational Medicinal Product/stabilised Sulforaphane |
| SOC | System Organ Class |
| TCD | Trans-Cranial Doppler |
| TCTC | The Clinical Trial Company Ltd |
| TEAE | Treatment Emergent Adverse Event |
| VT | Energy/Fatigue |
| WBC | White blood cells |
| WFNS | World Federation of Neurosurgical Scale |
| WHODD | World Health Organization Drug Dictionary |
| Z | Standardized Normal Distribution Test Statistic |

DocuSign Envelope ID: 07B92F3A-AB49-4D0C-B36F-26112605A392



# 1 Introduction

This document describes the objectives, analysis populations, endpoint derivations, statistical analyses and data presentations to be performed for the clinical protocol EVG001SAH entitled "SFX-01 After Subarachnoid Haemorrhage (SAS Study)". This is the final version of the SAP text, approved in February 2019 which updates previous draft versions of the SAP to provide more detail on endpoint derivations and the analyses to be performed. In general, details of the study design that can also be found in the protocol have been removed from this version. The supporting tables, listing and figures will be finalised in a separate document. Any changes made in the course of the evaluation and analysis of these data performed after the locking and un-blinding the database will be documented and fully justified in the final Clinical Study Report (CSR). The contents of the SAP are consistent with the principles described in the ICH E8 and E9 guidelines [1,2].

DocuSign Envelope ID: 07B92F3A-AB49-4D0C-B36F-26112605A392



# 2 Objectives

**Primary**:
To evaluate the safety of up to 28 days of SFX-01 dosed at up to 92 mg Sulforaphane (SFN) per day.
To investigate the pharmacokinetic properties of SFN in cerebrospinal fluid (CSF).
To determine if a minimum of 7 days treatment with SFX-01 reduces Middle Cerebral Artery (MCA) peak flow velocity following Subarachnoid Haemorrhage (SAH).

**Secondary**:

To determine if a minimum of 7 days treatment with SFX-01 improves clinical outcome following SAH as measured using the modified Rankin Scale assessed at 7 days, discharge, 28, 90 and 180 days post ictus.
To determine blood SFN levels (and its metabolites) with treatment with SFX-01 (300mg bid).
To determine CSF SFN levels and kinetics with treatment with SFX-01 (300mg bid).
To determine if up to 28 days treatment with SFX-01 increases blood haptoglobin (HP) levels and decreases malondialdehyde (MDA) levels following SAH.
To determine if up to 28 days treatment with SFX-01 can reduce the incidence of Delayed Cerebral Ischaemia (DCI) following SAH.
To determine if up to 28 days treatment with SFX-01 improves long-term outcome in subjects following SAH.
To determine if up to 28 days of treatment with SFX-01 can reduce iron deposition and cortical atrophy following SAH.

DocuSign Envelope ID: 07B92F3A-AB49-4D0C-B36F-26112605A392



# 3 Study Design

The study is a randomised, double-blind, parallel-group design comparing SFX-01 (300 mg bd) administered for up to 28 days versus Placebo control in 90 patients who meet the per-protocol criteria for efficacy analyses, who have had a SAH and are referred to neurosurgical units for tertiary care.

Treatment is taken twice-daily either orally or via nasogastric tube (NG). For the first 20 patients, treatment duration is determined by the length of time spent in tertiary care (up to Day 28 post ictus). Following a DSMB review (as per protocol) for all subsequent patients the intended treatment duration is 28 days regardless of whether they remain in tertiary care for the full 28-day period. After treatment all patients are then followed up at discharge from the neurosurgical unit and then subsequently on Day 28, Day 90 and Day 180 post ictus.

A separate and more detailed investigation (sub-study) of the pharmacokinetic properties of SFN parent drug, and the primary metabolite, both in CSF and blood is also undertaken. This will involve up to 12 patients who have an External Ventricular Drain (EVD) fitted as part of their normal standard of care and is undertaken on two separate occasions within the first 7 days post ictus.

The protocol was amended part way through recruitment to stratify the randomisation by WFNS score (1-3 v 4-5) and centre.

## *3.1 Sample Size*

Following protocol amendment 5, the sample size was increased from 90 to up to 120 patients in order to provide 90 who would meet the per protocol criteria and be evaluable for the primary efficacy analysis. Furthermore, this protocol amendment also specified that patients who had potentially received insufficient or incorrect study medication may be replaced.

If the standard deviation is approximately half (53%) of the mean difference in the maximum MCA flow velocity, 90 evaluable patients will have 80% power to detect a statistically significant difference between treatment arms using a 1-sided type 1 error rate of 5%. Statistical significance will be declared if the 2-sided p-value is <0.05, in this case the trial will have >80% power with 90 evaluable patients if the standard deviation is <59% of the mean difference.

# 4 Analysis Populations

Data from this study will be analysed on two occasions although efficacy endpoints will only be analysed on one occasion. The data cut-off for the first analysis of the study will be 28 days after the last patient is randomized. After this first data-cut-off, the TCD data will be analysed and available safety and PK data will be reported. The second data cut-off will occur 6 months after the last patient is randomized. This second and final analysis will include all available safety, pharmacokinetic and secondary efficacy endpoint data.

## *4.1 Population Definitions*

Four patient populations will provide the basis for all statistical analyses and data presentations.

**Per Protocol (PP) population:** This will consist of all patients who receive at least 10 doses of randomised treatment within the first 7 days of the first dose of study medication. However, there were nine patients (014, 015, 016, 017, 020, 021, 032, 033, 034) who were either known to have had (n=2), potentially had (n=3), or were associated with patients who had (n=4), a discrepancy in dispensing of randomised therapy, these patients will be excluded from the PP population.

**Intent-To-Treat (ITT) population**: All randomised patients who receive at least one dose of study medication. This population will include the nine patients excluded from the PP population due to possible errors in dispensing.

The primary population for efficacy endpoints will be the PP population with select efficacy endpoints also analysed in the ITT population (see Section 6.2)

**Safety population**: All randomised patients who have taken at least one dose of study medication including the nine patients excluded from the PP population due to possible errors in dispensing. This population will be applied to all safety endpoints and pharmacokinetic data recorded in all patients. The safety population will be identical to the ITT population if all patients dosed have efficacy data recorded.

**PK Sub-Study population:** A group of up to 12 patients fitted with an EVD as part of their normal treatment are to be selected for the pharmacokinetic sub-study.

Given the early stage of development, the PP population is of primary interest and all patients will be analysed according to the treatment they actually receive in all populations. For the ITT population, if due to dispending errors it is known patients received a mixture of SFX-01 and placebo capsules, they will be assigned to the arm for which they received medication for the majority of time.

In addition, clinically important protocol violations will be identified in a blinded manner and then summarised and listed in the clinical study report (CSR).

DocuSign Envelope ID: 07B92F3A-AB49-4D0C-B36F-26112605A392



# 5 Primary and Secondary Variables

## *5.1 Trans-Cranial Doppler*

An initial TCD reading is taken within 48 hours of ictus. Subsequent TCD readings are then taken three times a week on alternate days (according to standard care procedures). They are performed at least until Day 7 post ictus (± 1)) and then until no longer clinically indicated. Table 1 describes the data collected at each timepoint noting that the individual value recorded represents the maximum of the mean flow recorded during the recording period.

**Table 1. TCD Recordings**

| **Reading** | **Units/Response** |
|---|---|
| Left Middle Cerebral Artery Mean Flow Velocity | cm/s |
| Right Middle Cerebral Artery Mean Flow Velocity | cm/s |
| Left Internal Cerebral Artery Mean Flow Velocity | cm/s |
| Right Internal Cerebral Artery Mean Flow Velocity | cm/s |
| Assessment Performed | Pre/Post |
| Lindergaard Ratio Left | none |
| Lindergaard Ratio Right | none |

The primary endpoint is defined as the maximum of all left middle cerebral artery mean flow velocities (MCA-MFV) and right MCA-MFV values recorded after the first dose of randomised therapy including any taken after Day 7.

Baseline values will be defined as:

- The lowest of the left and right MCA-MFV values recorded at the earliest TCD assessment made within 2 days of first dose
- If a patient does not have a TCD recorded within 2 days of dosing, a predicted baseline value will be imputed based on their age, WFNS score (1 to 5), a past medical history of hypertension (yes/no), surgical procedure (clipping, coiling, none), log-CRP and centre as described below.

Amongst patients with baseline values recorded within 2 days of dosing, their baseline value will be regressed on age, WFNS score, hypertension, surgical procedure, log-CRP and centre. The imputed value for patients with a missing baseline value will equal $\mu + \sum x_{ij}\beta_j$ where $x_{ij}$ represent the observed values of the j covariate levels for the $i^{th}$ patient and $\beta_j$ the corresponding parameter estimates from the regression amongst patients with observed baseline values.

Supplementary analyses will also be performed on the mean of the three largest right or left MCA-MFV recorded at any timepoint after dosing. Additionally, an analysis will be performed to describe the effect over time with timepoints grouped, relative to date of Ictus, as Days 3-4, 5-6, 7-9, 10-14, 15-21, 22-28. Within each timepoint the largest MCA-MFV value will be included in the analysis for each patient. For these two analyses, the same baseline as the primary analysis will be used. Using the timepoint analysis the mean effect of Days 5 to 9 will also be estimated.

The analysis of Lindergaard ratio will mirror the primary analysis of MCA-MFV values: the maximum of all ratios recorded after the first dose of randomised therapy will be analysed and

baseline will be defined as the lowest of the left and right ratios recorded at the earliest TCD recording made within 2 days of first dose, imputing data using the same model approach if baseline values are otherwise missing.

All TCD endpoints will be $\log_e$-transformed prior to analysis.

## 5.2 Modified Rankin Score

The Modified Rankin Scale (mRS) is recorded at Day 7, 28, 90 and 180 as well as at discharge. mRS will be analysed as a score ranging from 0-6 as displayed in Table 2. Any patient who has died before the respective timepoint will be given a score of 6. Any mRS recorded at the discharge visit that is contained within the protocolled defined time-windows and is closer to the nominal time than any other recordings will be used at that timepoint.

**Table 2. Modified Rankin Score (mRS)**

| mRS recorded response | Score assigned for analysis |
|---|---|
| No symptom at all | 0 |
| No significant disability despite symptoms; able to carry out all usual duties and activities | 1 |
| Slight disability; unable to carry out all previous activities, but able to look after own affairs without assistance | 2 |
| Moderate disability; requiring some help, but able to walk without assistance | 3 |
| Moderately severe disability; unable to walk without assistance and unable to attend to own bodily needs without assistance | 4 |
| Severe disability; bedridden, incontinent and requiring constant nursing care and attention | 5 |
| Dead | 6 |

## 5.3 Glasgow Outcome Score Extended

The Glasgow Outcome Scale – Extended (GOSE) is recorded at Day 28, 90 and 180. GOSE will be analysed as a score ranging from 1-8 as displayed in Table 3. Any patient who has died before the respective timepoint will be given a score of 1.

**Table 3. Glasgow Outcome Scale – Extended (GOSE)**

| GOSE recorded response | Score assigned for analysis |
|---|---|
| Death | 1 |
| Vegetative state | 2 |
| Lower severe disability | 3 |
| Upper severe disability | 4 |
| Lower moderate disability | 5 |
| Upper moderate disability | 6 |
| Lower good recovery | 7 |
| Upper good recovery | 8 |

## 5.4 Incidence of Delayed Cerebral Ischaemia

Delayed Cerebral Ischaemia (DCI) is defined as a new focal deficit or reduction in Glasgow Coma Scale ≥2 if not explained by other causes (i.e. re-bleed, hydrocephalus, seizure, meningitis, sepsis or hyponatremia). Patients with the following MedDRA terms will be examined as part of a blinded medical review to decide whether the patient has had a DCI satisfying the outlined criteria:

Delayed ischaemic neurological deficit
Cerebral ischaemia

It is possible additional patients will be identified in a blinded fashion based on other terms.

## 5.5 Incidence of New Cerebral Infarct

Patients with the following MedDRA terms will be examined as part of a blinded medical review to determine whether the patient experienced a new cerebral infarct on Computed Tomography (CT) or Magnetic Resonance Imaging (MRI):

Cerebral infarction
Haemorrhagic cerebral infarction
Thrombotic cerebral infarction
Haemorrhagic stroke
Ischaemic stroke
Thrombotic stroke
Basal ganglia stroke
Brain stem stroke
Cerebral thrombosis

It is possible additional patients will be identified in a blinded fashion based on other terms.

## 5.6 Triple H therapy

The dedicated Triple H Therapy for Cerebral Vasospasm CRF will be used to determine for each patient whether such therapy was received.

## 5.7 SF-36 Quality of Life Survey

The SF-36 Quality of Life Survey is recorded at Day 28, 90 and 180. There are 36 questions in the SF-36 survey each of which are recoded into a value between 0 and 100 as presented in Table 4[3] where 100 represents the best outcome.

**Table 4. SF36 recoding individual responses**

| Item Numbers | Recorded Response Category | Recoded Score |
|---|---|---|
| 1, 2, 20, 22, 34, 36 | 1 → | 100 |

| | | |
|---|---|---|
| | 2 → | 75 |
| | 3 → | 50 |
| | 4 → | 25 |
| | 5 → | 0 |
| 3, 4, 5, 6, 7, 8, 9, 10, 11, 12 | 1 → | 0 |
| | 2 → | 50 |
| | 3 → | 100 |
| 13, 14, 15, 16, 17, 18, 19 | 1 → | 0 |
| | 2 → | 100 |
| 21, 23, 26, 27, 30 | 1 → | 100 |
| | 2 → | 80 |
| | 3 → | 60 |
| | 4 → | 40 |
| | 5 → | 20 |
| | 6 → | 0 |
| 24, 25, 28, 29, 31 | 1 → | 0 |
| | 2 → | 20 |
| | 3 → | 40 |
| | 4 → | 60 |
| | 5 → | 80 |
| | 6 → | 100 |
| 32, 33, 35 | 1 → | 0 |
| | 2 → | 25 |
| | 3 → | 50 |
| | 4 → | 75 |
| | 5 → | 100 |

Using these recoded scores, the Physical Health and Mental Health Score will be derived as follows[4]:

1. 35 of the 36 items will be assigned to one of eight subscales as defined in Table 5 and the mean of the recoded scores, $\bar{X}_i$ (i=1 to 8), calculated within each subscale, noting that Item 2 is not assigned to a subscale.
2. A Z score for each subscale, $Z_i$ (i=1 to 8), will then be derived as
$$Z_i = \frac{\bar{X}_i - x_i}{sd_i}$$
where $x_i$ and $sd_i$ are the population reference means and standard deviations for a healthy population. Table 6 displays the appropriate reference means.
3. Each $Z_i$ is multiplied by the corresponding Physical Health coefficient and summed to give an overall Physical Health Sum. The same approach is applied to the Mental Health coefficients to give an overall Mental Health Sum
4. The Physical Health and Mental Health Scores are then calculated by multiplying the respective Physical Health and Mental Health Sum by 10 and adding 50 to the product. A patient with responses typical of a general population will have a score of 50 and perfect health would correspond to Physical Health and Mental Health Scores of 57.87 and 62.14 respectively.

Whilst reference means for a US population are to be applied it has been shown that their application to a UK population gives nearly identical results for the Physical Health and Mental Health Score compared to the use of UK specific reference means[5].

Any patient who has died before the respective timepoint will be assigned a 0 recoded score for each item of the SF-36 prior to deriving the Physical Health and Mental Health Scores.

If less than 50% of the SF-36 questions are completed at a given timepoint or no responses are completed for an individual subscale, the SF-36 data will not be included in the analysis for that patient at that timepoint. If ≥50% of the SF-36 questions are completed, the mean of the observed individual item recorded scores within the respective subscales will be applied to the algorithm.

**Table 5. SF36 subscale items**

| Subscale | Number of Items | Item Numbers included |
|---|---|---|
| Physical functioning (PF) | 10 | 3 4 5 6 7 8 9 10 11 12 |
| Role limitations due to physical health (RP) | 4 | 13 14 15 16 |
| Role limitations due to emotional problems (RE) | 3 | 17 18 19 |
| Energy/fatigue (VT) | 4 | 23 27 29 31 |
| Emotional well-being (EW) | 5 | 24 25 26 28 30 |
| Social functioning (SF) | 2 | 20 32 |
| Pain (BP) | 2 | 21 22 |
| General health (GH) | 5 | 1 33 34 35 36 |

**Table 6. SF-36 Reference Values and Physical and Mental Health Coefficients**

| Subscale | Mean | SD | PH Coefficient | MH Coefficient |
|---|---|---|---|---|
| PF | 84.52 | 22.89 | 0.42402 | -0.22999 |
| RP | 81.20 | 33.80 | 0.35119 | -0.12329 |
| RE | 81.29 | 33.03 | -0.19206 | 0.43407 |
| VT | 61.05 | 20.87 | 0.02877 | 0.23534 |
| EW | 74.84 | 18.01 | -0.22069 | 0.48581 |
| SF | 83.60 | 22.38 | -0.00753 | 0.26876 |
| BP | 75.49 | 23.56 | 0.31754 | -0.09731 |
| GH | 72.21 | 20.17 | 0.24954 | -0.01571 |

PH = Physical Health, MH=Mental Health

## *5.8 CLCE-24*

The Checklist for Cognitive and Emotional Consequences (CLCE-24) is recorded at 90 and 180 days. The CLCE-24 instrument consists of 24 questions with possible responses of 'No', 'Yes, but not severe', 'Yes, severely hindering daily life' and 'I am not sure'. The problems are split into cognitive problems, corresponding to the first 13 questions, and emotional problems, corresponding to the next 9 questions. The final 2 questions solicit information on

DocuSign Envelope ID: 07B92F3A-AB49-4D0C-B36F-26112605A392



other specified problems; these will not be used in the analysis. Data will be analysed separately for emotional and cognitive problems. The number of questions, within each subscale, that has a response of either ‘Yes, severely hindering daily life’ or ‘Yes, but not severe’ will be calculated and this number will be recoded to a CLCE score as described in Table 7. The CLCE-24 score will be subject to statistical analysis.

If a patient dies before a given timepoint they will be assigned a score of 3. If less than 50% of the CLCE-24 questions are completed at a given timepoint the data will not be included in the analysis for that patient at that timepoint. Otherwise, the percentage of responses will be calculated using 24 as the denominator. In determining evaluability, a response of ‘I am not sure’ will be considered a completed response.

**Table 7. CLCE-24 Score**

| **Percentage of responses recorded as ‘Yes, severely hindering daily life’ or ‘Yes, but not severe’** | **Score assigned for analysis** |
|---|---|
| 0% | 0 |
| 0% < to < 25% | 1 |
| 25% ≤ to < 50% | 2 |
| ≥50% | 3 |

## *5.9 BICRO-39*

The Brain Injury Community Rehabilitation Outcomes Scale (BICRO-39) is recorded at 90 and 180 days and consists of 39 questions scored from 0= the most favourable response (no help or prompts, once a week or more, most or all days, several hours a day, more than 20 hours a week, never - as appropriate) to 5=the least favourable response (don’t do at all, not applicable or never, almost always – as appropriate). The BICRO-39 is divided into 8 subscales as presented in Table 8. The subscale score is calculated as the mean of the individual scores within that subscale.

An overall score will also be calculated as the mean score across the 34 individual scores that are not included in the contact with partner/children and contact with parents/siblings subscales. These scales are excluded as the desirable direction may vary between patients; some patients may wish to be less reliant on carers if they are relatives and in others alienation from family member maybe a problem[6].

Any patient who has died before the respective timepoint will be assigned a subscale and an overall score of 5 corresponding to the worst outcome. If less than 50% of the 39 questions are completed at a given timepoint, or no questions have been answered in one of the 6 subscales contributing to the overall score, none of the BICRO-39 data will be included in the analysis at that timepoint. Otherwise, if ≥50% but less than 100% of the BICRO-39 questions are completed, the mean of the recorded scores within the respective subscales will be used in the analysis of subscales. However in this case, the overall score will be calculated as a weighted average of the individual subscale scores with weights of 6/34 for all subscales included apart from productive employment which receives a weight of 4/34. This approach ensures that if missing data predominates in one subscale its contribution is not down-weighted in the overall score.

DocuSign Envelope ID: 07B92F3A-AB49-4D0C-B36F-26112605A392



**Table 8. BICRO subscales**

| Subscale | Number of items |
|---|---|
| Personal care | 6 |
| Mobility | 6 |
| Self-organisation | 6 |
| Contact with partner/children | 2 |
| Contact with parents/siblings | 3 |
| Socialising | 6 |
| Productive employment | 4 |
| Psychological well-being | 6 |

## *5.10 Subarachnoid Haemorrhage Outcomes Tool*

The Subarachnoid Haemorrhage Outcomes Tool (SAHOT) is recorded at Day 28, 90 and 180. days and consists of 56 questions scored 0 for 'No change', 1 for 'Some change' and 2 for 'Large or severe change'.   The only possible exception is the 'Quality of relationship with those closest' question, which, if the subsequent question indicates the relationship is better, will be scored as 0 regardless of the response.

The sum of the scores across all 56 questions is calculated to give a raw total score and depending on the outcome corresponds to a SAHOT category[7] (Table 9), which be analysed statistically.  If the patient has died prior to the timepoint they are assigned to the worst category of 9.

**Table 9. SAHOT categories**

| Raw score | SAHOT category |
|---|---|
| 0–7 | 1 (best outcome) |
| 8–17 | 2 |
| 18–29 | 3 |
| 30–42 | 4 |
| 43–56 | 5 |
| 57–73 | 6 |
| 74–89 | 7 |
| 90–112 | 8 |
| Not applicable | 9 (death) |

If less than 50% of the 56 questions are either not completed or no questions are completed in a subscale, the data will not be included in the analysis for that patient at that timepoint. Otherwise, the raw score will be scaled up prior to assigning the SAHOT category as follows:

$$\boldsymbol{Scaled\ raw\ score} = \sum_{j=1}^{3} \frac{1}{p_j} \sum_{i=1}^{n_j} x_i$$

where $x_i$ represents the individual question score for the $n_j$ recorded answers in the $j^{th}$ subscale and $p_j$ represents the proportion of intended questions answered for the $j^{th}$ subscale.

DocuSign Envelope ID: 07B92F3A-AB49-4D0C-B36F-26112605A392



This approach will ensure that if missing data predominates in one subscale it is not under-represented in the scaled raw score. A response of 'NA' will count as a completed response.

## 5.11 Length of acute hospital stay

The length of the acute hospital stay will be defined as the date of discharge (as recorded on the DISCHARG CRF) – date of ictus+1. Patients who die prior to discharge from tertiary care will be censored one day longer than the longest observed stay in either treatment group. If any patient has yet to be discharged at the time of the data cut-off they will be censored at the data cut-off date minus date of ictus+1.

A sensitivity analysis will be performed by setting the censored date of discharge at the date of death for patients who die prior to discharge.

Discharge location will be summarised only and not subject to formal statistical analysis.

## 5.12 MRI Susceptibility Weighted Imaging

These data will be summarised separately from the CSR.

## 5.13 Safety Data

Recording of AEs starts from informed consent and continues until at least 30 days post last dose. The definition of AEs and SAEs are given in the protocol.

Adverse events starting on or after the first treatment dose are therefore defined as Treatment Emergent Adverse Events (TEAEs). Any AE that starts more than 30 days past last dose and is considered to be at least possibly related to study treatment should also be defined as a TEAE. Any event starting before the first dose of study drug is identified as a Pre-Treatment Adverse Event (PTAE. Any event starting before the first dose of study drug that subsequently worsens is also be defined as a TEAE.

All events are coded using the MedDRA dictionary. The actual description of the AE is matched, as closely as possible, with the Lower Level Term in the dictionary.

All AEs are classified as described in Table 10.

**Table 10. Classification of AEs**

| AE Classification | AE Categories |
|---|---|
| Severity | Mild/Moderate/Severe/Life threatening |
| Serious | Yes / No |
| Relationship | Certain/Probable/Possible/Unlikely |
| Acton taken | Permanently discontinued/Stopped temporarily/Dose reduced/ Dose increased/ No action taken/ Unknown |
| Outcome | Recovered or resolved / Recovering or resolving / Not recovering or resolved / Recovered or resolved with sequelae / Fatal / Unknown |

Drug related events will be defined as ones whose relationship is given as certain, probable or possible. Any events with missing relationship to study drug will be tabulated as related. Any events with missing intensity will be tabulated in a separate category. If a patient has an AE which changes in severity this will be recorded as a separate occurrence of the same event. Serious adverse events (SAEs) are classified as described in the protocol. If a patient has more than one occurrence of the same AE and at least one is considered drug-related, the patient will be included as having a drug-related AE for that AE preferred term. Similarly, if a patient has more than one occurrence of the same AE and they have different severities, they will be summarised at the worst severity for that AE preferred term.

When summarising the time to onset of an AE the day of first dose will be used throughout as a reference point. If only a month is provided for date of onset, a conservative approach will be followed and events will be assumed to start at max(date of first dose, first day of the month). Likewise, if only a month is provided for the end date, the event will be assumed to have resolved at min(last date of month, date of onset for a new occurrence of the same AE for that patient).

For laboratory data all numeric blood results are classified by the investigator as Clinically Significant / Not Clinically Significant. All categorical urine results are classified as Clinically Significant (Yes / No). All blood and urine samples taken for safety evaluations are assayed by the local laboratory at each hospital. Data will be pooled across centres and summarised by treatment arm despite any differences in reference ranges.

## 5.14 Pharmacokinetic Data

No derived parameters for CSF and blood HP and MDA concentrations will be created. The method of analysis and presentation is described in a later section. This also applies to Sulforaphane (SFN) and SFN metabolite concentrations at Day 7 from patients who do not take part in the sub-study.

For patients entering the sub-study, SFN parent drug and metabolite concentrations are measured serially in both CSF and blood samples obtained pre-dosing and then hourly for up to 6 hours in a subset of 12 EVD patients on one of the first 3 doses and at Day 7. PK parameters for both CSF and blood will be derived for each individual patient using the concentrations over time profiles, data permitting. Full details of the pharmacokinetic analyses will be provided separately.

## 5.15 Time-Windows

The protocolled defined visit windows for assessments are +/-1 for Day 7, -2 at Discharge, -6/+2 at Day 28, +/-14 at Day 90 and +/-28 at Day 180. However, for the purpose of any timepoint analyses, data will be included at the visit closest to the time the recordings were taken.

DocuSign Envelope ID: 07B92F3A-AB49-4D0C-B36F-26112605A392



# 6 Statistical Methods

## 6.1 General Considerations

All data will be described and analysed according to treatment arm (SFX-01, Placebo) and day and time of assessment (if appropriate), for each patient population (PP, ITT, Safety, sub-study PK).

A (two-sided) significance level of 5% (<0.05) will be implemented throughout. The null hypothesis assumed throughout is that there is no difference between the active treatment and the control for any comparison performed (SFX-01 vs Placebo).

Part way through the trial the randomization was amended to stratify by WFNS score (1-3 v 4-5) and centre. For purposes of analyses that are stratified, WFNS score will be taken from the CRFs for patients randomized prior to the amendment and from the random scheme for patients randomized after the amendment.

## 6.2 Analysis Methods

Each endpoint subject to formal statistical analysis is listed in Table 11. To investigate the robustness of the primary analysis of each endpoint additional analyses will be performed. These are described as sensitivity analyses if they assess whether the results are influenced by departures from assumptions made in the primary analysis and are described as supplementary analyses if they assess a closely related measure not considered as important as the one chosen in the primary analysis.

**Table 11. Formal statistical analyses to be conducted and pre-planned supplementary and sensitivity analyses**

| Endpoint | Notes |
|---|---|
| **TCD** | Primary: maximum post-dose MCA mean flow velocity<br><br>Sensitivity analysis:<br>• Removing baseline data from the analysis model if it appears SFX-01 has had an effect by Day2<br>Supplementary analyses:<br>• Mean of 3 largest post-dose MCA mean flow velocities<br>• Maximum post-dose Lindergaard ratio<br>• Maximum MCA mean flow velocity per timepoint<br>• Mean effect over days 5 to 9 of maximum MCA mean flow velocity. |
| **Modified Rankin Scale** | Analysis at Day 7, 28, 90 and 180 and at discharge<br><br>Sensitivity analysis:<br>• If more than 15% of patients have missing data at Day 180 in either treatment arm, the analysis at each timepoint will be repeated by carrying forward the |

DocuSign Envelope ID: 07B92F3A-AB49-4D0C-B36F-26112605A392



| | |
|---|---|
| | outcome from the most recent timepoint with recorded data.. |
| **Glasgow Outcome Score - Extended** | Analysis at Day 28, 90 and 180<br><br>Sensitivity analysis:<br>• If more than 15% of patients have missing data at Day 180 in either treatment arm, , the analysis at each timepoint will be repeated by carrying forward the outcome from the most recent timepoint with recorded data.. |
| **Delayed Cerebral Ischaemia** | Proportion of patients with an event |
| **New Cerebral Infarct** | Proportion of patients with an event |
| **Triple H Therapy** | Proportion of patients with hypertensive (Triple H) therapy |
| **SF-36** | Physical and Mental Health scores at Day 28, 90 and 180.<br><br>Sensitivity analysis:<br>• If more than 15% of patients have missing data at Day 180 in either treatment arm, missing data diagnostics performed. |
| **CLCE-24** | Score based on the percentage of Cognitive and Emotional questions at Day 90 and 180 that indicate problems that severely hinder daily life<br><br>Sensitivity analysis:<br>• If more than 15% of patients have missing data at Day 180 in either treatment arm, missing data diagnostics performed. |
| **BICRO-39** | Primary: overall BICRO score at Day 90 and 180<br><br>Supplementary analyses:<br>• 8 subscale scores at Day 90 and 180<br>Sensitivity analysis:<br>• If more than 15% of patients have missing data at Day 180 in either treatment arm, missing data diagnostics performed. |
| **SAHOT** | SAHOT category at Day 28, 90 and 180<br><br>Sensitivity analysis:<br>• If more than 15% of patients have missing data at Day 180 in either treatment arm, missing data diagnostics performed |
| **Length of Acute Hospital Stay** | Primary: including patients who die prior to discharge censoring them 1 day longer than the largest observed stay<br><br>Sensitivity analysis;<br>• Censoring at the date of death as end of hospital stay for patients who die prior to discharge |
| **Blood & CSF HP and MDA** | Blood concentrations of HP & MDA at Day 7 and D28<br>CSF concentrations of HP & MDA at Day 7 |

DocuSign Envelope ID: 07B92F3A-AB49-4D0C-B36F-26112605A392



Analyses of the TCD, mRS and GOSE endpoints will be performed in both the PP and ITT populations with the PP population considered as primary. Other endpoints will initially only be analysed in the PP population and will only be analysed in the ITT population if there is a meaningful discrepancy in the results of endpoints analysed in both populations.

## 6.3 Multiplicity

The primary endpoint is the maximum post-dose MCA mean flow velocity.

Whilst no formal multiplicity adjustments will be made amongst the secondary endpoints, the two key secondary endpoints are:
1) Modified Rankin Score at 90 days
2) GOSE score at 90 days

Individual BICRO subscales will not be considered statistically significant unless the overall BICRO score is also statistically significant at the same timepoint.

The TCD data will analysed in advance of the other secondary endpoints. However, given that each endpoint will be analysed only once no adjustments will be made to the significance levels for each endpoint.

## 6.4 Analysis of the Primary Endpoint

All TCD endpoints will be $\log_e$-transformed prior to analysis including baseline values. The primary endpoint of maximum post-dose MCA mean flow velocity will be analysed using an Analysis of Variance (ANOVA) model with terms for treatment, baseline (as defined in Section 5.1), WFNS grade (1-3 v 4-5), hypertension (yes vs no), surgical procedure (clipping vs coiling vs none), log-CRP and age fitted a continuous covariate. The statistical significance of treatment will be based on type III sums of squares. The supplementary analyses of the mean of the 3 largest post-dose MCA mean flow velocities and the maximum post-dose Lindergaard ratio will use the same methods.

The maximum MCA mean flow velocity per timepoint will be analysed using a mixed model repeated measures (MMRM) approach with visits grouped, relative to date of Ictus, as Days 3-4, 5-6, 7-9, 10-14, 15-21, 22-28 and baseline covariates as defined for the primary analysis. Treatment effects for each visit will be obtained from the same model using the corresponding treatment-by-visit estimate. The MMRM will include terms for treatment, visit, treatment-by-visit, baseline-by-visit, covariates and observations blocked by subject. An unstructured covariance matrix will be used to model the within-subject error, along with restricted maximum likelihood and the Kenward-Roger approximation to estimate the degrees of freedom. If the model will not converge with an unstructured covariance matrix, heterogenous Toeplitz and Toeplitz patterns will be tried in that order until the model will converge.

An example of the corresponding SAS code is given below

```
PROC MIXED data=data1 method=reml;
 CLASS trt vis pat wfns htn surg;
 MODEL lnTCD=base trt vis trt*vis base*vis age wfns htn surg lcrp / ddfm= KR;
```

DocuSign Envelope ID: 07B92F3A-AB49-4D0C-B36F-26112605A392



```
 REPEATED vis / type=un subject=pat;
 LSMEANS trt*vis / slice=vis;
RUN;
```

The mean effect over days 5 to 9 will be estimated from the same model using an appropriate ESTIMATE statement weighting each timeperiod equally.

The treatment effect for each analysis will be presented in terms of a ratio (SFX-01:placebo), representing the ratio of the geometric lsmeans (glsmean) for each treatment arm, together with its 95% confidence interval. The glsmean for each arm will be calculated by exponentiating the lsmeans produced from the analysis of the $\log_e$-transformed data.

The assumptions of normality will be assessed by normal probability plots of the residuals and plotting standardized residuals versus predicted values.

Many of the baseline values used in the analysis will have been recorded shortly after the patient has been dosed with randomized treatment but before it is anticipated any treatment effect will merge, will be included. If there is any evidence that SFX-01 has had an effect by Day2 a sensitivity analysis will be performed where the baseline term is removed from the ANOVA model and centre is added to the list of covariates.

## *6.5 Analysis of Secondary Efficacy Endpoints*

The mRS, GOSE, SAHOT and CLCE will be analysed using a proportional odds logistic regression model to each timepoint separately including the following covariates: treatment, age (fitted as continuous variable), WFNS score (fitted as a class variable), hypertension (yes vs no), surgical procedure (clipping vs coiling vs none) and log-CRP. The treatment effect will be described using an odds ratio together with its likelihood ratio based 95% confidence interval. The statistical significance of the treatment effect will be taken from the chi-square test for the difference in deviance when removing treatment from the model. GOSE scores will be reversed prior to analysis so the odds of a better response is being modelled. PROC LOGISTIC can be used to analyse the data. If the model does not converge, any covariates that are not statistically significant will be removed in order from least significant until the model converges. If the model still does not converge, the outcome level with the fewest observations will be combined with the next most severe outcome and this process repeated until convergence is achieved.

BICRO and SF-36 will be analysed using Van-Elteren’s test with 4 strata defined by age (above and below the median) and WFNS score (1-3 v 4-5). The treatment effect will be described using an un-stratified Hodges-Lehmann (HL) estimate of median difference together with its 95% confidence interval. The test can be implemented in SAS as follows:

```
proc npar1way data=data1 HL;
 strata strata/wilcoxon;
 class trt;
 var resp;
 run;
```

The proportion of patients with delayed cerebral ischaemia, new cerebral infarct and receiving triple H therapy will be analysed using logistic regression with the same set of covariates used for mRS. The statistical significance of the treatment effect will be taken from the chi-square test for the difference in deviance when removing treatment from the model. The treatment

DocuSign Envelope ID: 07B92F3A-AB49-4D0C-B36F-26112605A392



effect will be described in terms of an odds ratio together with its likelihood ratio based 95% confidence interval.

The length of acute hospital stay will be analysed using a Cox proportional hazards model with the same set of covariates used for mRS. The statistical significance of the treatment effect will be taken from the chi-square test for the difference in deviance when removing treatment from the model. The treatment effect will be described in terms of a hazard ratio together with its profile likelihood 95% confidence interval. In addition, the median will be reported and taken from the $50^{th}$ percentile of the associated Kaplan-Meier curves.

## 6.6 Analysis of Pharmacokinetic Endpoints

Blood concentrations, $\log_e$-transformed prior to analysis, of HP & MDA at Day 7 and D28 will be analysed by MMRM using the same model as described in the analysis of TCD data. Both EVD and non-EVD data will be included in the same model. This model will be used to derive separate treatment effects at Day 7 and Day 28. The baseline term in the models will be the blood concentrations recorded between pre-dose and 48h. A separate summary of the baseline concentrations will confirm that no treatment effect has emerged by 48h by summarising data according to whether data were collected pre-dose, within 24h and between 24 and 48h after dosing. If there is evidence of an effect within 48h, the nominal baseline values will be fitted as a separate visit and a covariate added indicating whether the patient had an EVD sited or not.

$\text{Log}_e$-transformed CSF concentrations of HP & MDA at Day 7 will be analysed using an ANCOVA model with a 2-level covariate denoting whether the CSF values were taken from an EVD or lumbar puncture (LP). A treatment-by-covariate (EVD vs LP) interaction will be added to assess whether the effect of SFX-01 differs by whether the CSF was obtained from EVD or LP together with subgroup analyses for EVD and LP patients separately.

In all cases, the treatment effect will be presented as the ratio of glsmeans and associated 95% confidence interval having exponentiated the lsmeans and confidence limits calculated on the $\log_e$-scale.

Other pharmacokinetic measurements will be summarised only.

## 6.7 Missing Data Diagnostics

For the mRS, GOSE, SAHOT, CLCE, BICRO and SF-36 endpoints, if more than 15% of patients have missing data for the respective endpoint in either treatment arm at Day 180, the possible impact of these data on the primary analyses will be explored as follows:

- For all endpoints the number of patients with missing data will be summarised. In addition, baseline WFNS score (1-3 v 4-5) will be summarized by treatment arm for each endpoint at each timepoint according to whether data were missing at that timepoint.
- For mRS and GOSE only the analysis at each timepoint will be repeated by carrying forward the outcome from the most recent timepoint with recorded data.

The purpose of these analyses is to assess whether the amount of missing data differs by treatment arm and whether patients with missing data initially had a worse prognosis or were performing worse on other measures recorded at the same timepoint. For example, if there

are more patients in the placebo arm with missing data and those that have missing data were tending to respond less well, this would suggest the treatment effect may have been underestimated.

If the results are marginal for a particular endpoint these investigations may be performed despite neither arm having 15% of patients with missing data. Dependent on results further analyses may be performed to assess the possible impact of missing data.

For TCD endpoints it is not anticipated that missing data could have a bearing on the outcome of the results given the proximity of the measurements to dosing.

Only the year of birth has been recorded. When calculating age, a date of birth of 1st July will be assumed.

## 6.8 Subgroup analyses

Subgroup analyses will be performed for maximum post-dose MCA mean flow velocity, mRS at 90 days and GOSE at 90 days for the following variables: centre, WFNS score (1-3 v 4-5), Fisher grade, time from ictus (<24h, 24-48h, >48h), Age group (≤49, 50-59,≥60), gender, surgical procedure (coiling, clipping, not done), location of aneurysm (anterior, posterior), main vessel of aneurysm (anterior, middle, internal, vertebrobasilar) and whether the patient developed a secondary bleed prior to randomisation. Additionally, a subgroup analysis of the primary TCD endpoint will be performed by baseline value split according to quartiles. Results will not be presented for any subgroup level that contains fewer than 10 patients.

Data will be analysed separately for each level of the subgroup. For the primary TCD endpoint, the ANOVA model will be re-run and the treatment effect and associated 95% confidence intervals presented. For mRS and GOSE at 90 days, the logistic regression model will performed within each level of the subgroup with the odds ratio and associated 95% confidence intervals presented. Results from all subgroups will be presented in a forest plot for each endpoint separately.

If there is evidence of heterogeneity of the treatment effect amongst subgroups the following tests will be performed:

1. A treatment-by-subgroup interaction term will be added to a model containing the terms included in the primary analysis model together with the main effect of the subgroup if not already included in the model. The p-value for the change in model fit by adding the interaction term will be presented
2. A test of the collective heterogeneity amongst subgroups will performed. This will be achieved by comparing a base model including treatment and all main effects of subgroups with a model containing all two-way interactions of treatment and subgroup. The p-value for the change in model fit by adding all of the interaction terms simultaneously will be presented and described as a global interaction test.

Age will be fitted as a continuous covariate in the interaction tests.

## 6.9 Changes from Protocol

DocuSign Envelope ID: 07B92F3A-AB49-4D0C-B36F-26112605A392



1. The use of modelling rather than hypothesis testing where possible to allow more powerful analysis through inclusion of covariates and the generation of treatment estimates with confidence intervals.
2. Refinement of the per-protocol definition to consist of patients who receive at least 10 doses of randomised treatment within the first 7 days post-ictus and to exclude patients with potential dispensing errors.
3. Clarification that mRS and GOSE at 90 days are key secondary endpoints

DocuSign Envelope ID: 07B92F3A-AB49-4D0C-B36F-26112605A392



# 7 Presentation of Data

This section describes the presentation of the results, supporting displays will be detailed in a separate document.

***General Information***

All continuous data will be presented as means, standard deviation, minimum, maximum, median, lower quartile, and upper quartile as well as number of observations for both actual values and change from baseline data. $Log_e$-transformed data, PK and TCD, will be summarised using geometric mean, coefficient of variation (CV), minimum, maximum, median, lower quartile, and upper quartile as well as number of observations. CV will be calculated as sqrt(exp(sigma$^2$)-1), where sigma is the standard deviation of the $log_e$-transformed data. All descriptive summaries will be displayed to one more decimal place than actually measured. All categorical data will be presented in contingency tables as frequencies and percentages and the denominator will be the number of patients available in the relevant population who have data recorded.

All the tables will be summarised using the treatment arm allocation (SFX-01, Placebo), day and time post ictus or dosing as appropriate. Patients will be assigned to time windows as described in Section 5.15. Any assessments not assigned to time-windows, as other measurements are closer, will be flagged in the appropriate supporting listings.

All demographic summaries will be produced for both the ITT and PP populations according to treatment arm (SFX-01, Placebo). Summaries of TCD, mRS and GOSE data will also be produced for both the ITT and PP populations according to treatment arm (SFX-01, Placebo). Other secondary efficacy endpoints will be produced for only the PP population. Safety summaries and pharmacokinetic data reported outside of the sub-study will be produced for the Safety population and non-compartmental PK data from the sub-study will be summarised for the Sub-Study population only.

***Data presentations***

***Disposition***
A categorical summary of patient status detailing the number and percentage of: Screened, Randomized, Treated, Died, Completed will be produced by treatment arm. The denominator for percentages will be the number randomized so no percentage will be presented for the Screened row. This summary will be repeated for the patients in the PK sub-study. The number and percentage of subjects in each patient population (PP, ITT, Safety, PK) will also be given as well as the number of patients who have an EVD sited. Amongst patients who did not complete the study, the reason for withdrawal will also be summarised together with a cumulative incidence plot of time to withdrawal by treatment arm produced for both the PP and ITT populations.

***Major Protocol Violations And Deviations***
Major violations are to be summarised by treatment arm (SFX-01, Placebo) and overall for all patients and will include the nine patients associated with potential dispensing errors. The number of patients not meeting all inclusion and exclusion criteria will be summarised by treatment arm along with the reason. Supporting listings for both violations and deviations will be included which identify patient, treatment arm and the assigned population.

DocuSign Envelope ID: 07B92F3A-AB49-4D0C-B36F-26112605A392



***Qualifying Evaluations (Surgery, Eligibility And Screening Procedures)***

Dates and times of all qualifying evaluations will be listed as: Onset of Ictus, Surgery (plus WFNS and any Triple H Therapy for Cerebral Vasospasm), Eligibility, Investigator Consent, Randomisation and Dosing. Other reported dates included: Discharge, MRI at follow up and End Of Study (EOS).

The time between Ictus and the patient receiving their first dose of randomized medication will be summarized for both the ITT and PP populations by treatment arm.

***Surgery Details***

Surgery details will be summarised for the ITT and PP population by treatment arm. Type of scan (CT/MRI), Angiographic assessment performed (CTA/ DSA/MRA), Fisher grading (No haemorrhage, SAH <1mm, SAH>1 mm, SAH any thickness with IV haemorrhage or parenchymal extension) and Surgical procedure (Coiling/Clipping), Triple H therapy for Cerebral Vasospasm (Yes/No) and WFNS (Grading I, II, III, IV, V).

***Eligibility And Screening Procedures***

Demographic data will be summarised for the ITT and PP population by treatment arm which will include a summary of the data recorded on aneurysm location and whether the patient developed a secondary bleed prior to randomisation. Medical History, Physical Examination, Pregnancy Test (if applicable) and Prior Medications will be summarised for the Safety population by treatment arm.

***Concomitant Medications***

All medications taken after the start of treatment are summarised according to WHODD ATC categorised through to level 4.

***Randomisation, Dosing And Drug Accountability***

All patient randomisation details will be listed indicating the treatment randomised and the treatment actually received. Any miss-randomisations will be flagged. In particular, the intended and actual treatment received on each day for the nine patients associated with potential dispensing errors will be listed.

Exposure to study drug will be described by summarising the dosing duration (date of last dose – date of first dose +1), total number of doses given and compliance (100*total number of doses given /[2*dosing duration]/) by treatment arm for both the PP and Safety populations. Individual details will be listed.

***TCD***

The maximum post-dose MCA mean flow velocity and the corresponding baseline value (see Section 5.1) will be summarised according to treatment arm (SFX-01, Placebo) for both the PP and ITT populations. The number of patients who have data imputed at baseline will also be summarised by treatment arm. In addition, the baseline values will be split according to whether they occurred before dosing, on Day1 and Day 2 and summarised by treatment arm.

Results of the statistical analysis will be presented as described in Section 6.4. A graphical presentation of the treatment effects and lsmeans over time will be produced using the results of the MMRM analysis.

***Glasgow Coma Score (GCS)***

DocuSign Envelope ID: 07B92F3A-AB49-4D0C-B36F-26112605A392



The GCS daily scores (except Baseline) will be summarised as continuous data for both the both the best and worst daily score and for both the PP and ITT populations. Patients will be grouped into time windows as Days 3-4, 5-6, 7-9, 10-14, 15-21, 22-28.

***Secondary Efficacy Endpoints***

For mRS, CLCE-24, SAHOT and GOSE the percentage and cumulative percentage (best to worst score) of patients in each category will be summarised by treatment arm. SF-36 and BICRO-39 will be summarised as continuous data by treatment arm. The number and percentage patients with DCI, new cerebral infarct and institution of triple H therapy will be summarised by treatment arm together with a cumulative incidence plot displaying the time to each of these events across patients by treatment arm. A Kaplan-Meier (KM) curve of the length of acute hospital stay, will be presented along with an associated table presenting the $25^{th}$, $50^{th}$ (median) and $75^{th}$ percentiles derived from the KM curve. Discharge location will be summarised by treatment arm.

The results of the statistical analyses of all secondary endpoints will be presented as described in Section 6.5. Specified missing data diagnostics will also be presented by treatment arm as necessary as described in Section 6.7. Subgroup analyses will be presented as described in Section 6.8.

***Pharmacokinetic Endpoints***

The results of the analysis of blood HP and MDA concentrations at Day 7 and 28 and CSF HP and MDA concentrations at Day 7 will be presented as described in Section 6.6. The blood and CSF HP and MDA concentrations will be summarised by treatment arm at all timepoints noting that EVD patients have concentrations recorded at extra timepoints. For timepoints in common to both EVD and non-EVD patients data will pooled across groups as well as presenting the data split by the use of an EVD.

For patients in the sub-study, the derived PK parameters in both CSF and blood will be summarised as described for $\log_e$-transformed data except for Tmax where the geometric mean and CV will not be calculated and median presented. Supporting graphical displays of individual and mean profiles will be produced by sampling day.

Individual SFN and SFN metabolite concentrations recorded on Day 7 and any concentrations recorded in the sub-study for patients outside of the sub-study using the summary measures described for $\log_e$-transformed data pooled across EVD and non-EVD groups. If <50% of concentrations are below the lower limit of quantification (LOQ) then data will be summarised substituting the LOQ in the calculation of the geometric mean and CV, otherwise these two parameters will not be presented. The median, LQ and UQ will be displayed as 'NQ' if those statistics correspond to a value below the LOQ. These summaries will only be produced for the SFX-01 group.

Note proteomic and genetic data will not be summarised as part of the CSR.

***Adverse Events (AEs)***

MedDRA coded events will be summarized by treatment arm and overall. The number of patients reporting adverse events will be presented according to the coded Preferred Term and System Organ Class (SOC).

DocuSign Envelope ID: 07B92F3A-AB49-4D0C-B36F-26112605A392



An overview table of the number and percentage of patients with any Pre-Treatment Adverse Event (PTAE), TEAEs, Deaths, Serious TEAEs, All Serious AEs, TEAEs leading to discontinuation, maximum severity of TEAEs and Drug-Related TEAEs will be presented as a categorical summary according to treatment arm and overall. In the maximum severity row, patients will be counted once according to the worst severity categorized as missing/mild/moderate/severe/life threatening.

The following summarized of AEs will be produced:

- number and percentage of patients with at least one PTAE by Preferred term
- number and percentage of patients with at least one TEAE by Preferred term
- number and percentage of patients with at least one TEAE by Preferred term by maximum severity
- number and percentage of patients with at least one TEAE by Preferred term that is related to study drug
- number and percentage of patients with at least one TEAE by Preferred term that is serious
- number and percentage of patients with at least one adverse event that is serious, to include any serious AEs that occurred prior to dosing with study medication
- number and percentage of patients with at least one TEAE by Preferred term that lead to discontinuation of treatment corresponding to events where action taken with study treatment is 'Permanently discontinued'

In each table events will be grouped according to SOC and within each SOC events will be presented in order of decreasing frequency. The SOC row will present the number of patients with at least one TEAE within that SOC for the subset of events being summarized.

Serious TEAEs will be listed by patient and treatment arm, indicating the name of the AE, PT and SOC, together with details of the start and end dates, event duration (days) and maximum intensity. Additionally, the relation to study drug, action taken, outcome and the date of the first dose of study drug will also be displayed.

***Deaths***

All deaths during the study will be summarised by treatment arm (from EOS form). Details will also be listings. A KM plot of time to death measured from the date of ictus will be presented by treatment arm. Patients alive will be censored at the visit date completed for the End of Study CRF.

***Laboratory Safety Assessments***

Safety blood including lipid and coagulation tests performed throughout the study will be summarised as continuous variables by visit and treatment arm. Both actual values and change from baseline will be summarised. In addition, changes from baseline will displayed on a box-plot by visit and treatment arm for each parameter. Clinically significant findings for each parameter will be summarised in a shift-table by treatment arm, where the clinical significance of values recorded within 48h of ictus will determine the row and whether the patient has any later value recorded that is clinically significant will determine the column. Percentages will use the total number of patients in each row as a denominator. Corresponding shift tables will be produced for urine sample parameters.

DocuSign Envelope ID: 07B92F3A-AB49-4D0C-B36F-26112605A392



***Data Listings***

All data presented in summary tables will be supported by specific referenced data listings. These will identify the subject number, cohort number, treatment arm (SFX-01, placebo) Visit, Dose number, Dose day (if appropriate) and provide actual data or derived data as appropriate.

Listings will be created according to the ICH guidelines, as appropriate for the data collected.

DocuSign Envelope ID: 07B92F3A-AB49-4D0C-B36F-26112605A392



# 8 References

1- ICHE9 “Statistical Principles For Clinical Trials” February 1998.

2- ICHE8 “General Considerations For Clinical Trials” July 1997.

3 - ***https://www.rand.org/health/surveys_tools/mos/36-item-short-form/scoring.html***

4 -Taft C, Karlsson J, Sullivan M. Do SF-36 summary component scores accurately summarize subscale scores? Quality of Life Research (2001) 10:395-404

5 -Jenkinson C. Comparison of UK and US methods for weighting and scoring the SF-36 summary measures. J Public Health Medicine (1999) 21:372-376

6 – Powell J, Heslin J, Greenwood R. Community based rehabilitation after severe traumatic brain injury: a randomised controlled trial. J Neurol Neurosurg Psychiatry (2002);72:193–202

7 – Pace A, Mitchell S, Casselden E, Zolnourian A, Glazier J, Foulkes L, Bulters D, Galea I. A subarachnoid haemorrhage-specific outcome tool. Brain (2018); 141:1111-1121
