# Supplementary material for: A Randomised Controlled Trial of SFX-01 After Subarachnoid Haemorrhage — The SAS Study
Source: Transl Stroke Res. 2024 Jul 19;16(4):1031–43. doi: 10.1007/s12975-024-01278-1 (PMC12202693; doi:10.1007/s12975-024-01278-1)
Supplement: Supplementary file 5 — Supplementary file5 - Figures (DOCX 232 KB) [file 12975_2024_1278_MOESM5_ESM.docx]

**SAS Study Supplemental Figures**


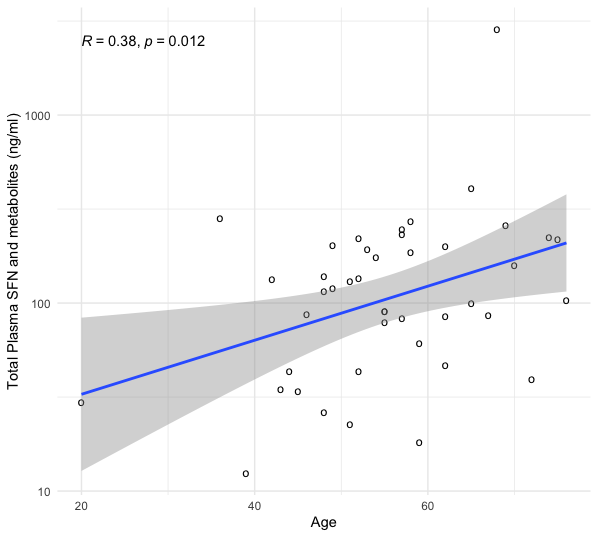


**Figure S1. Mean total plasma SFN and metabolites on day 7 vs age. Pearson correlation coefficient (R) and linear regression line with 95% confidence intervals. 43 patients taking SFX-01 per protocol included.**


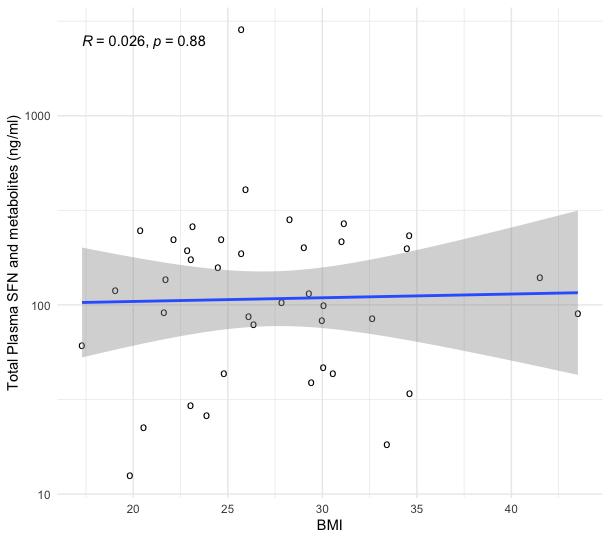


**Figure S2 Mean total plasma SFN and metabolites on day 7 vs Body Mass Index (BMI). Pearson correlation coefficient (R) and linear regression line with 95% confidence intervals. 43 patients taking SFX-01 per protocol included.**


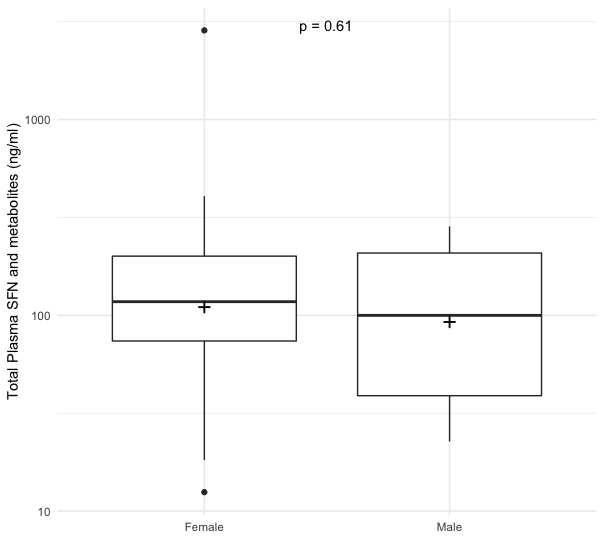


**Figure S3 Total Plasma SFN and metabolites (SFN + SFN-GSH + SFN-NAC) for male and female patients. 43 patients taking SFX-01 per protocol included with Plasma samples included. Values below the lower limit of quantification (LLOQ SFN 5mg/ml, SFN-GSH 10mg/ml, SFN-NAC 5mg/ml for both plasma and CSF) are represented as the midpoint of 0 and the LLOQ. P-value on t-test. Means depicted with +.**


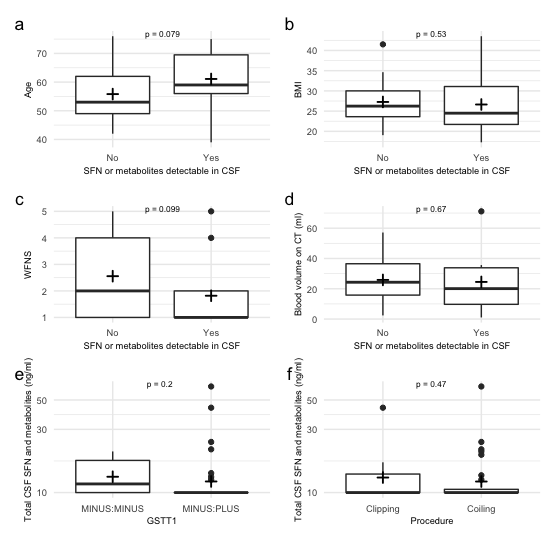


**Figure S4 Relationship between baseline variables and detection of SFN or metabolites in CSF in patients receiving SFX01 per protocol.**

**Blood volume on CT was measured in ml on baseline CT scans performed within 48 hours of ictus and prior to recruitment. Manual segmentation of blood was performed and quantified using MIPAV (Medical Image Processing, Imaging and Visualization) v11.0. The CT image signal intensity threshold was set between 50 and 80 Hounsfield units and converted to a binary mask. Regions of interest representing blood clot were drawn manually on each slice and summed into single 3-dimensional volumes.**


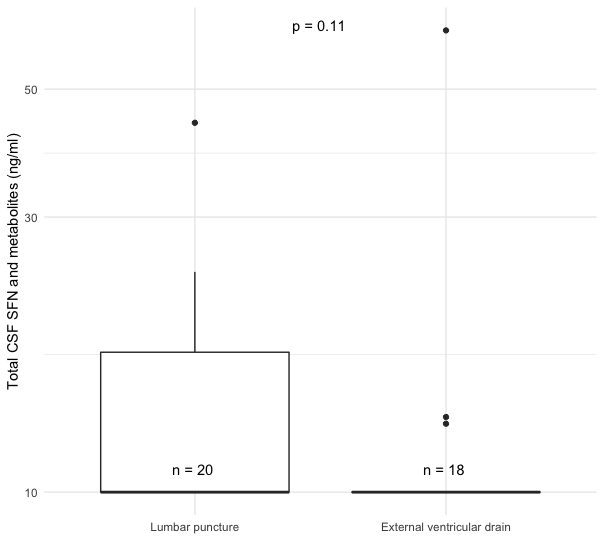


**Figure S5 Total CSF SFN and metabolites for samples obtained via lumbar puncture and external ventricular drain on day 7. Patients taking SFX-01 per protocol with CSF samples included. Values below the lower limit of quantification (LLOQ SFN 5mg/ml, SFN-GSH 10mg/ml, SFN-NAC 5mg/ml for both plasma and CSF) are represented as the midpoint of 0 and the LLOQ. There was no significant difference between groups detected (Wilcoxon signed rank W = 224, p = 0.112).**


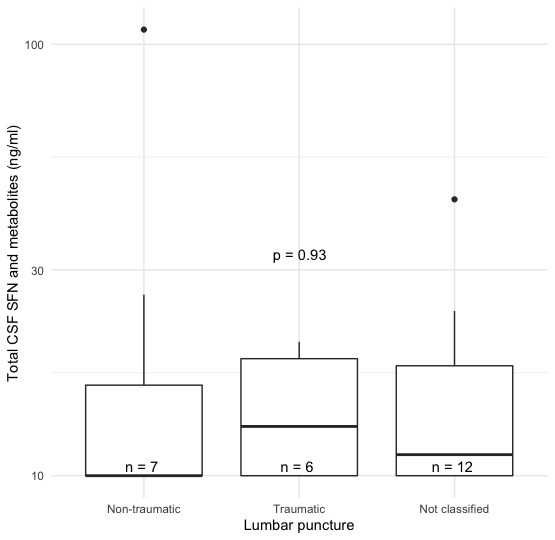

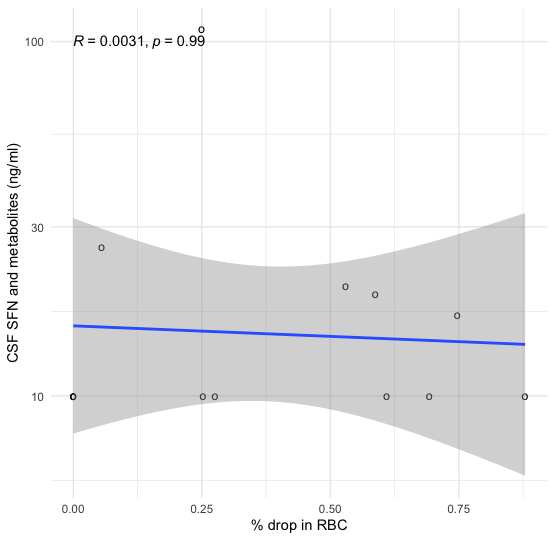


**Figure S6 a) Total SFN and metabolites in CSF obtained by lumbar puncture classed by whether it the lumbar puncture was traumatic or not. Non traumatic classed as a drop in red blood cell counts between 1^st^ and 4^th^ bottle of <30% and traumatic as >30% based on Gorchynski et al, and those missing a first or last sample as not classified. There was no significant difference between groups detected (Kruskal-Wallis Chi Square = 3.04, p = 0.219).**

**b) Relationship between the percentage drop in red blood cells (RBC) between first and last bottle of CSF and total CSF SFN and metabolites. All patients allocated to SFX-01 with CSF samples included and 1^st^ and 4^th^ bottle RBC shown. Spearman correlation coefficient (R) and linear regression line with 95% confidence intervals. Values below the lower limit of quantification (LLOQ SFN 5mg/ml, SFN-GSH 10mg/ml, SFN-NAC 5mg/ml for both plasma and CSF) are represented as the midpoint of 0 and the LLOQ.**
