# Supplementary material for: A Randomised Controlled Trial of SFX-01 After Subarachnoid Haemorrhage — The SAS Study
Source: Transl Stroke Res. 2024 Jul 19;16(4):1031–43. doi: 10.1007/s12975-024-01278-1 (PMC12202693; doi:10.1007/s12975-024-01278-1)
Supplement: Supplementary file 6 — Supplementary file6 - Methods (DOCX 14 KB) [file 12975_2024_1278_MOESM6_ESM.docx]

SAS Study Supplemental Methods 2

**Sulforaphane sample collection**

Plasma was collected in 6.0 mL K_2_EDTA collection tubes, containing 0.1 mL of 0.5M citric acid, inverted 8 times, placed on wet ice and centrifuged within 10 minutes at 1500 rcf for 10 min at 4°C before storing at -80. CSF (5mL) was collected in 30 mL universal tubes, containing 0.1 mL of 0.5M citric acid (after discarding the first 3ml if obtained from an EVD). The first 1ml of CSF was used for cell count analysis, and the next 10ml were centrifuged and stored, similar to plasma, for pharmacodynamic analyses.

**Sulforaphane quantification**

SFN and its metabolites, sulforaphane glutathione (SFN-GSH) and sulforaphane N-acetyl cysteine (SFN-NAC), were quantified by a Good Laboratory Practice (GLP) accredited laboratory (Alderley Analytical) using LC-MS/MS (Waters I-Class UPLC, coupled to a Waters TQ-S Mass Spectrometer) calibrated over 5 to 2000 ng/mL for SFN and SFN-NAC, and 10 to 2000 ng/mL for SFN-GSH. The validation report is in supplementary methods validation report.

**Malondialdehyde and Haptoglobin quantification**

Malondialdehyde was quantified using a standard colorimetric method (NorthWest Life Science Specialties), which relies on the detection of the reaction product between MDA and thiobarbituric acid, which absorbs strongly at 532 nm.

Haptoglobin was analyzed by rate nephelometry on a Beckman Coulter IMMAGE immunochemistry system, certified to clinical standards. Since haptoglobin determination in CSF requires a lower limit of detection than the Beckman Coulter system provides, an in house validated ultra-performance liquid chromatography (UPLC) assay that measures haptoglobin in the CSF was employed^1^; with this method, haptoglobin concentration in the CSF is expressed in terms of the concentration of haemoglobin it is able to bind.

1. Garland P, Morton MJ, Haskins W, et al. Haemoglobin causes neuronal damage in vivo which is preventable by haptoglobin. *Brain Commun*. 2020;2(1). doi:10.1093/braincomms/fcz053
