# Supplementary material for: A Randomised Controlled Trial of SFX-01 After Subarachnoid Haemorrhage — The SAS Study
Source: Transl Stroke Res. 2024 Jul 19;16(4):1031–43. doi: 10.1007/s12975-024-01278-1 (PMC12202693; doi:10.1007/s12975-024-01278-1)
Supplement: Supplementary file 7 — Supplementary file7 - Tables (DOCX 28 KB) [file 12975_2024_1278_MOESM7_ESM.docx]

**SAS study Supplemental tables**

| **Treatment emergent adverse events** | **SFX-300 mg** | **Placebo** |
| --- | --- | --- |
| Acute Kidney Injury | 3 | 0 |
| Anaemia | 1 | 3 |
| Arachnoiditis | 1 | 0 |
| Ataxia | 1 | 0 |
| Atrial fibrillation | 4 | 1 |
| Blurred Vision | 1 | 0 |
| Confusion | 1 | 3 |
| Constipation | 1 | 3 |
| Cough | 1 | 0 |
| Delayed Cerebral ischaemia | 12 | 7 |
| Deranged LFTs | 46 | 30 |
| Diarrhoea | 2 | 2 |
| Dry skin | 0 | 1 |
| Dysphagia | 1 | 0 |
| Epistaxis | 1 | 1 |
| Folliculitis | 0 | 1 |
| Hallucinations | 2 | 1 |
| Headache | 5 | 2 |
| Hydrocephalus | 10 | 7 |
| Hypercholesterolaemia | 10 | 14 |
| Hyperglycaemia | 1 | 1 |
| Hyperkalaemia | 1 | 1 |
| Hypernatraemia | 6 | 4 |
| Hypoalbuminaemia | 3 | 3 |
| Hypocalcaemia | 2 | 1 |
| Hypokalaemia | 16 | 13 |
| Hyponatraemia | 15 | 15 |
| Lower respiratory tract infection | 22 | 15 |
| Migraine | 1 | 0 |
| Nausea | 9 | 1 |
| Neutropenia | 0 | 1 |
| Oral thrush | 0 | 1 |
| Photophobia | 1 | 2 |
| Pruritis | 0 | 1 |
| Pulmonary oedema | 6 | 2 |
| Raised fibrinogen | 4 | 6 |
| Raised urea | 2 | 3 |
| Rash | 0 | 2 |
| Retinal artery occlusion | 0 | 1 |
| Seizure | 1 | 2 |
| Tachycardia | 2 | 0 |
| Thrombocytopenia | 1 | 0 |
| Thrombocytosis | 2 | 1 |
| Tinnitus | 1 | 0 |
| Urinary tract infection | 10 | 17 |
| Vasovagal | 1 | 0 |
| Ventriculitis | 5 | 3 |
| Vitreous haemorrhage | 7 | 1 |
| Vomiting | 5 | 1 |

**Table S1 A list of treatment emergent adverse events in the safety population that received at least one dose of SFX-01 or placebo.**

|  | AUC_last_ (h×ng/ml) | C_max_ (ng/ml) | T_max_ (h) |
| --- | --- | --- | --- |
| SFN | 16.2 (0.725-362) | 23.6 (2.12-263) | 2.6 (1.52) |
| SFN-GSH | 277 (77.0-995) | 118 (35.0-399) | 2.5h (1.69) |
| SFN-NAC | 415 (147-1170) | 129 (36.7-452) | 2.25h (1.16) |

**Table S2 Day 7 plasma concentrations of SFN, SFN-GSH and SFN-NAC. AUC_last_ geometric mean in h×ng/ml (-sd +sd), C_max_ geometric mean in ng/ml (-sd +sd), and T_max_ arithmetic mean (sd). SFN based on 5 patients with available data.**

|  | SFX-01 |  | Placebo |  |  |  |  |
| --- | --- | --- | --- | --- | --- | --- | --- |
| Time | n | GLS mean | n | GLS mean | Ratio of means | 95% CI | P value |
| Serum haptoglobin (g/L) | | | | | | | |
| Baseline | 41 | 1.26 | 37 | 1.55 |  |  |  |
| Day 7 | 39 | 2.39 | 36 | 2.55 | 0.937 | 0.791-1.109 | 0.446 |
| Day 28 | 35 | 1.72 | 35 | 1.67 | 1.031 | 0.865-1.228 | 0.731 |
| CSF haptoglobin (mg/L) | | | | | | | |
| Day 7 | 40 | 2.27 | 37 | 1.17 | 1.981 | 0.992-3.786 | 0.052 |
| Plasma malondialdehyde (μM) | | | | | | | |
| Baseline | 42 | 0.40 | 37 | 0.37 |  |  |  |
| Day 7 | 42 | 0.34 | 37 | 0.38 | 0.907 | 0.737-1.116 | 0.355 |
| Day 28 | 35 | 0.30 | 35 | 0.31 | 0.974 | 0.785-1.209 | 0.810 |
| CSF malondialdehyde (g/L) | | | | | | | |
| Day 7 | 40 | 0.116 | 37 | 0.103 | 1.123 | 0.747-1.687 | 0.572 |

**Table S3. Mixed Models Repeated Measures analysis of serum haptoglobin and plasma malondialdehyde and analysis of variance for CSF haptoglobin and CSF malondialdehyde in the per protocol population. Mixed Models Repeated Measures analysis included terms for treatment, visit, log-baseline, WFNS grade, hypertension, surgical procedure, log-CRP, age, treatment*visit, log-baseline*visit. CSF haptoglobin and malondialdehyde were modelled with an analysis of variance including terms for treatment and CSF source. The geometric least squares (GLS) mean is presented.**

| SFX-01 |  | Placebo |  |  |  |  |
| --- | --- | --- | --- | --- | --- | --- |
| n | GLS mean | n | GLS mean | Ratio of means | 95% CI | P value |
| Maximum MCA Flow Velocity at any time | | | | | | |
| 44 | 122 | 41 | 116 | 1.046 | 0.903-1.211 | 0.545 |
| Maximum Lindegaard ratio at any time | | | | | | |
| 37 | 3.29 | 38 | 3.11 | 1.061 | 0.905-1.243 | 0.461 |

**Table S4. Analysis of variance of maximum MCA flow velocity and maximum Lindegaard ratio recorded at any time**

| MCA Flow Velocity | | | | | | | |
| --- | --- | --- | --- | --- | --- | --- | --- |
|  | SFX-01 |  | Placebo |  |  |  |  |
| Time | n | GLS mean | n | GLS mean | Ratio of means | 95% CI | P value |
| Baseline | 44 | 61.8 | 40 | 64.9 |  |  |  |
| Days 3-4 | 19 | 82.3 | 16 | 99.0 | 0.832 | 0.673-1.028 | 0.088 |
| Days 5-6 | 34 | 97.7 | 26 | 100.9 | 0.968 | 0.814-1.151 | 0.711 |
| Days 7-9 | 38 | 105.5 | 40 | 109.1 | 0.966 | 0.824-1.133 | 0.672 |
| Days 10-14 | 28 | 115.9 | 23 | 99.3 | 1.166 | 0.971-1.400 | 0.099 |
| Days 15-21 | 17 | 90.8 | 17 | 86.5 | 1.050 | 0.844-1.306 | 0.662 |
| Days 22-28 | 7 | 57.7 | 7 | 72.6 | 0.794 | 0.577-1.093 | 0.153 |

**Table S5. Mixed Models Repeated Measures analysis of MCA flow velocity.**

| SFX-01 |  | Placebo |  |  |  |  |
| --- | --- | --- | --- | --- | --- | --- |
| n | Events | n | Events (%) | Odds Ratio | 95% CI | P value |
| Delayed Cerebral Ischaemia | | | | | | |
| 46 | 9 (19.6%) | 44 | 6 (13.6%) | 1.728 | 0.500-6.463 | 0.390 |
| Hypertensive therapy | | | | | | |
| 46 | 6 (17.4%) | 43 | 7 (16.3%) | 0.759 | 0.218-2.575 | 0.656 |

**Table S6. Logistic regression analysis of proportion of patients developing delayed cerebral ischaemia and receiving hypertensive therapy.**

|  | SFX-01 | Placebo |  |  |  |
| --- | --- | --- | --- | --- | --- |
| Time | n | n | Odds Ratio | 95% CI | P value |
| Day 7 | 45 | 43 | 1.352 | 0.589-3.134 | 0.477 |
| Day 28 | 43 | 41 | 1.346 | 0.583-3.129 | 0.487 |
| Day 90 | 43 | 40 | 1.598 | 0.699-3.704 | 0.268 |
| Day 180 | 42 | 38 | 1.647 | 0.721-3.821 | 0.237 |

**Table S7. Proportional odds logistic regression analysis for modified Rankin Score in the per protocol population. Odds ratios >1 favour SFX-01.**

|  | SFX-01 | Placebo |  |  |  |
| --- | --- | --- | --- | --- | --- |
| Time | n | n | Odds Ratio | 95% CI | P value |
| Day 28 | 42 | 39 | 0.979 | 0.422-2.263 | 0.960 |
| Day 90 | 39 | 40 | 0.917 | 0.406-2.068 | 0.834 |
| Day 180 | 42 | 38 | 1.263 | 0.560-2.868 | 0.574 |

**Table S8. Proportional odds logistic regression analysis for extended Glasgow Outcome Scores in the per protocol population. GOSE scores were reversed prior to analysis so the odds of a better response was being modelled. Odds ratios >1 favour SFX-01.**

| SFX-01 | Placebo |  |  |  |
| --- | --- | --- | --- | --- |
| n | n | Odds Ratio | 95% CI | P value |
| SF-36 Physical Health | | | | |
| 38 | 39 | -0.539 | -4.862-3.784 | 0.850 |
| 36 | 32 | -0.010 | -6.606-6.587 | 0.540 |
| 41 | 35 | 2.270 | -3.566-8.107 | 0.521 |
| SF-36 Mental Health | | | | |
| 38 | 39 | -2.208 | -7.771-3.356 | 0.319 |
| 36 | 32 | -0.440 | -7.792-6.912 | 0.992 |
| 41 | 35 | -2.938 | -10.228-4.352 | 0.492 |

**Table S9. Analysis of SF-36 in the per protocol population. SF-36 was analysed using Van-Elteren’s test with four strata defined by age (above and below the median) and WFNS score (1-3 v 4-5). The treatment effect was described using an un-stratified Hodges-Lehmann (HL) estimate of median difference together with its 95% confidence interval.**

|  | SFX-01 | Placebo |  |  |  |
| --- | --- | --- | --- | --- | --- |
| Time | n | n | Odds Ratio | 95% CI | P value |
| Day 28 | 42 | 41 | 0.667 | 0.295-1.487 | 0.323 |
| Day 90 | 39 | 34 | 0.811 | 0.348-1.882 | 0.626 |
| Day 180 | 41 | 36 | 1.082 | 0.464-2.525 | 0.855 |

**Table S10. Proportional odds logistic regression analysis for the SAHOT in the per protocol population. Odds ratios >1 favour SFX-01.**
